# Fluorescent Marinoquinoline Derivative as Inhibitors of *Plasmodium falciparum*: SAR Analysis, Mode of Action and In Vivo Studies

**DOI:** 10.1021/acs.jmedchem.5c00138

**Published:** 2025-09-30

**Authors:** Patricia Santos Barbosa, Guilherme Eduardo Souza, Sarah El Chamy Maluf, Vinícius Bonatto, Caio Silva Moura, Giovana Rossi Mendes, Talita Alvarenga Valdes, Yasmin Annunciato, Barbara dos Santos Rossetto, Priscilla Dantas de Souza Ventura, Gilberto Gaspar Duarte Ortin, Wellington da Silva, Marcelo Yudi Icimoto, Amália dos Santos Ferreira, Fabio C. Cruz, Carolina B. G. Teles, Dhelio B. Pereira, Gustavo Capatti Cassiano, Sofia Santana, Miguel Prudêncio, Camila S. Barbosa, Igor M. R. Moura, Renan Marcel Giampauli, Irene Layane De Sousa, Silvana Aparecida Rocco, Marcos L. Gazarini, Carlos Roque Duarte Correia, Anna Caroline Campos Aguiar, Rafael Victorio Carvalho Guido

**Affiliations:** † Chemistry Institute, 28132 University of Campinas (UNICAMP), Campinas, São Paulo 13083-970, Brazil; ‡ São Carlos of Physics Institute, 6396 University of São Paulo (USP), São Carlos, São Paulo 13566-590, Brazil; § Department of Biosciences, 28105 Federal University of São Paulo (UNIFESP), Santos, São Paulo 11015-020, Brazil; ∥ Department of Biophysics, 58804 Federal University of Sao Paulo, (UNIFESP), Escola Paulista de Medicina, São Paulo, São Paulo CEP 04023-062, Brazil; ⊥ Leishmaniasis and Malaria Bioassay Platform, Oswaldo Cruz Foundation, Porto Velho, Rondônia 76812-245, Brazil; # Department of Pharmacology, Federal University of São Paulo (UNIFESP), Escola Paulista de Medicina, São Paulo, São Paulo 04023-062, Brazil; ¶ Research Center in Tropical Medicine of Rondônia, Porto Velho, Rondônia 76812-245, Brazil; ∇ Global Health and Tropical Medicine (GHTM), Associate Laboratory in Translation and Innovation Towards Global Health (LA-REAL), Instituto de Higiene e Medicina Tropical, (IHMT), Universidade NOVA de Lisboa (UNL), Lisbon 1099-085, Portugal; ○ Gulbenkian Institute for Molecular Medicine, Lisboa 1649-035, Portugal; ⧫ 37809 Faculdade de Medicina da Universidade de Lisboa, Lisboa 1649-028, Portugal; †† Department of Microbiology, Immunology and Parasitology, Federal University of São Paulo (UNIFESP), Escola Paulista de Medicina, São Paulo, São Paulo 04023-062, Brazil; ‡‡ Brazilian Biosciences National Laboratory and Brazilian Center for Research in Energy and Materials (LNBio-CNPEM), Campinas, São Paulo 13083-100, Brazil

## Abstract

We present insights into the mechanism of action of marinoquinolines (MQ), a novel class of lead candidates. Using a divergent synthetic approach, we developed a series of 20 new analogues with fluorescence properties. Structure–activity relationships analysis identified **19** as an attractive compound showing a combination of favorable in vitro (IC_50_^3D7^ = 0.28 μM; CC_50_^HepG2^ = 53 μM), ex vivo (EC_50_^*Pf*^ = 1.2 μM; EC_50_^*Pv*^ = 0.53 μM), in vivo (3 × 50 mg/kg oral dose resulted in a 96% reduction in parasitemia in *Plasmodium berghei*-infected mice), physicochemical (Sol_7.4_ = 171 μM; LogD_7.4_ = 3.9), and pharmacokinetic (P_app = 9.4 × 10^–6^ cm/s, human Cl_int_^hep,mic^ = 0.61–0.68 μL min^–1^ mg^–1^) properties. Compound **19** selectively accumulates in infected erythrocytes, enters the digest vacuole and inhibits *Plasmodium falciparum* proteolytic activity, suggesting that MQs act as protease inhibitors. These findings strengthen the evidence that MQs are promising lead candidates for antimalarial drug discovery.

## Graphical abstract

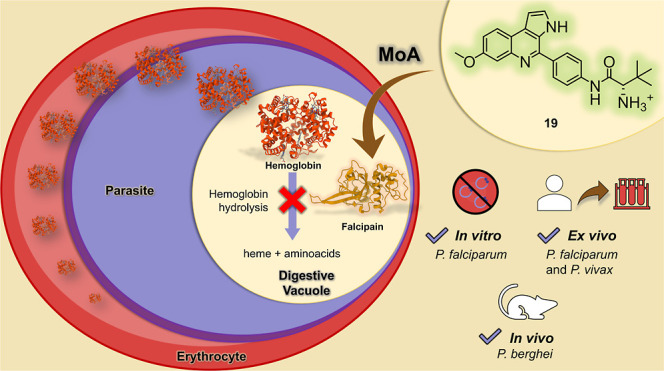

## 1 Introduction

Malaria, a parasitic disease caused by various *Plasmodium* species, including *Plasmodium falciparum* and *Plasmodium vivax*, poses a substantial global health burden.^1^ In 2023, the disease resulted in 263 million reported cases and an estimated 597,000 deaths worldwide. *P. falciparum* infections are particularly concerning due to their rapid clinical progression, often leading to severe malaria for untreated patients.^2^ The current gold standard for malaria treatment is Artemisinin-based Combination Therapies (ACTs).^1^ While ACTs have been crucial to malaria treatment success, delayed *P. falciparum* clearance following treatment with either artemisinin monotherapy or an ACT has become common in the Greater Mekong Subregion (GMS), which includes Cambodia, Thailand, Vietnam, Myanmar, and Laos.^3^ Delayed parasite clearance has also been reported in Africa, compromising the efficacy of antimalarial therapy and increasing the urgency for new drugs with novel mechanisms of action.^4,5^

The ongoing search for new antimalarial candidates has led to the discovery of natural pyrroloquinoline derivatives from marine gliding bacteria, *Rapidithrix thailandica* and *Ohtaekwangia kribbensis*, known as marinoquinolines (MQ).^6,7^ The fused pyrroloquinoline core with C-3a and C-9b positions blocked, along with C-4 substitution, prevents epoxidation via enamine during metabolism, reducing mutagenic potential.^8^ As a result, MQs show promise as antiplasmodial agents with greater metabolic stability and potentially lower toxicity than standard quinoline drugs.^9^ As part of our efforts to discover new lead MQ analogs, we advanced compound **1** (Figure 1) in a hit-to-lead investigation. This compound displayed potent in vitro activity against both *P. falciparum* sensitive and resistant strains (IC_50_^3D7^ = 39 nM; IC_50_^K1^ = 41 nM), fast-acting inhibition, and dual–stage activity targeting both blood and liver stages of parasite development. Additionally, compound **1** demonstrated a high selectivity index against HepG2 hepatic cells (SI > 6410), excellent tolerability in mice, and oral efficacy at 50 mg/kg in a *Plasmodium berghei* mouse malaria model, achieving a 62% reduction in parasitemia on day 5 postinfection.^9^ Despite these promising features, certain limitations as poor physicochemical properties, including low solubility and high lipophilicity, and the presence of a *tert*-butyl carbamate group linked to potential genotoxicity, prevented further advancement of this molecule in the drug discovery pipeline and the full elucidation of its mode of action.

**Figure 1:**
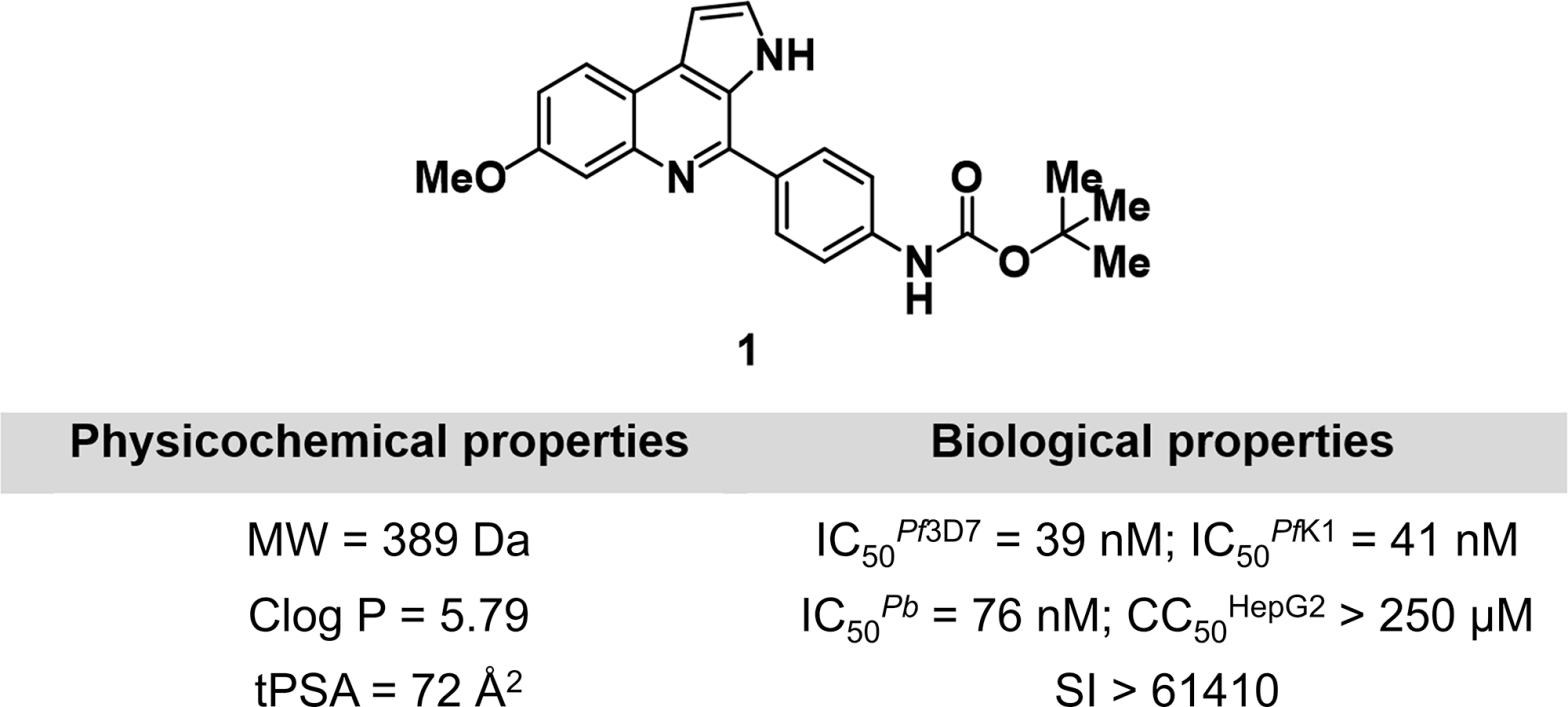
Chemical structure, antiplasmodial activity, and physicochemical properties of compound **1**.^9^

In this study, we report the synthesis of new MQ analogs with potent antiplasmodial activity, high selectivity, and enhanced lipophilicity and solubility. These compounds also exhibit fluorescent properties, enabling cell-based visualization and localization within specific organelles. This cell distribution suggested that MQ analogs act as potential protease inhibitors.

## 2 Results and Discussion

### 2.1 Synthesis and SAR Investigation

As previously observed in the discovery of compound **1**, SAR studies indicated that the presence of a methoxy group at the 7-position is required to maintain high potency, while the carbamate group could be replaced with related groups.^9^ In line with that, 20 new MQ analogs (**6–27**) were synthesized via bioisosteric replacement of the carbamate moiety with substituted amide groups.^10,11^ These new MQ analogs exhibited enhanced fluorescence, enabling its application in imaging studies of both *P. falciparum*-infected red blood cells (iRBCs) and hepatocellular carcinoma (HepG2) cells.

The synthesis of the new MQ series started with the construction of the MQ nucleus (Scheme 1). The Pictet–Spengler reaction between a previously synthesized methoxy-substituted arylpyrrole **2** and the commercially available 4-nitrobenzaldehyde **3** afforded the intermediate **4** in moderate yield. Subsequent reduction of the nitro group on **4** produced the aniline intermediate **5** in full conversion, which proceeded to the next step without further purification.

**Scheme 1:**
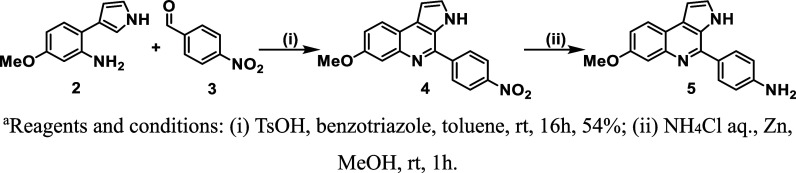
Construction of Marinoquinoline Core Through Pictet–Spengler Reaction

Amides **6–16** were prepared by coupling aniline **5** with various *N*-Boc-protected amino acids, including both l-series and unnatural amino acids, some of which were previously prepared or commercially available. HATU was used as coupling agent in moderate to excellent yields. The final reaction step involved Boc group removal via in situ generation of hydrochloric acid by the treatment of acetyl chloride in methanol. This strategy afforded the new MQ derivatives (**17–29**) as hydrochloric salts, in moderate to good yields (Scheme 2). Therefore, using both natural and unnatural amino acids in the amide formation step, we successfully synthesized a series of 20 compounds to enable SAR analysis.

**Scheme 2:**
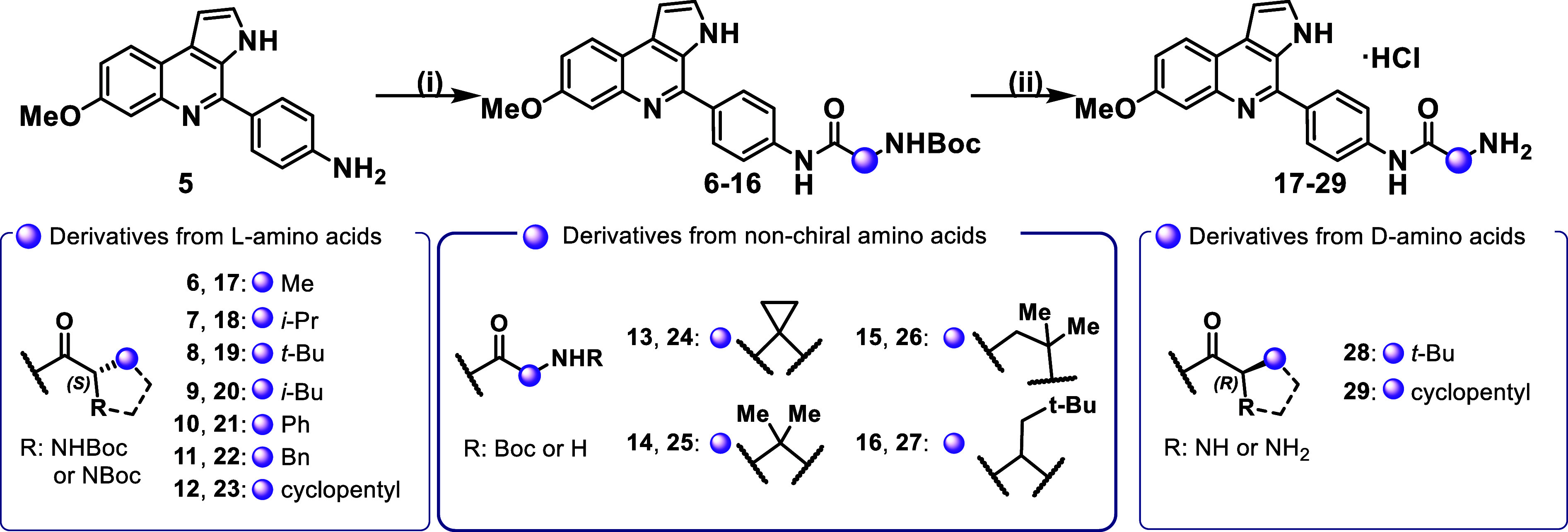
Synthesis of the Analogs Containing the Amide Side-chain

a Reagents and conditions: (i) *N*-Boc-amino acid, HATU, DIPEA, DMF, 50 °C, 4 h (58–91%); (ii) AcCl, MeOH, 0 °C-rt, 24 h (39–68%).

Incorporating the amino acid side chains into the MQ structure allowed us to evaluate the effects of steric volume and stereogenic centers within the series. SAR analysis is essential in identifying a lead candidate or a chemical probe.^12^ Our previous investigation on the SAR of MQ series emphasized the importance of a bulky group, such as a *tert*-butyl group, at the carbamate substituent to enhance compound activity.^9^ However, bulky groups can negatively impact physicochemical properties, reducing water solubility and increasing lipophilicity. Thus, achieving a balance among potency, selectivity, and physicochemical properties is crucial for drug or probe design.^12^ Antiplasmodial activity of MQ derivatives **6–29** was tested in vitro against the *P. falciparum* 3D7 strain, while cytotoxic activity was assessed against HepG2 cells (Figures S1 and S2). Physicochemical properties were calculated using Optibrium StarDrop software. MQ derivatives synthesized with l-amino acids (compounds **6–12**) demonstrated low cytotoxicity on HepG2 cells (CC_50_ > 50 μM) and excellent selectivity indices (SIs value of 50 to >975) (Table 1). Compounds **6** (IC_50_ = 2.1 μM) and **7** (IC_50_ = 4 μM), l-Ala and l-Val analogs, respectively, showed inhibitory activity in the low micromolar range, while compounds **10** (IC_50_ = 0.7 μM) and **11** (IC_50_ = 0.41 μM), featuring phenyl and benzyl substituents, respectively, exhibited submicromolar potency against the *P. falciparum* 3D7 strain. These findings suggest that steric volume positively impacts the inhibitory activity of *N*-protected l-amino acid derivatives **6–11**. However, the most potent analogs, such as **10** and **11**, which have bulky substituents, showed poor predicted physicochemical properties (i.e., cLogD_7.4_ > 4.5 and cLogS_7.4_ ≈ −0.4, Table 1), which are unfavorable for series development. Exploring alternative amino acid side chains with steric constriction but reduced steric volume led to compound **12**, a 5-membered ring derived from l-proline, which exhibited poor inhibitory activity under assay conditions (IC_50_ > 10 μM).

**Table 1:**
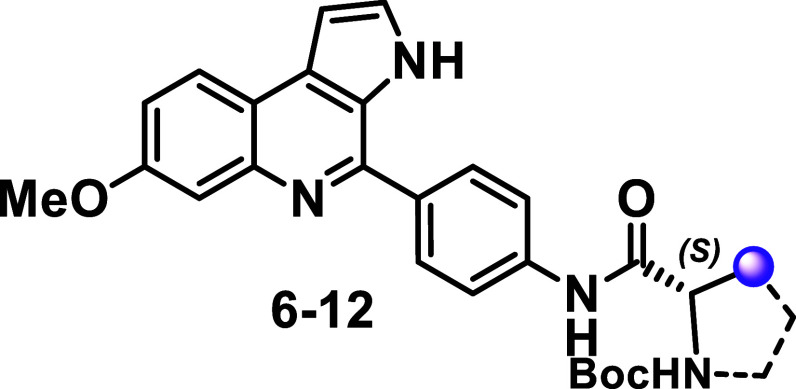

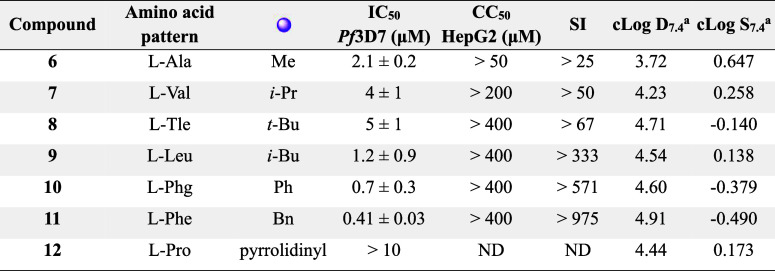
Antiplasmodial, Cytotoxic Activity, and Physicochemical Data of l-series of *N*-Boc-Protected-Amides MQ Analogs

a Optibrium StarDrop was used for predictions; SI = selectivity Index. ND = not determined.

Subsequently, we investigated the effect of nonchiral amino acids on antiplasmodial activity (compounds **13–16**, Table 2). In general, nonchiral amino acid substituents were tolerated and showed lower cytotoxicity. Compound **13**, which contains a 3-membered ring as a side chain, demonstrated low micromolar potency (IC_50_ = 4.1 μM) against the parasite and low cytotoxicity in liver cells (CC_50_ > 50 μM). Substitutions on the Cα amide bond, containing a stereogenic center in the MQ-based moieties from l-amino acids, yielded two new analogs, **14** and **15**. Compound **14** (IC_50_ = 1.9 μM) is an achiral analogue of compound **6** (IC_50_ = 7.4 μM), while compound **15** (IC_50_ = 0.4 μM) is a β-derivative of compound **7** (IC_50_ = 4 μM). Both compounds showed improved inhibitory activity, with 4- to 10-fold increases in potency compared to their chiral counterparts.

**Table 2:**
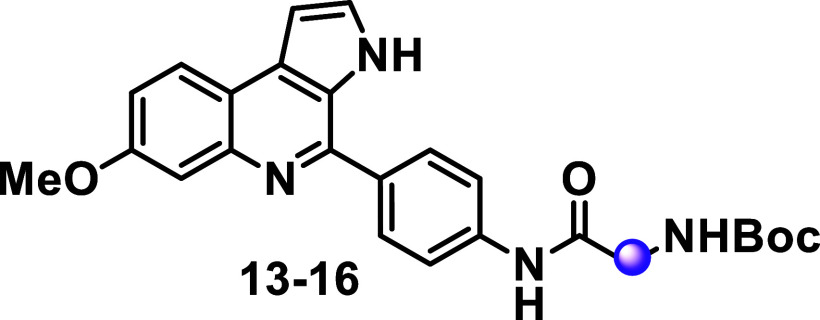

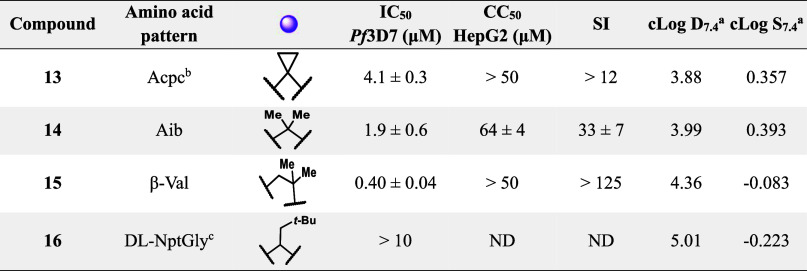
Antiplasmodial, Cytotoxicity Activity and Physicochemical Data of Non-Chiral *N*-Boc-Protected-Amides MQ Analogs^a^ ^,^ ^b^ ^,^ ^c^

a Optibrium StarDrop was used for predictions; SI = selectivity Index. ND = not determined.

b Abbreviation for 1-aminocyclopropanecarboxylic acid.

c Abbreviation for neopentylglycine.

Compound **15** was the most potent representative of this subset, exhibiting submicromolar potency against the parasite, low cytotoxicity in liver cells (CC_50_ > 50 μM), and a favorable selectivity index (SI > 125). Additionally, compound **15** (cLogD_7.4_ = 4.36) had a slightly lower cLogD_7.4_ value than compound **11** (cLogD_7.4_ = 4.91) but with comparable inhibitory activity. Compound **16** (IC_50_ > 10 μM), which possesses a stereogenic center at Cα and was synthesized as a racemate, was designed to assess the effect of a bulky side chain in the nonchiral series. The data indicated that the presence of a bulky substituent negatively impacted antiplasmodial activity (Table 2).

While significant progress has been made in enhancing inhibitory activity, the most potent compounds (**11** and **15**) exhibited high predicted LogD7.4 values (cLogD_7.4_ > 4). As a result, the next design cycle focused on replacing the carbamate group of parent compound **1** with amine-based substituents. MQ analogs containing amine groups derived from l-amino acids (compounds **17–23**, Table 3) were subsequently synthesized and evaluated. A steady increase in inhibitory potency was observed with an increase in the side-chain volume (e.g., compounds **17** to **19**). For instance, compound **17** (IC_50_ = 1 μM), a methyl analogue, displayed moderate activity, whereas compound **19** (IC_50_ = 0.28 μM), a *tert*-butyl analogue, emerged as the most potent derivative in the series. Substituents with larger groups, such as isobutyl (**20**) and phenyl (**21**) substituents, were tolerated, with the resulting compounds showing moderate activity (IC_50_ ∼1 μM). Compound **22** (IC_50_ = 0.47 μM), a benzyl analogue, demonstrated submicromolar potency, indicating that substituent size is not the sole factor influencing antiplasmodial activity. Compound **23**, an l-proline derivative, also displayed submicromolar potency (IC_50_ = 0.33 μM), which contrasts with the poor activity observed in its Boc-containing counterpart, compound **12** (IC_50_ > 10 μM). Overall, the amine group-containing MQ analogs showed low cytotoxic effects on HepG2 cells (CC_50_ > 10 μM) and acceptable selectivity indices (SI > 70). These findings suggest that the removal of the Boc group enhances both antiplasmodial activity and physicochemical properties. Notably, this modification introduces a basic nitrogen in the resulting amine moiety (p*K*_a_ > 7, according to DataWarrior), which may further benefit antiplasmodial activity.^13^ Even compounds with bulky substituents, such as *tert*-butyl (**19**) and benzyl (**22**), exhibited reasonable predicted lipophilicity (cLogD_7.4_ < 4) and solubility (cLogS_7.4_ ≥ 2).

**Table 3:**
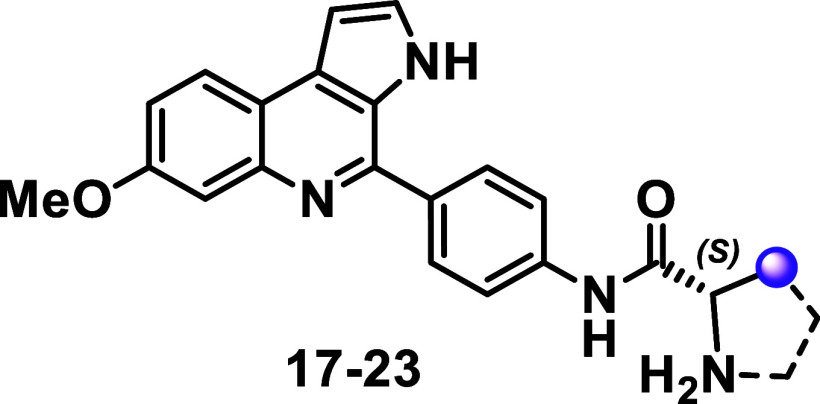

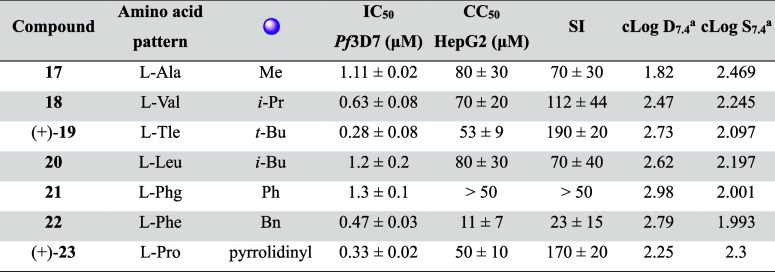
Antiplasmodial, Cytotoxicity Activity and Physical-Chemical Data of l-Series Amine-Containing Marinoquinoline Analogs^a^

a Optibrium StarDrop was used for predictions; SI = selectivity Index.

Amine-containing MQ analogs derived from nonchiral amino acids were also designed and evaluated (compounds **24–27**, Table 4). The inhibitory activity of the cyclopropane-containing derivative, compound **24** (IC_50_ = 7 μM), was lower than that of its precursor, compound **13** (IC_50_ = 1 μM, Table 2). In contrast, removing the Boc group from analogs **14–16** improved antiplasmodial activity (IC_50_ = 0.3–0.63 μM) while maintaining low cytotoxic effects (CC_50_ = 18–50 μM) in analogs **25–27**. Additionally, these analogs showed reasonable predicted cLogD_7.4_ (2–2.87) and cLogS_7.4_ (2.05–2.42) values. Amine-substituted analogs **25** (IC_50_ = 0.6 μM, cLogD_7.4_ = 2) and **26** (IC_50_ = 0.3 μM, cLogD_7.4_ = 2) demonstrated enhanced inhibitory potency, favorable lipophilicity, and improved selectivity (SI > 100), marking them as suitable candidates for further investigation. Compound **27** further exemplified the improvement in inhibitory activity and physicochemical properties achieved by introducing a basic nitrogen atom. The parent compound **16** (IC_50_ > 10 μM, Table 2) from the nonchiral *N*-Boc series was inactive; however, removing the Boc substituent produced the racemic analogue **27**, which displayed submicromolar potency (IC_50_ = 0.6 μM) along with attractive lipophilicity (cLogD_7.4_ = 2.87) and solubility (cLogS_7.4_ = 2.05) values.

**Table 4:**
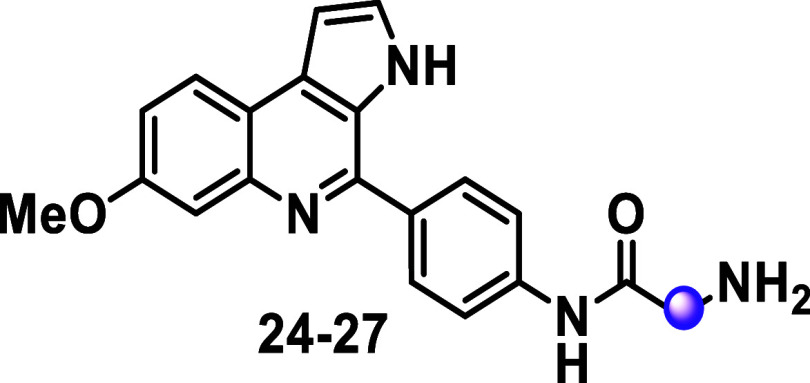

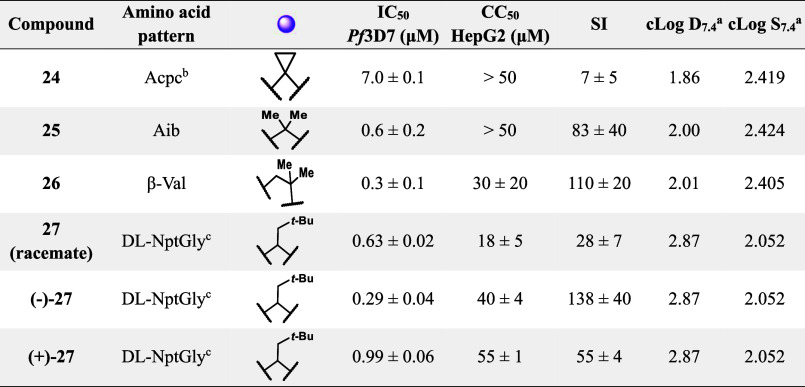
Antiplasmodial, Cytotoxicity Activity and Physicochemical Data of Non-Chiral Alfa-Amino Amide Marinoquinoline Analogs^a^ ^,^ ^b^ ^,^ ^c^

a Optibrium StarDrop was used for predictions; SI = selectivity Index. ND = not determined.

b Abbreviation for 1-aminocyclopropanecarboxylic acid.

c Abbreviation for neopentylglycine.

Given that compound **27** was obtained as a racemic mixture, biological evaluation of its pure enantiomers was investigated. However, a suitable chiral HPLC condition to separate the enantiomers of compound **27** directly was not identified. As an alternative, we achieved the chiral resolution of its intermediate, compound **16**, using a Daicel CHIRALPAK IB column with 20% isopropanol in hexanes as the mobile phase. Each enantiomer of **16** then underwent a subsequent Boc removal step to yield the pure enantiomers of **27**. Notably, enantiomer (−)-**27** (IC_50_ = 0.29 μM) demonstrated 3-fold greater antiplasmodial activity than enantiomer (+)-**27** (IC_50_ = 0.99 μM) (Table 4). Both enantiomers exhibited low cytotoxicity in HepG2 cells, with reasonable selectivity indices (SI values of 130 and 55, respectively).

Given the significant impact of the stereogenic center on the biological activity of **27**, we investigated the antiplasmodial activity of the two most potent representatives of the amine-containing MQ analogs. Consequently, the pure enantiomers of compounds **19** and **23** were obtained and evaluated (Table 5). The d- and l-series enantiomers exhibited comparable inhibitory potencies, suggesting that the presence of a stereogenic center is not critical for their inhibitory activity. Overall, the bioisosteric replacement strategy and Boc group removal led to the discovery of new MQ analogs with submicromolar potency, favorable selectivity, and improved lipophilicity and solubility. These properties make the new MQ analogs attractive candidates for mode of action studies.

**Table 5:**
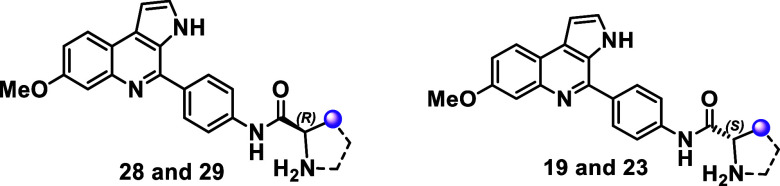

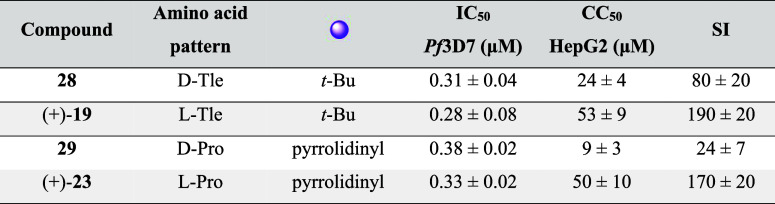
Antiplasmodial and Cytotoxicity Activity of d- and l Amino-Acids-Containing Marinoquinoline Analogs^a^

a SI = selectivity index. *Compounds **19** and **23** were added to the table for comparison reasons.

### 2.2 In Vitro Physicochemical and ADME Properties Assessment of 19

We experimentally assessed physicochemical (e.g., solubility, stability, logD_7.4_) and pharmacokinetic (e.g., metabolic stability, plasma protein binding, stability in human plasma, and permeability) properties of the most potent compound of the MQ series, compound **19** (Table 6). The kinetic solubility of **19** was investigated at 25 °C under three physiologically relevant pH conditions (1.7, 7.4, and 8.9), simulating the gastric, plasma, and intestinal environments, respectively. Solubility was measured at 0 and 1.5 h. Compound **19** exhibited the highest soluble fraction (>95%) at pH 1.7 at both time points, indicating that the compound is highly soluble under acidic conditions. In contrast, reduced solubility at both time points was observed at neutral (pH 7.4–80%) and basic pH (pH 8.9–65%). The positive control, alprenolol, showed high solubility across all conditions (Table S1 and Figure S3). In terms of absolute concentration, compound **19** showed solubility values of 207, 171, and 138 μM at pHs 1.7, 7.4, and 8.9, respectively (Table 6). These data indicate that **19** presents a pH-dependent solubility profile, with greater solubility in acidic environments. This behavior is consistent with the physicochemical properties of weakly basic compounds, which tend to ionize and dissolve more readily under acidic conditions.

**Table 6:** Experimentally Measured Physicochemical and Pharmacokinetic Properties of **19**

property	**19**
solubility (25 °C)	207 ± 10 μM at pH 1.7
	171 ± 4 μM at pH 7.4
	138 ± 4 μM at pH 8.9
chemical stability (37 °C)	171 ± 11 μM at pH 1.7
	213 ± 14 μM at pH 7.4
	142 ± 9 μM at pH 8.9
LogD_7.4_	3.9 ± 0.1
permeability (PAMPA model)	9.4 × 10^–6^ cm/s at pH 7.4
	3.1 × 10^–6^ cm/s at pH 5.5
plasma stability	81 ± 1%
human plasma protein binding (PPB)	% bound = 81 ± 1%
	% unbound = 19 ± 1%
half-life (*t*_1/2_)	mouse = 151 min
	rat = 182 min
	human = 204 min
CL int. microsomes	mouse = 0.92 μL min^–1^ mg^–1^
	rat = 0.76 μL min^–1^ mg^–1^
	human = 0.68 μL min^–1^ mg^–1^
CL int. hepatic	mouse = 3.73 μL min^–1^ mg^–1^
	rat = 1.37 μL min^–1^ mg^–1^
	human = 0.61 μL min^–1^ mg^–1^
CL hepatic	mouse = 0.69 mL min^–1^ kg^–1^
	rat = 0.25 mL min^–1^ kg^–1^
	human = 0.11 mL min^–1^ kg^–1^

The chemical stability of **19** was evaluated at 37 °C under three physiological pH conditions (1.7, 7.4, and 8.9), and solubility was monitored at 0, 1.5, and 24 h. The positive control, alprenolol, maintained high solubility and apparent stability under all conditions tested, with soluble fractions consistently above 100%. At time zero, compound **19** exhibited 100% solubility at pHs 1.7 and 7.4, while a lower soluble fraction was observed at pH 8.9 (62 ± 3%). After 1.5 h of incubation at 37 °C, a slight decrease in solubility was observed at pH 1.7 (95 ± 1%) and pH 7.4 (95 ± 4%), while solubility at pH 8.9 showed a modest increase (69 ± 3%). After 24 h, further reductions were observed across all pH values (86 ± 3% at pH 1.7, 87 ± 1% at pH 7.4, and 63 ± 3% at pH 8.9) (Table S2 and Figure S4). The absolute concentration values support these findings. The average concentrations of compound **19** over the three time points were 171, 213, and 142 μM at pHs 1.7, 7.4, and 8.9, respectively (Table 6). Taken together, compound **19** exhibited favorable kinetic solubility across physiological pH values, with minimal variation over 1.5 h at 25 °C, indicating low risk of immediate precipitation. Chemical stability assessments at 37 °C revealed consistent solubility retention over 24 h at pH 1.7 and 7.4, with a modest decrease under alkaline conditions (pH 8.9).

The lipophilicity of **19** was evaluated by experimental determination of the LogD at pH 7.4 using the classical shake-flask method.^14^ Tolbutamide and ketoconazole were used as controls (Table S3 and Figure S5). Compound **19** exhibited a LogD_7.4_ of 3.9 ± 0.1 (Table 6), slightly higher than that of ketoconazole (3.67 ± 0.05), and one log unit greater than the predicted LogD_7.4_ (2.7) by Optibrium StarDrop. This result indicates that **19** shows high lipophilicity but decreased compared to the parent compound **1**. Overall, the assessed physicochemical properties of **19** indicated that the inhibitor exhibited moderate aqueous solubility, chemical stability across physiological pH values, and a lipophilic character consistent with favorable membrane permeability.

The PAMPA assay was employed to evaluate the passive permeability of **19** at two physiologically relevant pH conditions (5.5 and 7.4), with alprenolol used as a reference compound (Figure S6). Alprenolol exhibited high apparent permeability (P_app) at pH 7.4 (19.2 × 10^–6^ cm/s) and decreased permeability at pH 5.5 (1.6 × 10^–6^ cm/s) (Table 6), consistent with its well-established membrane diffusion properties. Compound **19** showed a pH-dependent permeability, as well, with a P_app of 9.4 × 10^–6^ cm/s at pH 7.4 and 3.1 × 10^–6^ cm/s at pH 5.5. Compounds are considered highly permeable when their apparent (Papp) exceeds 4.0 × 10^–6^ cm/s in the acceptor compartment.^15,16^ These findings suggest that **19** has moderate-to-high passive permeability under neutral conditions, which may contribute positively to its absorption and oral bioavailability.

The plasma stability and plasma protein binding (PPB) of **19** were evaluated and compared to the reference compound verapamil. Verapamil exhibited high plasma stability, with 93.2 ± 0.1% of the compound remaining intact. Regarding PPB, verapamil showed a higher extent of protein binding, with 93.2 ± 0.1% bound and an unbound fraction (fu) of 6.8 ± 0.2%, consistent with literature data for this compound.^17^ The percentage of compound **19** remaining in plasma was consistent across all time points, with an average of 81 ± 1%, indicating negligible degradation. In the PPB assay, compound **19** demonstrated extensive binding properties, with 81 ± 1% bound and 19 ± 1% unbound fraction (fu), indicating moderate-to-high protein binding (Table 6). These findings indicate that **19** is stable in plasma and exhibits a moderately high degree of protein binding.

The metabolic stability of **19** was evaluated in liver microsomes from mouse, rat, and human using a protein concentration of 1 mg/mL and incubation times up to 60 min. Verapamil was used as positive control and was rapidly metabolized in all three species (*t*_1/2_ < 30 min; CL int. mic. = 20–30 μL min^–1^ mg^–1^; CL int. hep. = 18–120 μL min^–1^ mg^–1^; CL_hep_ = 1–8 mL min^–1^ kg^–1^), thereby confirming the validity of the assay (Figure S7 and Table S4).^18^ By contrast, compound **19** exhibited a slow rate of degradation in all species tested (Table 6). After 60 min, more than 60% of the parent compound remained in the incubation mixtures. The calculated half-lives were 151, 182, 204 min for mouse, rat, and human, respectively. These values were consistent with the low intrinsic microsomal clearance (CL_int mic._) values of 0.92, 0.76, and 0.68 μL min^–1^ mg^–1^ determined for mouse, rat, and human, respectively (Table 6). These findings indicated that **19** is highly metabolically stable in vitro, with minimal involvement of phase I hepatic enzymes. Furthermore, the calculated hepatic clearance (CL_hep_) for **19** was 0.69, 0.25, 0.11 mL min^–1^ kg^–1^ for mouse, rat, and human, respectively (Table 6). These values represent less than 1% of the hepatic blood flow for each species, classifying **19** as a low-clearance compound. In summary, **19** undergoes minimal hepatic metabolism across species, suggesting limited phase I hepatic clearance.

### 2.3 In Vitro, Ex Vivo, and In Vivo Assessments of **19**

To investigate the in vitro, ex vivo, and in vivo activity of the MQ analogs, compound **19** was selected as a representative analog of the series. Compound **19** demonstrated favorable biological, physicochemical, and biophysical (e.g., enhanced fluorescence) (Figure S8) properties for investigating its mode of action.^19^ In addition to the low cytotoxic effect on HepG2 cells (CC_50_ = 53 μM), compound **19** showed no hemolytic activity on fresh red blood cells at 10 μM after 24, 48, and 72 h of incubation (Figure S9).

The cross-resistance profile of compound **19** was assessed against a representative panel of *P. falciparum* strains, including six drug-resistant strains (Figure 2). The panel consisted of parasite strains resistant to clinically used drugs, such as endoperoxides (artesunate—ART), naphthoquinones (atovaquone—ATO), antifolates (pyrimethamine—PYR and cycloguanil—CG), 4-aminoquinolines (chloroquine—CQ), and piperazines (MMV692848, a phosphatidylinositol-4-kinase—PI4K—inhibitor). The strains included the *P. falciparum* 3D7 strain (sensitive to conventional antimalarials), K1 and Dd2 strains (multidrug-resistant to standard antimalarials, including CQ, CG, and PYR), TM90C6B strain (resistant to ATO, CQ, and PYR), IPC4912 (partially resistant to artemisinin derivatives, evidenced by slow parasite clearance in the Ring Survival Assay—RSA),^20^ and 3D7^R^_MMV848 strain (resistant to MMV692848).

**Figure 2:**
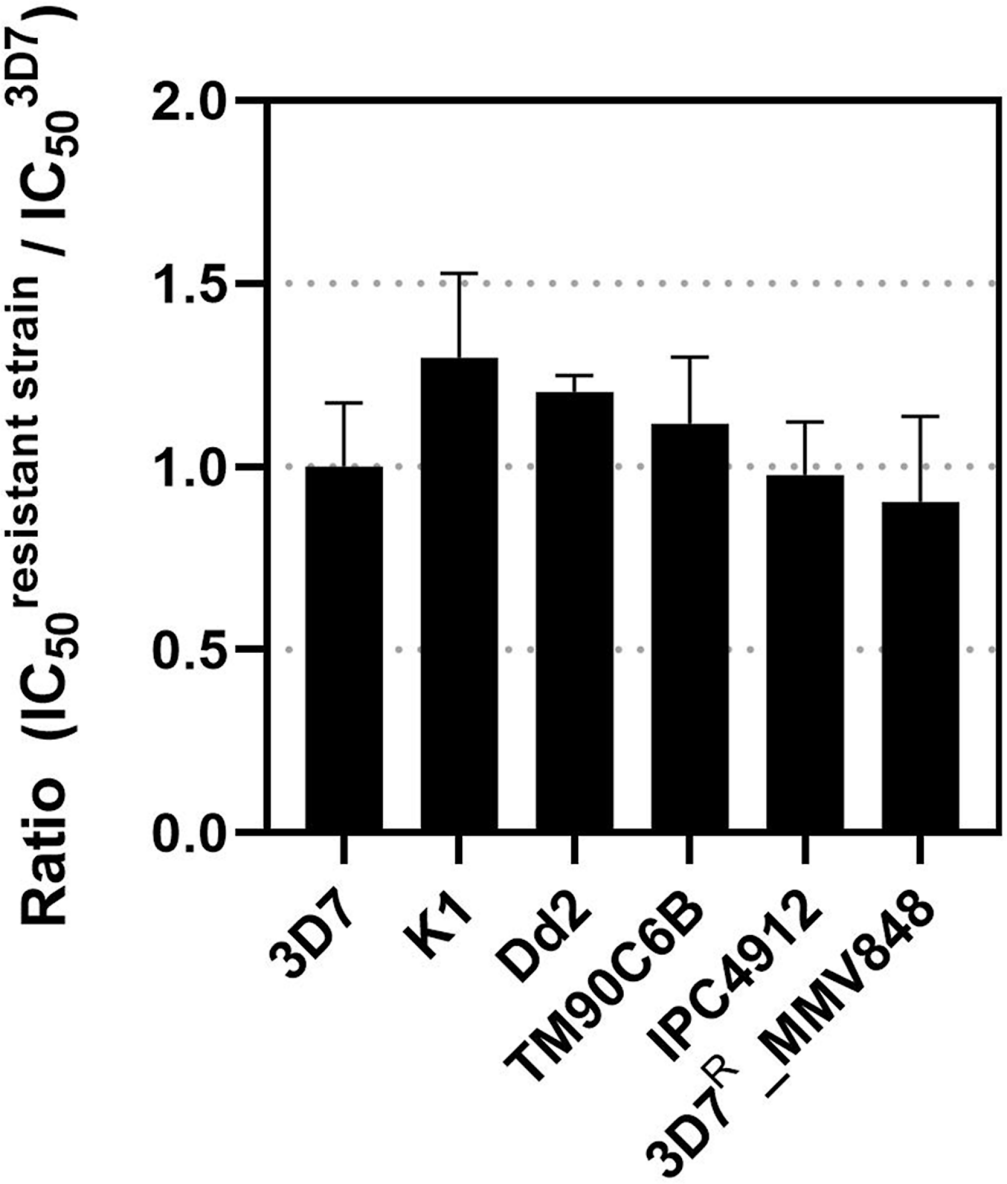
Resistance indices of compound **19** against a representative panel of multidrug-resistant *P. falciparum* strains. The results are displayed as ratios (IC_50_^resistant strain^/IC_50_^3D7^), calculated for 3D7 (sensitive strain), K1 (chloroquine-resistant), Dd2 (chloroquine- and mefloquine-resistant), TM90C6B (atovaquone-resistant), IPC4912 (partially artemisinin-resistant), and 3D7^R^_MMV848 (resistant to MMV692848) strains.

Standard antimalarial drugs were employed as positive controls to validate the resistance profile of each strain. Compound **19** exhibited comparable IC_50_ values against all the resistant parasites tested, with resistance index (RI) values ranging from 0.9 to 1.3 (Figure 2). No statistically significant differences were observed in potency between the sensitive and resistant strains. Furthermore, the calculated RI values were considerably lower than those of standard antimalarials, to which the strains employed are resistant. A compound is considered a cross-resistance inhibitor if it shows a shift greater than 5-fold in IC_50_ values.^21,22^ Therefore, these findings strongly suggest that **19** is a potent inhibitor of resistant *P. falciparum* strains and does not exhibit cross-resistance with standard antimalarials.

To further validate the antiplasmodial activity of the MQ series, compound **19** was subjected to an ex vivo drug susceptibility study using field isolates of *P. falciparum* and *P. vivax* from an endemic area of the Brazilian Amazon rainforest (Porto Velho, Rondônia State).^23^ These Brazilian field isolates are known for their multidrug resistance profiles.^24^ The assay included patients monoinfected with either *P. vivax* (*n* = 10) or *P. falciparum* (*n* = 8), with CQ and ART used as positive controls for inhibition. The baseline characteristics of parasites in patients infection are summarized in Table S5. Median EC_50_ values for *P. vivax* and *P. falciparum* isolates are shown in Figure 3. In *P. vivax* isolates, CQ exhibited a median EC_50_ of 39 nM (range: 24 to 79 nM), and ART showed a median EC_50_ of 0.2 nM (range: 0.1 to 0.4 nM). Compound **19** displayed a median EC_50_ of 531 nM (range: 95 to 1770 nM) in *P. vivax* isolates. For *P. falciparum* isolates, CQ and ART had median EC_50_ values of 619 nM (range: 27 to 1250 nM) and 0.3 nM (range: 0.2 to 1.5 nM), respectively, while compound **19** showed a median EC_50_ of 1270 nM (range: 360 to 2580 nM). These findings indicate that the MQ analogue is active against circulating parasite strains, with inhibitory activities comparable to those observed in laboratory strains. Notably, compound **19** exhibited inhibitory potency against *P. vivax*, a clinically relevant *Plasmodium* species, comparable to *P. falciparum* isolates.

**Figure 3:**
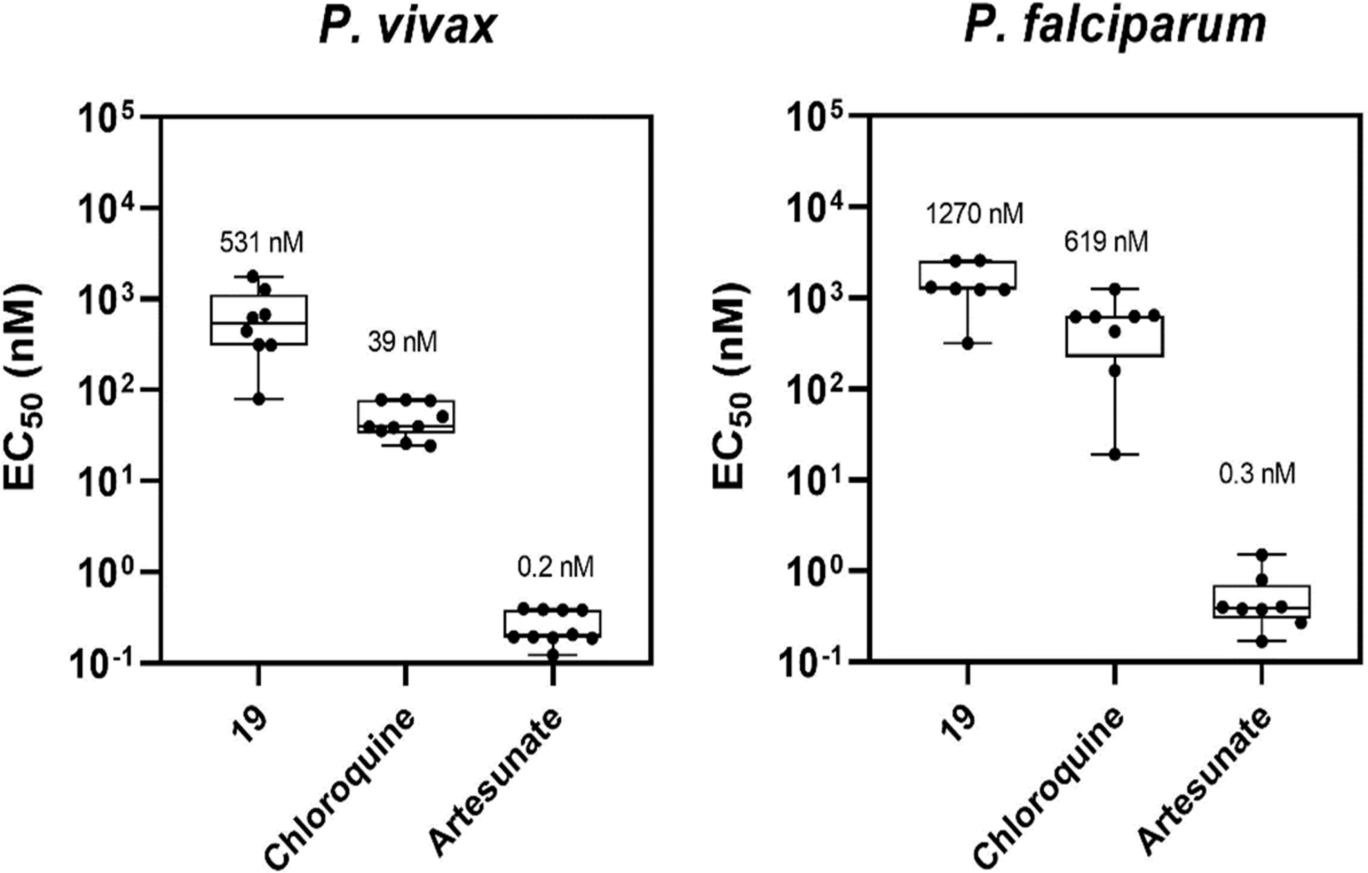
Median EC_50_ values of **19**, CQ, and ART (positive controls for inhibition) in *P. vivax* and *P. falciparum* field isolates.

To assess whether the structural modification used to generate **19** affected its speed-of-action, a time-dependent assessment was conducted at 24, 48, and 72 h of exposure. IC_50_ values for each time point were measured and compared to the 72 h IC_50_ to determine whether the compound acted as a fast- or slow-acting inhibitor. Fast-acting inhibitors maintain consistent IC_50_ values across time points, while slow-acting inhibitors show increased activity at later time points. Additionally, the morphological development of the parasite was monitored alongside IC_50_ assessments to confirm the speed-of-action. Data showed that parasites in the negative control group developed according to expected timelines (Figure 4). PYR, a slow-acting inhibitor used as a positive control, did not affect parasite development in the first 24 h (Figure 4C). As expected, PYR impaired parasite development and induced parasite death at 48 and 72 h, confirming its slow-acting inhibitory activity (Figure 4C). In contrast, CQ, a fast-acting control inhibitor, affected parasite development and caused parasite death within the first 24 h, as evidenced by parasite clearance (Figure 4B). Compound **19** similarly caused parasite death within the first 24 h, showing clearance of parasitemia at this time point, comparable to CQ (Figure 4A). Furthermore, IC_50_ values for compound **19** remained consistent over 24, 48, and 72 h (IC_50_ ratios ∼1, Figure 4A), which aligns with the profile of a fast-acting inhibitor, like CQ (Figure 4B). Therefore, the structural modification applied to develop a fluorescent chemical probe did not alter the speed-of-action of the MQ analogue, suggesting its suitability for mode-of-action investigations.

**Figure 4:**
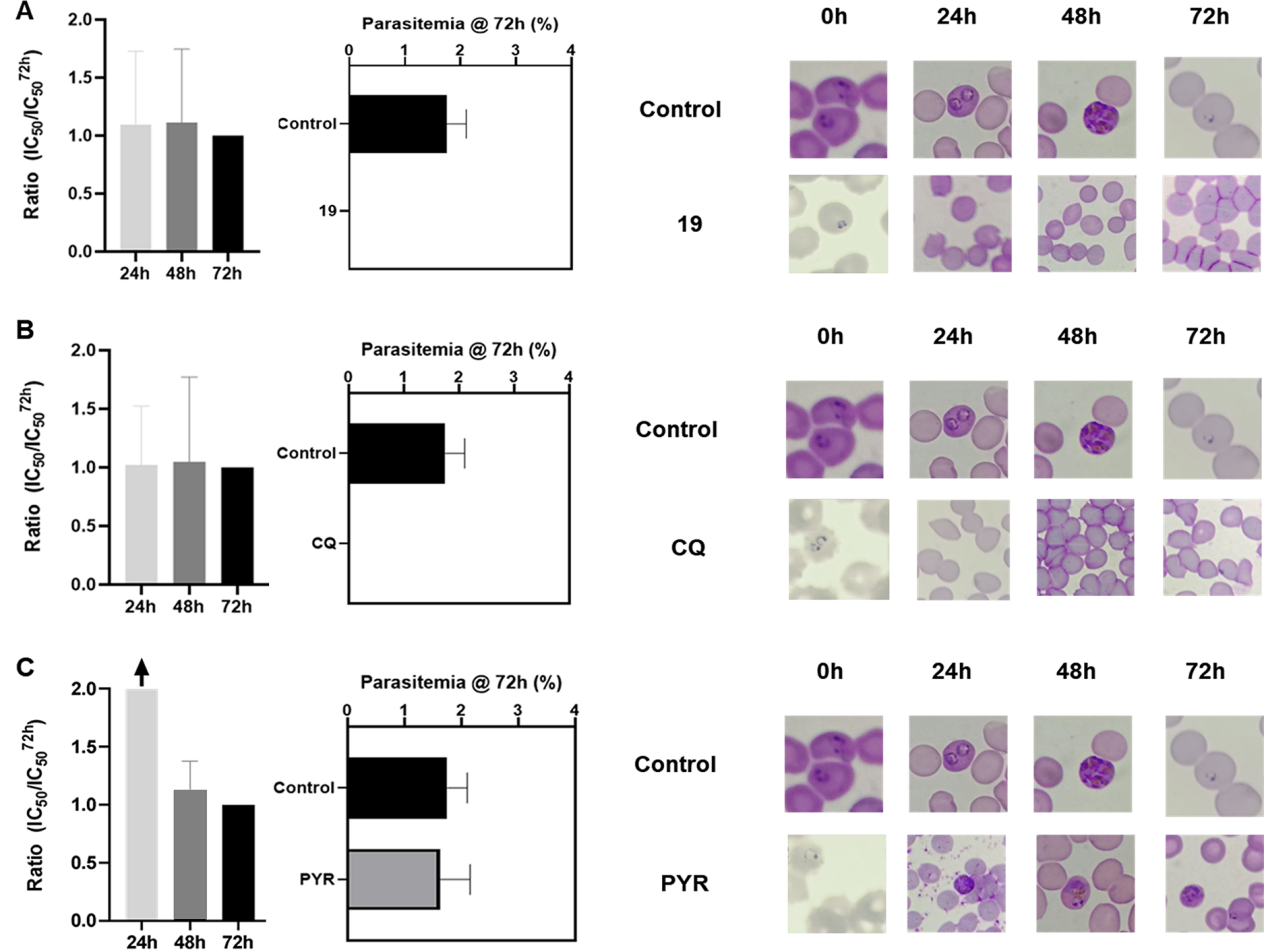
Speed of action assessment. Compound **19** (A), chloroquine (CQ, fast-acting control) (B), and pyrimethamine (PYR, slow-acting control) (C). IC_50_ values were determined at 24, 48, and 72 h. The morphological development of parasites was evaluated over time in *P. falciparum* cultures stained with Giemsa. Results were normalized to the IC_50_ values at 72 h. Data represent the mean IC_50_ ratios from three independent experiments.

We also investigated the combination of compound **19** with standard antimalarials including artesunate, chloroquine, atovaquone, pyrimethamine, and pyronaridine as a potential partners in combination therapies. Compound **19** was tested in combination with antimalarials at eight fixed ratios (1:0, 6:1, 5:2, 4:3, 3:4, 2:5, 1:6, 0:1). The Hand model was used to determine the additivity isobole.^25,26^ For each proportion of the **19**–antimalarial combination, fractional inhibitory concentration (FIC_50_) values, expressed as IC_50_ equivalents, were calculated (Figure 5A–E). Statistical analysis was performed to assess the effects of the combination. When no significant difference was found between the experimental values and the additivity isobole, the combination was classified as additive. Significant deviations from the isobole indicated either synergy (values below the isobole) or antagonism (values above the isobole). The combination of compound **19** with clinically used antimalarial drugs demonstrated antagonistic profiles (Figure 5A–E), as experimental data points (red dots and red shaded region) were positioned above the additivity curve (black line). Statistical analyses confirmed a significant deviation from the additivity isobole for each combination (*p*-values = 0.0018–0.0701), supporting the observed antagonistic interaction and suggesting that the use of **19** in combination therapies may not be pharmacologically justified.

**Figure 5:**
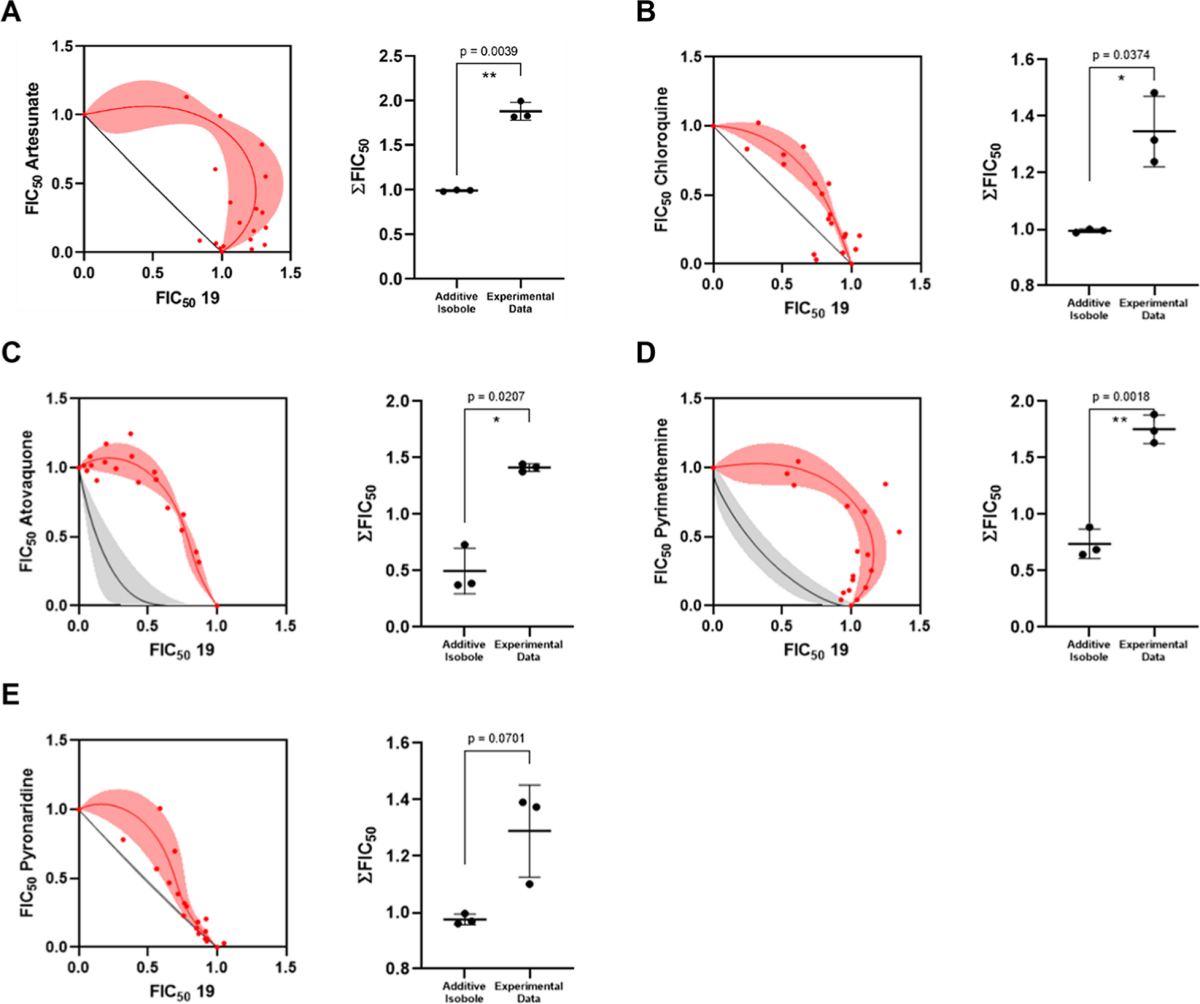
Evaluation of the combination of **19** with antimalarials. Isobolograms and statistical analysis for the combination of **19** with artesunate (A), chloroquine (B), atovaquone (C), pyrimethamine (D), and pyronaridine (E). The black lines represent the additivity curves, while the red dots and red shaded area denote the experimental data and standard deviation, respectively. The panels display the ∑FIC_50_ values derived from three independent experiments. A *p*-value < 0.05 indicates a statistically significant difference between the experimental data and the additivity isobole.

The liver-stage activity of **19** was assessed using a *P. berghei* hepatic infection model by measuring the parasite load of treated and control in Huh7 cells exposed to luciferase-expressing parasites.^27^ This stage precedes the symptomatic blood-stage infection, making compounds active against liver forms potentially useful for chemoprotection. Compound **19** showed concentration-dependent inhibition of hepatic infection, with an IC_50_ value of 3.8 μM and no toxicity against host cells (Figure S5).

Following promising in vitro and ex vivo results, the efficacy of compound **19** was evaluated in a murine malaria model. Compound **19** was administered orally at 50 mg/kg for three consecutive days postinfection. Parasitemia levels were monitored until day 11 (Figure 6 and Table 7), and survival rates were assessed up to day 30 post-treatment. CQ served as positive control at a dosage of 20 mg/kg for 3 days. CQ-treated animals exhibited no detectable parasitemia throughout the evaluation period (Figure 6B) and survived to the end of the experiment (Figure 6A). Compound **19** showed a substantial reduction in parasitemia, with decreases of 96, 58 and 68% on days 5, 8, and 11 postinfection, respectively (Figure 6B). Moreover, survival rates of the **19**-treated mice were significantly greater than those of the untreated control group (*p*-value < 0.05, Mann–Whitney test) (Figure 6A). This in vivo activity exceeded that observed for compound **1**, which reduced parasitemia by 62% on day 5 postinfection,^9^ confirming that the medicinal chemistry strategies to enhance the biological and physicochemical properties of **19** were successful in delivering an inhibitor with improved in vivo efficacy. Additionally, these findings indicate that the compound was well-tolerated, resulting in improved survival rates in the treated group compared to the untreated group (Figure 6A).

**Figure 6:**
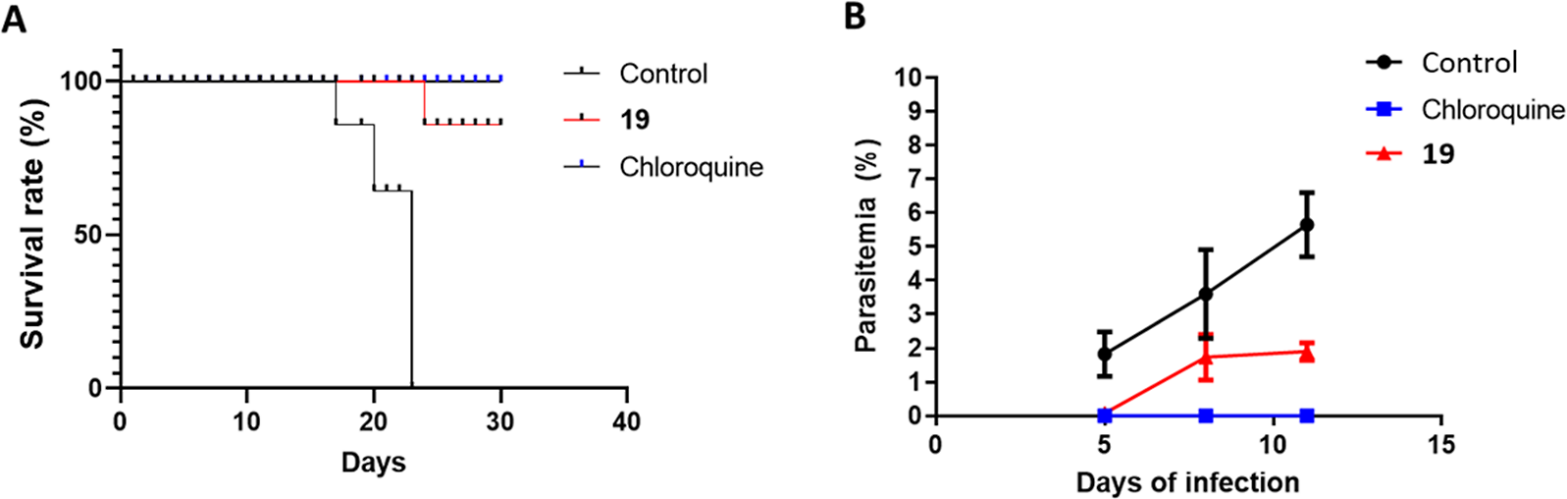
Antimalarial activity of compound 19 in mice infected with *P. berghei*. (A) In vivo survival following treatment with compound **19** (red line), chloroquine (CQ, blue line), and untreated control animals (black line). (B) Percentage of parasitemia on days 5, 8, and 11 postinfection. Treatment consisted of daily doses administered over three consecutive days (CQ at 20 mg/kg; compound **19** at 50 mg/kg).

**Table 7:** Parasitemia of Non-Treated Animals and Animals Treated With Chloroquine and **19**

	parasitemia ±SD (%)		
day	control	**19**	chloroquine
5	1.8 ± 0.7	0.08 ± 0.07	0 ± 0
8	4 ± 1	1.7 ± 0.7	0 ± 0
11	6 ± 1	1.9 ± 0.3	0 ± 0

### 2.4 Mode of Action Investigation

The initial strategy to investigate the mode of action of the MQ series involved generating resistant strains of the parasite for whole genome sequencing analysis (WGSA). For this purpose, we inoculated three culture flasks with 1 × 10^9^ *P. falciparum* parasites (Dd2 strain) per flask and exposed them to **19** at 3xIC_90_ concentration. Parasite clearance was observed on day 4, after which the inhibitor was removed, and cultures were monitored for recrudescence over 60 days. However, no recrudescence was observed for **19** during the 60 day experiment. This finding suggests that the MQ series has a low propensity to generate resistant strains, though it complicates efforts to determine the mechanism of action. The lack of resistance development may be linked to the fast-acting inhibitory activity of compound **19** and the absence of pre-existing resistance.^28^

As an alternative approach, we used confocal microscopy and biochemical assays to investigate the MQ series’ mode of action. Compound **19** exhibited fluorescence excitation and emission maxima at 380 and 460 nm, respectively, comparable to the DAPI/Hoechst dye spectrum (λ_max_^Ex^ = 405 nm and λ_max_^Em^ = 450–590 nm). This autofluorescence enabled direct visualization of its intracellular distribution in live *P. falciparum*-infected erythrocytes by fluorescence microscopy without the need for an external fluorophore.^29^ To perform this study, infected erythrocytes were incubated with **19** at a concentration of 10 μM (∼35xIC_50_). Compound **19** rapidly accumulated in *P. falciparum* and displayed diffusive cytoplasmic fluorescence in all infected red blood cells (iRBCs), with notably increased fluorescence intensity within the digestive vacuole (DV) of the parasites (Figure 7A,B). No labeling was observed in uninfected red blood cells, indicating selectivity for *P. falciparum*-iRBCs (Figure 7A,B).

**Figure 7:**
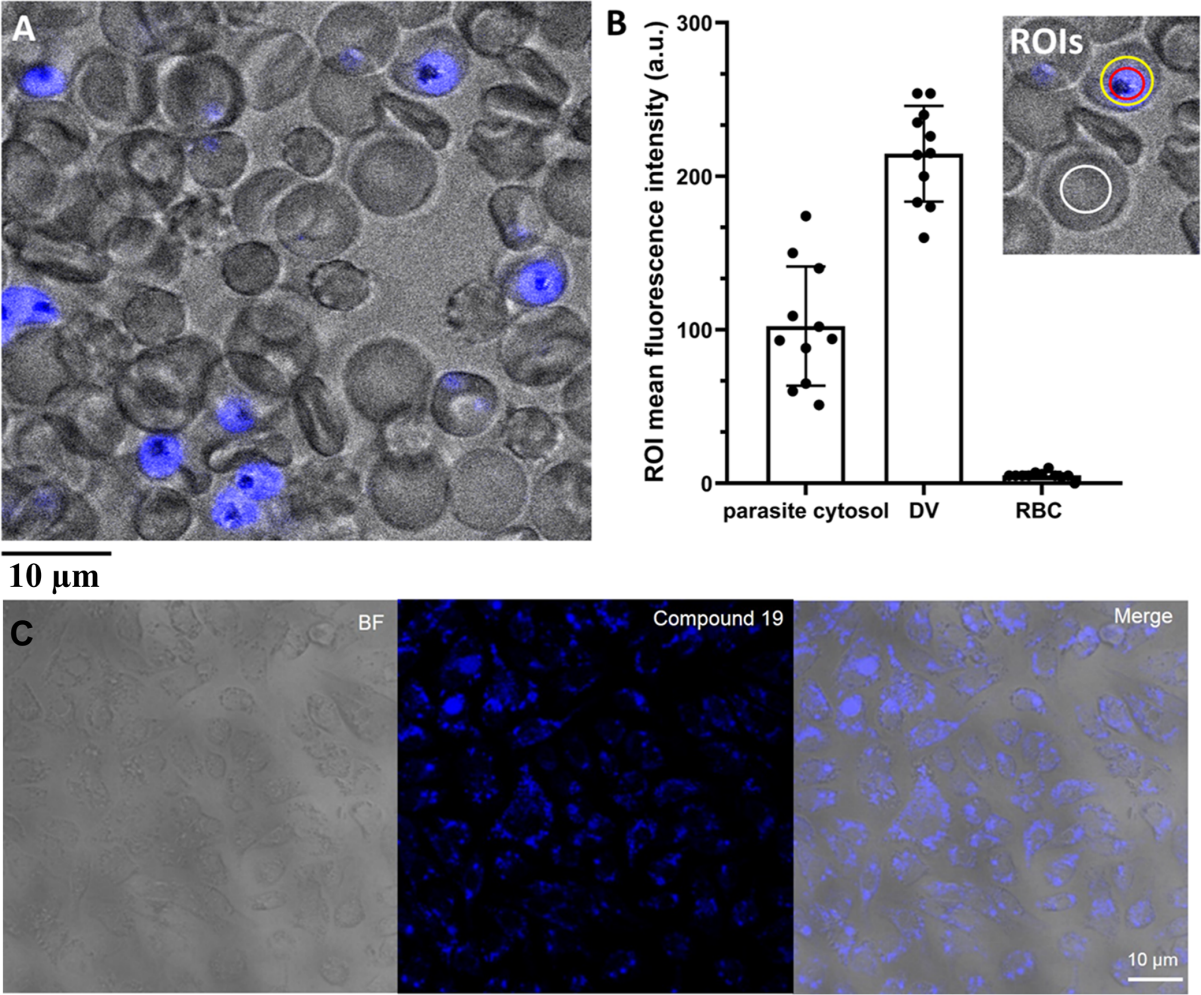
Intracellular distribution of compound 19. (A) Live-cell confocal microscopy of *P. falciparum*-infected erythrocytes. Compound **19** was visualized using its autofluorescence at 405 nm (excitation) and 450–490 nm (emission). Images were acquired with a TCS SP8 confocal microscope (Leica Microsystems, Germany) equipped with a 60 × 1.4 numerical aperture oil immersion lens. Scale bar: 10 μm. (B) Quantification of fluorescence intensity by region of interest (ROI) analysis in the digestive vacuole (DV), parasite cytoplasm, and red blood cell (RBC) cytoplasm. (C) Live-cell confocal microscopy of HepG2 cells showing **19** autofluorescence (excitation at 405 nm; emission at 450–490 nm). Scale bar: 10 μm.

To assess selectivity for *P. falciparum*-infected erythrocytes over mammalian cells, compound **19** was also tested in human hepatocarcinoma (HepG2) cells via confocal microscopy. Images confirmed uptake of the MQ analogue by HepG2 cells (Figure 7C), showing strong intracellular fluorescence. Although **19** was taken up by HepG2 cells, it demonstrated low cytotoxicity against this cell line (CC_50_ = 53 μM, Table 3).

Given the structural similarity between the pyrroloquinoline core of MQ and the quinoline ring of chloroquine (CQ), known to interact with the parasite’s digestive vacuole (DV), the interaction of **19** with the DV was evaluated using the lysosomotropic probe acridine orange (AO, Sigma-Aldrich). AO accumulates within acidic organelles, including the parasite’s digestive vacuole, and emits red fluorescence (λ_em_ > 560 nm). Fluorometric analyses indicated that **19** (at 40 μM) disrupted proton (H^+^) homeostasis, as shown by a significant increase in fluorescence post-treatment, comparable to the effect observed with CQ (at 40 μM), leading to reduced acidity within the organelle (Figure 8A). In contrast, artesunate and **12** (IC_50_ > 10 μM), an inactive MQ analog, did not induce AO mobilization from the DV, as no increase in fluorescence was observed following treatment. These findings suggest that **19** interacts with the DV (Figure 8A).^30^

**Figure 8:**
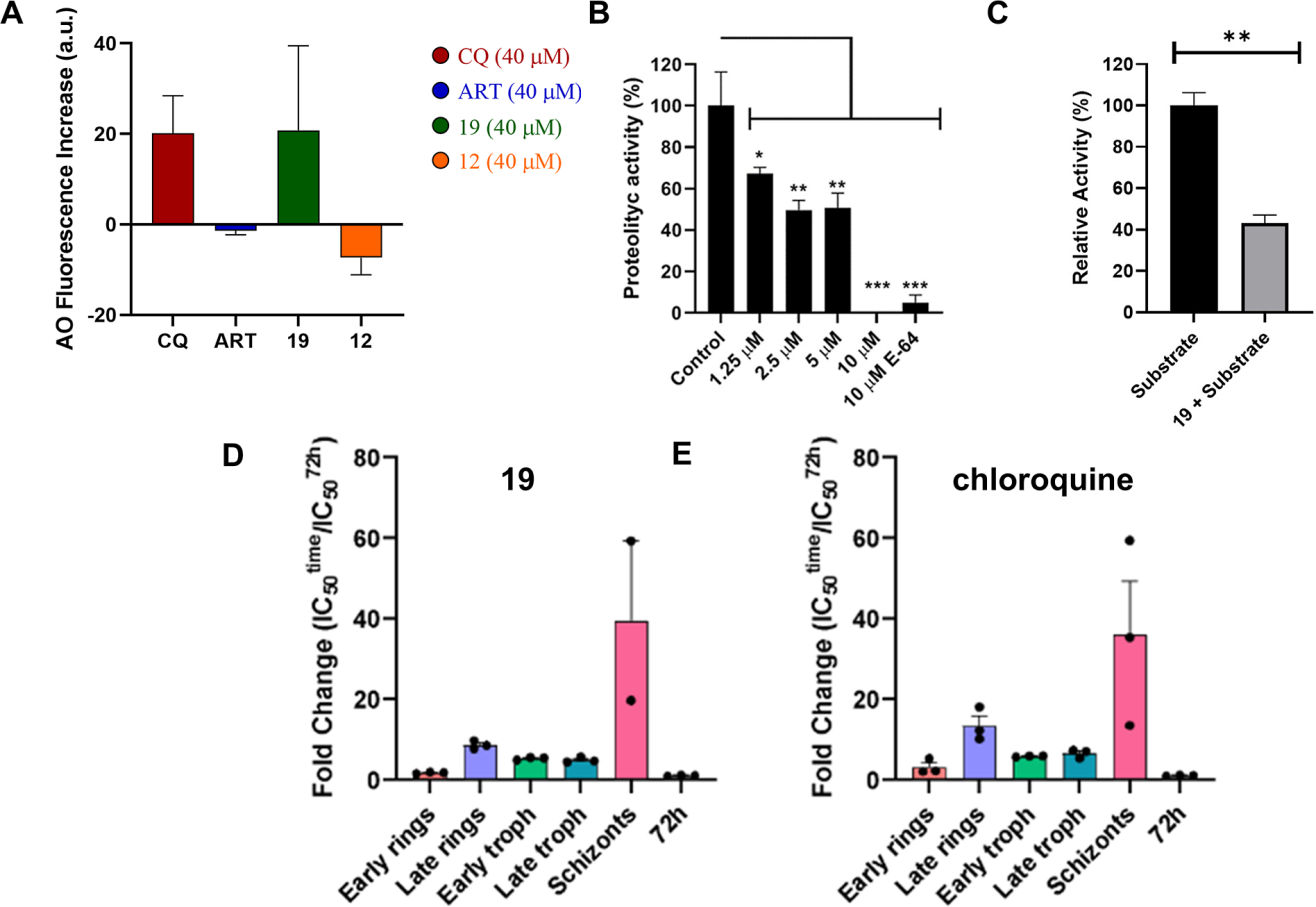
Effect of **19**, **12**, chloroquine (CQ) and artesunate (ART) on acridine orange (AO) mobilization from acidic compartments in isolated *P. falciparum* parasites, inhibition of *P. falciparum*proteases by **19**, and stage-specific activity. (A) Purified parasites were loaded with the lysosomotropic dye acridine orange (AO, 5 μM) and subsequently treated with **19** (40 μM), chloroquine (CQ, 40 μM), artesunate (ART, 40 μM), or **12** (inactive analog, 40 μM), during real-time fluorescence monitoring in a spectrofluorometer cuvette. The bar graph represents the change in fluorescence intensity (Δfluorescence) calculated as the difference between final and baseline fluorescence values after compound addition, expressed in arbitrary fluorescence units (AFU).^26^ *n* = 2 independent experiments. (B) Isolated parasites at the trophozoite stage were preincubated for 1 h with agitation in MOPS buffer (pH 7.4) in the presence of compound **19** at concentrations ranging from 1.25 to 10 μM. Cysteine protease activity was measured continuously by monitoring the hydrolysis of Z-Phe-Arg-AMC (10 μM) at 37 °C using a Hitachi F-7000 spectrofluorimeter (λ_ex_ = 380 nm; λ_em_ = 460 nm). Data was analyzed using one-way ANOVA followed by Bonferroni *posthoc* testing. (C) Inhibition of recombinant papain enzymatic activity by compound **19**. Papain activity was assayed in 100 mM sodium acetate buffer (pH 5.0) with Z-Phe-Arg-AMC substrate, in the absence or presence of compound **19** (10 μM). Statistical significance was calculated using a two-sided unpaired Student’s *t*-test between the indicated groups (**p < 0.016). Stage-specific activity of **19** (D) and chloroquine (control) (E) in highly synchronized *P. falciparum* cultures initiated at 0 h postinvasion (hpi). Parasites were treated at key developmental stages (early/late ring, early/late trophozoite, and schizont), and parasite viability was assessed.

Proteases are involved in multiple stages of the *Plasmodium* life cycle, playing essential roles from invasion to egress from host erythrocytes and hepatocytes. Thus, targeting proteolytic activity, particularly through the inhibition of parasite cysteine proteases, represents a promising approach for antimalarial drug discovery.^31^ In this context, the potential of **19** to inhibit cysteine proteases was investigated. The inhibition assay monitored the hydrolysis of the fluorogenic substrate Z-Phe-Arg-AMC, with E-64 serving as a positive control for cysteine protease inhibition.^32^ This method also allowed us to evaluate the impact of **19** on proteolytic activity in the parasite.

Compound **19** was tested at four concentrations (1.25–10 μM) and exhibited concentration-dependent inhibition of proteolytic activity (Figure 8B). At lower concentrations (1.25–5 μM), compound **19** inhibited 40% to 50% of proteolytic activity. At 10 μM, it completely abolished intracellular proteolytic activity, demonstrating inhibition comparable to E-64 (10 μM), a potent irreversible inhibitor of cysteine proteases.^33^ These findings corroborate our previous observation that the mechanism of action of MQs extends beyond the inhibition of hemozoin formation.^9^ A stage-specific assay indicated that **19** demonstrates pronounced inhibitory activity during both early and late ring and trophozoite stages, while exhibiting decreased effect at the schizont stage (Figure 8D). The pattern of inhibition is comparable to that observed with chloroquine (Figure 8E). These results agree with the expression profiles of cysteine proteases, which display maximal gene expression and enzymatic activity during the ring and trophozoite stages. To further evaluate the effect of compound **19** on cysteine protease enzymatic activity, a biochemical assay was conducted using recombinant papain and the Z-Phe-Arg-AMC substrate. Papain is a surrogate enzyme widely used in drug discovery campaigns to design potent and selective cysteine protease inhibitors.^34^ After a 10 min preincubation with compound **19** (10 μM) and papain, the substrate was added to the reaction mixture, and fluorescence release was monitored. Under these conditions, compound **19** reduced protease activity by 60% (Figure 8C), confirming that the MQ analogue acts as a cysteine protease inhibitor with moderate inhibitory activity.

The antagonistic effect of **19** when combined with artesunate (Figure 5A) suggests that the mode of action of **19** could be associated with the inhibition of parasitic protease activity involved in hemoglobin digestion. Artesunate relies on the release of free heme-iron to generate reactive oxygen species, which are crucial for its antimalarial effect. Consequently, the inhibition of proteases that sequentially hydrolyses hemoglobin into smaller components would prevent the release of free heme-iron, thereby reducing the efficacy of ACTs.^35,36^ To further explore this hypothesis, we investigated the effect of combining artesunate with E-64, a known cysteine protease inhibitor. As expected, the E64/artesunate combination exhibited an antagonistic profile (Figure S6), consistent with the antagonism observed for the **19**/artesunate pair. This finding aligns with previous work by Klonis et al.,^35^ who reported that inhibition of falcipain-2a (FP-2a), a major cysteine protease of the parasite, antagonized artemisinin’s action, reinforcing the hypothesis that the mechanism of action of **19** involves the inhibition of parasite proteases essential for hemoglobin digestion.

Inhibitors of *P. falciparum* proteases do not exhibit cross-resistance with Dd2 or W2 strains.^37–39^ Moreover, protease inhibitors display broad-spectrum effects, as proteases are essential for key parasitic processes, including hemoglobin digestion during blood-stage infection, liver-stage cell invasion, and the oocyst stage in the mosquito vector.^40^ Notably, cysteine protease inhibitors active against *P. falciparum* have also shown inhibitory activity against proteases from *P. vivax*.^41^ Furthermore, in vivo studies have demonstrated that inhibiting *P. falciparum* cysteine proteases can delay parasite growth or even cure *P. berghei*-infected mice.^42^ Collectively, these findings, along with the results presented here, suggest that the mechanism of action of the MQ series is related to the inhibition of parasitic proteases, particularly cysteine proteases.

### 2.5 Molecular Modeling Studies

Given that the substrate Z-Phe-Arg-AMC, used in our previous assays, is a standard substrate for the C1 family of cysteine proteases (e.g., human cathepsins B, L, K, S, and falcipains), and considering the evidence that **19** interacts with the DV of the parasite, we extended our investigation to computationally explore its interaction with two essential DV-acting cysteine proteases of the parasite involved in hemoglobin degradation: falcipain-2a (FP-2a) and falcipain-3 (FP-3).^43^

Docking studies of **19** in the active sites of FP-2a and FP-3 identified two reasonable binding poses (Figure 9). In the top-ranked pose, as scored by the ChemPLP function, the inhibitor predominantly bound to the S1/S1′ subpockets. In contrast, the second putative pose interacted with the S2 subpocket. The top-ranked pose undergoes π-stacking interactions with W206 and W208 of FP-2a and FP-3, respectively, along with hydrogen bonds between the inhibitor’s carbonyl group and the backbone carbonyl groups of N173 (FP-2a) and N175 (FP-3) (Figure 9A). In the S2 binding pose, the pyrroloquinoline core of **19** bound to a hydrophobic pocket spanning the S2 and S3 subsites, shared by both enzymes, and formed hydrogen bonds with the backbone carbonyl groups of G40/C80 and G42/C82 in FP-2a and FP-3, respectively (Figure 9B). To evaluate the stability of these binding modes, molecular dynamics (MD) simulations were performed for 300 ns on each pose in both enzymes (Figures S7 and S8). The top-ranked pose exhibited significant conformational shifts during the simulation, yielding multiple orientations that hindered the identification of a representative binding mode (Figures S9–S12). Conversely, cluster analysis of the simulation trajectory suggested that the S2 binding pose is more likely to occur. In this conformation, the pyrroloquinoline core of compound **19** interacted with residues from the S1 and S3 subpockets, while the methoxy group consistently occupied the hydrophobic S2 subpocket (88% of the simulation) (Figure 9C). The ability to bind to the S2 pocket is critical for inhibiting members of the C1 family of cysteine proteases.^44^ For instance, peptides with leucine or similar substituents at the P2 position are known to enhance inhibition of FP-2a and FP-3.^45^ Interestingly, our prior studies with MQ derivatives demonstrated that removal of the methoxy substituent led to less potent or inactive compounds.^9^

**Figure 9:**
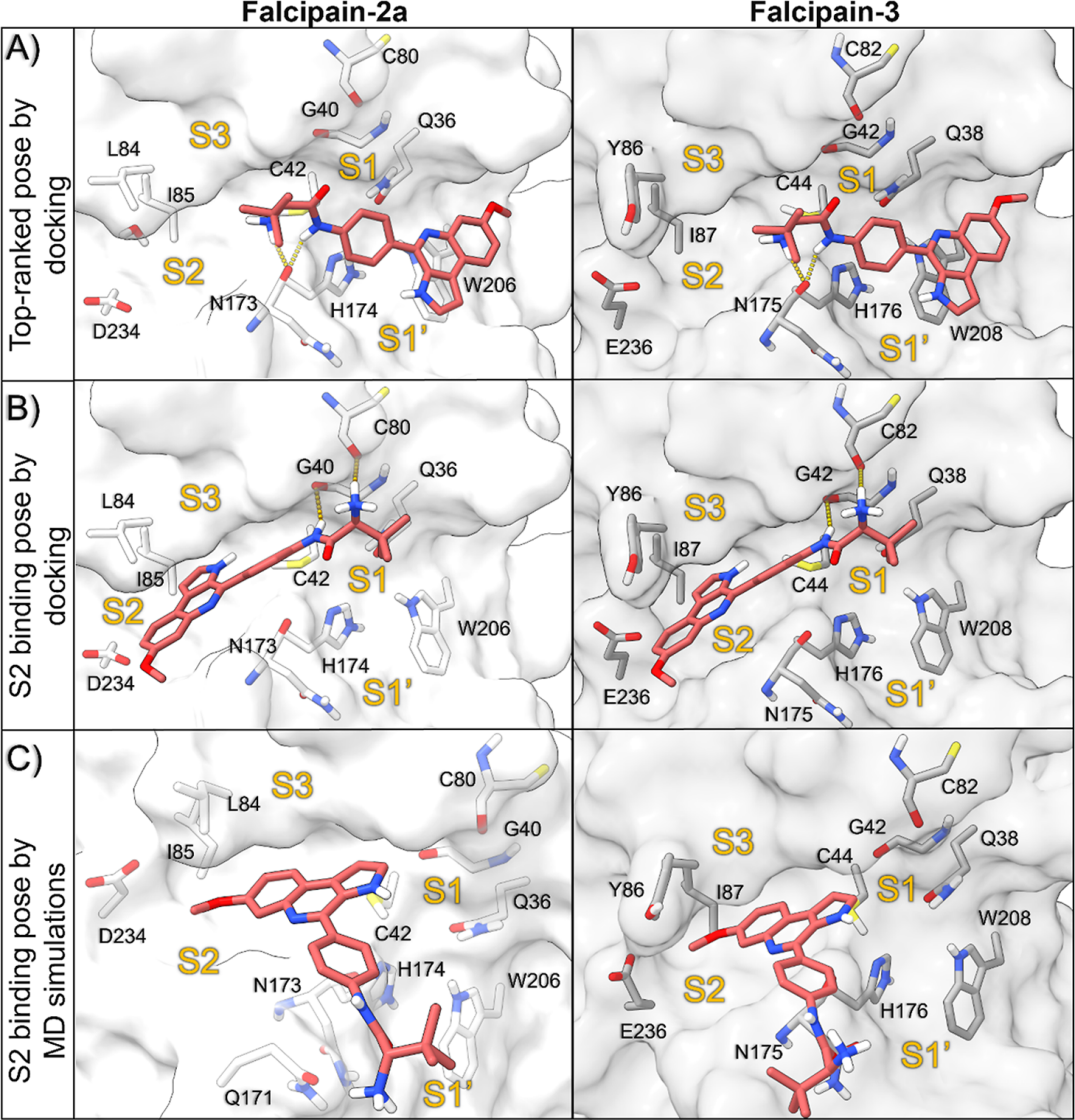
Putative binding modes of 19 against FP-2a (PDB ID: 2OUL) and FP-3 (PDB ID: 3BWK) obtained by molecular docking and dynamic simulation. (A) Top-ranked pose indicated by molecular docking. (B) Representative pose bound to the S2 subpocket indicated by molecular docking. (C) Representative binding modes of **19** in FP-2a and FP-3 subpockets after 300 ns of MD simulations.

To further investigate the role of the methoxy substituent in FP-2a inhibition, we conducted alchemical free energy calculations to transform the OMe group into a hydrogen atom (OMe → H).^46^ Using thermodynamic integration as an estimator for the relative binding free energy (RBFE), the calculations revealed a significant ΔΔG increase of 1.8 ± 0.1 kcal/mol (Table S6). This substantial shift in free energy highlights the critical contribution of the methoxy group in stabilizing the binding of compound **19** to FP-2a. These findings suggest that the most plausible binding mode for compound **19** involves its conformation within the S2 subpocket of FP-2a and FP-3. This hypothesis aligns with comparable results reported by Barbosa da Silva et al.,^47^ who demonstrated that quinazoline-based inhibitors of cruzain from *Trypanosoma cruzi* adopt a similar binding mode in the active site of cysteine proteases.

## 3 Conclusion

The identification of new antimalarial agents targeting intraerythrocytic development is essential to address the clinical challenges associated with malaria symptoms, which often result in periodic disability. In the search for innovative antimalarial drug candidates, the MQ series has emerged as a promising alternative. Among these, compound **19** demonstrated a favorable combination of biological and physicochemical properties. Notably, compound **19** exhibited activity against field isolates of *P. falciparum* and *P. vivax* and showed significant in vivo efficacy, reducing parasitemia in mice by up to 96% on the fifth day postinfection. Biochemical and imaging studies indicated that compound **19** acts via a distinct mechanism of action compared to standard antimalarials, selectively accumulating in infected erythrocytes. Imaging studies revealed a diffuse accumulation of compound **19** in the parasite cytoplasm with pronounced fluorescence intensity within the digestive vacuole (DV) of the parasites. Biochemical investigations further demonstrated that **19** interacts with the parasite’s DV, inhibits proteolytic activity within the parasite, and modulates the enzymatic activity of papain, a cysteine protease used as a surrogate for the parasite homologue. Additionally, molecular modeling studies identified a plausible binding mode for compound **19** with falcipain-2a and falcipain-3, key cysteine proteases involved in hemoglobin digestion. These unique properties position compound **19** as an attractive lead candidate for further optimization. By targeting cysteine proteases, compound **19** offers a novel approach to combat drug resistance and improve treatment outcomes in malaria-affected regions.

## 4 Experimental Section

### 4.1 Chemistry

Commercially available chemicals were purchased from Sigma-Aldrich Chemical Co. or Oakwood Products Inc. and used as received. All the reactions were carried out in a nitrogen atmosphere unless otherwise stated. Solvents used for chromatography were technical grade and were distilled before use. Analytical thin-layer chromatography (TLC) was carried out using Merck Silica gel 60 F254 plates and visualization was accomplished with UV light (365 nm), iodine, vanillin/sulfuric acid and/or ninhydrin staining solutions followed by heating. Flash column chromatography was performed on Merck Silica gel 60 (230–400 mesh) as stationary phase and hexanes/ethyl acetate mixtures as mobile phase, following the flash chromatography procedure described by Still, Khan and Mitra (1978),^48^ or using a Biotage Isolera One purification instrument equipped with Spektra features on Biotage SNAP Ultra 10g or 25 g cartridges as stationary phase and hexanes: ethyl acetate mixtures as mobile phase operating on a gradient mode, or on Biotage SNAP C18 reversed phase 12g cartridges as stationary phase and water/acetonitrile (+0.1% TFA) mixtures as mobile phase operating on a gradient mode. Nuclear magnetic resonance (NMR) analyses were performed on Bruker 250, 400, or 500 MHz spectrometers in solvents as indicated. Chemical shifts (δ) for ^1^H and ^13^C NMR spectra are given in ppm relative to residual solvent chemical shifts converted to the TMS scale (CDCl_3_: δH = 7.26 ppm, δC = 77.16 ppm, DMSO-*d*_6_: δH = 2.50 ppm, δC = 39.52 ppm, D_2_O: δH = 4.79 ppm, MeOD: δH = 3.31 ppm, δC = 49.00 ppm; acetone-*d*_6_: δH = 2.05 ppm). Data are reported as follows: chemical shift (δ), multiplicity, coupling constants (*J*) in Hertz, and integrated intensity. High-Resolution Mass Spectrometry (HRMS) analyses were recorded on an Agilent 6550 Accurate-Mass Q-TOF LC/MS system with Agilent Jet Stream technology for electrospray ionization, on a Thermo Scientific QExactive Hybrid Quadrupole-Orbitrap spectrometers working with electrospray ionization (ESI) in positive mode, or on a BRUKER Impact II mass spectrometer in positive mode. Melting points were determined using a Stuart SMP30 apparatus and are uncorrected. The purity of the tested compounds was analyzed through High-Performance Liquid Chromatography (HPLC) on a Shimadzu prominence HPLC LC-20AT equipment with a photodiode array detector (DAD) detector, on a UPLC system (Waters Acquity H-Class, Waters Corporation) equipped with DAD and fluorescence detectors or on an Agilent Technologies 1260 Infinity with DAD detector. The stationary phase was equipped with Ascentis Express C-18 HPLC column (5 μm particle size, 10 cm × 4.6 mm) or with Acquity UPLC BEH C18 column (1.7 μm particle size, 2.1 × 50 mm) or with Zorbax SB-C18 HPLC column (3.5 μm particle size, 4.6 × 150 mm); and H_2_O/ACN (+0.1% TFA) mixtures as mobile phase (method A: 5–85% of ACN in H_2_O with 0.1% TFA, 40 min; method B: 50–100% of ACN in H_2_O with 0.1% TFA, 23 min; method C: 5–85% of ACN in H_2_O with 0.1% TFA, 15 min; method D: 40% of ACN/H_2_O with 0.1% of formic acid, 10 min; method E: 10–85% of ACN/H_2_O with 0.1% of formic acid, 60 min). HPLC analysis confirmed that all target compounds possessed purities ≥95%.

#### 4.1.1 Synthesis and Characterization of Intermediates **S1–S5** and **2–4**

Described in the Supporting Information.

#### 4.1.2 Synthesis of 4-(7-Methoxy-3*H*-pyrrolo­[2,3-*c*]­quinolin-4-yl)­aniline (**5**)

To a solution of the marinoquinoline **4** (5.4 mmol, 1.72 g) in MeOH (92 mL) was added a solution of NH_4_Cl (0.49 g, 9.2 mmol in 2.7 mL of H_2_O). The resulting reaction mixture was vigorously stirred and zinc dust (83.7 mmol, 5.47 g) was added. The resulting reaction mixture was stirred at room temperature for 2 h. After that time, the reaction mixture was filtered through a short pad of Celite and washed with ethyl acetate (1 × 50 mL) and methanol (1 × 50 mL). The filtered was dried over Na_2_SO_4_, the solvent was evaporated and the compound **5** was employed in the next step without further purification. ^1^H NMR (500 MHz, MeOD) δ: 8.12 (dd, *J* = 8.9, 2.8 Hz, 1H), 7.71 (d, *J* = 8.4 Hz, 2H), 7.51 (d, *J* = 2.6 Hz, 2H), 7.18 (dt, *J* = 8.9, 2.3 Hz, 1H), 7.04 (t, *J* = 2.8 Hz, 1H), 6.92 (d, *J* = 8.4 Hz, 2H), 3.94 (s, 3H). HRMS (ESI+) *m*/*z* calculated for C_18_H_16_N_3_O^+^ [M + H]^+^, 290.12879, found, 290.12838.

#### 4.1.3 General Procedure for the Synthesis of Carbamate-Containing Marinoquinoline Compounds (**6–16**)

To a solution of the respective *N*-Boc-amino acid (0.3 mmol), compound **5** (0.2 mmol, 57.9 mg), and DIPEA (1.15 mmol, 0.2 mL) in anhydrous DMF (1.0 mL) was added HATU (0.24 mmol, 91.3 mg). The resulting reaction mixture was stirred at 50 °C for 4 h. After this period, the reaction was quenched by the addition of water (20 mL) and the aqueous phase was extracted with ethyl acetate (3 × 20 mL). The organic layer was washed with brine (10 mL) and dried over Na_2_SO_4_. The solvent was concentrated under reduced pressure and the residue was purified by RPFC (12g cartridge) eluting with a gradient acetonitrile (5–95%, v/v) in water (+0.1% TFA) to give the corresponding marinoquinolines **6–16**.

##### 4.1.3.1 *tert*-Butyl (*S*)-(1-((4-(7-Methoxy-3*H*-pyrrolo­[2,3-*c*]­quinolin-4-yl)­phenyl)­amino)-1-oxopropan-2-yl)­carbamate (**6**)

Obtained from *N*-Boc-l-Ala-OH (56.8 mg) as a yellow gum in 83% yield (76.5 mg). *R*_f_: 0.53 (Hex/EtOAc 4:1). ${\left[\alpha \right]}_{D}^{20}$ −37 (1.0; MeOH). ^1^H NMR (300 MHz, acetone-*d*_6_) δ: 11.00 (s, 1H), 9.48 (s, 1H), 8.19 (d, *J* = 8.9 Hz, 1H), 8.05 (d, *J* = 8.5 Hz, 2H), 7.87 (d, *J* = 8.4 Hz, 2H), 7.61 (t, *J* = 2.8 Hz, 1H), 7.54 (d, *J* = 2.6 Hz, 1H), 7.20 (dd, *J* = 9.0, 2.6 Hz, 1H), 7.13 (s, 1H), 6.29 (s, 1H), 4.32 (t, *J* = 7.0 Hz, 1H), 3.95 (s, 3H), 1.45 (s, 3H), 1.44 (s, 9H). ^13^C NMR (63 MHz, DMSO-*d*_6_) δ: 172.2, 157.7, 155.3, 145.6, 143.2, 140.0, 129.6, 129.2, 125.9, 124.2, 119.1, 117.1, 116.9, 108.5, 100.7, 79.2, 78.1, 55.2, 50.6, 28.2, 18.0. HRMS (ESI+) *m*/*z* calcd for C_26_H_29_N_4_O_4_^+^ [M + H]^+^, 461.21833, found, 461.21836.

##### 4.1.3.2 *tert*-Butyl (*S*)-(1-((4-(7-Methoxy-3*H*-pyrrolo­[2,3-*c*]­quinolin-4-yl)­phenyl)­amino)-3-Methyl-1-oxobutan-2-yl)­carbamate (**7**)

Obtained from *N*-Boc-l-Val-OH (65.2 mg) as a yellow gum in 91% yield (88.9 mg). *R*_f_: 0.52 (Hex/EtOAc 2:3). ${\left[\alpha \right]}_{D}^{20}$ −14 (1.0; MeOH). ^1^H NMR (250 MHz, DMSO-*d*_6_) δ: 11.76 (br s, 1H), 10.24 (br s, 1H), 8.21 (d, *J* = 8.8 Hz, 1H), 8.01 (d, *J* = 8.6 Hz, 2H), 7.86 (d, *J* = 8.6 Hz, 2H), 7.61 (s, 1H), 7.49 (d, *J* = 2.2 Hz, 1H), 7.21 (dd, *J* = 8.9, 2.2 Hz, 1H), 7.14 (s, 1H), 6.95 (d, *J* = 8.3 Hz, 1H), 3.99 (t, *J* = 8.0 Hz, 1H), 3.91 (s, 3H), 2.13–1.95 (m, 1H), 1.41 (s, 9H), 0.94 (d, *J* = 6.5 Hz, 6H). ^13^C NMR (63 MHz, DMSO-*d*_6_) δ: 171.1, 157.7, 155.7, 145.6, 143.2, 139.8, 132.8, 129.7, 129.3, 128.8, 125.9, 124.2, 119.2, 117.1, 116.9, 108.4, 100.7, 78.2, 60.8, 55.2, 28.2, 19.3, 18.6. HRMS (ESI+) *m*/*z* calcd for C_28_H_33_N_4_O_4_^+^ [M + H]^+^, 489.24963, found, 489.24954.

##### 4.1.3.3 *tert*-Butyl (*S*)-(1-((4-(7-Methoxy-3*H*-pyrrolo­[2,3-*c*]­quinolin-4-yl)­phenyl)­amino)-3,3-dimethyl-1-oxobutan-2-yl)­carbamate (**8**)

Obtained from *N*-Boc-L-Tle–OH (69.4 mg) as a yellow gum in 68% yield (68.4 mg). *R*_f_: 0.57 (Hex/EtOAc 1:1). ${\left[\alpha \right]}_{D}^{20}$ −14 (1.0; MeOH). ^1^H NMR (250 MHz, DMSO-*d*_6_) δ: 11.73 (br s, 1H), 10.26 (br s, 1H), 8.19 (d, *J* = 8.9 Hz, 1H), 8.03 (d, *J* = 8.6 Hz, 2H), 7.88 (d, *J* = 8.6 Hz, 2H), 7.58 (t, *J* = 2.7 Hz, 1H), 7.50 (d, *J* = 2.5 Hz, 1H), 7.20 (dd, *J* = 8.9, 2.6 Hz, 1H), 7.12 (d, *J* = 1.1 Hz, 1H), 6.70 (d, *J* = 9.0 Hz, 1H), 4.15 (d, *J* = 9.0 Hz, 1H), 3.91 (s, 3H), 1.41 (s, 9H), 1.01 (s, 9H). ^13^C NMR (63 MHz, DMSO-*d*_6_) δ: 170.0, 157.6, 155.6, 145.8, 143.6, 139.5, 133.3, 129.5, 129.2, 128.3, 126.0, 124.1, 119.3, 117.2, 116.8, 108.7, 100.6, 78.3, 62.7, 55.2, 34.3, 28.2, 26.6. HRMS (ESI+) *m*/*z* calcd for C_29_H_35_N_4_O_4_^+^ [M + H]^+^, 503.26528, found, 503.26528.

##### 4.1.3.4 *tert*-Butyl (*S*)-(1-((4-(7-Methoxy-3*H*-pyrrolo­[2,3-*c*]­quinolin-4-yl)­phenyl)­amino)-4-Methyl-1-oxopentan-2-yl)­carbamate (**9**)

Obtained from *N*-Boc-l-Leu-OH (69.4 mg) as a yellow gum in 91% yield (91.5 mg). *R*_f_: 0.47 (Hex/EtOAc 1:1). ${\left[\alpha \right]}_{D}^{20}$ −22 (1.0; MeOH). ^1^H NMR (250 MHz, DMSO-*d*_6_) δ: 11.72 (br s, 1H), 10.21 (br s, 1H), 8.19 (d, *J* = 8.9 Hz, 1H), 8.02 (d, *J* = 8.6 Hz, 2H), 7.86 (d, *J* = 8.7 Hz, 2H), 7.59 (t, *J* = 2.8 Hz, 1H), 7.50 (d, *J* = 2.5 Hz, 1H), 7.20 (dd, *J* = 8.8, 2.6 Hz, 1H), 7.12 (dd, *J* = 2.7, 1.5 Hz, 1H), 7.09 (d, *J* = 7.9 Hz, 1H), 4.24–4.15 (m, 1H), 3.91 (s, 3H), 1.76–1.47 (m, 3H), 1.40 (s, 9H), 0.93 (dd, *J* = 6.3, 1.0 Hz, 6H). ^13^C NMR (63 MHz, DMSO-*d*_6_) δ: 172.2, 157.6, 155.6, 145.7, 143.5, 139.9, 133.0, 129.5, 129.2, 128.4, 126.0, 124.1, 119.2, 117.2, 116.8, 108.7, 100.6, 78.1, 55.2, 53.7, 28.2, 24.4, 23.0, 21.6. HRMS (ESI+) *m*/*z* calcd for C_29_H_35_N_4_O_4_^+^ [M + H]^+^, 503.26528, found, 503.26487.

##### 4.1.3.5 *tert*-Butyl (*S*)-(2-((4-(7-Methoxy-3*H*-pyrrolo­[2,3-*c*]­quinolin-4-yl)­phenyl)­amino)-2-oxo-1-phenylethyl)­carbamate (**10**)

Obtained from *N*-Boc-L-Phg-OH (75.4 mg) as a yellow gum in 88% yield (92.0 mg). *R*_f_: 0.52 (Hex/EtOAc 3:2). ${\left[\alpha \right]}_{D}^{20}$ +6 (1.0; MeOH). ^1^H NMR (500 MHz, DMSO-*d*_6_) δ: 11.69 (br s, 1H), 10.49 (br s, 1H), 8.19 (d, *J* = 8.8 Hz, 1H), 8.00 (d, *J* = 8.6 Hz, 2H), 7.83 (d, *J* = 8.6 Hz, 2H), 7.58 (t, *J* = 2.7 Hz, 1H), 7.55 (d, *J* = 7.5 Hz, 2H), 7.49 (d, *J* = 2.4 Hz, 1H), 7.39 (t, *J* = 7.5 Hz, 2H), 7.32 (t, *J* = 7.3 Hz, 1H), 7.20 (dd, *J* = 8.8, 2.6 Hz, 1H), 7.12 (dd, *J* = 2.5, 1.7 Hz, 1H), 5.43 (d, *J* = 8.1 Hz, 1H), 3.90 (s, 3H), 1.41 (s, 9H). ^13^C NMR (126 MHz, DMSO-*d*_6_) δ: 169.4, 157.6, 155.2, 145.6, 143.5, 139.6, 138.0, 133.3, 129.5, 129.2, 128.5, 128.4, 127.9, 127.5, 126.0, 124.1, 119.1, 117.2, 116.9, 108.7, 100.6, 78.5, 55.2, 38.3, 28.2. HRMS (ESI+) *m*/*z* calcd for C_31_H_31_N_4_O_4_^+^ [M + H]^+^, 523.23398, found, 523.23373.

##### 4.1.3.6 *tert*-Butyl (*S*)-(1-((4-(7-Methoxy-3*H*-pyrrolo­[2,3-*c*]­quinolin-4-yl)­phenyl)­amino)-1-oxo-3-phenylpropan-2-yl)­carbamate (11)

Obtained from **11** *N*-Boc-l-Phe-OH (79.6 mg) as a yellow gum in 60% yield (64.4 mg). *R*_f_: 0.55 (Hex/EtOAc 3:2). ${\left[\alpha \right]}_{D}^{20}$ +48 (1.0; MeOH). ^1^H NMR (500 MHz, DMSO-*d*_6_) (mixture of rotamers) δ: 11.73 (br s, 1H), 10.25 (10.22) (br s, 1H), 8.20 (d, *J* = 8.8 Hz, 1H), 8.02 (d, *J* = 8.6 Hz, 2H), 7.84 (d, *J* = 8.5 Hz, 2H), 7.60 (t, *J* = 2.6 Hz, 1H), 7.49 (d, *J* = 2.3 Hz, 1H), 7.36 (d, *J* = 7.4 Hz, 2H), 7.30 (t, *J* = 7.5 Hz, 2H), 7.23–7.19 (m, 2H), 7.17 (d, *J* = 8.2 Hz, 1H), 7.15–7.11 (m, 1H), 4.42–4.38 (4.28–4.24) (m, 1H), 3.91 (s, 3H), 3.05 (dd, *J* = 13.7, 4.6 Hz, 1H), 2.90 (dd, *J* = 13.4, 10.2 Hz, 1H), 1.34 (s, 9H). ^13^C NMR (126 MHz, DMSO-*d*_6_) δ: 171.1, 157.6, 155.5, 145.7, 143.5, 139.8, 137.9, 133.1, 129.5, 129.3, 129.2, 128.4, 128.1, 126.4, 125.9, 124.1, 119.2, 117.1, 116.8, 108.7, 100.6, 78.2, 56.7, 55.2, 37.5, 28.2. HRMS (ESI+) *m*/*z* calcd for C_32_H_33_N_4_O_4_^+^ [M + H]^+^, 537.24963, found, 537.24963.

##### 4.1.3.7 *tert*-Butyl (*S*)-2-((4-(7-Methoxy-3*H*-pyrrolo­[2,3-*c*]­quinolin-4-yl)­phenyl)­carbamoyl)­pyrrolidine-1-carboxylate (**12**)

Obtained from *N*-Boc-l-Pro-OH (64.6 mg) as a yellow gum in 86% yield (83.7 mg). *R*_f_: 0.48 (Hex/EtOAc 3:17). ${\left[\alpha \right]}_{D}^{20}$ −22 (1.0; MeOH). ^1^H NMR (250 MHz, DMSO-*d*_6_) (mixture of rotamers 35:65) δ: 11.80 (br s, 1H), 10.52 (br s, 0.61H), 10.49 (br s, 0.32H), 8.19 (d, *J* = 8.9 Hz, 1H), 8.02 (d, *J* = 8.5 Hz, 2H), 7.89 (d, *J* = 8.6 Hz, 2H), 7.59 (t, *J* = 2.6 Hz, 1H), 7.48 (d, *J* = 2.4 Hz, 1H), 7.19 (dd, *J* = 8.9, 2.5 Hz, 1H), 7.12 (s, 1H), 4.40–4.33 (m, 1H), 3.90 (s, 3H), 3.49–3.32 (m, 2H + H_2_0), 2.36–2.13 (m, 1H), 2.00–1.75 (m, 3H), 1.41 (s, 3.13H), 1.31 (s, 5.86H). ^13^C NMR (63 MHz, DMSO-*d*_6_) (mixture of rotamers 35:65) δ: 171.9 (171.4), 157.8, 153.2 (153.7), 145.4, 142.9, 140.2, 132.4 (132.3), 129.8, 129.3 (129.1), 125.8 (125.9), 124.2, 119.2 (119.1), 117.0 (116.9), 108.2, 100.8, 78.6 (78.7), 60.5 (60.1), 55.2, 46.6 (46.8), 31.1, 30.3, 28.0 (28.2), 24.0, 23.5. HRMS (ESI+) *m*/*z* calcd for C_28_H_31_N_4_O_4_^+^ [M + H]^+^, 487.23398, found, 487.23398.

##### 4.1.3.8 *tert*-Butyl (1-((4-(7-Methoxy-3*H*-pyrrolo­[2,3-*c*]­quinolin-4-yl)­phenyl)­carbamoyl)­cyclopropyl)­carbamate (**13**)

Obtained from 1-((*tert*-butoxycarbonyl)­amino)­cyclopropane-1-carboxylic acid (60.4 mg) as a yellow gum in 60% yield (94.5 mg). *R*_f_: 0.42 (Hex/EtOAc 3:7). ^1^H NMR (300 MHz, Acetone-*d*_6_) δ: 11.19 (br s, 1H), 9.40 (br s, 1H), 8.20 (d, *J* = 8.9 Hz, 1H), 8.00 (d, *J* = 8.4 Hz, 2H), 7.84 (d, *J* = 8.4 Hz, 2H), 7.68 (d, *J* = 3.0 Hz, 1H), 7.55 (d, *J* = 2.6 Hz, 1H), 7.22 (dd, *J* = 8.9, 2.6 Hz, 1H), 7.15 (d, *J* = 2.9 Hz, 1H), 6.95 (br s, 1H), 3.92 (s, 3H), 1.52 (q, *J* = 4.4 Hz, 2H), 1.45 (s, 9H), 1.11 (q, *J* = 4.4 Hz, 2H). ^13^C RMN (125 MHz, DMSO-*d*_6_) δ: 171.1, 157.6, 155.9, 145.8, 143.6, 139.8, 133.2, 129.5, 128.9, 128.3, 126.0, 124.1, 120.0, 117.1, 116.8, 108.7, 100.6, 78.7, 69.8, 55.20, 28.2, 16.9. HRMS (ESI+) *m*/*z* calcd for C_27_H_29_N_4_O_4_^+^ [M + H]^+^, 473.21833, found, 473.21829.

##### 4.1.3.9 *tert*-Butyl (1-((4-(7-Methoxy-3*H*-pyrrolo­[2,3-*c*]­quinolin-4-yl)­phenyl)­amino)-2-methyl-1-oxopropan-2-yl)­carbamate (**14**)

Obtained from 2-((*tert*-butoxycarbonyl)­amino)-2-methylpropanoic acid (61.0 mg) as a yellow gum in 86% yield (81.6 mg). *R*_f_: 0.45 (Hex/EtOAc 7:3). ^1^H RMN (500 MHz, DMSO-*d*_6_) δ: 11.73 (br s, 1H), 9.69 (br s, 1H), 8.19 (d, *J* = 8.8 Hz, 1H), 7.99 (d, *J* = 8.3 Hz, 2H), 7.89 (s, 2H), 7.58 (d, *J* = 2.6 Hz, 1H), 7.49 (d, *J* = 2.5 Hz, 1H), 7.20 (dd, *J* = 8.8, 2.6 Hz, 1H), 7.12 (d, *J* = 2.8 Hz, 1H), 7.03 (br s, 1H), 3.91 (s, 3H), 1.43 (s, 7H), 1.38 (d, *J* = 8.9 Hz, 8H). ^13^C RMN (125 MHz, DMSO-*d*_6_) δ: 173.6, 157.6, 154.4, 145.8, 143.5, 140.4, 132.8, 129.4, 128.8, 128.3, 126.0, 124.1, 119.7, 117.1, 116.7, 108.7, 100.6, 78.3, 56.6, 55.2, 28.2, 25.0. HRMS (ESI+) *m*/*z* calcd for C_27_H_31_N_4_O_4_^+^ [M + H]^+^, 475.23398, found, 475.23404.

##### 4.1.3.10 *tert*-Butyl (4-((4-(7-Methoxy-3*H*-pyrrolo­[2,3-*c*]­quinolin-4-yl)­phenyl)­amino)-2-methyl-4-oxobutan-2-yl)­carbamate (**15**)

Obtained from 3-((*tert*-butoxycarbonyl)­amino)-3-methylbutanoic acid (65.2 mg) as a yellow gum in 62% yield (60.6 mg). *R*_f_: 0.52 (Hex/EtOAc 7:3). ^1^H RMN (500 MHz, DMSO-*d*_6_) δ: 11.71 (br s, 1H), 10.11 (br s, 1H), 8.19 (d, *J* = 8.8 Hz, 1H), 7.99 (d, *J* = 8.5 Hz, 2H), 7.85 (d, *J* = 8.6 Hz, 2H), 7.58 (s, 1H), 7.48 (d, *J* = 2.5 Hz, 1H), 7.20 (dd, *J* = 8.8, 2.5 Hz, 1H), 7.12 (d, *J* = 2.7 Hz, 1H), 3.91 (s, 3H), 2.70 (s, 2H), 1.39 (s, 9H), 1.37 (s, 6H). ^13^C RMN (125 MHz, DMSO-*d*_6_) δ: 169.5, 157.6, 154.3, 145.8, 143.5, 139.9, 133.0, 129.4, 129.1, 128.3, 126.0, 124.1, 119.2, 117.1, 116.8, 108.7, 100.6, 77.5, 55.2, 51.3, 46.6, 28.3, 27.0. HRMS (ESI+) *m*/*z* calcd for C_28_H_33_N_4_O_4_^+^ [M + H]^+^, 489.24963, found, 489.24969.

##### 4.1.3.11 *tert*-Butyl (1-((4-(7-Methoxy-3*H*-pyrrolo­[2,3-*c*]­quinolin-4-yl)­phenyl)­amino)-4,4-dimethyl-1-oxopentan-2-yl)­carbamate (**16**)

Obtained from *N*-Boc-NptGly-OH (73.3 mg) as a yellow gum in 58% yield (59.9 mg). *R*_f_: 0.48 (Hex/EtOAc 1:1). ^1^H NMR (250 MHz, DMSO-*d*_6_) δ: 11.71 (s, 1H), 10.16 (s, 1H), 8.19 (d, *J* = 8.9 Hz, 1H), 8.01 (d, *J* = 8.7 Hz, 2H), 7.85 (d, *J* = 8.7 Hz, 2H), 7.58 (t, *J* = 2.8 Hz, 1H), 7.48 (d, *J* = 2.6 Hz, 1H), 7.20 (dd, *J* = 8.8, 2.6 Hz, 1H), 7.12 (dd, *J* = 2.9, 1.6 Hz, 1H), 7.05 (d, *J* = 8.2 Hz, 1H), 4.24 (q, *J* = 7.3 Hz, 1H), 3.90 (s, 3H), 1.38 (d, *J* = 6.5 Hz, 11H), 0.96 (s, 9H). ^13^C NMR (63 MHz, DMSO-*d*_6_) δ: 172.31, 157.66, 155.15, 145.74, 143.56, 139.92, 133.15, 129.49, 129.16, 128.38, 125.98, 124.97, 119.35, 117.18, 116.85, 108.74, 100.65, 78.23, 55.23, 53.02, 30.47, 30.32, 29.67, 28.29. HRMS (ESI+) *m*/*z* calcd for C_30_H_37_N_4_O_4_^+^ [M + H]^+^, 517.28093, found, 517.28128.

#### 4.1.4 General Procedure for the Synthesis of Amine-Containing Marinoquinoline Compounds (**17–29**)

Freshly distilled acetyl chloride (0.5 mmol, 36 μL) was added dropwise to anhydrous methanol (0.3 mL) at 0 °C and the resulting solution was stirred for 5 min at 0 °C. The solution of the corresponding carbamate (0.1 mmol) in anhydrous methanol (0.5 mL) was then added dropwise. The mixture was warmed gradually to room temperature for 24 h. After this time, the solvent was removed under reduced pressure. The crude residue was then solubilized in methanol and the final compound precipitated by the addition of diethyl ether. The precipitated was triturated or washed with diethyl ether (3 × 4 mL) and subsequently dried under high vacuum to afford the corresponding hydrochlorides **16–25**, which were obtained with high purity without further purification.

##### 4.1.4.1 (*S*)-2-Amino-*N*-(4-(7-methoxy-3*H*-pyrrolo­[2,3-*c*]­quinolin-4-yl)­phenyl)­propanamide Hydrochloride (**17**)

Obtained from marinoquinoline **6** (46.1 mg) as a yellow solid in 46% yield (18.3 mg). mp (°C) 258–259. ${\left[\alpha \right]}_{D}^{20}$ +27 (0.1; MeOH). ^1^H NMR (500 MHz, MeOD) δ: 8.44 (d, *J* = 9.1 Hz, 1H), 8.18 (d, *J* = 2.8 Hz, 1H), 8.11 (d, *J* = 8.7 Hz, 2H), 8.05 (d, *J* = 8.7 Hz, 2H), 7.63 (d, *J* = 2.3 Hz, 1H), 7.48 (dd, *J* = 9.1, 2.4 Hz, 1H), 7.44 (d, *J* = 2.8 Hz, 1H), 4.24 (q, *J* = 7.1 Hz, 1H), 4.02 (s, 3H), 1.68 (d, *J* = 7.1 Hz, 3H). ^13^C NMR (126 MHz, MeOD) δ: 168.59, 161.2, 142.0, 141.9, 138.6, 135.6, 134.9, 131.1, 130.4, 125.5, 124.9, 120.3, 119.7, 116.1, 102.9, 99.8, 55.1, 49.7, 16.3. HRMS (ESI+) *m*/*z* calcd for C_21_H_21_N_4_O_2_^+^ [M + H]^+^, 361.1659, found, 361.1661.

##### 4.1.4.2 (*S*)-2-Amino-*N*-(4-(7-methoxy-3*H*-pyrrolo­[2,3-*c*]­quinolin-4-yl)­phenyl)-3-methylbutanamide Hydrochloride (**18**)

Obtained from marinoquinoline **7** (48.9 mg) as a yellow solid in 54% yield (23.0 mg). mp (°C) 256–258. ${\left[\alpha \right]}_{D}^{20}$ +50 (0.1; MeOH). ^1^H NMR (500 MHz, MeOD) δ: 8.38 (dd, *J* = 9.0, 3.0 Hz, 1H), 8.17 (d, *J* = 2.7 Hz, 1H), 8.13 (d, *J* = 8.7 Hz, 2H), 8.04 (d, *J* = 8.6 Hz, 2H), 7.65 (d, *J* = 2.0 Hz, 1H), 7.45–7.40 (m, 1H), 7.39 (d, *J* = 2.6 Hz, 1H), 4.07 (d, *J* = 5.7 Hz, 1H), 3.99 (s, 3H), 2.42 (dp, *J* = 13.5, 6.7 Hz, 1H), 1.19 (d, *J* = 6.9 Hz, 3H), 1.15 (d, *J* = 7.0 Hz, 3H). ^13^C NMR (126 MHz, MeOD) δ: 168.8, 162.4, 143.1, 143.1, 140.0, 136.8, 136.2, 131.8, 126.8, 126.2, 126.1, 121.7, 121.0, 117.4, 104.2, 101.2, 60.4, 56.5, 31.8, 19.1, 17.9. HRMS (ESI+) *m*/*z* calcd for C_23_H_25_N_4_O_2_^+^ [M + H]^+^, 389.19720, found, 389.19695.

##### 4.1.4.3 (*S*)-2-Amino-*N*-(4-(7-methoxy-3*H*-pyrrolo­[2,3-*c*]­quinolin-4-yl)­phenyl)-3,3-dimethylbutanamide Hydrochloride (**19**)

Obtained from marinoquinoline **8** (50.3 mg) as a yellow solid in 44% yield (19.3 mg). mp (°C) 272. ${\left[\alpha \right]}_{D}^{20}$ +52 (0.1; MeOH). ^1^H NMR (250 MHz, MeOD) δ: 8.33 (d, *J* = 9.1 Hz, 1H), 8.18–8.10 (m, 3H), 8.03 (d, *J* = 8.6 Hz, 2H), 7.60 (d, *J* = 2.2 Hz, 1H), 7.38 (dd, *J* = 9.8, 2.4 Hz, 2H), 4.01 (s, 1H), 3.97 (s, 3H), 1.22 (s, 9H). ^13^C NMR (63 MHz, MeOD) δ: 168.2, 162.4, 143.0, 142.9, 140.0, 136.8, 136.1, 131.8, 126.8, 126.2, 126.0, 121.8, 121.0, 117.3, 104.2, 101.1, 63.4, 56.5, 34.8, 26.8. HRMS (ESI+) *m*/*z* calcd for C_24_H_27_N_4_O_2_^+^ [M + H]^+^, 403.21285, found, 403.21268.

##### 4.1.4.4 (*S*)-2-Amino-*N*-(4-(7-methoxy-3*H*-pyrrolo­[2,3-*c*]­quinolin-4-yl)­phenyl)-4-methylpentanamide Hydrochloride (**20**)

Obtained from marinoquinoline **9** (50.3 mg) as a yellow solid in 45% yield (19.8 mg). mp (°C) 259–260. ${\left[\alpha \right]}_{D}^{20}$ +28 (0.1; MeOH). ^1^H NMR (500 MHz, MeOD) δ: 8.41 (d, *J* = 9.1 Hz, 1H), 8.18 (d, *J* = 2.8 Hz, 1H), 8.13 (d, *J* = 8.7 Hz, 2H), 8.05 (d, *J* = 8.7 Hz, 2H), 7.64 (d, *J* = 2.3 Hz, 1H), 7.45 (dd, *J* = 9.1, 2.4 Hz, 1H), 7.42 (d, *J* = 2.8 Hz, 1H), 4.22 (dd, *J* = 8.0, 6.2 Hz, 1H), 4.01 (s, 3H), 1.95–1.78 (m, 3H), 1.08 (dd, *J* = 6.4, 3.3 Hz, 6H). ^13^C NMR (126 MHz, MeOD) δ: 169.8, 162.5, 143.2, 143.2, 140.0, 136.9, 136.3, 131.8, 126.9, 126.3, 126.2, 121.8, 121.0, 117.4, 104.2, 101.2, 56.5, 53.9, 41.7, 25.6, 23.3, 22.1. HRMS (ESI+) *m*/*z* calcd for C_24_H_27_N_4_O_2_^+^ [M + H]^+^, 403.21285, found, 403.21289.

##### 4.1.4.5 (*S*)-2-Amino-*N*-(4-(7-methoxy-3*H*-pyrrolo­[2,3-*c*]­quinolin-4-yl)­phenyl)-2-phenylacetamide Hydrochloride (**21**)

Obtained from marinoquinoline **10** (52.3 mg) as a yellow solid in 51% yield (21.6 mg). mp (°C) 294. ${\left[\alpha \right]}_{D}^{20}$ +16 (1.0; MeOH). ^1^H NMR (300 MHz, D_2_O) δ: 8.10 (d, *J* = 9.2 Hz, 1H), 8.03 (d, *J* = 2.8 Hz, 1H), 7.87 (d, *J* = 8.5 Hz, 2H), 7.79 (d, *J* = 8.5 Hz, 2H), 7.69–7.56 (m, 5H), 7.26 (d, *J* = 8.1 Hz, 2H), 7.17 (d, *J* = 2.8 Hz, 1H), 5.40 (s, 1H), 3.89 (s, 3H). ^13^C NMR (126 MHz, MeOD) δ: 167.8, 162.4, 143.3, 143.2, 139.9, 136.8, 136.3, 134.0, 131.7, 131.3, 130.6, 129.5, 126.8, 126.3, 126.1, 121.6, 121.0, 117.4, 104.2, 101.3, 58.5, 56.5. HRMS (ESI+) *m*/*z* calcd for C_26_H_23_N_4_O_2_^+^ [M + H]^+^, 423.18155, found, 423.18178.

##### 4.1.4.6 (*S*)-2-Amino-*N*-(4-(7-methoxy-3*H*-pyrrolo­[2,3-*c*]­quinolin-4-yl)­phenyl)-3-phenylpropanamide Hydrochloride (**22**)

Obtained from marinoquinoline **11** (53,7 mg) as a yellow solid in 48% yield (22,7 mg). mp (°C) 272–273. ${\left[\alpha \right]}_{D}^{20}$ +96 (0.1; MeOH). ^1^H NMR (600 MHz, D_2_O) δ: 7.96 (d, *J* = 2.7 Hz, 1H), 7.86 (d, *J* = 9.0 Hz, 1H), 7.79 (d, *J* = 8.2 Hz, 2H), 7.71 (d, *J* = 8.2 Hz, 2H), 7.42 (dd, *J* = 14.2, 7.0 Hz, 3H), 7.36 (d, *J* = 7.4 Hz, 2H), 7.09 (d, *J* = 8.7 Hz, 1H), 7.05–7.00 (m, 2H), 4.45 (t, *J* = 7.3 Hz, 1H), 3.80 (s, 3H), 3.38 (dd, *J* = 14.1, 6.9 Hz, 1H), 3.32 (dd, *J* = 14.1, 7.9 Hz, 1H). ^13^C NMR (126 MHz, MeOD) δ: 167.2, 161.2, 141.9, 141.6, 138.6, 135.6, 134.9, 134.0, 130.3, 129.2, 128.8, 127.6, 125.6, 125.1, 124.9, 120.3, 119.7, 116.1, 102.9, 99.8, 55.2, 55.1, 37.4. HRMS (ESI+) *m*/*z* calcd for C_27_H_25_N_4_O_2_^+^ [M + H]^+^, 437.1972, found, 437.1962.

##### 4.1.4.7 (*S*)-*N*-(4-(7-Methoxy-3*H*-pyrrolo­[2,3-*c*]­quinolin-4-yl)­phenyl)­pyrrolidine-2-carboxamide Hydrochloride (**23**)

Obtained from marinoquinoline **12** (48.7 mg) as a yellow solid in 47% yield (19.9 mg). mp (°C) 253–254. ${\left[\alpha \right]}_{D}^{20}$ −3 (0.1; MeOH). ^1^H NMR (500 MHz, MeOD) δ: 8.44 (d, *J* = 9.1 Hz, 1H), 8.19 (d, *J* = 2.8 Hz, 1H), 8.10 (d, *J* = 8.7 Hz, 2H), 8.05 (d, *J* = 8.8 Hz, 2H), 7.63 (d, *J* = 2.3 Hz, 1H), 7.48 (dd, *J* = 9.1, 2.4 Hz, 1H), 7.44 (d, *J* = 2.8 Hz, 1H), 4.60–4.55 (m, 1H), 4.02 (s, 3H), 3.55–3.42 (m, 2H), 2.69–2.61 (m, 1H), 2.24–2.13 (m, 3H). ^13^C NMR (126 MHz, MeOD) δ: 168.6, 162.6, 143.2, 140.0, 137.0, 136.3, 131.8, 126.9, 126.4, 126.2, 121.7, 121.1, 117.5, 104.3, 101.2, 62.0, 56.5, 47.6, 31.1, 25.1. HRMS (ESI+) *m*/*z* calcd for C_23_H_23_N_4_O_2_^+^ [M + H]^+^, 387.18155, found, 387.18152.

##### 4.1.4.8 1-Amino-*N*-(4-(7-Methoxy-3*H*-pyrrolo­[2,3-*c*]­quinolin-4-yl)­phenyl)­cyclopropane-1-carboxamide Hydrochloride (**24**)

Obtained from marinoquinoline **13** (47.2 mg) as a yellow solid in 49% yield (20.0 mg). mp (°C) 232. ^1^H NMR (600 MHz, D_2_O) δ: 7.80 (s, 1H), 7.68 (d, *J* = 7.8 Hz, 2H), 7.53 (d, *J* = 8.0 Hz, 3H), 6.85 (d, *J* = 8.8 Hz, 1H), 6.77 (s, 2H), 3.67 (s, 3H), 1.84 (s, 2H), 1.69 (s, 2H). ^13^C NMR (126 MHz, MeOD) δ: 170.0, 162.5, 143.2, 143.2, 140.0, 136.9, 136.3, 131.5, 126.9, 126.3, 126.2, 122.8, 121.0, 117.4, 104.2, 101.2, 56.5, 37.3, 13.8. HRMS (ESI+) *m*/*z* calcd for C_22_H_22_N_4_O_2_^+^ [M + H]^+^, 373.16590, found, 373.16592.

##### 4.1.4.9 2-Amino-*N*-(4-(7-Methoxy-3*H*-pyrrolo­[2,3-*c*]­quinolin-4-yl)­phenyl)-2-Methylpropanamide Hydrochloride (**25**)

Obtained from marinoquinoline **14** (47.5 mg) as a yellow solid in 68% yield (28.0 mg). mp (°C) 266–267. ^1^H NMR (600 MHz, D_2_O) δ: 8.07 (d, *J* = 8.9 Hz, 1H), 8.03 (s, 1H), 7.91 (d, *J* = 8.2 Hz, 2H), 7.87 (d, *J* = 8.3 Hz, 2H), 7.25 (d, *J* = 9.0 Hz, 1H), 7.20 (s, 1H), 7.16 (s, 1H), 3.90 (s, 3H), 1.83 (s, 6H). ^13^C NMR (126 MHz, MeOD) δ: 172.1, 162.6, 143.4, 143.2, 139.9, 136.9, 136.5, 131.6, 127.0, 126.8, 126.3, 122.5, 121.0, 117.6, 104.2, 101.4, 59.1, 56.5, 23.9. HRMS (ESI+) *m*/*z* calcd for C_22_H_23_N_4_O_2_^+^ [M + H]^+^, 375.18155, found, 375.18155.

##### 4.1.4.10 3-Amino-*N*-(4-(7-Methoxy-3*H*-pyrrolo­[2,3-*c*]­quinolin-4-yl)­phenyl)-3-Methylbutanamide Hydrochloride (**26**)

Obtained from marinoquinoline **15** (48.9 mg) as a yellow solid in 44% yield (18.7 mg). mp (°C) 233–234. ^1^H NMR (500 MHz, MeOD) δ: 8.42 (d, *J* = 9.0 Hz, 1H), 8.17 (s, 1H), 8.10 (d, *J* = 8.1 Hz, 2H), 8.02 (d, *J* = 8.1 Hz, 2H), 7.63 (s, 1H), 7.47 (d, *J* = 8.7 Hz, 1H), 7.42 (s, 1H), 4.01 (s, 3H), 2.86 (s, 2H), 1.51 (s, 6H). ^13^C NMR (126 MHz, MeOD) δ: 171.2, 162.5, 143.5, 143.4, 139.9, 136.9, 136.3, 131.6, 126.9, 126.2, 126.0, 121.6, 121.0, 117.5, 104.2, 101.2, 56.5, 54.0, 45.2, 26.1. HRMS (ESI+) *m*/*z* calcd for C_23_H_25_N_4_O_2_^+^ [M + H]^+^, 389.19720, found, 389.19717.

##### 4.1.4.11 2-Amino-*N*-(4-(7-Methoxy-3*H*-pyrrolo­[2,3-*c*]­quinolin-4-yl)­phenyl)-4,4-dimethylpentanamide Hydrochloride (**27**)

Obtained from marinoquinoline **16** (51.7 mg) as a yellow solid in 38% yield (16.1 mg). mp 244–245 (°C). ^1^H NMR (250 MHz, MeOD) δ: 8.46 (d, *J* = 9.1 Hz, 1H), 8.18 (d, *J* = 2.9 Hz, 1H), 8.14 (d, *J* = 8.6 Hz, 2H), 8.06 (d, *J* = 8.7 Hz, 2H), 7.65 (d, *J* = 2.4 Hz, 1H), 7.49 (dd, *J* = 9.0, 2.4 Hz, 1H), 7.44 (d, *J* = 2.9 Hz, 1H), 4.21 (dd, *J* = 8.8, 4.5 Hz, 1H), 4.03 (s, 3H), 1.08 (s, 9H). ^13^C NMR (63 MHz, MeOD) δ: 168.50, 161.17, 141.97, 141.69, 138.46, 135.51, 135.10, 130.38, 125.52, 125.38, 124.92, 120.47, 119.66, 116.17, 102.82, 99.98, 65.50, 55.11, 51.96, 44.85, 29.68, 28.50.

HRMS (ESI+) *m*/*z* calcd for C_25_H_29_N_4_O_2_^+^ [M + H]^+^, 417.22850, found, 417.22730.

Chiral resolution by analytical HPLC of the compound **16**, using a Daicel CHIRALPAK IB column and 20% of isopropanol in hexanes as mobile phase (1.0 mL/min), followed by deprotection step provided (−)-**27** and (+)-**27**.

(−)-**27**: ${\left[\alpha \right]}_{D}^{20}$ −3 (0.23; MeOH).

(+)-**27**: ${\left[\alpha \right]}_{D}^{20}$ +11 (0.54; MeOH).

##### 4.1.4.12 (*R*)-2-Amino-*N*-(4-(7-methoxy-3*H*-pyrrolo­[2,3-*c*]­quinolin-4-yl)­phenyl)-3,3-dimethylbutanamide Hydrochloride (**28**)

Obtained from *tert*-butyl (*R*)-(1-((4-(7-methoxy-3*H*-pyrrolo­[2,3-*c*]­quinolin-4-yl)­phenyl)­amino)-3,3-dimethyl-1-oxobutan-2-yl)­carbamate (0.05 mmol, 25.2 mg) as a yellow solid in 39% yield (10.5 mg). ${\left[\alpha \right]}_{D}^{20}$ +50 (0.1; MeOH). ^1^H NMR (250 MHz, MeOD) δ: 8.33 (d, *J* = 9.1 Hz, 1H), 8.19–8.09 (m, 3H), 8.03 (d, *J* = 8.7 Hz, 2H), 7.60 (d, *J* = 2.4 Hz, 1H), 7.44–7.32 (m, 2H), 4.01 (s, 1H), 3.97 (s, 3H), 1.22 (s, 9H). HRMS (ESI+) *m*/*z* calcd for C_24_H_27_N_4_O_2_^+^ [M + H]^+^, 403.21285, found, 403.21147.

##### 4.1.4.13 (*R*)-*N*-(4-(7-Methoxy-3*H*-pyrrolo­[2,3-*c*]­quinolin-4-yl)­phenyl)­pyrrolidine-2-carboxamide Hydrochloride (**29**)

Obtained from *tert*-butyl (*R*)-2-((4-(7-methoxy-3*H*-pyrrolo­[2,3-*c*]­quinolin-4-yl)­phenyl)­carbamoyl)­pyrrolidine-1-carboxylate (0.05 mmol, 19.32 mg) as a yellow solid in 47% yield (9.9 mg). ${\left[\alpha \right]}_{D}^{20}$ −5 (0.1; MeOH). ^1^H NMR (250 MHz, MeOD) δ: 8.48 (d, *J* = 9.1 Hz, 1H), 8.19 (d, *J* = 2.9 Hz, 1H), 8.12–8.08 (m, 4H), 8.07–8.01 (m, 2H), 7.62 (d, *J* = 2.4 Hz, 1H), 7.52 (dd, *J* = 9.1, 2.5 Hz, 1H), 7.46 (d, *J* = 2.9 Hz, 1H), 4.59–4.49 (m, 1H), 4.03 (s, 3H), 3.55–3.38 (m, 2H), 2.71–2.55 (m, 1H), 2.29–2.08 (m, 3H). HRMS (ESI+) *m*/*z* calcd for C_23_H_23_N_4_O_2_^+^ [M + H]^+^, 387.18155, found, 387.18093.

### 4.2 Biological Assays

#### 4.2.1 P. falciparum Culture

Continuous in vitro cultures of *P. falciparum* (strains 3D7, K1, Dd2, TM90C6B, IPC4912 and 3D7^R^_MMV848) were kept using an adaptation of the method described by Trager and Jansen with modifications.^49^ Cultures were maintained in a low-oxygen atmosphere (5% O_2_, 5% CO_2_, 90% N_2_) in a humidified environment at 37 °C. The parasites were cultivated in a 2% hematocrit suspension of O^+^ human red blood cells, in RPMI 1640 medium supplemented with 25 mM Hepes (pH 7.4), 21 mM sodium bicarbonate, 11 mM d-glucose, 3.67 mM hypoxanthine, 40 μg/mL of penicillin–streptomycin and 0.5% (w/v) Albumax. Parasite percentage was assessed through daily blood smears.

#### 4.2.2 Hemolytic Activity Assay

Fresh human red blood cells (RBCs) were employed to evaluate hemolytic activity. RBCs were incubated with MQ19 (10 μM), saponin (0.005%), or DMSO (at the highest concentration used in the compound dilutions) in 96-well plates at 37 °C, using a 2% hematocrit suspension. Hemolysis was assessed at 24, 48, and 72 h of incubation. As controls, RBCs incubated with RPMI medium alone served as the negative control, while 0.1% saponin was used as the positive control. Following each incubation period, the plates were centrifuged, and the supernatants were transferred to new plates for hemoglobin quantification by measuring absorbance at 540 nm. The percentage of hemolysis was calculated using the following equation

$$Hemolysis\;\left(\%\right)=100\times \frac{A_{sample}-A_{positive\;control}}{A_{positive\;control}-A_{negative\;control}}$$

where *A*_sample_ is the absorbance of the test sample, *A*_negative_ control is the absorbance of the untreated RBCs in RPMI medium, and *A*_positive_ control A is the absorbance of RBCs treated with 0.1% saponin. The saponin control was set as 100% hemolysis.

#### 4.2.3 SYBR Green I Growth Inhibition Assay Against *P. falciparum* Asexual Forms

*P. falciparum* culture was synchronized for assays by treatment with a sorbitol solution of 5%, as described by Lambros and Vanderberg (1979).^50^ Compounds were diluted to a stock concentration of 20 mM in 100% DMSO before the experiments and maintained at −20 °C. Compound inhibitory potencies were determined using the SYBR Green I phenotypic assay.^51^ Briefly, in 96-well plates, 180 μL of a parasite suspension in the early trophozoite (ring) form at 0.5% parasitemia and 2% hematocrit was incubated for 72 h with 20 μL of 10× concentrated serial dilutions of each compound. The antimalarial artesunate (ART) was tested against all strains as a positive control of inhibition for the experiments. The plates were kept at 37 °C in an atmosphere of 5% O_2_, 5% CO_2_, 90% N_2_. Positive and negative growth controls were added to each independent plate. The growth medium was then removed, and the deposited cells were resuspended in PBS buffer (116 mM NaCl, 10 mM Na_2_HPO_4_, 3 mM KH_2_PO_4_). A solution of SYBR GREEN I DNA Stain diluted in lysis buffer (20 mM Tris-base, 5 mM EDTA, 0.008% (w/v) saponin and 0,0008% (v/v) Triton X-100, at pH 7.5) was added to induce hemolysis. The plates were incubated for an additional 30 min, after which the fluorescence of the plate was measured (absorption and emission wavelengths of 485 and 535 nm, respectively). Fluorescence intensity was analyzed in terms of parasite viability as compared to controls, using the Origin 9.0 software (OriginLab Corp., MA, USA). Dose–response curves were built, and half-maximal inhibitory concentration (IC_50_) values were determined for each compound using nonlinear regression analysis.

#### 4.2.4 Cross-Resistance Assays

The antiplasmodial activity of the test compounds was assessed against a representative panel of *P. falciparum* resistant strains. This panel comprised the following: 3D7 (chloroquine-sensitive), Dd2 (resistant to chloroquine, mefloquine, and pyrimethamine), K1 (resistant to chloroquine, mefloquine, pyrimethamine, and sulfadoxine), and 3D7^R^_MMV848 (resistant to MMV692848, a PfPI4K inhibitor). The IC_50_ values of the compounds against each resistant strain were determined, as described previously. Following this, a resistance index (RI) was calculated by dividing the IC_50_ value of the resistant strain by that of 3D7. It is worth noting that RI values exceeding 5 were considered indicative of cross-resistance.

#### 4.2.5 MTT Assay for Cytotoxicity Evaluation

A culture of HepG2 cells was obtained from the BCRJ cell bank and kept in a flask in a humidified atmosphere of 5% CO_2_ at 37 °C. The culture medium used was RPMI 1640 supplemented with 25 mM HEPES (pH 7.4), 24 mM sodium bicarbonate, 11 mM d-glucose, 40 μg/mL penicillin–streptomycin and 10% (v/v) bovine fetal serum. Every three to 4 days, treatment with a 0.25% trypsin solution was used to release cells from the flask walls and a 1:4 proportion of the cells were maintained in culture. An adaptation of the MTT assay described by Denizot and Lang (1986) was employed to determine cytotoxic activity.^52^ HepG2 cells were distributed in a 96-well plate in a proportion of 1 × 10^4^ cells per well. The plate was incubated overnight at 37 °C, in a humidified atmosphere of 5% CO_2_. After that, serial dilutions of the compounds were to each well, and the plates were incubated for another 72 h. Positive (no cell) and negative (no compound) controls were added to each plate for normalization of results. The supernatant was then removed, and a solution of MTT salt (3-(4,5-dimethylthiazol-2-yl)-2,5-diphenyltetrazolium bromide) was added to each well. After 2 to 4 h of incubation, the formazan crystals formed by MTT reduction were solubilized in DMSO. The absorbance of the plate at the 570 nm wavelength was measured, and the intensity values obtained were converted to viability values. Concentration–response curves were constructed for each compound using the OriginPro 9.0 software (OriginLab, MA, USA) and the cytotoxic concentration for 50% of cells (CC_50_^HepG2^) was determined for each. The selectivity index (SI) was calculated by the ratio of CC_50_^HepG2^ to IC_50_^3D7^. Compounds with an SI over 10 are generally considered noncytotoxic, or rather, well-tolerated.

#### 4.2.6 Combination Assays

The combination assay was adapted from the work of Fivelman and collaborators (2004).^53^ The compounds were diluted and combined in a 96-well plate using eight fixed-ratio combinations (1:0, 6:1, 5:2, 4:3, 3:4, 2:5, 1:6, 0:1). The starting concentrations for all compounds were set at 10 × IC50, and the experiment was conducted with 0.5% parasitemia and 2% hematocrit. Serial dilutions of these combinations were prepared and incubated with the parasite to assess their antiplasmodial activity against *P. falciparum*. The SYBR Green I assay was used to determine the IC_50_ value for each combination, with data analysis performed using GraphPad Prism version 8.0.1 (GraphPad Software, San Diego, CA, USA). The additivity isobole was calculated based on the Hand model.^25,26^ Fractional inhibitory concentration (FIC_50_) values are expressed as IC_50_ equivalents and were determined for the seven different proportions of the compounds and artesunate. FIC_50_ values from three independent experiments were modeled using nonlinear fitting and compared statistically to the additivity isobole. A lack of statistical difference between the model and the additivity isobole indicated an additive drug combination, while significant deviations revealed a synergistic (model below the additivity curve) or antagonistic (model above the additivity curve) interaction.

#### 4.2.7 Ex Vivo Isolates from Porto Velho, Brazil

This study was approved by the Centro de Pesquisa em Medicina Tropical-CEPEM-Rondônia ethics committee (CAAE 58738416.1.0000.0011). All participants signed a written informed consent before blood collection by a trained nurse. *P. falciparum* and *P. vivax* clinical isolates were collected on September and October 2022 from patients recruited at the Centre of Malaria Control (CEPEM) in the city of Porto Velho, state of Rondônia, in the Brazilian Western Amazon. Solely monoinfected patients with *P. falciparum* with parasitaemia between 2000 and 80,000 parasites/μL and with at least 70% of ring stage parasites were recruited. Patients who had used an antimalarial in the previous month and/or presented with symptoms of severe malaria were excluded from the study. The study cohort comprised 18 patients living in this highly endemic area. A peripheral venous blood sample (5 mL) was collected from each individual by venipuncture in heparin-containing tubes and immediately used in the ex vivo drug susceptibility assay using preprepared plates containing diluted antimalarial compounds. Compound **19** and the control drugs (artesunate and chloroquine) were prepared from stock solutions in DMSO at 2 mM. Next, the compounds were diluted in assay medium, 20000-fold, and 40-fold and 20-fold to prepare the initial drug solution of 0.1 μM (artesunate), 50 μM (Chloroquine), 100 μM (compound **19**) then a 2-fold serial dilution was made in assay medium from this stock. Finally, 20 μL of each dilution was transferred into the ex vivo assay plate and diluted 10-fold into the final assay medium containing parasites. Next, 5 mL of whole blood were centrifuged at 800 g for 10 min and the plasma and buffy coat removed. The red blood cell pellet was then washed with culture medium (RPMI 1640 medium for *P. falciparum* isolates and IMDM medium for *P. vivax*) and diluted 1× (50% hematocrit) before filtration through a CF11 cellulose column.^54^ After the CF11 cellulose column step, the blood was centrifuged and the packed red blood cells with the parasites (iRBC) were diluted to a 2% hematocrit, using RPMI 1640 medium plus 20% human serum. Control assays on a 3D7 lab isolate was performed with media supplemented with human serum (20%). The iRBC (180 μL per well) were distributed in the predosed drug plate. For the maturation of parasites, rings to schizonts, the plates were maintained in a hypoxia incubator chamber (containing 5% O_2_, 5% CO_2_ and 90% N_2_) at 37 °C as described, for 40–47 h. Control wells containing iRBCs drug-free were cultured in culture medium. The parasite-drug incubation was terminated when 40% of the ring stages reached the schizont stage (at least three distinct nuclei per parasite) in the drug-free control wells. Thick blood films were then made from each well, dried, stained with 5% Giemsa solution for 30 min, and examined microscopically. The number of schizonts per 200 asexual stage parasites was determined for each drug concentration and then normalized by comparing with the schizont number in the drug-free control wells (considered as 100%).^55^ The half-maximal drug inhibitory response (EC_50_) was estimated by curve fitting using software from the OriginLab Corporation, Northampton, MA, USA and comparing with parasite growth in the drug-free controls.

#### 4.2.8 Speed of Action Assay

To categorize the compounds based on their fast or slow-acting profiles, two protocols were simultaneously conducted, adapted from Le Manach et al. (2013).^56^ The speed of action of the compounds was assessed by preparing three identical plates (A, B, and C) with equivalent compound dilutions. Each plate was incubated with *P. falciparum* 3D7 strain, wherein >90% of the stages were in the ring form (synchronized), adjusted to 2% hematocrit and 0.5% parasitaemia. The three plates were incubated with the inhibitor under growth conditions for durations of 24 h (plate A), 48 h (plate B), or 72 h (plate C). Following the respective incubation periods, plates A and B underwent three washes with RPMI medium to eliminate the inhibitors and were subsequently incubated for an additional 48 and 24 h, respectively. Plate C remained continuously incubated in the presence of the inhibitor throughout the entire period. Postincubation, viabilities, and IC_50_ values for each plate were assessed using the SYBr Green I assay. The IC_50_ values from the three incubation times were compared to ascertain any significant differences in inhibitory potency (IC_50_) resulting from each duration.

#### 4.2.9 *P. falciparum* Stage-Specificity Inhibition Assay

To evaluate the activity of **19** on various stages of *P. falciparum* intraerythrocytic development, a protocol adapted from Murithi et al. (2020) was employed.^57^ A *P. falciparum* sample was synchronized at ring-stage using **t**he MACS magnetic LS column and used to prepare a mixture with 0.5% parasitemia and 2% hematocrit. This mixture was distributed across six 96-well plates. Five plates assessed the inhibitory activity of the compound during specific 8 h intervals representing different intraerythrocytic development stages: Plate A (0–8 h, early ring), Plate B (8–16 h, late ring), Plate C (16–24 h, early trophozoite), Plate D (24–32 h, late trophozoite), and Plate E (32–40 h, schizont).^58^ Compound **19** was added to each plate according to its respective time interval, followed by washin**g** of iRBCs with RPMI-1640 medium after each exposure. The protocol was applied to all five plates (A, B, C, D, E) for each time point. All plates were kept at 37 °C in a humidified incubator with a gas mixture of 90% N_2_, 5% O_2_, and 5% CO_2_ for 60 h. Parasite viability was assessed at the end of 60 h using the SYBR Green I assay, indicating the inhibitory activity at each developmental stage. An additional plate was included to determine the antiplasmodial potency of **19** using the standard 72 h assay. Fluorescence intensity measurements were analyzed in relation to parasite viability compared to controls through GraphPad Prism version 8.0.1 (GraphPad Software, San Diego, CA, USA). Concentration–response curves were generated, and half-maximal inhibitory concentration (IC_50_) values were determined for each exposure period using nonlinear regression analysis.

#### 4.2.10 Confocal Microscopy

Erythrocytes infected with *P. falciparum* (3D7 strain) nonsynchronous parasites were washed in MOPS buffer (116 mM NaCl, 5.4 mM KCl, 0.8 mM MgSO_4_, 5.5 mM d-glucose, 50 mM MOPS, and 2 mM CaCl_2_, pH 7.4), resuspended in the same buffer and plated on a microscopy chamber previously pretreated for 1h with l-polylysine (1 mg/mL). The cultured HepG2 cells were detached by trypsinization using 0.25% trypsin–EDTA solution (Sigma-Aldrich) for 10 min and afterward, were centrifuged at 600*g* for 10 min. The supernatant was discarded, and the cell pellets were then resuspended in RPMI medium. HepG2 cells (1 × 10^6^ cells/ml; 200 μL/well) were transferred into 24-well microplates and incubated at 37 °C in an atmosphere humidified with 5% of CO_2_ for 24h. For fluorescence confocal microscopy, **19** (10 μM) was added to erythrocytes infected and HepG2 and image acquisition was performed in a Leica TCS SP8 confocal microscope (Leica Microsystems, Wetzlar, Germany) using an oil immersion 63× objective and 405 nm laser.

#### 4.2.11 Generation of Resistance Against **19** in Dd2 Strains

The high-pressure intermittent selection method described previously was the protocol followed to create a resistant line using *P. falciparum* strain Dd2.^28^ Parasites from Dd2 (MRA-1255), recently cloned, containing 1 × 10^9^ healthy rings were inoculated with 2.5% hematocrit in 40 mL of culture media, with a high compound pressure, 3× the defined IC_90_ against blood stages for 4 days, with media changes daily, under standard culture conditions. By then, parasitemia was undetectable by light microscopy, and the compound was removed. The culture medium was changed three times a week, and 1/3 of the cultured blood was changed weekly, for 60 days.

#### 4.2.12 *P. falciparum*’s Digestive Vacuole Homeostasis

Synchronous trophozoites of *P. falciparum* (3D7 strain) were marked with the lysosomotropic probe acridine orange (AO) (Sigma-Aldrich), as described by Bennett et al. (2004) with modifications.^59^ Briefly, the culture with 10% parasitaemia was centrifuged for 5 min at 9000*g* and resuspended in RPMI without phenol red. The RBC number was determined using a Neubauer chamber and it was adjusted to 1 × 10^7^ cells.mL^–1^ in MOPS buffer, pH 7.2 supplemented with 5 μM AO. The sample was incubated for 40 min at 37 °C. After that, cells were washed three times and resuspended in 700 μL of the same buffer. The fluorescence intensity (λ_ex_ = 488 nm; λ_em_ = 560 nm) was measured continuously, at 37 °C, before and after the addition of **19** and CQ at 40 μM. in a Hitachi F-7000 fluorimeter (Tokyo, Japan).^60^ Experiments were performed in triplicate.

#### 4.2.13 Spectrofluorimetric Assay to Assess the Proteolytic Activity

Isolated parasites were incubated at room temperature for 40 min, under agitation, in MOPS buffer pH 7.4, in the presence of **19** (at concentrations of 10 μM to 1.25 μM). The incubated were transferred to a black 96-well ELISA plate (200 μL/well) and the substrate Z-Phe-Arg-AMC (carbobenzoxyl-l-phenylalanyl-l-arginine-7-amino-4-methylcoumarine) (10 μM) was then added. The untreated parasites and parasites treated with cysteine protease inhibitor E-64 (10 μM) were used as control. Proteolysis was monitored continuously by measuring the hydrolysis of the substrate in a Hitachi 7000 spectrofluorimeter, at 37 °C (λ_Ex_ = 380 nm; λ_Em_ = 460 nm). The activity observed corresponded to arbitrary fluorescence units (AFU) measured for 20 min. The data were analyzed by Two-Way ANOVA and Bonferroni post-test (*n* = 3). The enzyme activity of recombinant papain was measured by hydrolyzing the fluorogenic peptide substrate Z-l-phenylalanyl-l-arginine MCA (Z-Phe-Arg-AMC). Papain was incubated in assay buffer containing 10 μM of **19** and 100 mM sodium acetate (pH 5.0). The fluorescence release was monitored over 100 s at 37 °C using a Hitachi 7000 spectrofluorimeter, with excitation at λ = 380 nm and emission at λ = 460 nm.

#### 4.2.14 *P. berghei* Hepatic Stage Assay

The sensitivity of firefly luciferase-expressing *P. berghei* hepatic stages to the compound was assessed by measuring luminescence as previously described.^61^ In this assay, 10^4^ sporozoites, obtained from the dissection of salivary glands from infected *Anopheles stephensi* mosquitoes, were seeded into each well of a 96-well plate containing Huh-7 cells (10^4^ per well) cultured in RPMI 1640 medium supplemented with 10% fetal calf serum (FCS), 1% penicillin/streptomycin, 1% glutamine, 1% nonessential amino acids, and 10 mM HEPES. The compound was added 1 h before sporozoite addition, and the parasite load was determined through a bioluminescence assay (Biotium) after 46 h of exoerythrocytic growth at 37 °C and 5% CO2. The viability of Huh-7 cells exposed to the compound was evaluated using the AlamarBlue assay (Invitrogen, U.K.) prior to bioluminescence measurement. The percentage of infection relative to control was calculated, and IC_50_ values were determined using the Prism GraphPad software.

#### 4.2.15 Antimalarial Tests Against *P. berghei* in Mice

A suppressive parasite growth test was used with *P. berghei*, NK65 strain (originally received from the New York University Medical School) infected mice as described previously^62^ with some modifications.^63^ Briefly, male adult Swiss outbred mice (20 ± 2 g of weight) were inoculated with 5 × 10^5^ red blood cells infected with *P. berghei*, by intraperitoneal route. The infected mice were maintained together for at least 2 h and then divided randomly into groups of 3–5 animals per cage, which were subsequently treated with 50 mg/kg of **19** diluted in DMSO 3% (v/v) given daily by oral gavage for three consecutive days. Two control groups were used in parallel, one treated with CQ (20 mg/kg/day) and one with the vehicle. Blood smears from mouse tails were prepared on days 5, 8, and 11 postinfection and then methanol-fixed, stained with Giemsa and examined microscopically. The parasitemia was evaluated and the percent inhibition of parasite growth calculated in relation to the untreated control group (considered 100% growth) using the following equation: [(*C* – *T*)/*C*]; where *C* is the parasitemia in the control group and *T* is the parasitemia in the treated group. The use of laboratory animals was approved by the Ethic Committee on Animal Use of the Federal University of Sao Paulo (CEUA/UNIFESP 3004230222). In the *P. berghei* study, after day 11, animals were monitored daily and classified according to pre-established humane end point criteria. These criteria included: changes in body weight and food/water intake; external physical appearance (ungroomed and ruffled fur, closed eyelids, ocular/nasal discharge, hunched posture, etc.); clinical signs (alterations in heart and respiratory rates, dyspnea, changes in defecation, body temperature, etc.); unprovoked behavioral changes (e.g., vocalization, self-injury); and behavioral responses to external stimuli (e.g., aggressiveness).^64^ All institutional and national guidelines for the care and use of laboratory animals were followed.

### 4.3 Molecular Modeling

#### 4.3.1 Molecular Docking

The structures of FP-2a and FP-3 were obtained from the Protein Data Bank using the PDB codes 2OUL and 3BWK, respectively. The protonation states of the amino acids were assigned with the PDB 2PQR^65^ at pH 5.5, which aligns with the biochemical inhibition assays and is very similar to the pH observed in the DV of *P. falciparum*. For compound **19**, we checked its p*K*_a_ with molGpKa^66^ and fix p*K*_a_, along with experimental p*K*_a_ values of similar fragments present in **19**,^67^ indicating that the amine group would be predominantly protonated, resulting in a net charge of +1 for the ligand. Omega^68^ was employed to generate the Mol2 file of the ligand from SMILES format. The ligand was then docked into the active sites of FP-2a and FP3 using GOLD,^69^ with the binding pocket defined as all atoms within 10 Å away from the catalytic Cys residue of the proteases. Pose selection for subsequent molecular dynamics simulations was based on the ranking according to the ChemPLP score function and visual inspection of the ligand’s occupation of the S2 pocket. Noteworthy, based on Hernández González’s work,^70^ we retained a crystallographic water molecule in the S2 pocket of FP-2a to evaluate potential hydrogen bond interactions. We allowed the docking program to determine whether the water molecule should be retained or displaced. Nonetheless, in all predicted poses, no interactions with the water were observed, and when the S2 pocket was occupied by the ligand, the water molecule was displaced.

#### 4.3.2 Molecular Dynamics Simulations

The most relevant poses obtained in docking were used as starting points to perform the MD simulations, resulting in two putative binding poses for each of the two proteins (FP-2a and FP-3). To obtain the partial charges of **19** we have used the RESP approach, implemented in the Antechamber package, in which the electrostatic potential of the ligand was calculated using Gaussian16 software, with the 6-31G* basis set at the Hartree–Fock level of theory. Next, the missing bonded and nonbonded parameters were assigned using the general amber force field, similar to what Panciera et al. have done for inhibitors containing the 3H-pyrrolo­[2,3-*c*]­quinolines moiety.^71^ FP-2a and FP-3 parameters were taken from the ff14SB force field,^72^ and the ligand–protein complexes were solvated in a truncated octahedron TIP3P water^73^ box with an appropriate number of Na^+^ cations added to neutralize the system. For each system, initially, only the hydrogen atoms were minimized, followed by water molecules and ions using 10000 cycles of steepest descent and conjugate gradient algorithms. Subsequently, the entire system was minimized and gradually heated to 300 K in the NVT ensemble using a Langevin thermostat. This was followed by an NPT equilibration at 300 K, and finally, a 300 ns production run. In the end, a total production run of 1.2 μs was performed for the four different complexes, encompassing the two putative binding poses of compound **19** with FP-2a and FP-3. The all-atom MD simulations were performed using Amber24 ^74^ with *pmemd.cuda*. To analyze the trajectory for each system and most representative poses along with their centroids, *cpptraj* ^75^ was employed to perform k-means clustering into 10 clusters over 500 iterations based on the RMSD of the ligand and the protein, while excluding hydrogen atoms. Additionally, *cpptraj* was used to evaluate hydrogen bond interactions and conduct RMSD and RSMF analysis.

#### 4.3.3 TI Free Energy Calculations

The relative binding affinity of the transformation of the methoxy group of **19** into hydrogen was evaluated in Amber24 using the same combinations of force fields previously described. The ΔΔG for this transformation was calculated through the following equation

1 $$\Delta \Delta G_{OMe\to H}=\Delta G_{OMe\to H,total,protein}-\Delta G_{OMe\to H,total,water}$$

This mutation was performed to assess the importance of the OMe group, which occupies the S2 pocket, in inhibiting FP-2a by compound **19.** The thermodynamic cycle for the OMe → H transformation was constructed using a stepwise decharge–vdW–recharge protocol. In each of the steps, to connect the thermodynamic states, 11 λ-windows with an increment of 0.1 was employed. Each window underwent a 1 ns equilibrium phase followed by 5 ns for the production run to obtain the free energy for each window in all of the steps, all these calculations were performed in triplicate to ensure reliability. Finally, to analyze the outcomes of the alchemical calculations, the *alchemlyb* ^76^ python library was adopted using the thermodynamic integration as the estimator of the free energy.

## Supplementary Material

















## Data Availability

The data underlying this study are available in the published article and its online Supporting Information.

## Notes

Additional details on the synthesis and characterization of Bpin-pyrrole intermediates, additional synthetic methods, quantitative ^1^H NMR of compound **19**, ^1^H and ^13^C NMR spectra for target compounds (**6–29**), chiral chromatography data of compound **16**, baseline characteristics of the isolates for the ex vivo assay, liver-stage activity of **19**, methods for in vitro physicochemical and ADME studies, histogram of the fluorescence of **19** (PDF)
In silico docking against the target enzymes (In silico docking file 1, FP2a-19—Top-ranked pose) (PDB)
In silico docking file 2, FP3-19—Top-ranked pose (PDB)
In silico docking file 3, FP2a-19—S2 binding pose (PDB)
In silico docking file 4, FP3-19—S2 binding pose (PDB)
In silico docking file 5, FP2a-19—S2 binding pose after MD (PDB)
In silico docking file 6, FP3-19—S2 binding pose after MD (PDB)
Molecular formula strings (CSV)

## Abbreviations

AcCl: acetyl chloride
Acpc: 1-aminocyclopropanecarboxylic acid
Aib: 2-aminoisobutyric acid
ACT: artemisinin combination therapy
AFU: arbitrary fluorescence units
ANOVA: analysis of variance
AO: acridine orange
ART: artesunate
ATO: atovaquone
^13^C NMR: carbon-13 nuclear magnetic resonance
calcd: calculated
CC_50_: cytotoxic concentration of an inhibitor that is required for 50% inhibition in vitro
CG: cycloguanil
CLogP: calculated logarithm of the *n*-octanol/buffer distribution coefficient
CL int: intrinsic clearance
CQ: chloroquine
DAD: photodiode array detector
DIPEA: *N*,*N*-diisopropylethylamine
DL-NptGly: 2-Amino-4,4-dimethylpentanoic acid
DMF: *N*,*N*-dimethylformamide
DMSO: dimethyl sulfoxide
DMSO-*d*_6_: deuterated dimethyl sulfoxide
DV: digestive vacuole
EC_50_: effective concentration of an inhibitor that is required for 50% inhibition
E-64: cysteine protease inhibitor
∑FIC_50_: sum of the fraction concentration of a drug that is required for 50% inhibition in vitro
FP-2a: falcipain 2a
FP-3: falcipain 3
fu: unbound fraction
GMS: Greater Mekong Subregion
^1^H NMR: proton nuclear magnetic resonance
HATU: (1-[Bis­(dimethylamino)­methylene]-1*H*-1,2,3-triazolo­[4,5-*b*]­pyridinium 3-oxid hexafluorophosphate)
HEPES: 2-[4-(2-hydroxyethyl)­piperazin-1-yl]­ethanesulfonic acid
HepG2: hepatocellular carcinoma cells
HPLC: high pressure liquid chromatography
HRMS: high-resolution mass spectrometry
IC_50_: concentration of a drug that is required for 50% inhibition in vitro
iRBCs: infected red blood cells
LC-MS: liquid chromatography mass spectrometry
LogD_7.4_: logarithm of the *n*-octanol/buffer distribution coefficient at pH 7.4
LogS_7.4_: logarithm of solubility coefficient at pH 7.4
MD: molecular dynamics
MeOH: methanol
MP: melting point
MQ: marinoquinoline
MTT: 3-(4,5-dimethyl thiazol-2-yl)-2,5-diphenyltetrazolium bromide
*N*-Boc: tert-butyloxycarbonyl
ND: not determined
NptGly: neopentylglycine
PAMPA: parallel artificial membrane permeability assay
*P. berguei*: *Plasmodium berguei*
*Pb*: *Plasmodium berguei*
PDB: protein database
PPB: plasma protein binding
*P. falciparum*: *Plasmodium falciparum*
*Pf*: *Plasmodium falciparum*
*P. vivax*: *Plasmodium vivax*
*Pv*: *Plasmodium vivax*
*Pv*: *Plasmodium vivax*
PYR: pyrimethamine
RBC: red blood cell
RBFE: relative binding free energy
*R*_f_: retardation factor
RI: resistance index
RMSD: root-mean-square deviation
RPMI 1640: Roswell park memorial institute medium
RSA: ring survival assay
RSMF: root-mean-square fluctuation
SAR: structure–activity relationship
SD: standard deviation
SI: selectivity index
*t*_1/2_: half-life
TLC: thin layer chromatography
*po*: per oral
TMS: tetramethylsilane
TsOH: *p*-toluenesulfonic acid
tr: retention time
TFA: trifluoroacetic acid
TI: thermodynamic integration
UPLC: ultra performance liquid chromatography
UV: ultraviolet
vdW: van der Waals
WGSA: whole genome sequencing analysis
Z-Phe-Arg-AMC: Z-Phe-Arg 7-amido-4-methylcoumarin
δH: chemical shift in ^1^H NMR spectrum
δC: chemical shift in ^13^C NMR spectrum
*m*/*z*: mass to charge ratio.
