# Supplementary material for: Fluorescent Marinoquinoline Derivative as Inhibitors of Plasmodium falciparum: SAR Analysis, Mode of Action and In Vivo Studies
Source: J Med Chem. 2025 Sep 30;68(20):21120–43. doi: 10.1021/acs.jmedchem.5c00138 (PMC12557366; doi:10.1021/acs.jmedchem.5c00138)
Supplement: Supplementary file 1 [file jm5c00138_si_001.pdf]

Supporting Information

For

Fluorescent marinoquinoline derivative as inhibitors of *Plasmodium falciparum*: SAR analysis, mode of action and *in vivo* studies

Patricia Santos Barbosa[#a], Guilherme Eduardo Souza[#b], Sarah El Chamy Maluf[b], Vinícius Bonatto[b], Caio Silva Moura[c], Giovana Rossi Mendes[b], Talita Alvarenga Valdes[b], Yasmin Annunciato[c], Barbara dos Santos Rossetto[c], Priscilla Dantas de Souza Ventura[c], Gilberto Gaspar Duarte Ortin[a], Wellington da Silva[a], Marcelo Yudi Icimoto[d], Amália dos Santos Ferreira[e], Fabio C. Cruz[f], Carolina B. G. Teles[e], Dhelio B. Pereira[g], Gustavo Capatti Cassiano[h], Sofia Santana[i], Miguel Prudêncio[i,j], Camila S. Barbosa[k], Igor M. R. Moura[b], Renan Marcel Giampauli[L], Irene Layane De Sousa[L], Silvana Aparecida Rocco[L], Marcos L. Gazarini[c], Carlos Roque Duarte Correia[a], Anna Caroline Campos Aguiar*[c,k], Rafael Victorio Carvalho Guido*[b]*

[a] Chemistry Institute, University of Campinas (UNICAMP), 13083-970, Campinas, SP, Brazil.
[b] São Carlos of Physics Institute, University of São Paulo (USP), 13566-590, São Carlos, SP, Brazil.
[c] Department of Biosciences, Federal University of São Paulo (UNIFESP), 11015-020, Santos, SP, Brazil.
[d] Department of Biophysics, Federal University of Sao Paulo, (UNIFESP), Escola Paulista de Medicina, CEP 04023-062, São Paulo, SP, Brazil.
[e] Oswaldo Cruz Foundation, Leishmaniasis and Malaria Bioassay Platform, 76812-245, Porto Velho, RO, Brazil.
[f] Department of Pharmacology, Federal University of São Paulo (UNIFESP), Escola Paulista de Medicina, 04023-062, São Paulo, SP, Brazil.
[g] Research Center in Tropical Medicine of Rondônia, 76812-245 Porto Velho, RO, Brazil.
[h] Global Health and Tropical Medicine (GHTM), Associate Laboratory in Translation and Innovation Towards Global Health (LA-REAL), Instituto de Higiene e Medicina Tropical, (IHMT), Universidade NOVA de Lisboa (UNL), Lisbon, Portugal.
[i] Gulbenkian Institute for Molecular Medicine, 1649-035, Lisboa, Portugal
[j] Faculdade de Medicina da Universidade de Lisboa, 1649-028 Lisboa, Portugal.
[k] Department of Microbiology, Immunology and Parasitology, Federal University of São Paulo (UNIFESP), Escola Paulista de Medicina, 04023-062, São Paulo, SP, Brazil.
[L] Brazilian Biosciences National Laboratory and Brazilian Center for Research in Energy and Materials, 13083-100, Campinas, SP, Brazil.

# These authors contributed equally to this work
*Corresponding authors: rvcguido@usp.br, caroline.aguiar@unifesp.br, croque@unicamp.br

TABLE OF CONTENTS

EXPERIMENTAL PROCEDURES

Experimental procedures: Organic Synthesis ……………………………………………………………….Page S03
Experimental procedures: Physicochemical and pharmacokinetic analyses……………………………Page S07

FIGURES

Figure S1. Concentration-response curves for compounds **6-29** against *P. falciparum* (3D7 strain)…………………………………………………………………………………………......Pages S10-12
Figure S2. Concentration-response curves for compounds **6-29** against HepG2 cells……………. Pages S13-14
Figure S3. Kinetic solubility profiles of positive control alprenolol (**A**) compound **19** (**B**) determined after 0 and 1.5 h of incubation at 25 °C, across pH values of 1.7, 7.4, and 8.9. Data are presented as mean soluble fraction (%, ± SD)………………………………………………………………………………………………….Page S15
Figure S4. Chemical stability and solubility of positive control alprenolol (**A**) compound **19** (**B**) after 0, 1.5, and 24 h of incubation at 37 °C under acidic (pH 1.7), neutral (pH 7.4), and basic (pH 8.9) conditions. Results are shown as mean soluble fraction (%, ± SD)……………………………………………………………Page S15
Figure S5. Experimental LogD values at pH 7.4 for **19** compared with reference compounds tolbutamide and ketoconazole. Calculated LogD and LogP values for all compounds are also included for comparison.Page S15
Figure S6. Apparent permeability coefficients (P_app, $\times 10^{-6}$ cm/s) of **19** (green) and alprenolol (black) determined in the PAMPA assay at pH 5.5 and 7.4. Each bar represents the mean ± standard deviation (n = 3)………………………………………………………………………………………………...Page S16
Figure S7: Microsomal stability profiles of **19** (**A**) and verapamil (**B**) in liver microsomes from mouse (yellow), rat (magenta), and human (green). The percentage of parent compound remaining over time was determined by LC-MS/MS. Compound **19** (**A**) shows slow degradation and high metabolic stability, while verapamil (**B**) demonstrates rapid metabolism, especially in mouse microsomes………………………………………..Page S16.
Figure S8. (A) Comparison of fluorescence emission profiles for marinoquinoline analogs **10**, **11**, **18**, **19**, **22**, and **23** excited at 400 nm, indicating that **19** exhibits the strongest emission among the tested compounds. (**B**) Sensitivity curve for **19**……………………………………………………………………………………….. Page S17
Figure S9. Hemolytic activity of **19** (10 µM) in fresh human red blood cells after 24, 48, and 72 h of incubation. Saponin (0.1%) and DMSO were used as positive and vehicle controls, respectively…………………..Page S17
Figure S10. Liver-stage activity of compound **19** by *P. berghei* luciferase-expressing parasites………Page S18
Figure S11. Evaluation of the combination of **19** with E-64. The black line and gray region depict the additivity curve, while the red region and red dots represent the experimental data. Column (A) Isobolograms for the combinations. Column (B) Statistical analysis of the combinations. These panels show the ∑FIC50 values derived from three independent experiments. A p-value < 0.05 indicates a statistically significant difference between the experimental data and the additivity isobole………………………………………………………Page S18
Figure S12. RMSD of the **19**-FP2a complex, **19** and RMSF of the protein over 300 ns of simulation time, respectively. **A)** For the top-ranked pose by docking. **B)** For the S2 binding mode pose………………...Page S19
Figure S13. RMSD of the **19**-FP3 complex, **19** and RMSF of the protein over 300 ns of simulation time, respectively. **A)** For the top-ranked pose by docking. **B)** For the S2 binding mode pose………………Poage S19
Figure S14. Representative structures from clusters 1–10, derived from the 300 ns MD simulation of the top-ranked docking pose against FP2a. The distinct conformations highlight the structural variability observed throughout the simulation, emphasizing the challenge in identifying a definitive binding pose…………Page S20
Figure S15. Alignment of the scaffold for **19** representative structures from clusters 1–10, derived from the 300 ns MD simulation of the top-ranked docking pose against FP2a…………………………………………Page S21
Figure S16. Representative structures from clusters 1–10, derived from the 300 ns MD simulation of the top-ranked docking pose against FP3. The distinct conformations highlight the structural variability observed throughout the simulation, emphasizing the challenge in identifying a definitive binding pose…………..Page S22

Figure S17. Alignment of the scaffold for **19** representative structures from clusters 1–10, derived from the 300 ns MD simulation of the top-ranked docking pose against FP3……………………………………………Page S23
Figure S18. HPLC purity and quantitative ^{1}H NMR of compound **19** employed in biochemical and *in vivo* assays ………………………………………………………………………………………………… Pages S24-25
Figures S19-S64. ^{1}H and ^{13}C NMR spectra of the tested compounds ……………………...............Pages S26-S48
Figure S65. Chiral chromatography data of compound (16) ……………………………………….. ...Page S49

TABLES

Table S1. Kinetic solubility of compound **19** and the positive control alprenolol following 0 and 1.5 h of incubation at 25 °C in aqueous buffers at pH 1.7 (acidic), 7.4 (neutral), and 8.9 (basic). Results are expressed as mean soluble fraction ± standard deviation……………………………………………………………….................. Page S50
Table S2. Summary of the chemical stability and solubility of compound 19 and the positive control alprenolol after 0, 1.5, and 24 h of incubation at 37 °C in acidic (pH 1.7), neutral (pH 7.4), and basic (pH 8.9) aqueous buffers. Data are expressed as mean soluble fraction (% of initial concentration) ± standard deviation...Page S50
Table S3. Predicted and experimental $LogD_{7.4}$ determined using the shake-flask method for **19** and reference compounds (tolbutamide and ketoconazole)………………………………………………………………Page S51
Table S4: *In vitro* metabolic stability parameters of **19** and verapamil (control) in liver microsomes from mouse, rat, and human…………………………………………………………………………………………Page S51
Table S5. Baseline characteristics of the isolates for the *ex vivo* assay……………………………………Page S51
Table S6: Outcomes of all FEP transformation steps in triplicate run of the **19**-FP2a complex…………Page S52
References …………………………………………………….. Page S52

1. SYNTHETIC PROCEDURES

1.1. Synthesis and characterization of Bpin-pyrrole intermediate (S3).

Scheme S1. Synthetic route to obtain pyrrole intermediate (S3).

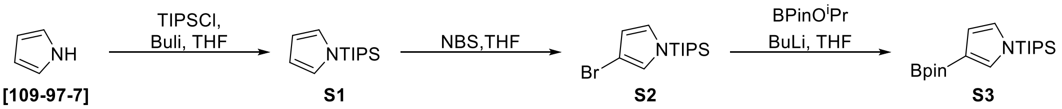

Synthesis of 1-(triisopropylsilyl)-1*H*-pyrrole (S1)[1]

To a solution of pyrrole (60 mmol, 4.03 g) in anhydrous THF (100 mL) at -78 °C was added BuLi (1.6 M in hexanes) (66 mmol, 41.3 mL) dropwise over 30 minutes. In sequence, triisopropylsilyl chloride (60 mmol, 12.9 mL) was added dropwise and the resulting reaction mixture was stirred for 3 hours at -78 °C. After that time, the reaction mixture was warmed gradually to room temperature and stirred for additional 30 minutes. The solvent was concentrated, water (100 mL) was added to the residue and the product was extracted with diethyl ether (3 x 100 mL). The combined organic layers were washed with water and brine, dried over Na_2SO_4, and concentrated to give the crude product as a dark brown oil. The crude material was purified by flash chromatography over silica eluting with a gradient of ethyl acetate (0–5%, v/v) in hexanes to give the corresponding **S1** as a colourless oil in 91% yield (12.2 g). **^{1}H NMR (250 MHz, $CDCl_3$)** δ 6.86 (d, *J* = 1.6 Hz, 2H), 6.38 (d, *J* = 1.7 Hz, 2H), 1.61 – 1.40 (m, 3H), 1.16 (d, *J* = 7.4 Hz, 18H). **^{13}C NMR (63 MHz, $CDCl_3$)** δ 124.1, 110.2, 17.9, 11.9.

Synthesis of 3-bromo-1-(triisopropylsilyl)-1*H*-pyrrole (S2)[1]

To a solution of **S1** (12.2 g, 54.6 mmol) in anhydrous THF (120 mL) at -78 °C was added freshly recrystalised *N*-bromosuccinimide (54.6 mmol, 9.7 g). The resulting reaction mixture was kept at -78 °C for 3 hours and then warmed gradually to room temperature for 13 hours. The solvent was concentrated, and the crude product was taken up in hexane, filtered through a plug of neutral alumina and concentrated to yield the corresponding **S2** as a colourless oil in 90% yield (14.9 g). **Rf** 0.71 (hexanes 100%). **^{1}H NMR (250 MHz, $CDCl_3$)** δ 6.75 – 6.70 (m, 1H), 6.67 (t, *J* = 2.5 Hz, 1H), 6.29 (dd, *J* = 2.8, 1.4 Hz, 1H), 1.48 – 1.33 (m, 3H), 1.09 (d, *J* = 7.4 Hz, 18H). **^{13}C NMR (63 MHz, $CDCl_3$)** δ 124.8, 123.5, 113.2, 98.0, 17.9, 11.7.

Synthesis of 3-(4,4,5,5-tetramethyl-1,3,2-dioxaborolan-2-yl)-1-(triisopropylsilyl)-1*H*-pyrrole (S3) [2]

To a solution of **S2** (49.14 mmol, 14.9 g) in anhydrous THF (246 mL) at -78 °C was added BuLi (1.6M in hexanes) (98.3 mmol, 61.4 mL) dropwise over 20 minutes. The resulting mixture was stirred for 1 hour at -78°C and subsequently, 2-isopropoxy-4,4,5,5-tetramethyl-1,3,2-dioxaborolane (294.1 mmol, 60 mL) was added dropwise over 20 minutes. The resulting reaction mixture was stirred for 3 hours at -78 °C then warmed gradually to room temperature for 13 hours. The reaction was quenched by addition of a saturated aqueous NH_4Cl solution (150 mL) and the resulting mixture was extracted with diethyl ether (3 x 150 mL). The combined organic phases were dried over Na_2SO_4 and concentrated under reduced pressure. The crude corresponding **S3** was employed in next step without further purification. **Rf** 0.56 (Hex: AcOEt 19:1). **^{1}H NMR (500 MHz, $CDCl_3$)** δ 7.25 – 7.22 (m, 1H), 6.82 – 6.80 (m, 1H), 6.62 (dd, J = 2.6, 1.1 Hz, 1H), 1.46 (hept, J = 7.5 Hz, 3H), 1.32 (s, 12H), 1.09 (d, J = 7.5 Hz, 18H). **^{13}C RMN (125 MHz; $CDCl_3$)** δ 133.8, 125.1, 115.8, 82.9, 25.0, 18.0, 11.8.

1.3 Synthesis and characterization of the methoxylated pyrroloaniline (1).

Scheme S2. Synthesis and characterization of the pyrroloaniline (1).

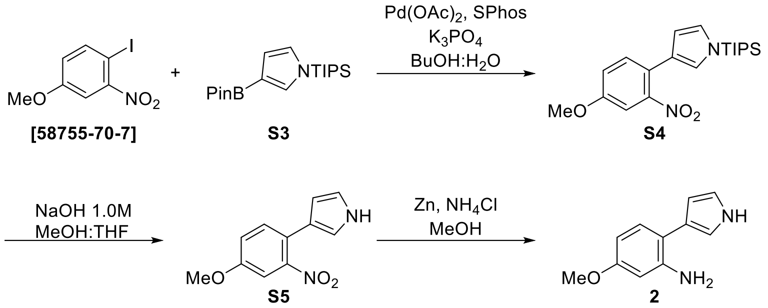

Synthesis of 3-(4-methoxy-2-nitrophenyl)-1-(triisopropylsilyl)-1*H*-pyrrole (S4).[3]

A Schlenk tube was charged with $Pd(OAc)_2$ (0.276 mmol, 62.0 mg), SPhos (0.552 mmol, 226.6 mg), **S3** (20.7 mmol, 4.60 g), 1-Iodo-4-methoxy-2-nitrobenzene (13.8 mmol, 3.85 g) and K_3PO_4 (27.6 mmol, 5.86 g). The Schlenk tube was capped with a rubber septum and then evacuated and backfilled with N_2. *n*-Butanol (19.7 mL) and water (7.9 mL) were added, and the reaction mixture was heated to 80 °C for 18 hours. The reaction mixture was cooled to room temperature and was then filtered through a thin pad of silica gel (eluting with ethyl acetate) and the eluent was concentrated under reduced pressure. The crude material was purified by flash chromatography over silica eluting with a gradient of ethyl acetate (0-10%, v/v) in hexanes to give the corresponding **S3** as a dark brown oil in 89% yield (4.61 g). **^{1}H RMN (400 MHz, $CDCl_3$)** δ 7.45 (d, *J* = 8.7 Hz, 1H), 7.14 (d, *J* = 2.7 Hz, 1H), 7.06 (dd, *J* = 8.6, 2.7 Hz, 1H), 6.88 – 6.84 (m, 1H), 6.81 – 6.77 (m, 1H), 6.38 (dd, *J* = 2.7, 1.5 Hz, 1H), 3.84 (s, 3H), 1.53 – 1.41 (m, 3H), 1.13 (d, *J* = 7.7 Hz, 18H).

Synthesis of 3-(4-methoxy-2-nitrophenyl)-1*H*-pyrrole (S5).

To a solution of **S4** (12.3 mmol, 4.61 g) in a mixture of MeOH:THF (1:1, 120 mL) was slowly added NaOH (60 mL, 1.0 M). The resulting reaction mixture was stirred at room temperature for 16 hours. After this period, the organic solvent was removed under reduced pressure. The pH was neutralized with citric acid and the aqueous phase was extracted with EtOAc (3 x 60 mL). The combinated organic layers were washed with brine, dried over Na_2SO_4 and concentrated. The crude **S5** was obtained as a dark brown solid (12 mmol, 2.62 g) and employed in the next step without further purification. **^{1}H RMN (250 MHz, $CDCl_3$)** δ 8.36 (s, 1H), 7.41 (d, *J* = 8.6 Hz, 1H), 7.15 (d, *J*= 2.6 Hz, 1H), 7.07 (dd, *J* = 8.6, 2.7 Hz, 1H), 6.90 (dd, *J* = 4.2, 1.9 Hz, 1H), 6.81 (dd, *J* = 4.8, 2.5 Hz, 1H), 6.27 (dd, *J* = 4.3, 2.6 Hz, 1H), 3.86 (s, 3H). **^{13}C RMN (63 MHz, $CDCl_3$)** δ 158.1, 149.6, 132.2, 122.5, 119.1, 118.8, 118.4, 116.5, 108.6, 108.3, 56.0.

Synthesis of 5-methoxy-2-(1*H*-pyrrol-3-yl)aniline (2).

To a solution of **S5** (12 mmol, 2.62 g) in MeOH (200 mL) was added a solution of NH_4Cl (1.09 g, 20.4 mmol in 6 mL of H_2O). The resulting mixture was vigorously stirred and Zn dust (186 mmol, 12.16 g) was added. The resulting reaction mixture was stirred at room temperature for 3 hours. After that time, the reaction mixture was filtered through a short pad of Celite and washed with EtOAc (100 mL) and MeOH (1 x 100 mL). The filtered was dried over Na_2SO_4 and the solvent was evaporated. The crude material was purified by flash chromatography over silica eluting with a gradient of ethyl acetate (30-50%, v/v) in hexanes to give the corresponding pyrroloaniline **2** as a brown solid in 93% yield (2.1 g). **^{1}H NMR (250 MHz, $CDCl_3$)** δ 8.40 (sl, 1H), 7.17 (d, *J* = 8.3 Hz, 1H), 6.90 (dd, *J* = 3.8, 1.8 Hz, 1H), 6.85 (dd, *J* = 4.8, 2.5 Hz, 1H), 6.44 – 6.38 (m, 2H), 6.35 (d, *J* = 2.4 Hz, 1H), 3.99 (s, 2H), 3.80 (s, 3H). **^{13}C NMR (63 MHz, $CDCl_3$)** δ 159.3, 145.0, 130.8, 121.6, 118.5, 116.0, 115.6, 108.8, 104.2, 101.2, 55.3.

1.4. Synthesis of the advanced marinoquinoline intermediate (5)

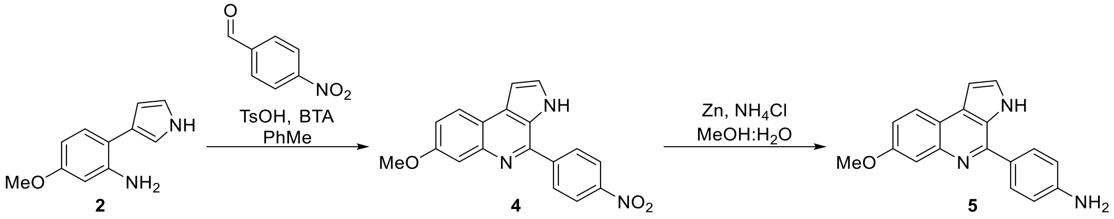

Synthesis of 7-methoxy-4-(4-nitrophenyl)-3*H*-pyrrolo[2,3-*c*]quinoline (4).[4]

To a solution of the aryl pyrrole **2** (1 mmol, 188.2 mg) and benzotriazole (1 mmol, 119.1 mg) in anhydrous toluene (4.0 mL) were added the 4-nitrobenzaldehyde (1.2 mmol, 181.3 mg), and TsOH (0.1 mmol, 19.02 mg). The resulting reaction mixture was stirred for 16 hours at room temperature and open flask. After this period, the reaction was quenched by the addition of saturated $NaHCO_3$ solution (30 mL) and the aqueous phase was extracted with EtOAc (3 x 50 mL). The organic layer was washed with water (20 mL), brine (20 mL) and dried over Na_2SO_4. The solvent was concentrated under reduced pressure and the crude was then purified by flash chromatography over silica (10g cartridge) eluting with a gradiente of ethyl acetate (0-20%, v/v) in hexanes to give the corresponding compound **4** as a yellow gummy in 54 % yield (172.4 mg). **Rf** 0.45 (Hex: EtOAc 7:3). **^{1}H NMR (400 MHz, $CDCl_3$)** δ 8.78 (brs, 1H), 8.46 (d, *J* = 8.7 Hz, 2H), 8.19 (d, *J* = 8.7 Hz, 2H), 8.15 (d, *J* = 8.9 Hz, 1H), 7.63 (d, *J* = 2.5 Hz, 1H), 7.49 (t, *J* = 2.8 Hz, 1H), 7.31 (dd, *J* = 8.9, 2.6 Hz, 1H), 7.14 – 7.09 (m, 1H), 3.98 (s, 3H).

HPLC-UV Analysis

Chromatographic analyses were performed on a Waters Alliance 2695 HPLC system (Milford, MA, USA) equipped with a quaternary pump, autosampler, in-line degasser, and a Waters 2996 photodiode array (PDA) detector. Data acquisition and processing were performed using Empower 2002 software. The separation was achieved using a reversed-phase Luna C18 analytical column (150 × 4.6 mm i.d., 5 µm particle size; Phenomenex), preceded by a guard column of the same stationary phase (4 × 3 mm i.d.). Details of the chromatographic conditions are provided in Table 1.

HPLC-UV Conditions for the Quantitative Analysis of Compound 19

HPLC system	Waters Alliance 2695 (Milford, MA, USA), equipped with quaternary pump, sample manager and degasser		
Detector	Waters 2996 Uv-Vis set in 210-400 nm range		
System control, data acquisition, and processing	Waters Empower 2002 chromatography software		
Column	Reversed-phase Luna C18<br>(150 x 4.6 mm I.D.; 5µm particle size)<br>Phenomenex		
Guard Column	Reversed-phase Luna C18 (4 x 3 mm I. D.)<br>Phenomenex		
Mobile phase	Solvent A- Acetonitrile<br>Solvent B- 0.10 M ammonium acetate,<br>pH 7.0		
Isocratic conditions	Time (min.)	A (%)	B (%)
	5	60	40
Flow	1.0 mL/min		
Injection volume	25 µL		
Temperature	25°C		
CPD 19 and Alprenolol Detections	UV@ 254 and 270 nm		
Run time	5 minutes		

PHYSICOCHEMICAL AND PHARMACOKINETIC ANALYSES

Kinetic Solubility and Chemical Stability Assays

The solubility and chemical stability of **19** were evaluated under simulated physiological pH conditions. Kinetic solubility was assessed at a final concentration of 200 µM, prepared by diluting a 20 mM stock solution in DMSO. The final DMSO content was adjusted to 1% (v/v) in all assay media. Experiments were carried out in aqueous buffer systems at pH 1.7 (Clark-Lubs buffer, 0.2 M), pH 7.4 (sodium phosphate buffer, 0.1 M), and pH 8.9 (Tris buffer adjusted with 2 M hydrochloric acid), mimicking gastric, plasma, and intestinal conditions, respectively. Solubility assays were performed at 25 °C using a thermal mixer (Eppendorf), with incubation times of 0 and 1.5 h. Following incubation, samples were analyzed by HPLC-UV using the conditions described in Table 1. Solubility was determined by comparing chromatographic peak areas of compound 19 in the test media with those from fully soluble reference solutions.

Chemical stability was evaluated under the same buffer conditions at 37 °C, with incubation times of 0, 1.5, and 24 h. Stability was monitored by comparing the peak areas at each time point to those at zero. All experiments were conducted in triplicate. Quantification of **19** was based on a calibration curve prepared from serial dilutions of the 20 mM stock solution in DMSO, resulting in final concentrations of 400, 200, 100, 50, 25, 10, 5, and 2.5 µM. The linear regression equation (y = ax + b) derived from the curve was used to calculate the concentrations in experimental samples.

Lipophilicity Determination by Shake-Flask Method: Log D at pH 7.4

The determination of Log D values was performed using the shake-flask method, following the guidelines established by OECD 107 (1995)[5] and EPA (1996)[6]. Prior to partitioning, phosphate buffer (pH 7.4) was saturated with n-octanol, and conversely, n-octanol was saturated with the aqueous phase. Both solutions were mechanically stirred (magnetic stirring, Corning stirrer) at room temperature for 24 hours and allowed to stand for an additional 24 hours to ensure complete phase separation. Partition systems were prepared in triplicate using different aqueous-to-organic phase ratios: 1:1 and 10:1 for tolbutamide (used as the positive control for aqueous solubility); 100:1 and 300:1 for **19** and ketoconazole, the latter selected as a highly lipophilic reference compound with physicochemical properties comparable to **19**. Stock solutions of each compound were prepared at 20 mM in DMSO. From these, 30 µM standard working solutions in phosphate buffer (pH 7.4) were prepared and added to each biphasic system. Samples were equilibrated by shaking for 90 minutes at room temperature using a roller shaker (Basic First Lab Orbital). After equilibration, the phases were allowed to separate, and the aqueous phase was collected and analyzed by HPLC-UV for compound quantification, using previously optimized chromatographic conditions. The predicted cLogD and cLogP values for **19** were calculated using Drug Metabolism (DM) software and ChemDraw platforms.

Permeability Assay using Parallel Artificial Membrane Permeability Model (PAMPA)

The parallel artificial membrane permeability assay (PAMPA) was conducted using a 96-well pre-coated plate system (Corning® BioCoat™ PAMPA Plate System). alprenolol (reference compound) and **19** were prepared by diluting 10 mM DMSO stock solutions in phosphate-buffered saline (PBS, pH 5.5) and (PBS, pH 7.4) to a final concentration of 100 µM, ensuring a final DMSO concentration below 1%. A volume of 200 µL of each solution was added to the donor wells, while 300 µL of PBS was added to the corresponding acceptor wells.

The donor and acceptor plates were assembled and incubated at 37 °C for 5h. Prior to incubation, aliquots of the initial donor solution (T0) were collected and stored at −20 °C. At the end of the incubation period, samples were collected from both donor and acceptor compartments and transferred to plastic tubes containing

30% (v/v) acetonitrile. T0 samples were processed in the same manner. Compound concentrations in the donor, acceptor, and T0 samples were quantified by HPLC-UV, using the chromatographic conditions described in Table 1. The apparent permeability coefficient (P_{app}) was calculated according to the standard PAMPA equation. All experiments were performed in triplicate. Chromatograms for **19** and alprenolol were acquired under identical analytical conditions. The data presented correspond to the calibration curve (range: 150 to 2.5 µM), which was used to quantify compound concentrations in the samples collected from both donor and acceptor compartments (upper and lower fractions) of the PAMPA plate, under the different pH conditions tested.

Plasma Stability Assay

The plasma stability[7–10] of **19** was assessed to ensure that degradation would not interfere with subsequent plasma protein binding analysis. Stock solutions (10 mM in DMSO) were diluted to 500 µM in acetonitrile and further adjusted to a final concentration of 5 µM in plasma. Samples were incubated at 37 °C for up to 6 h. Aliquots were collected at 0, 120, 240, and 360 min, followed by protein precipitation with acetonitrile and centrifugation at 10,000 rpm for 5 min. Supernatants were transferred to vials and analyzed by LC-MS/MS [19–22].

Plasma Protein Binding by Equilibrium Dialysis

Plasma protein binding was determined using an equilibrium dialysis method with RED Device Inserts (Plasma Protein Binding Equilibrium Dialysis, Thermo Fisher Scientific), following the manufacturer's instructions.[7–10] Compound **19** and the reference compound verapamil were evaluated individually. Each compound was prepared at a final concentration of 5 µM in plasma. Stock solutions (10 mM in DMSO) were diluted to 500 µM in acetonitrile, and 5 µL of this solution were added to 495 µL of plasma (final solvent composition: 0.05% DMSO, 0.95% acetonitrile). The RED plates were incubated at 37 °C in an orbital shaker (1.5 g) for 6 hours. At the end of incubation, aliquots were collected from both the donor (plasma) and receptor (PBS) compartments, quenched with 300 µL of acetonitrile, and centrifuged at 10,000 rpm for 5 minutes. The supernatants were analyzed by LC-MS/MS.
The fraction unbound (fu) was calculated using the following equation:

$$fu = AUC_{PBS\ (Receptor)} / AUC_{Plasma\ (Donor)}$$

***In Vitro* Metabolic Stability in Human, Rat and Mouse Liver Microsomes**

The in vitro metabolic stability of compound **19** was evaluated in pooled human liver microsomes (HLM, 20 mg/mL), Sprague-Dawley rat liver microsomes (RLM, 20 mg/mL), and CD-1 mouse liver microsomes (MLM, 20 mg/mL), all purchased from GIBCO (Thermo Fisher Scientific). Stock solutions of compound **19** and the reference compound verapamil were prepared at 10 mM in DMSO and diluted appropriately in buffer. Compound **19** was tested at a final concentration of 0.5 µM, and verapamil at 1 µM. The final DMSO concentration was kept at 0.1% (v/v).

Microsomal incubations were performed in plastic tubes at 37°C, in a total volume of 200 µL, using 100 mM phosphate buffer (pH 7.4) and 1 mM NADPH to initiate the reaction. Microsomal protein concentration was 1.0 mg/mL for compound 19 and 0.25 mg/mL for verapamil. Samples were withdrawn at 0, 5, 15, 30, and 60 minutes. Microsomal stability assays were performed in triplicate. Each reaction was quenched with ice-cold acetonitrile containing 30% of the final volume, followed by centrifugation at 10,000 rpm for 10 minutes to precipitate proteins. The supernatants were analyzed by LC–MS/MS. Chromatographic separation was performed on an Acquity UPLC BEH C18 column (2.1 × 10 mm, 1.7 µm particle size), maintained at 40 °C. The

mobile phases were (A) water with 0.1% formic acid and (B) acetonitrile with 0.1% formic acid. A binary gradient was used as follows: 0–1 min: 90% A; 1–4 min: 40% A; 4–5 min: 40% A; 5–6 min: 90% A. The flow rate was 0.4 mL/min, and the injection volume was 1 μL. Total run time was 6 minutes. Compound **19** was monitored in MRM mode using the precursor ion m/z 403.24, with product ions m/z 246.97, 290.07, and 358.13. Verapamil was monitored using the precursor ion m/z 455.28 and product ions m/z 149.95, 164.97, and 303.11.

The analytes peak areas were normalized to the time zero value (set as 100%) to determine the percentage of compound remaining at each time point. The elimination rate constant (k_el) was obtained from the slope of the linear regression of the natural logarithm of the percentage remaining versus time.[11–14] The in vitro half-life ($t_{1/2}$, min) was calculated as:

$$t_{1/2} = LN2/Kel$$

The intrinsic microsomal clearance (CL_int,mic, in μL/min/mg protein) was calculated using:

$$CL_{int,mic} = (LN2/t_{1/2}) * VI/PM$$

where VI is the incubation volume (μL) and PM is the microsomal protein concentration (mg). To extrapolate intrinsic clearance to hepatic intrinsic clearance ($CL\__{int,hep}$), [11–14] the following equation was used:

$$CL_{int,hep} = CL_{int,mic} * ML * LB$$

where ML is the microsomal protein yield (mg microsomal protein/g liver) and LB is the liver weight (g liver/kg body weight).

The hepatic clearance (CL_{hep}) was calculated using the well-stirred model according to the following equation[12,13]:

$$CL_{hep} = (Q_h * f_u * CL_{int,hep})/(Q_h + f_u * CL_{int,hep})$$

where:

- Qh is the hepatic blood flow ($mL \cdot min^{-1}\ kg^{-1}$), specific to each species;
- fu is the fraction of unbound compound to plasma proteins;
- $CL_{int,hep}$ is the intrinsic hepatic clearance;
- The resulting clearance values are expressed as $mL \cdot min^{-1}\ kg^{-1}$

6

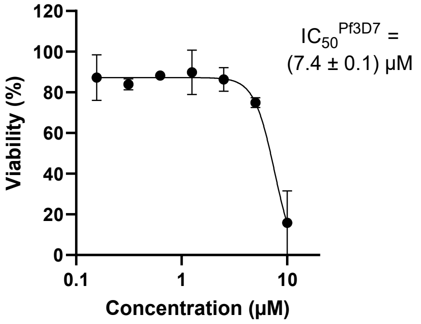

7

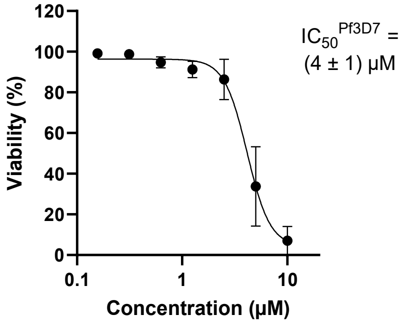

8

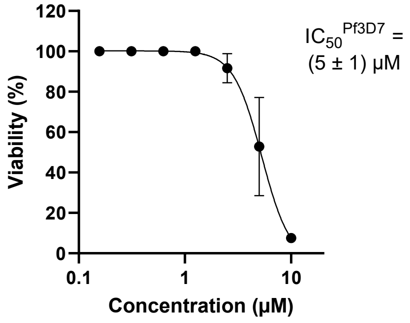

9

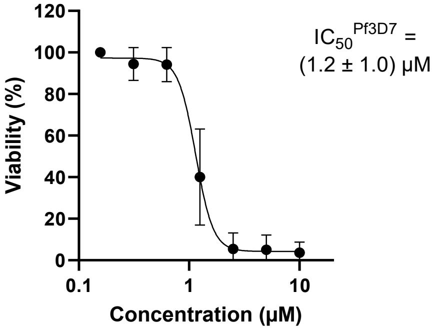

10

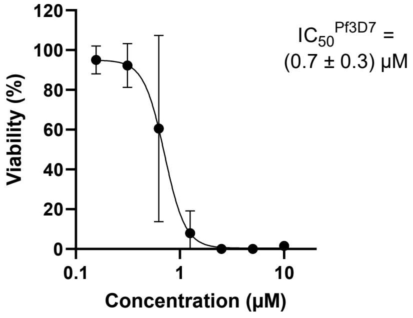

11

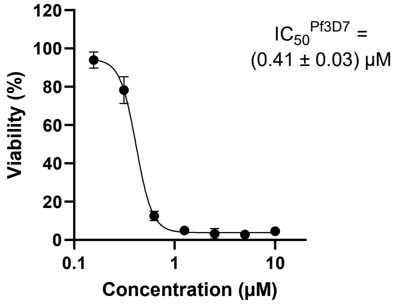

12

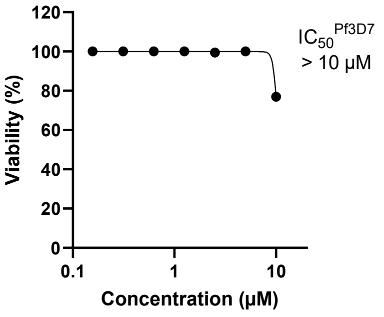

13

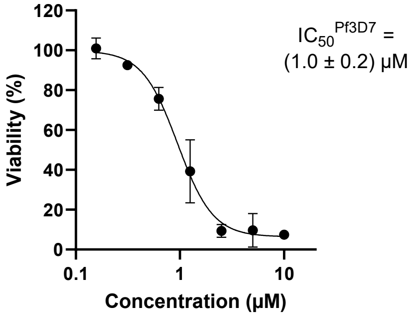

14

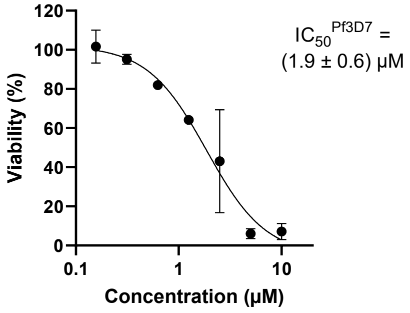

15

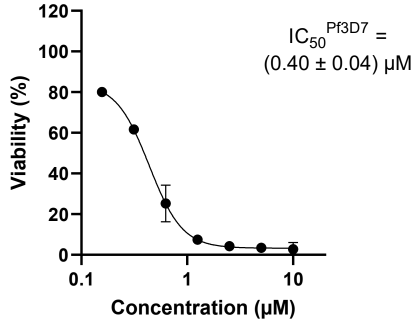

16

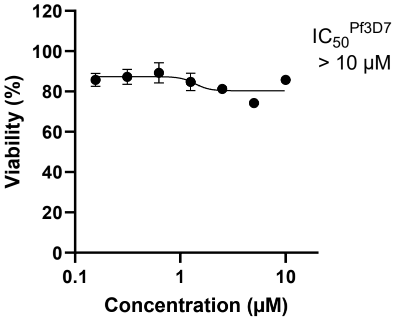

17

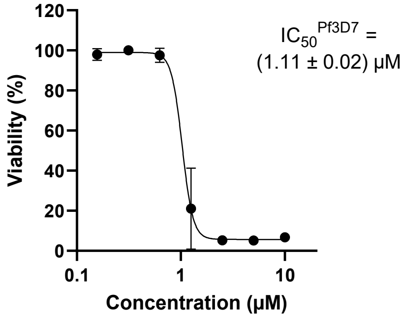

18

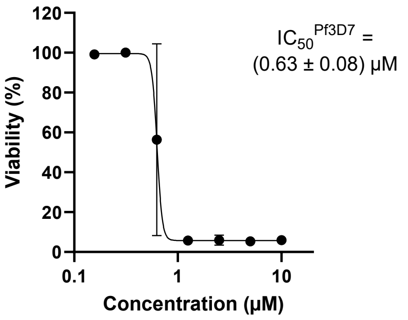

19

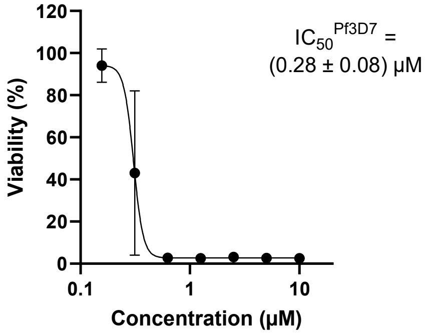

20

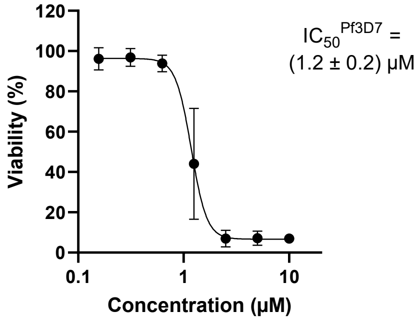

21

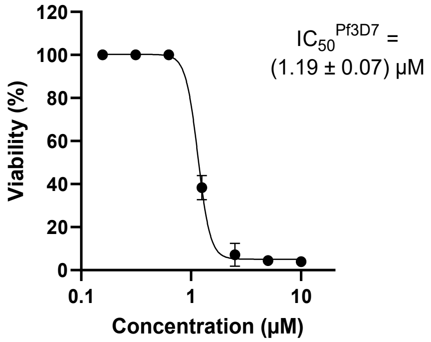

22

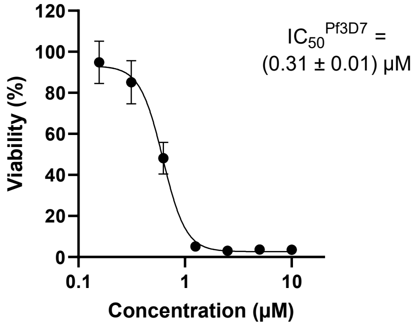

23

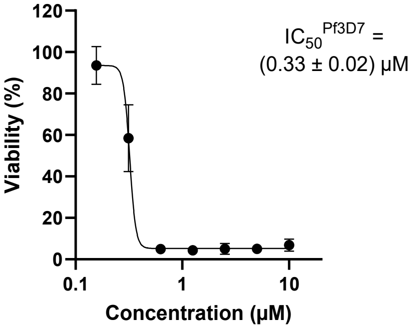

24

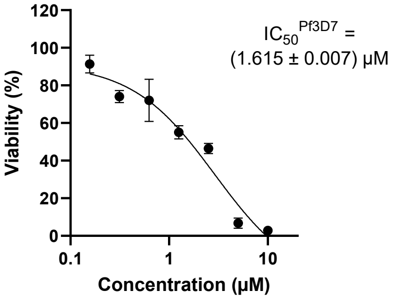

25

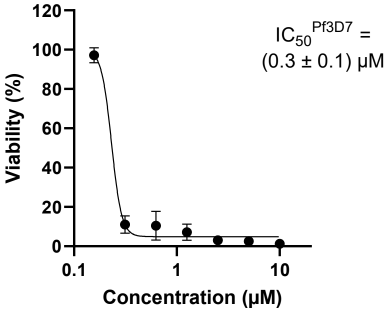

26

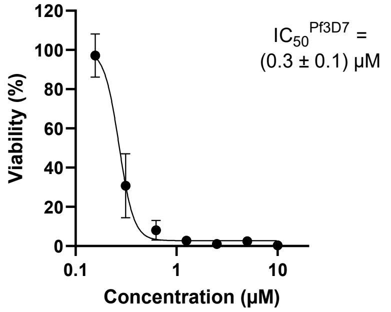

27

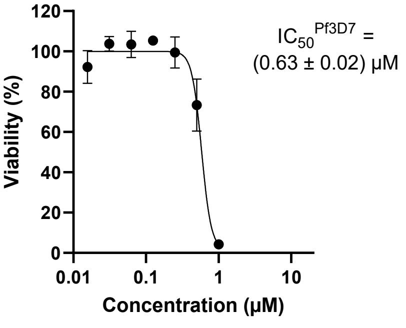

28

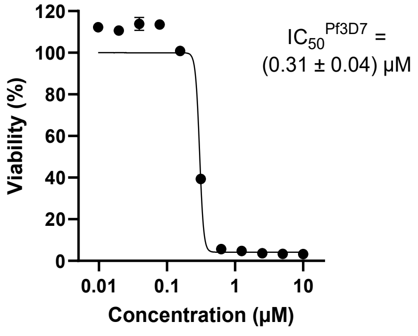

29

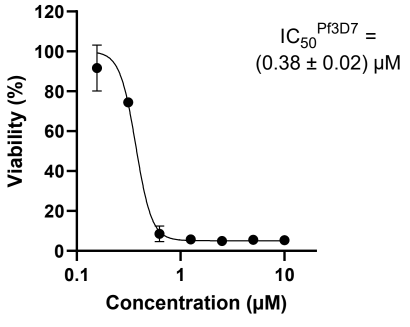

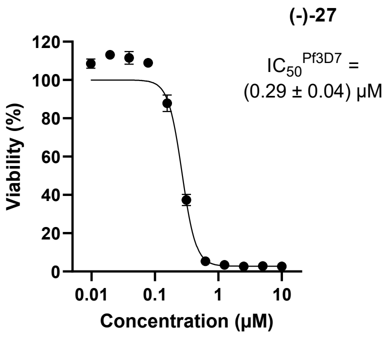

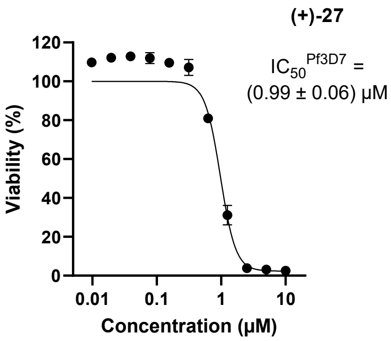

Figure S1. Concentration-response curves for compounds **6-29** against *P. falciparum* (3D7 strain).

6

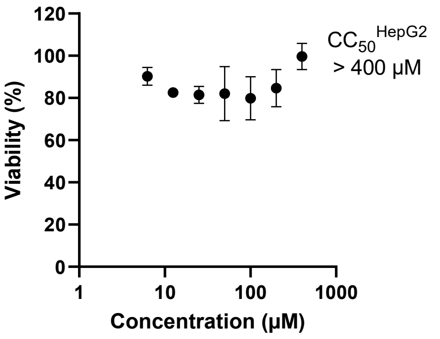

7

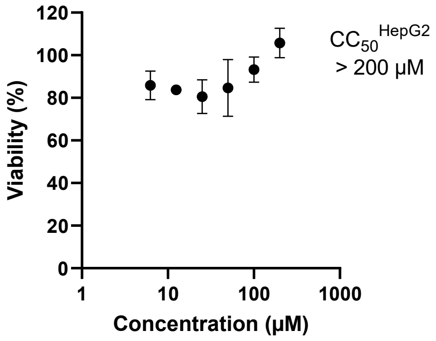

8

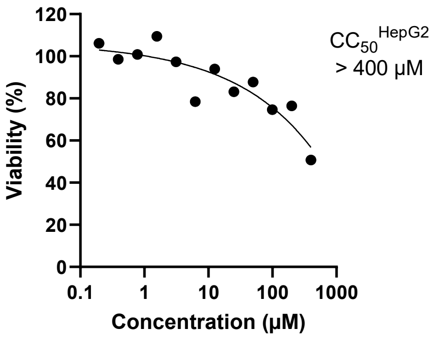

9

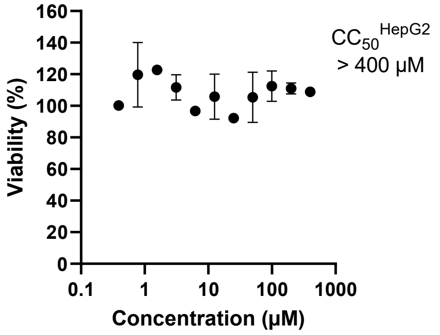

10

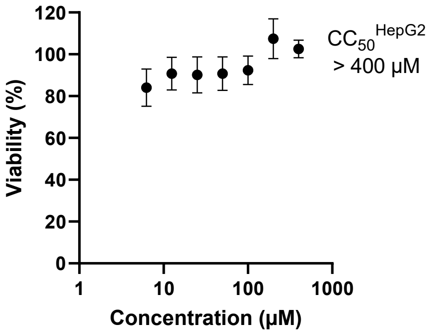

11

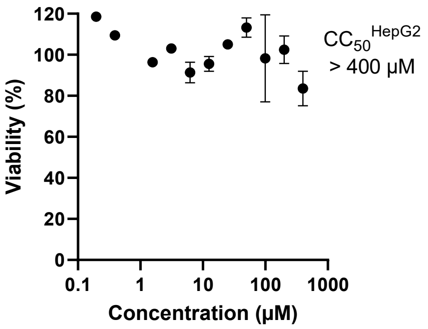

13

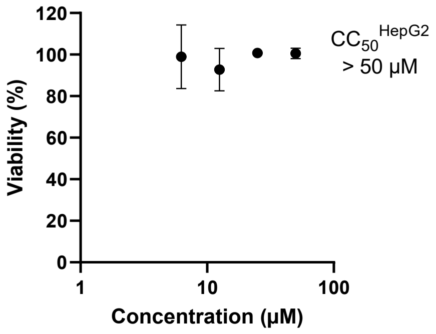

14

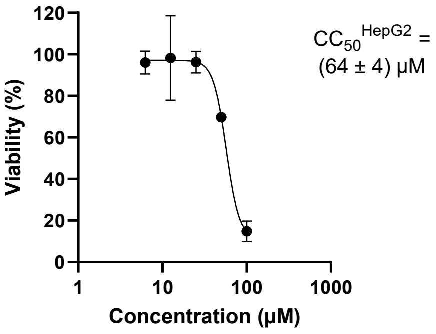

15

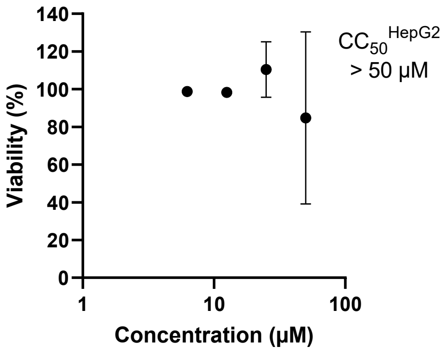

17

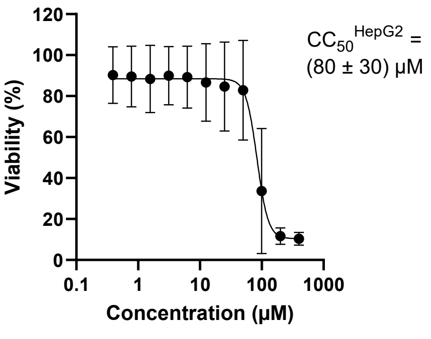

18

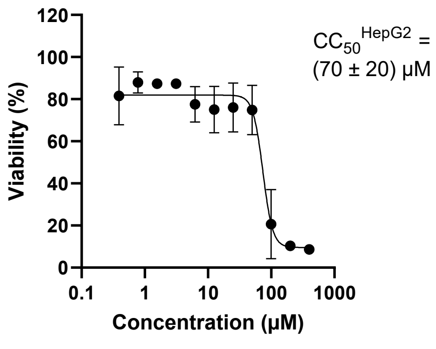

19

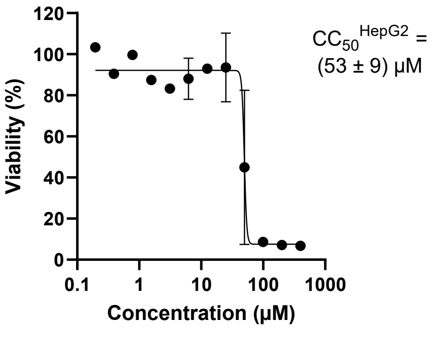

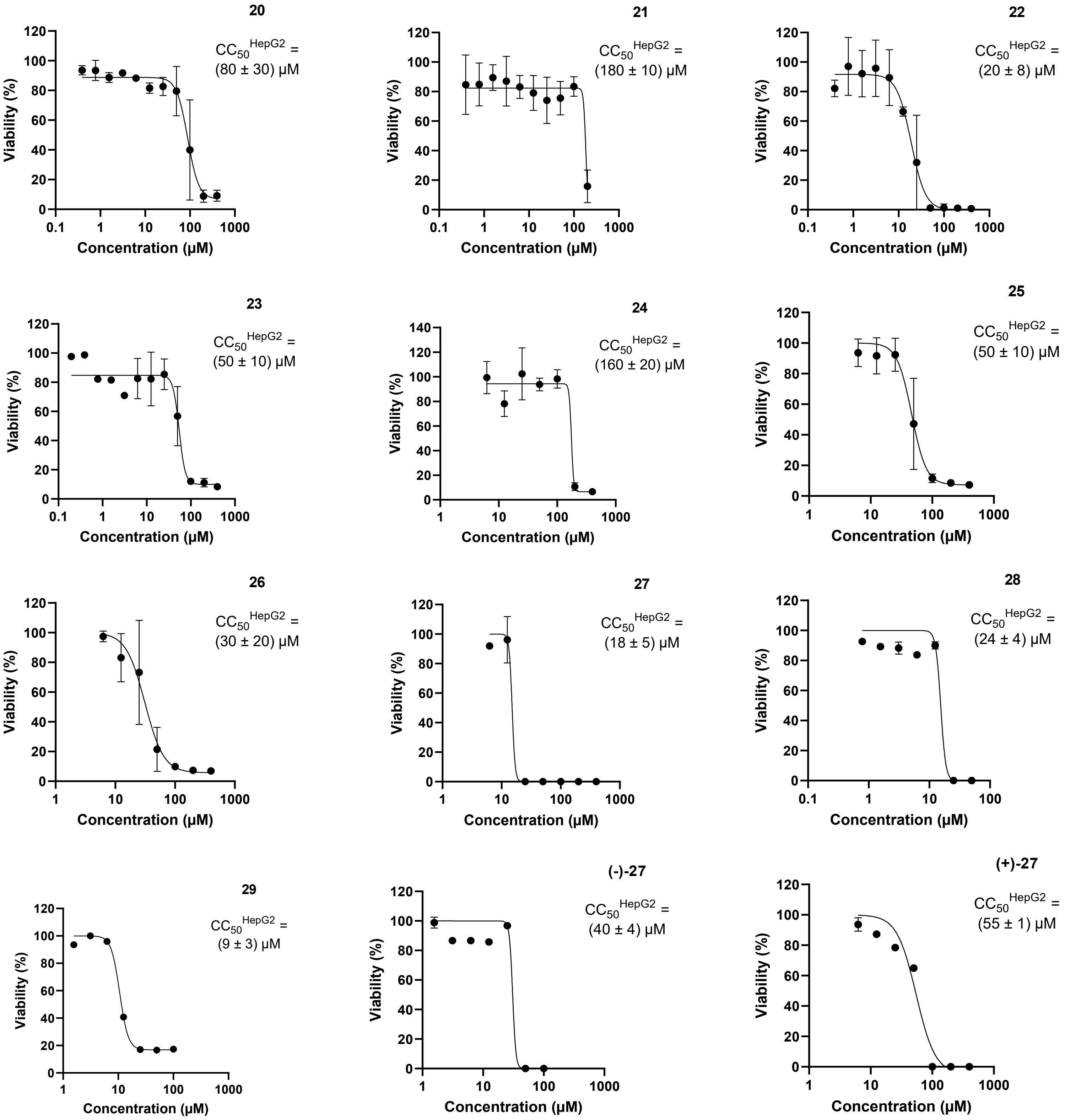

Figure S2. Concentration-response curves for compounds **6-29** against HepG2 cells.

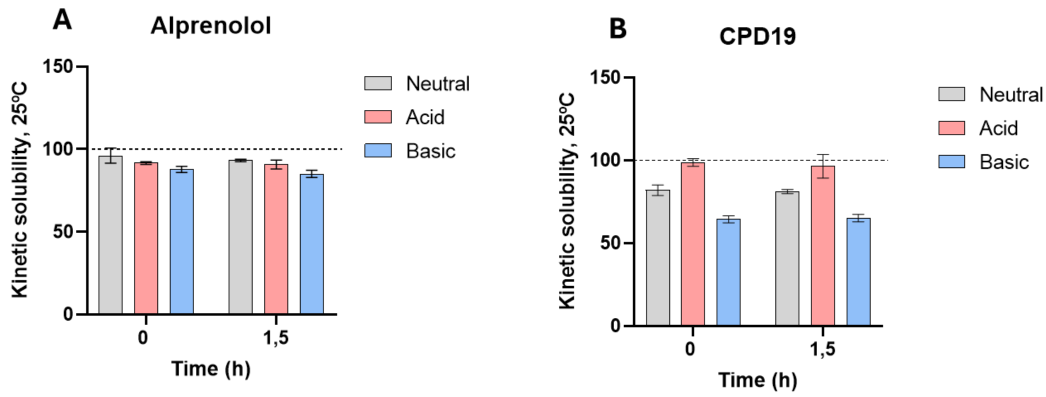

Figure S3. Kinetic solubility profiles of positive control alprenolol (**A**) compound **19** (**B**) determined after 0 and 1.5 h of incubation at 25 °C, across pH values of 1.7, 7.4, and 8.9. Data are presented as mean soluble fraction (%, ± SD).

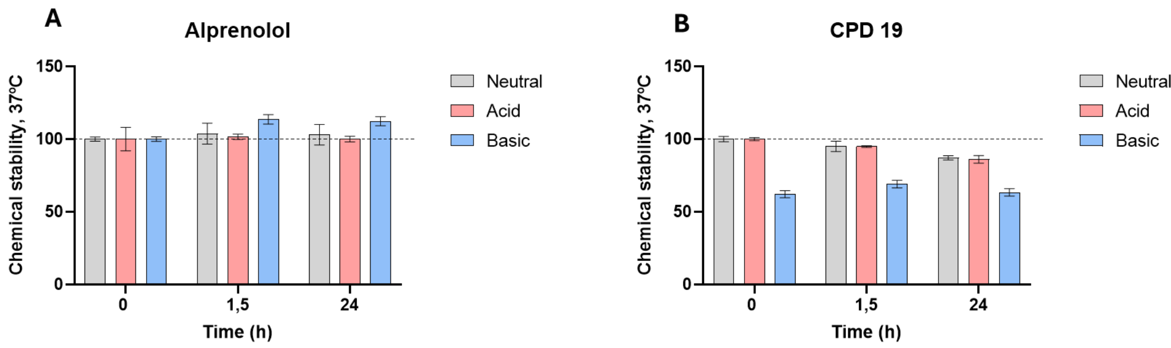

Figure S4. Chemical stability and solubility of positive control alprenolol (**A**) compound **19** (**B**) after 0, 1.5, and 24 h of incubation at 37 °C under acidic (pH 1.7), neutral (pH 7.4), and basic (pH 8.9) conditions. Results are shown as mean soluble fraction (%, ± SD).

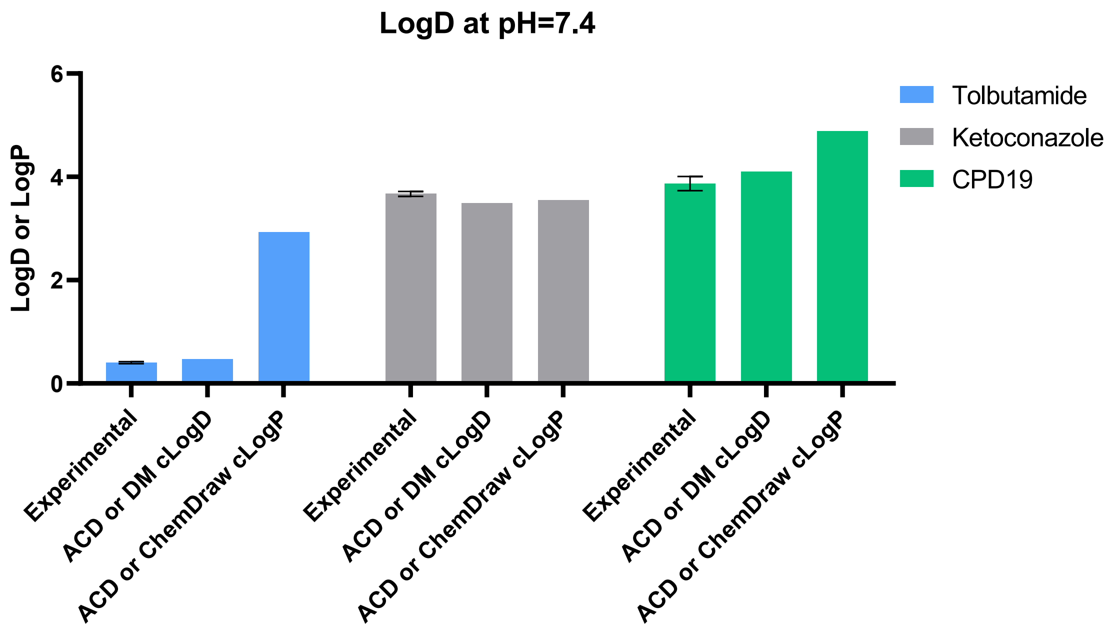

Figure S5. Experimental LogD values at pH 7.4 for **19** compared with reference compounds tolbutamide and ketoconazole. Calculated LogD and LogP values for all compounds are also included for comparison.

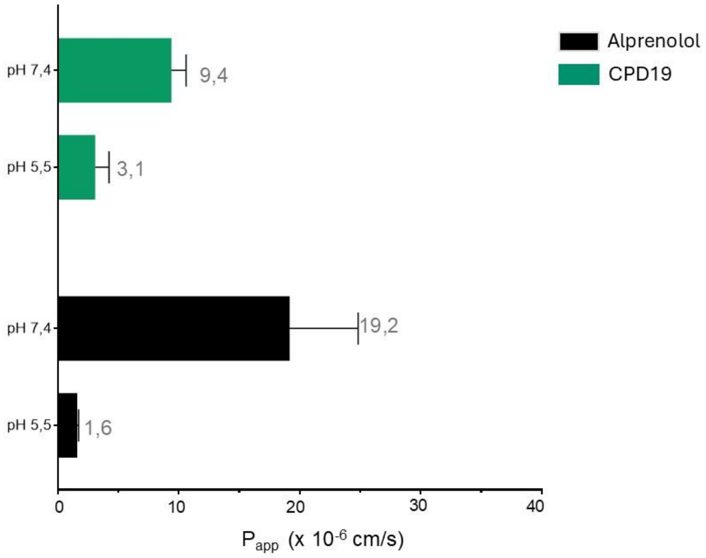

Figure S6. Apparent permeability coefficients (P_app, × 10^{-6} cm/s) of **19** (green) and alprenolol (black) determined in the PAMPA assay at pH 5.5 and 7.4. Each bar represents the mean ± standard deviation (n = 3).

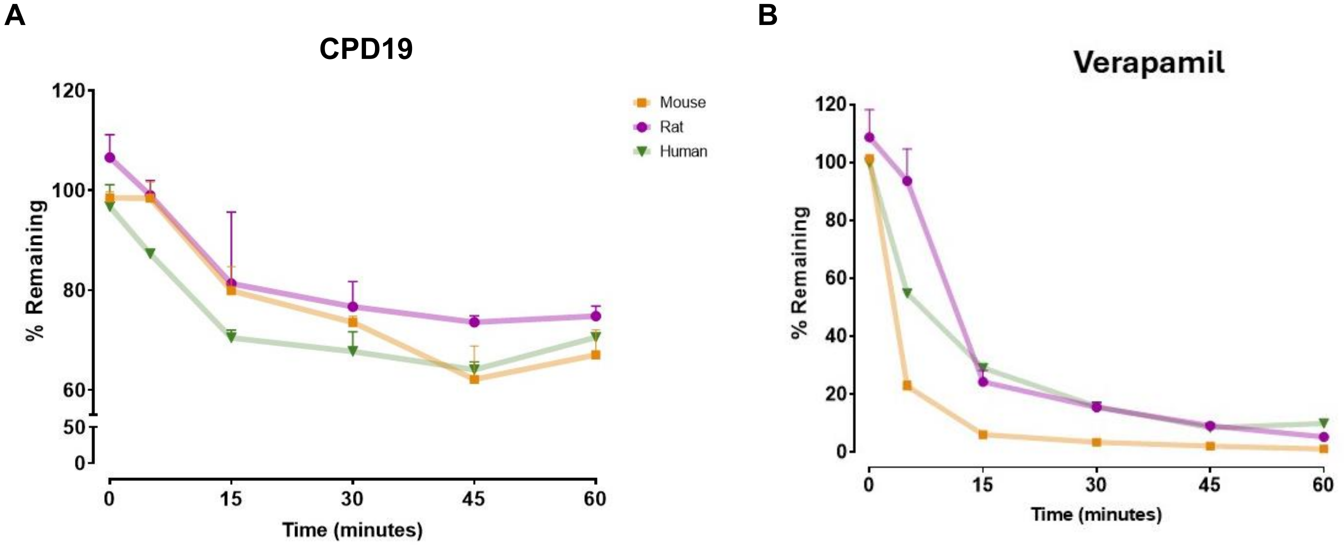

Figure S7: Microsomal stability profiles of **19** (**A**) and verapamil (**B**) in liver microsomes from mouse (yellow), rat (magenta), and human (green). The percentage of parent compound remaining over time was determined by LC-MS/MS. Compound **19** (**A**) shows slow degradation and high metabolic stability, while verapamil (**B**) demonstrates rapid metabolism, especially in mouse microsomes.

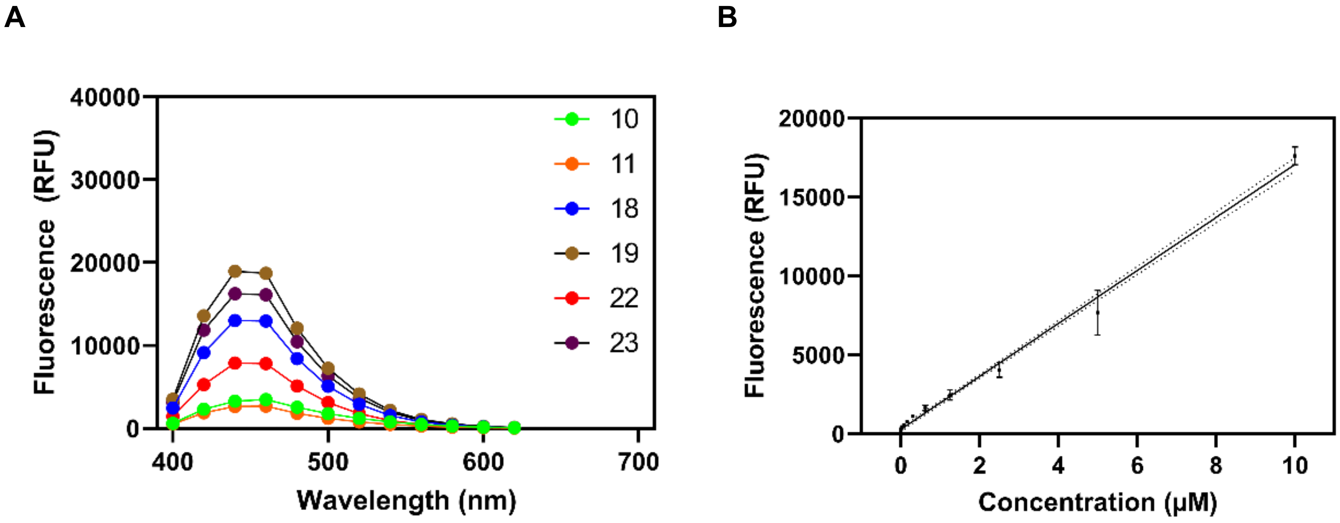

Figure S8. (A) Comparison of fluorescence emission profiles for marinoquinoline analogs **10**, **11**, **18**, **19**, **22**, and **23** excited at 400 nm, indicating that **19** exhibits the strongest emission among the tested compounds. (**B**) Sensitivity curve for **19**.

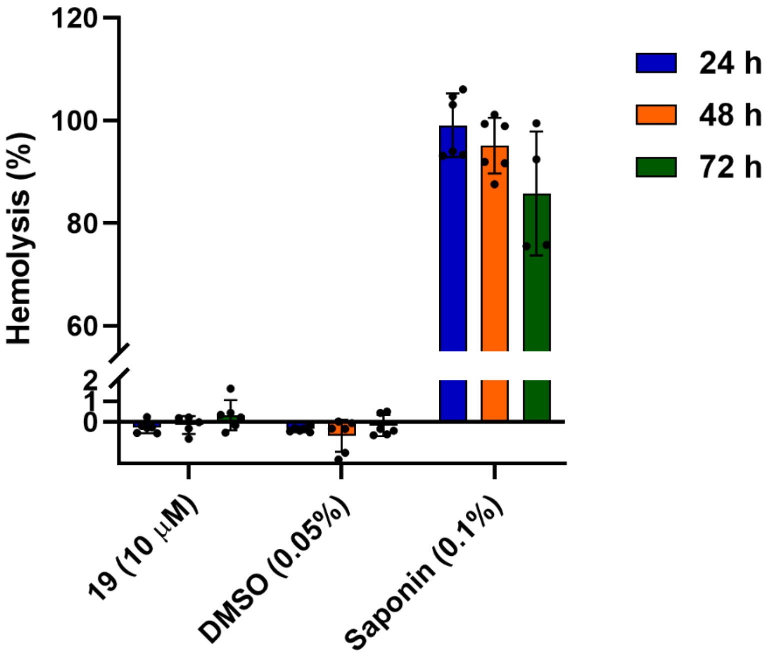

Figure S9. Hemolytic activity of **19** (10 μM) in fresh human red blood cells after 24, 48, and 72 h of incubation. Saponin (0.1%) and DMSO were used as positive and vehicle controls, respectively.

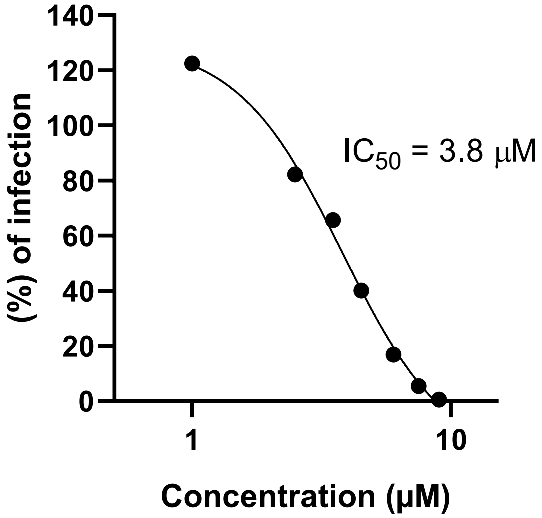

Figure S10. Liver-stage activity of compound **19** by *P. berghei* luciferase-expressing parasites.

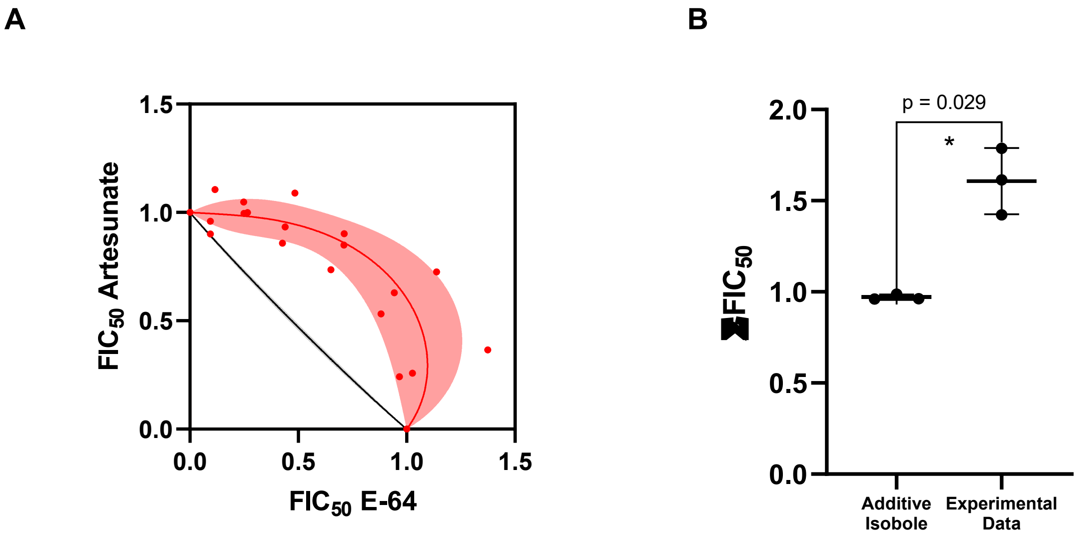

Figure S11. Evaluation of the combination of **19** with E-64. The black line and gray region depict the additivity curve, while the red region and red dots represent the experimental data. Column (A) Isobolograms for the combinations. Column (B) Statistical analysis of the combinations. These panels show the ∑FIC50 values derived from three independent experiments. A p-value < 0.05 indicates a statistically significant difference between the experimental data and the additivity isobole.

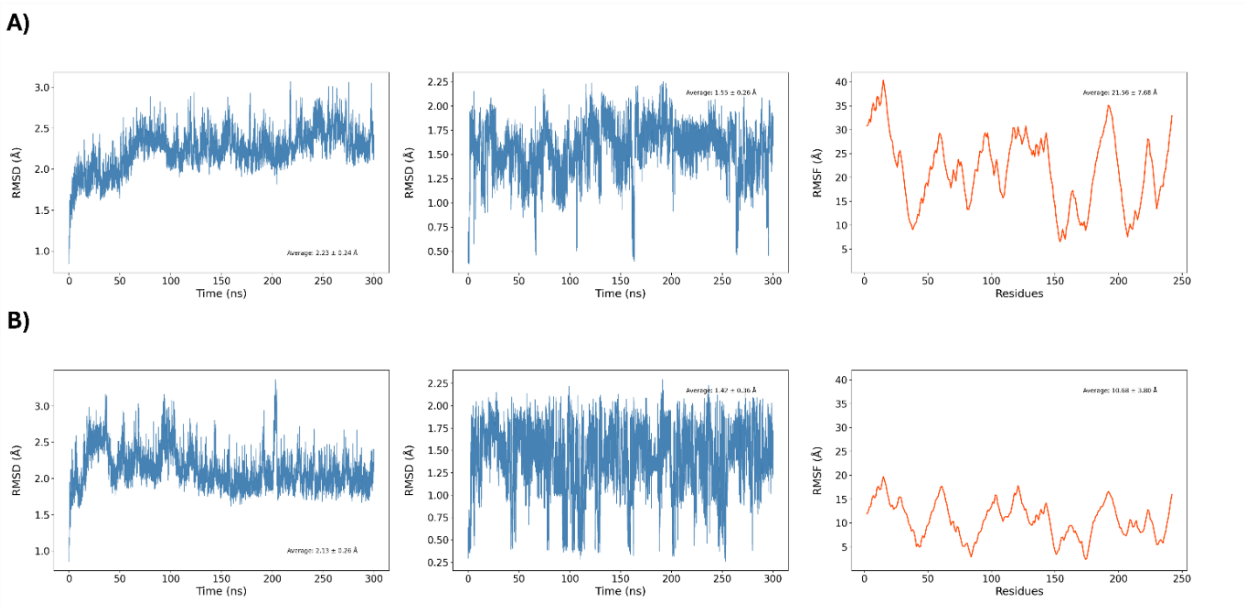

Figure S12. RMSD of the **19**-FP2a complex, **19** and RMSF of the protein over 300 ns of simulation time, respectively. **A)** For the top-ranked pose by docking. **B)** For the S2 binding mode pose.

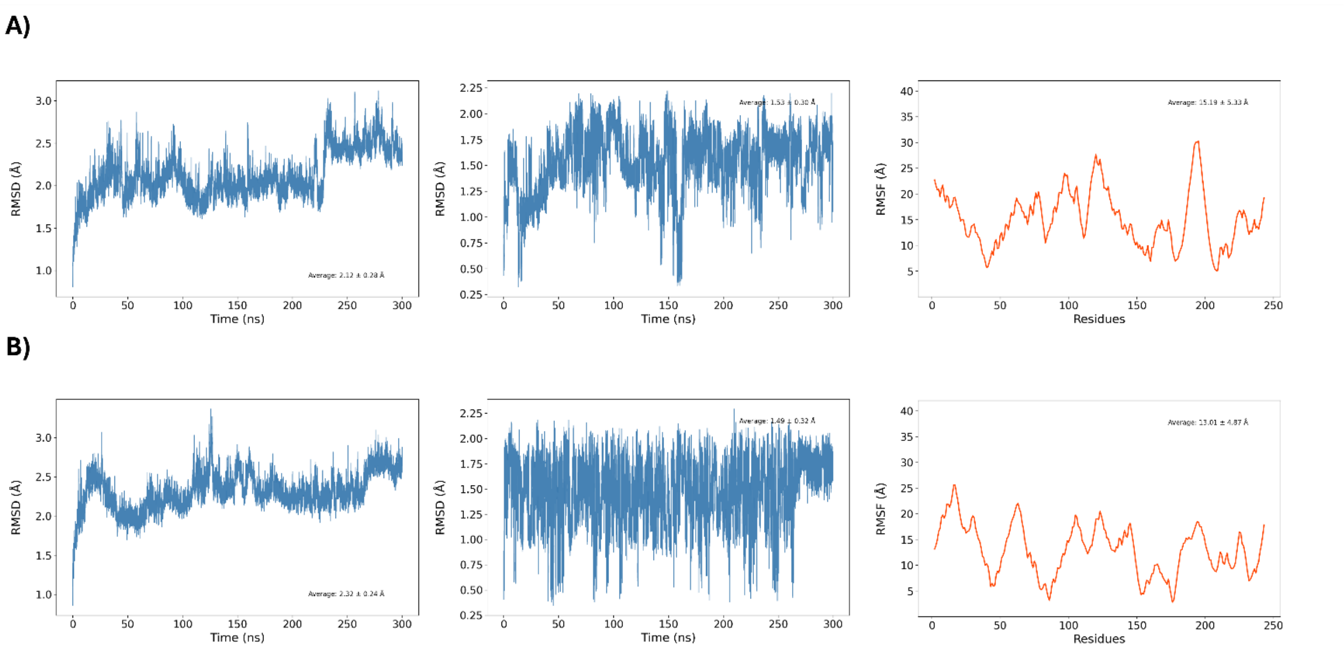

Figure S13. RMSD of the **19**-FP3 complex, **19** and RMSF of the protein over 300 ns of simulation time, respectively. **A)** For the top-ranked pose by docking. **B)** For the S2 binding mode pose.

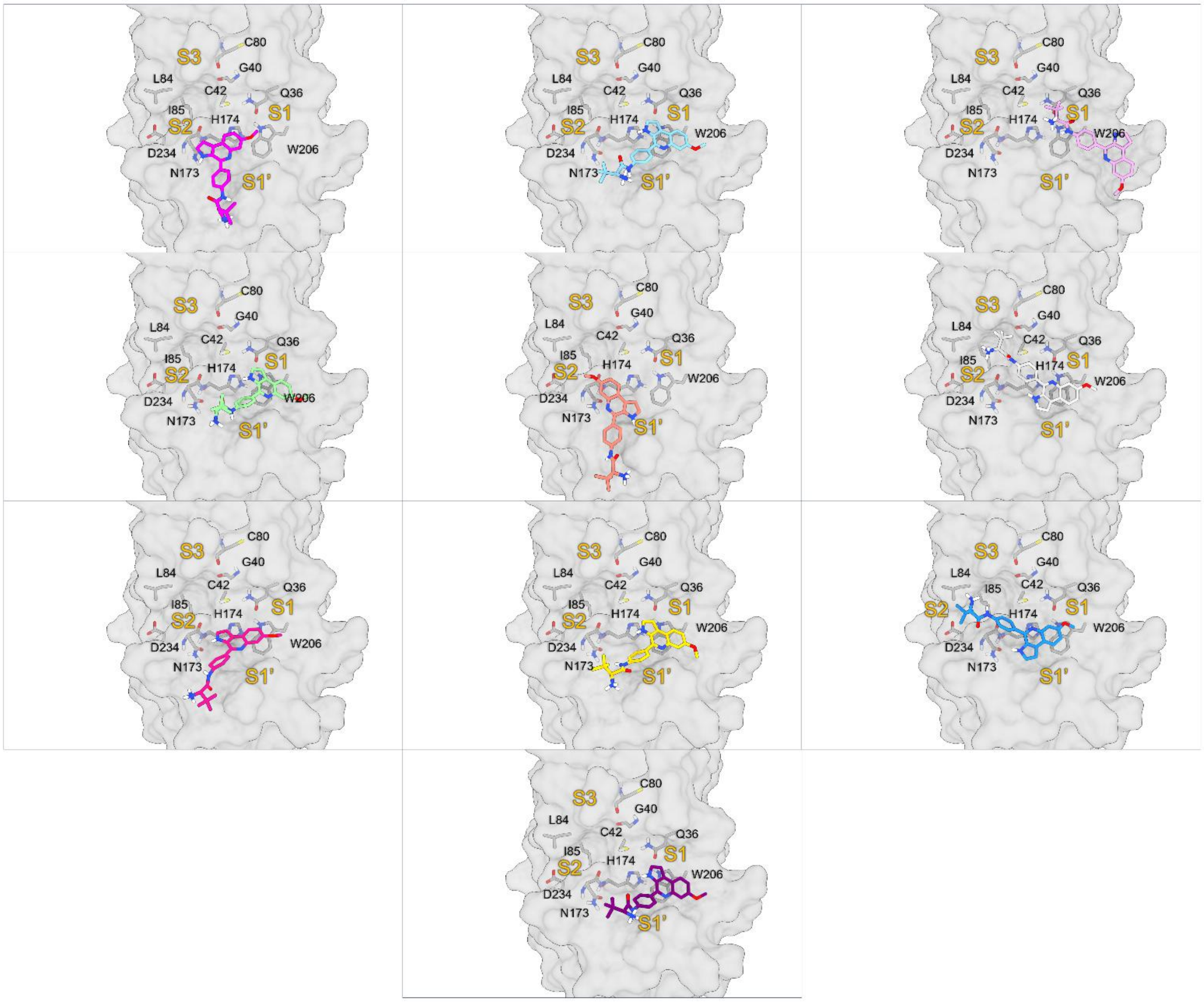

Figure S14. Representative structures from clusters 1–10, derived from the 300 ns MD simulation of the top-ranked docking pose against FP2a. The distinct conformations highlight the structural variability observed throughout the simulation, emphasizing the challenge in identifying a definitive binding pose.

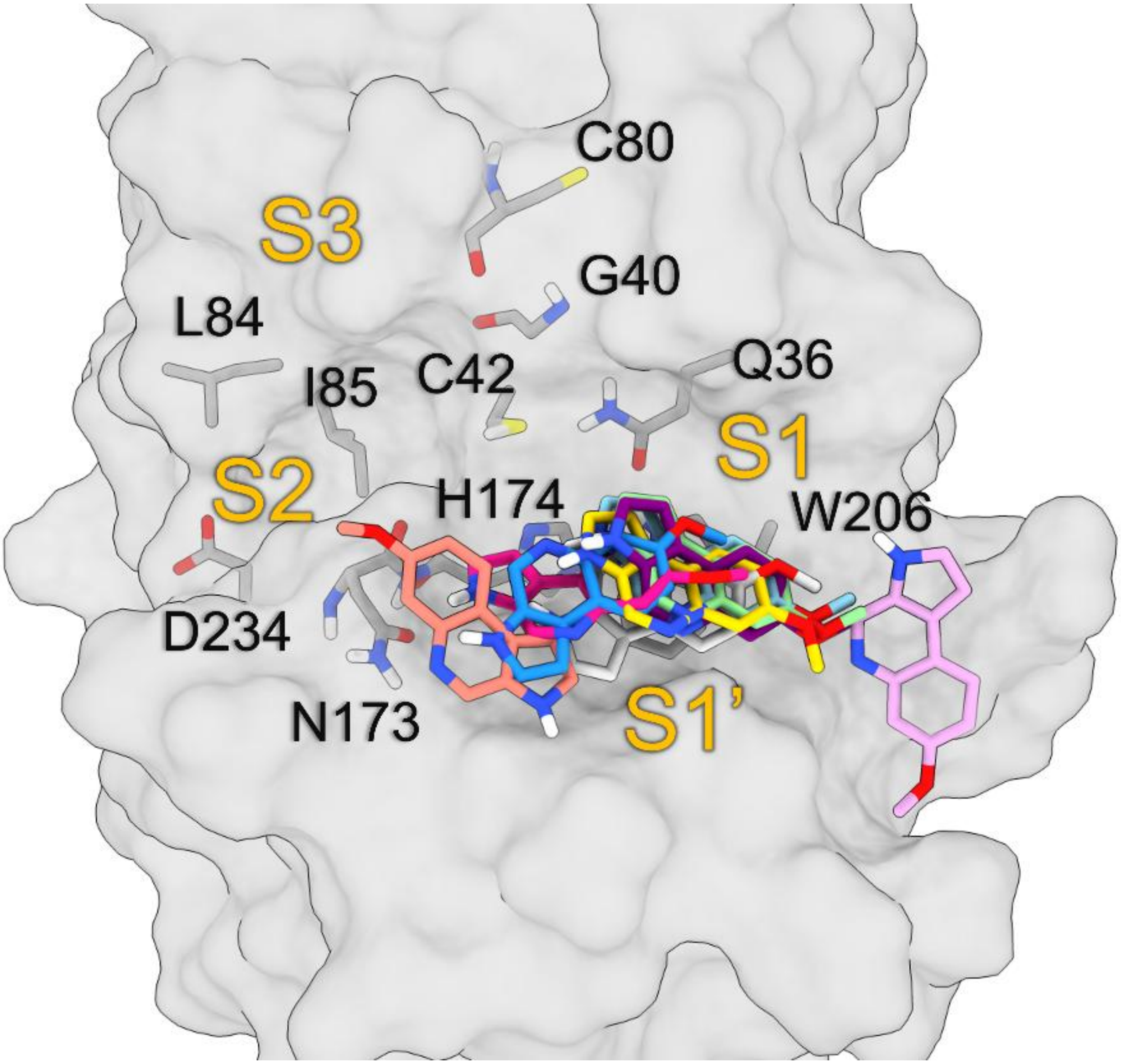

Figure S15. Alignment of the scaffold for **19** representative structures from clusters 1–10, derived from the 300 ns MD simulation of the top-ranked docking pose against FP2a.

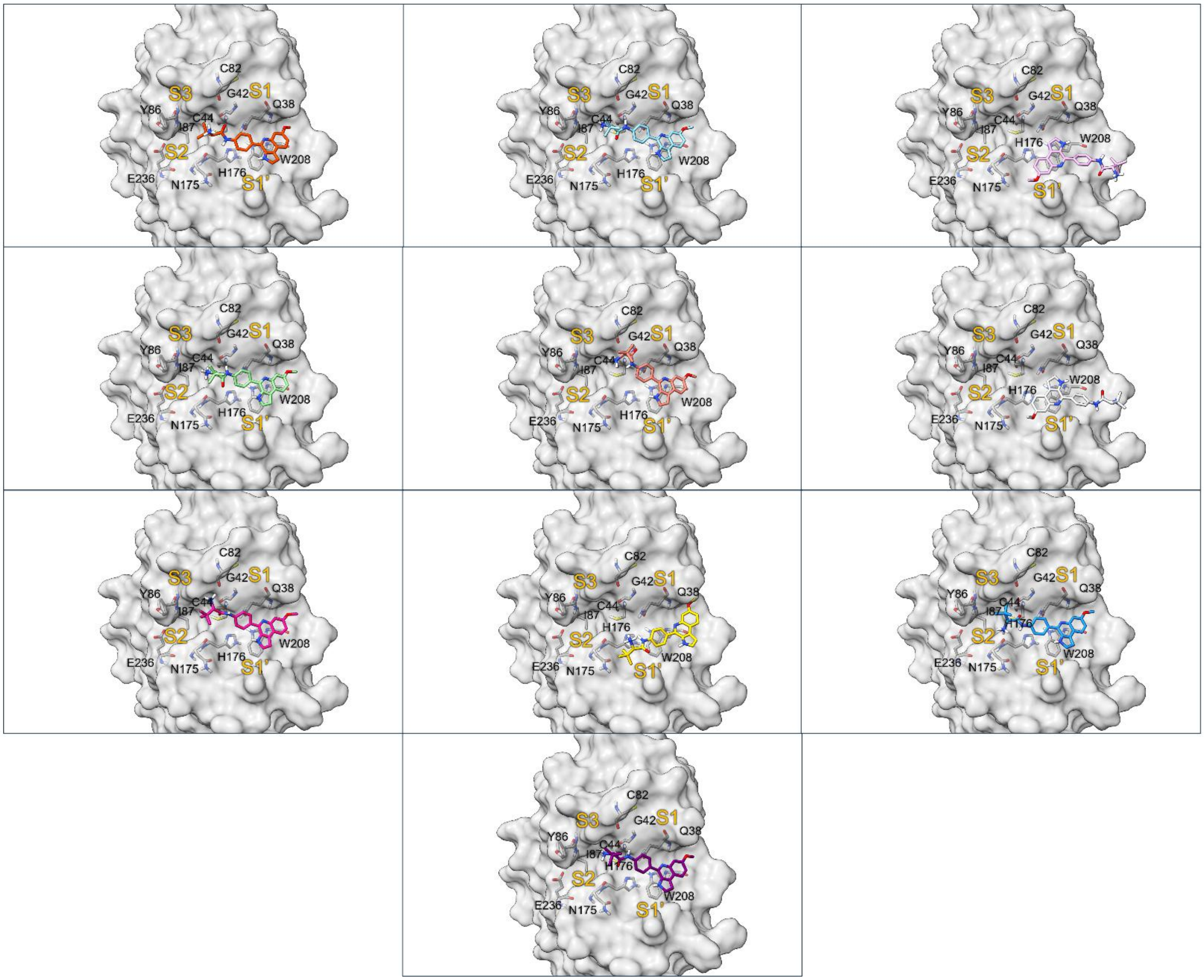

Figure S16. Representative structures from clusters 1–10, derived from the 300 ns MD simulation of the top-ranked docking pose against FP3. The distinct conformations highlight the structural variability observed throughout the simulation, emphasizing the challenge in identifying a definitive binding pose.

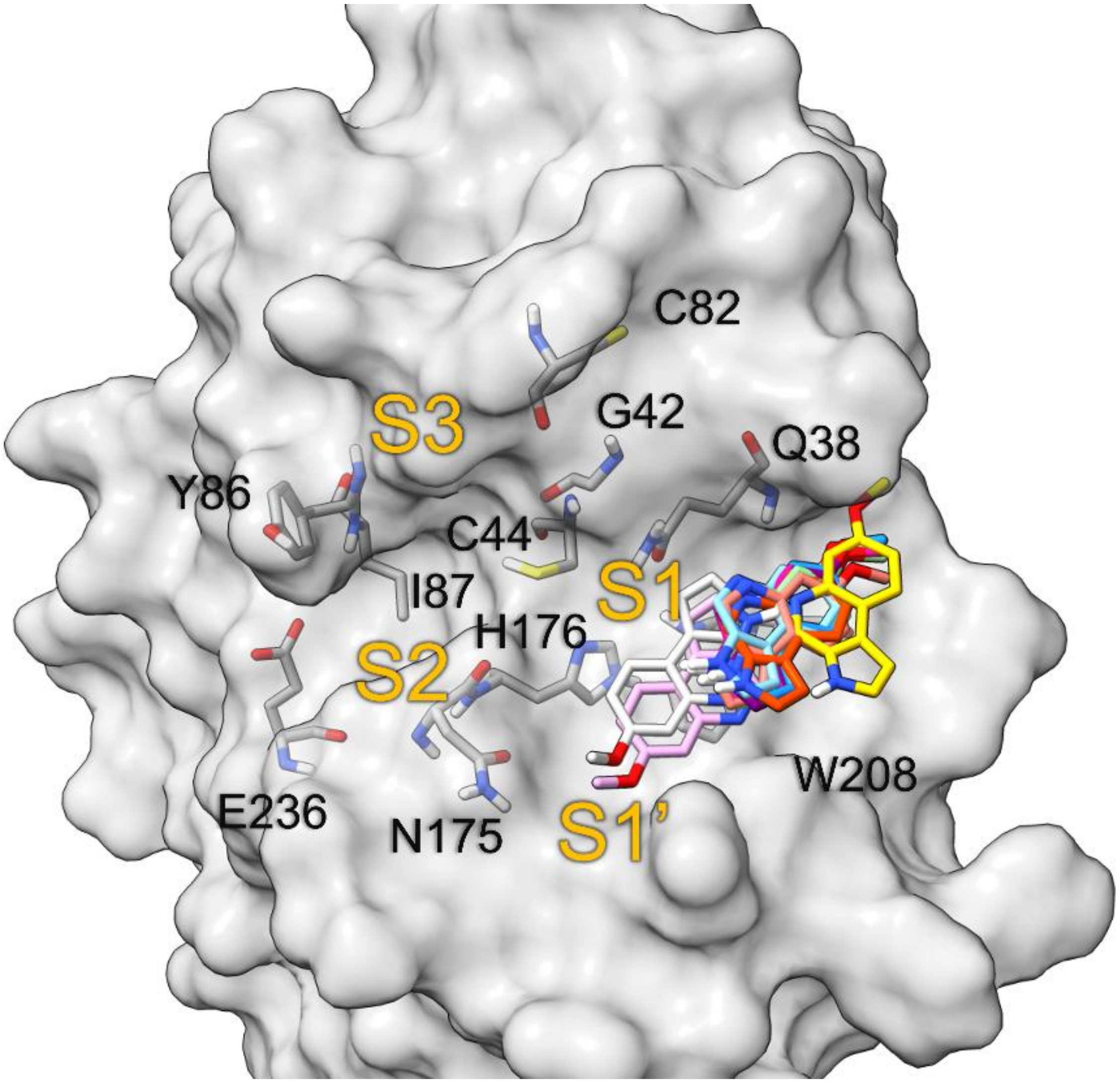

Figure S17. Alignment of the scaffold for **19** representative structures from clusters 1–10, derived from the 300 ns MD simulation of the top-ranked docking pose against FP3.

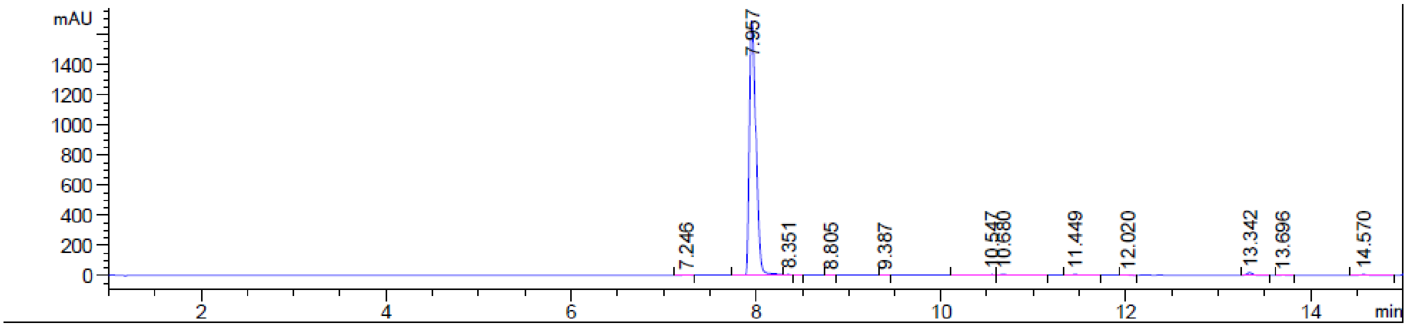

Peak #	RetTime [min]	Area [mAu*s]	Area %
1	7.246	5.49238	0.0645
**2**	**7.957**	**8261.27832**	**97.0167**
3	8.351	5.47411	0.0643
4	8.805	8.02012	0.0942
5	9.387	5.48956	0.0645
6	10.547	49.02007	0.5757
7	10.680	41.70085	0.4897
8	11.449	23.12832	0.2716
9	12.020	7.12441	0.0837
10	13.342	69.52850	0.8165
11	13.696	7.20608	0.0846
12	14.570	31.85272	0.3741

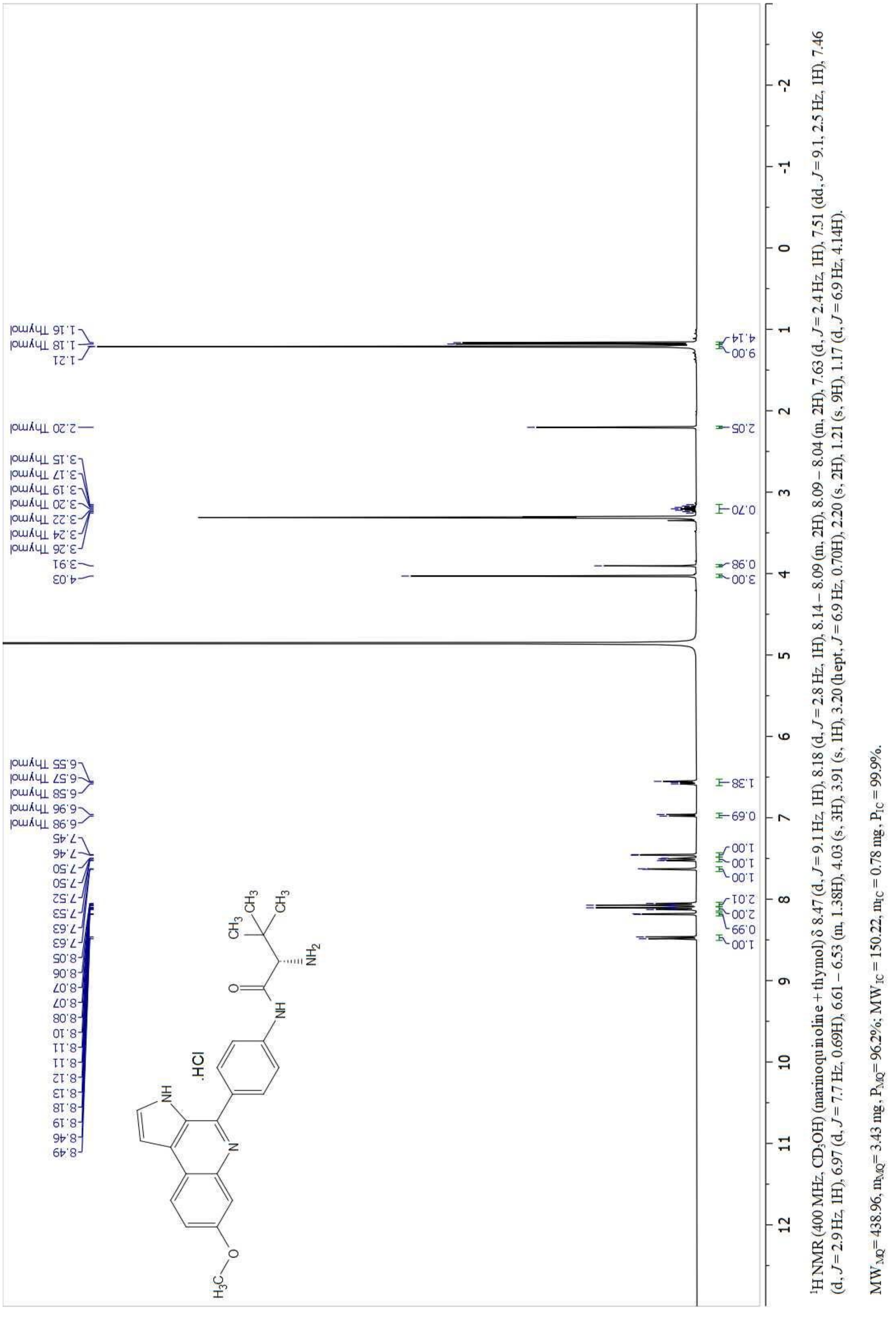

Figure S18. HPLC purity and quantitative ^{1}H NMR of compound **19** employed in biochemical and *in vivo* assays**.**

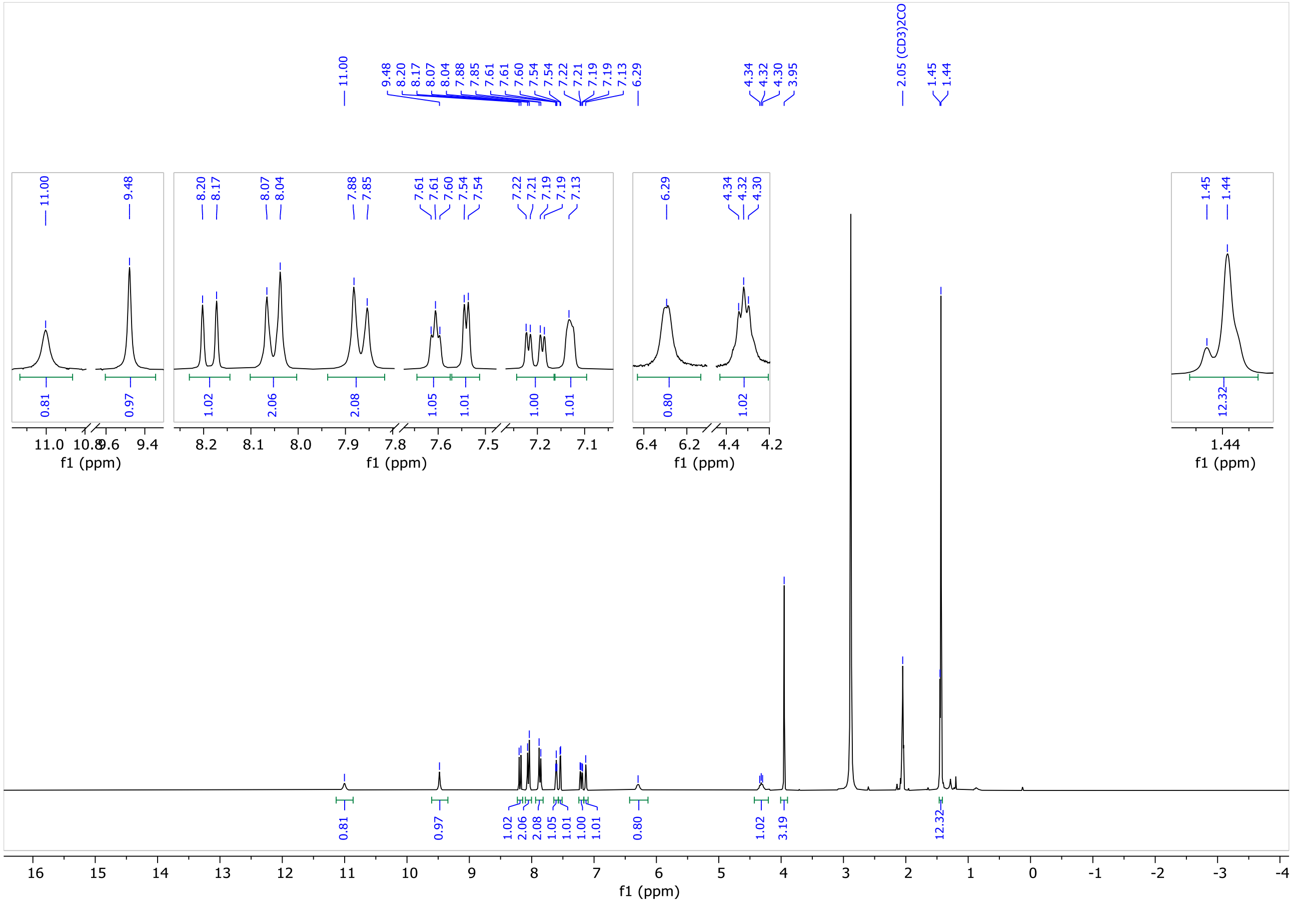

Figure S19. NMR Spectrum of ^{1}H of **6** (300 MHz, Acetone-d_6).

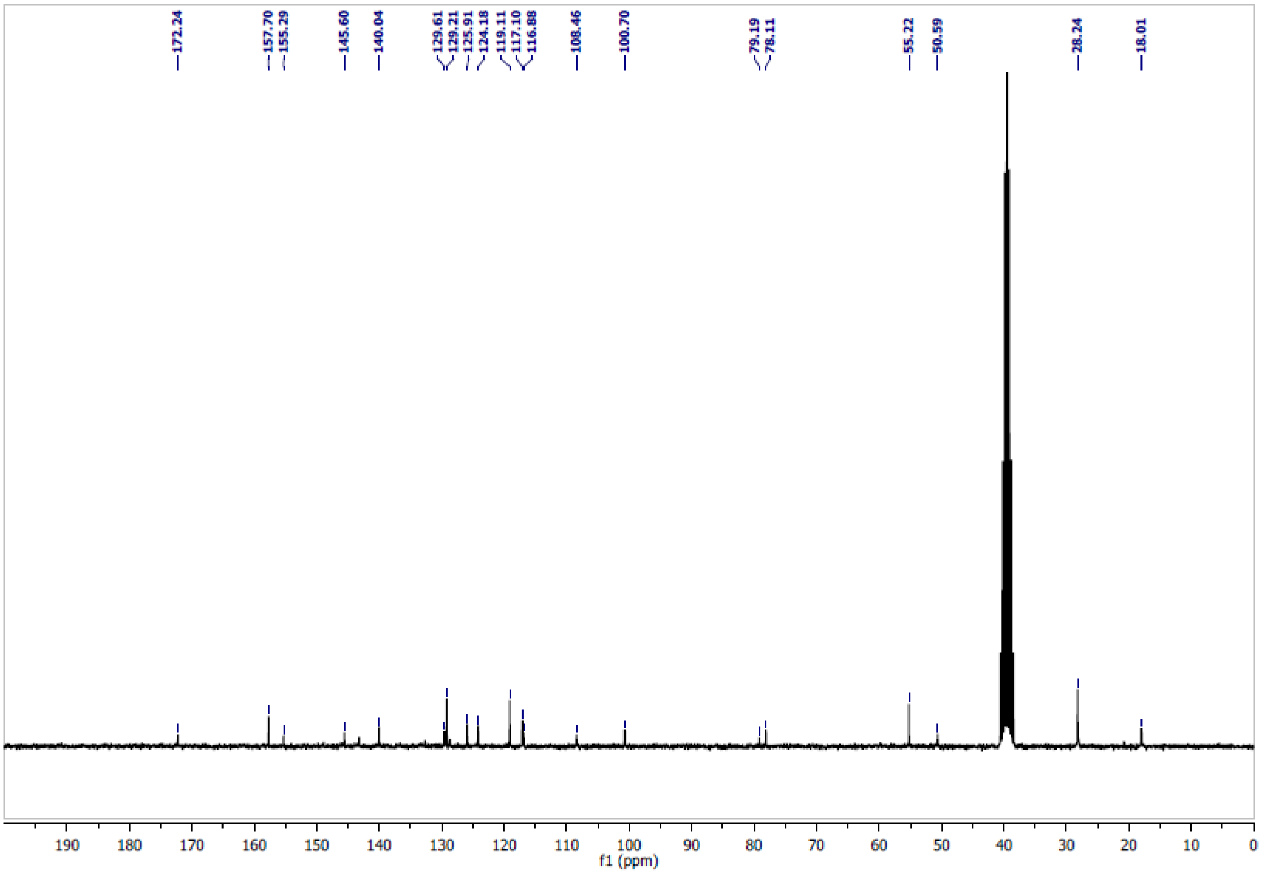

Figure S20. NMR Spectrum of ^{13}C of **6** (63 MHz, DMSO-d6).

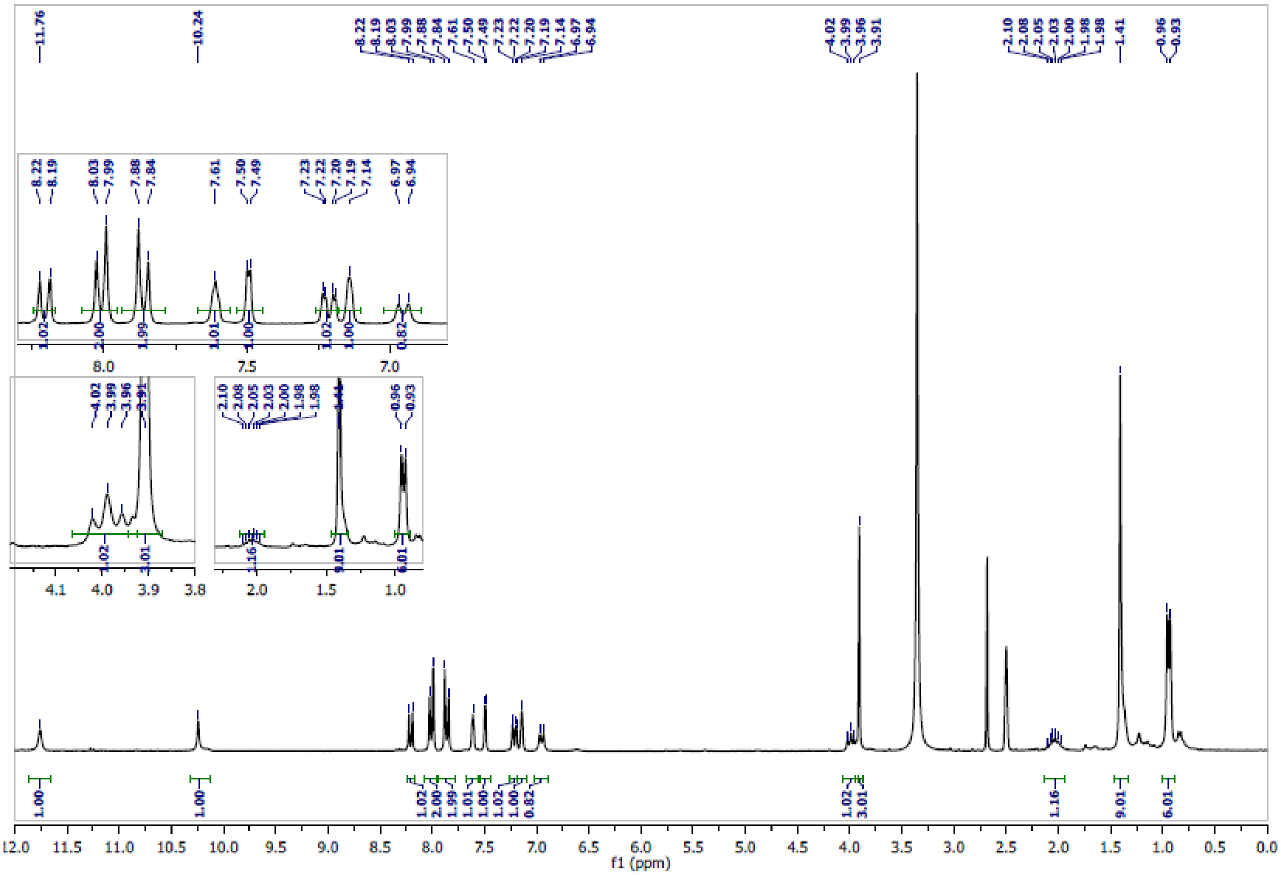

Figure S21. NMR Spectrum of ^{1}H of **7** (250 MHz, DMSO-d6).

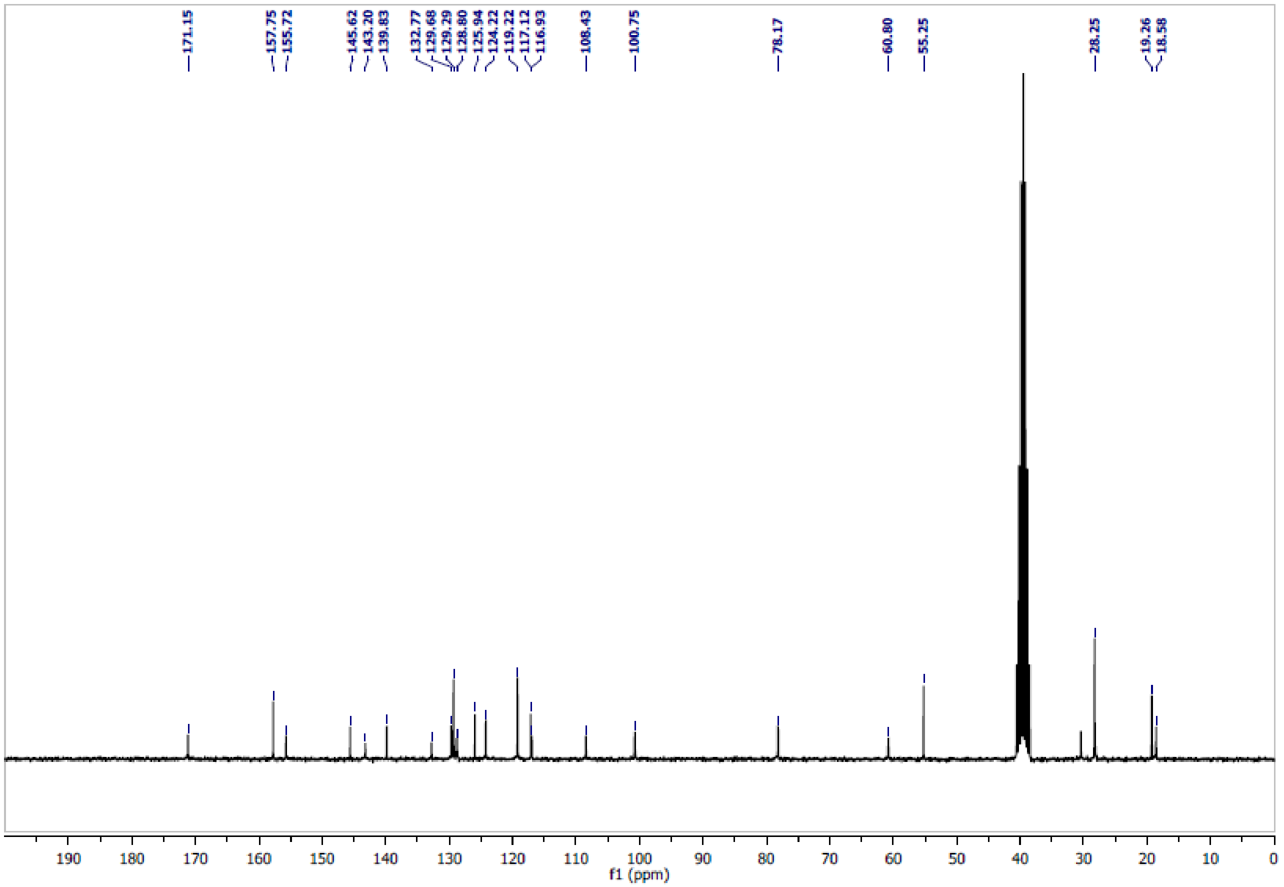

Figure S22. NMR Spectrum of ^{13}C of **7** (63 MHz, DMSO-d6).

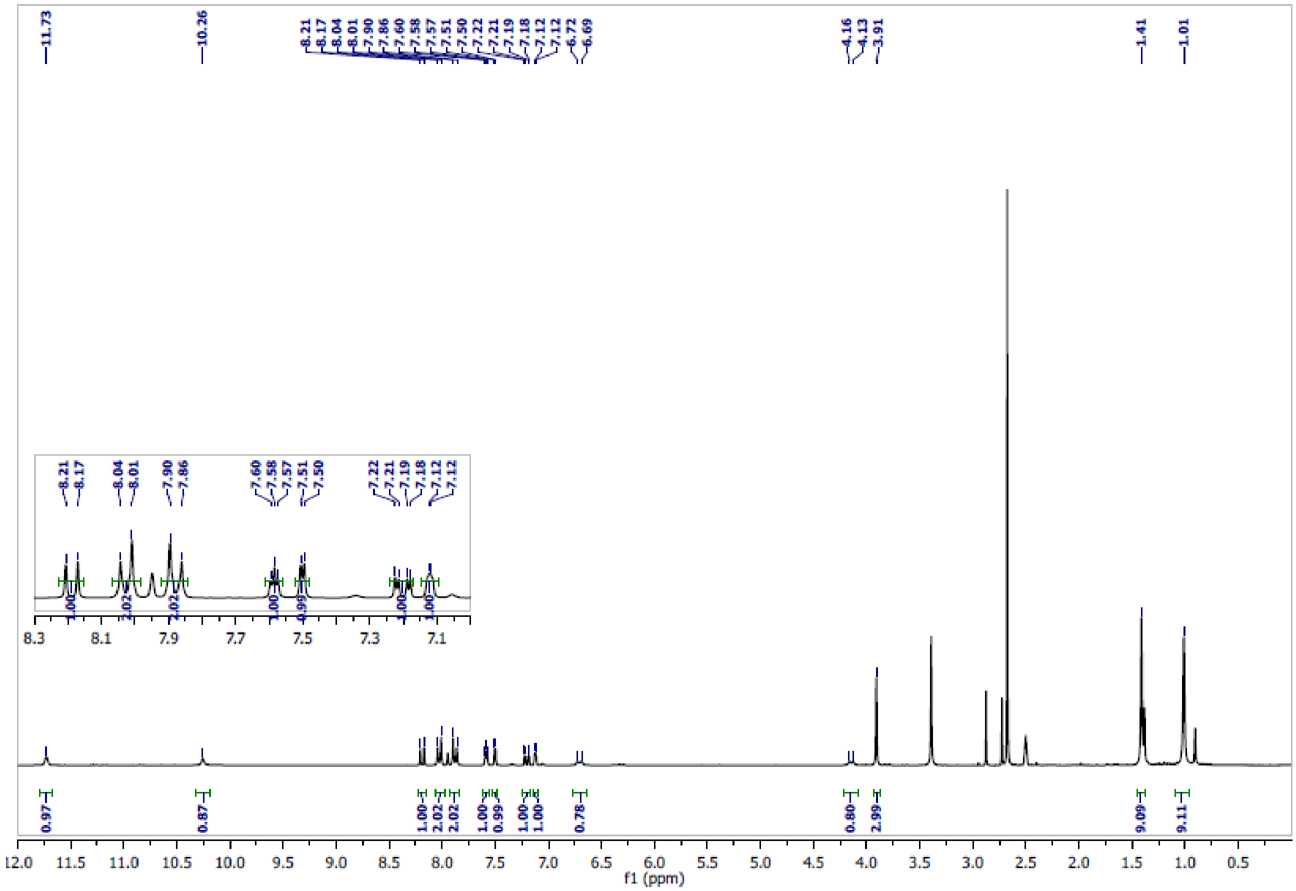

Figure S23. NMR Spectrum of ^{1}H of **8** (250 MHz, DMSO-d6).

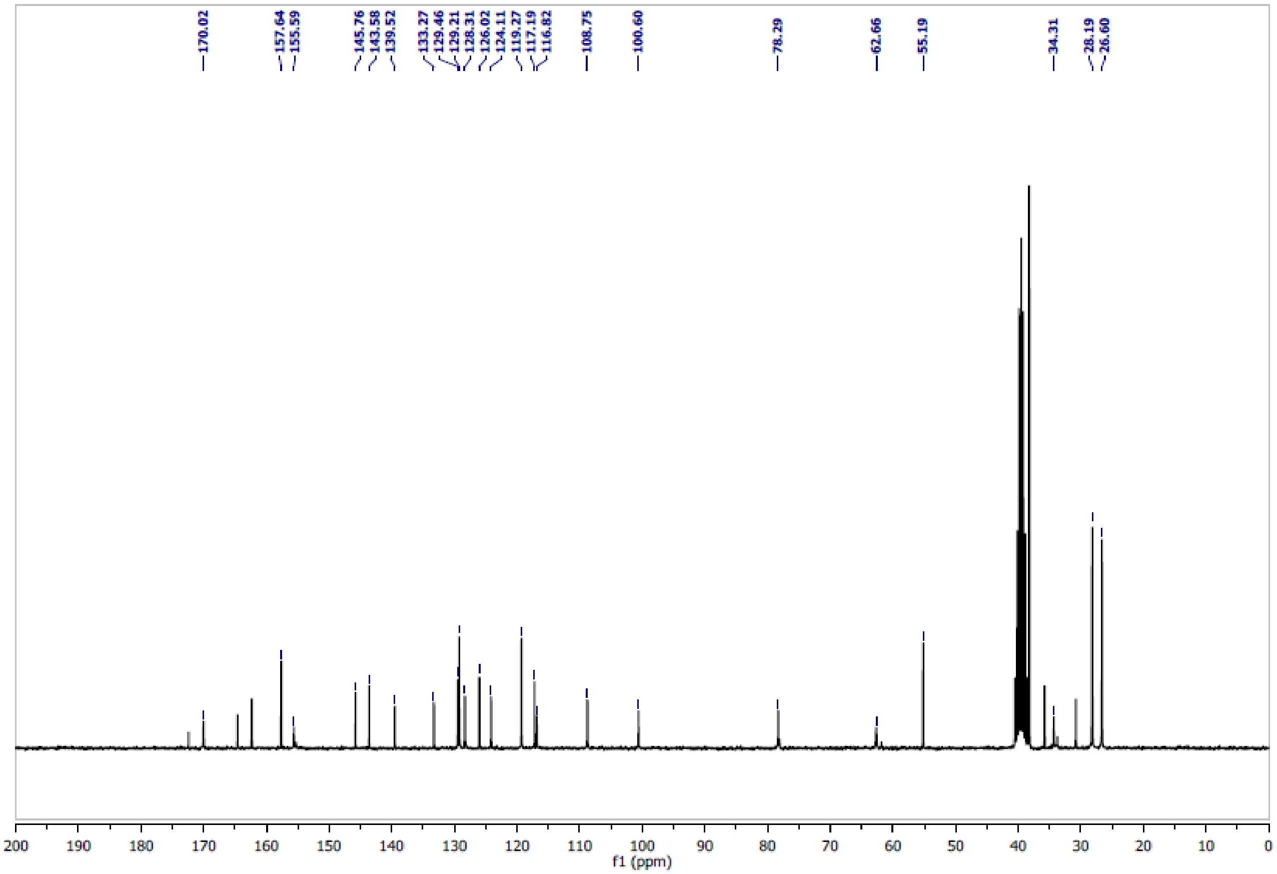

Figure S24. NMR Spectrum of ^{13}C of **8** (63 MHz, DMSO-d6).

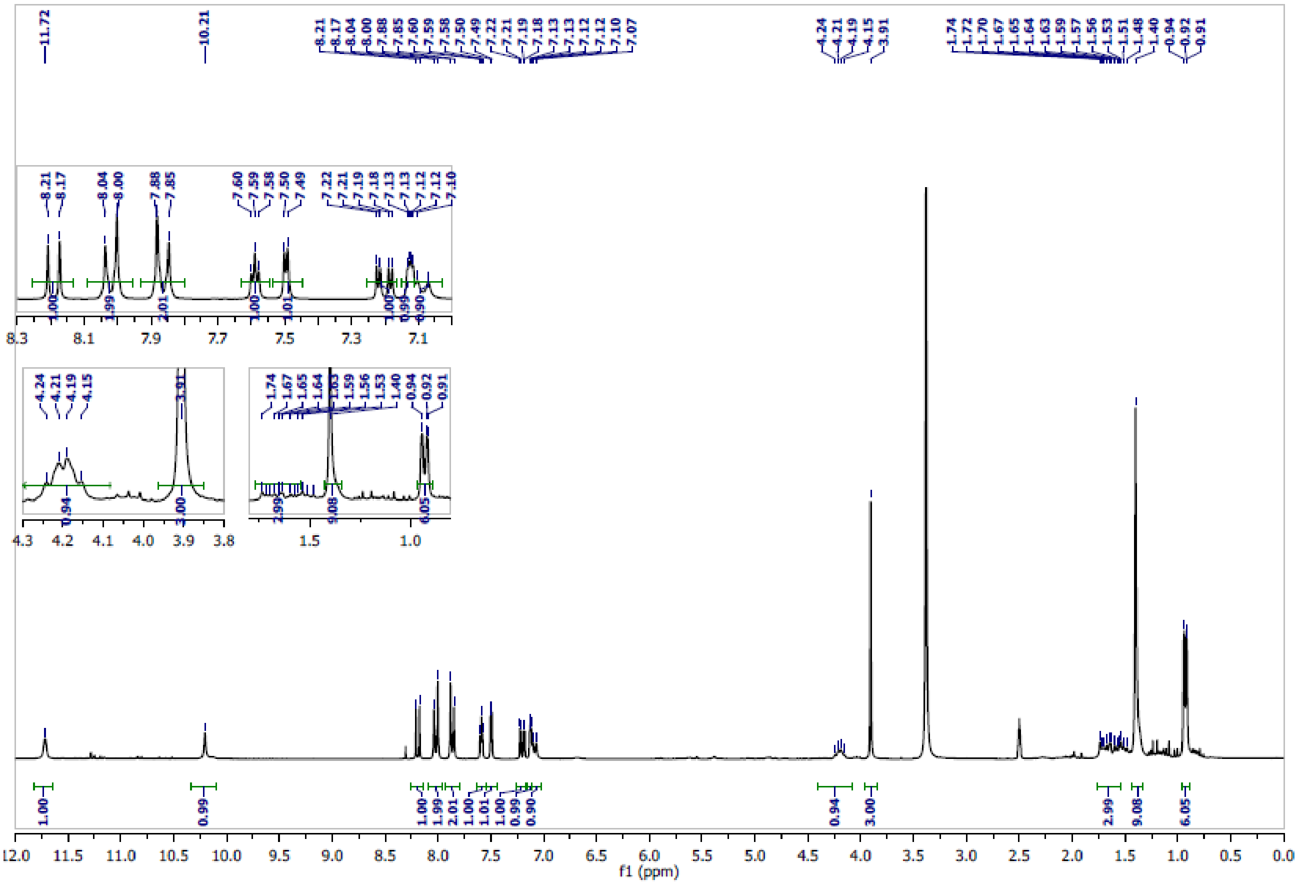

Figure S25. NMR Spectrum of ^{1}H of **9** (250 MHz, DMSO-d6).

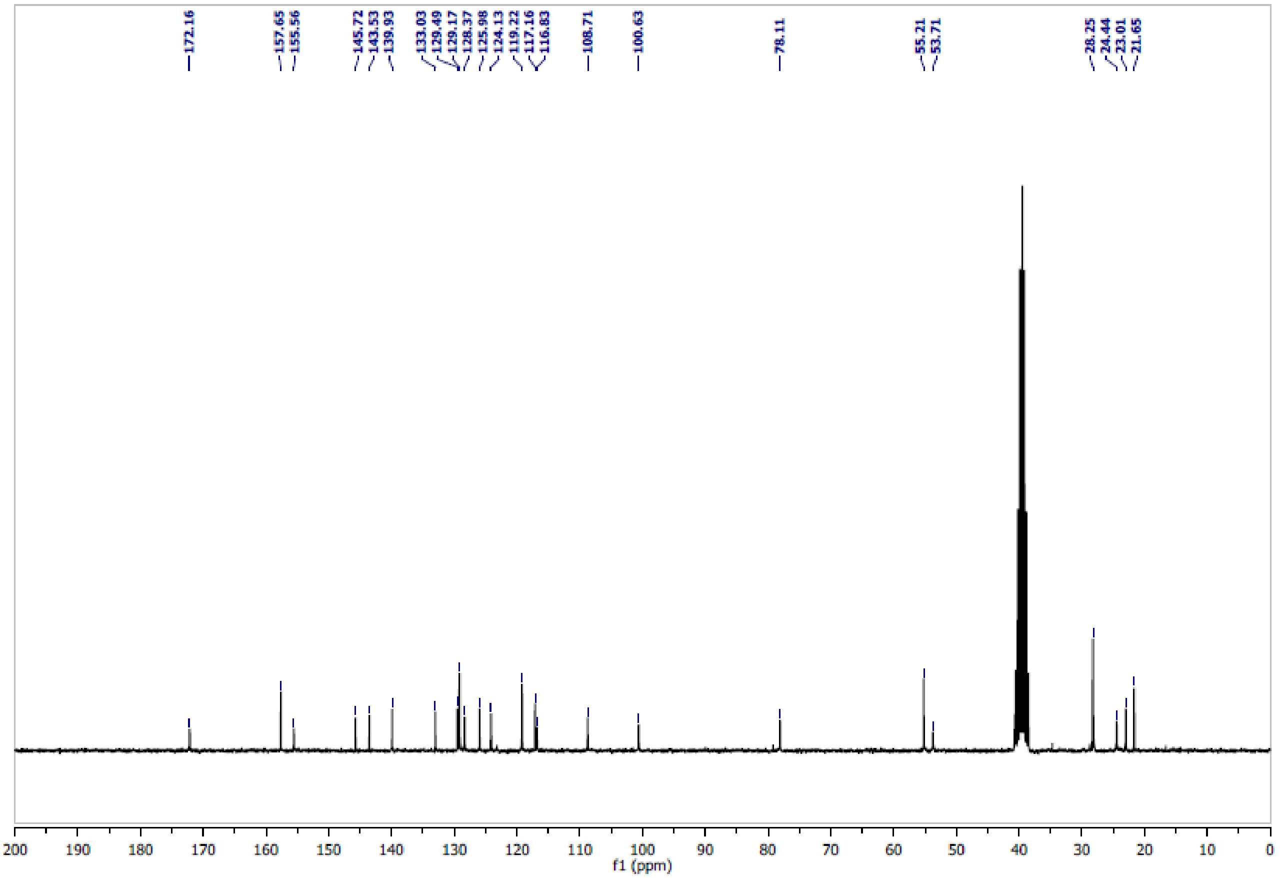

Figure S26. NMR Spectrum of ^{13}C of **9** (63 MHz, DMSO-d6).

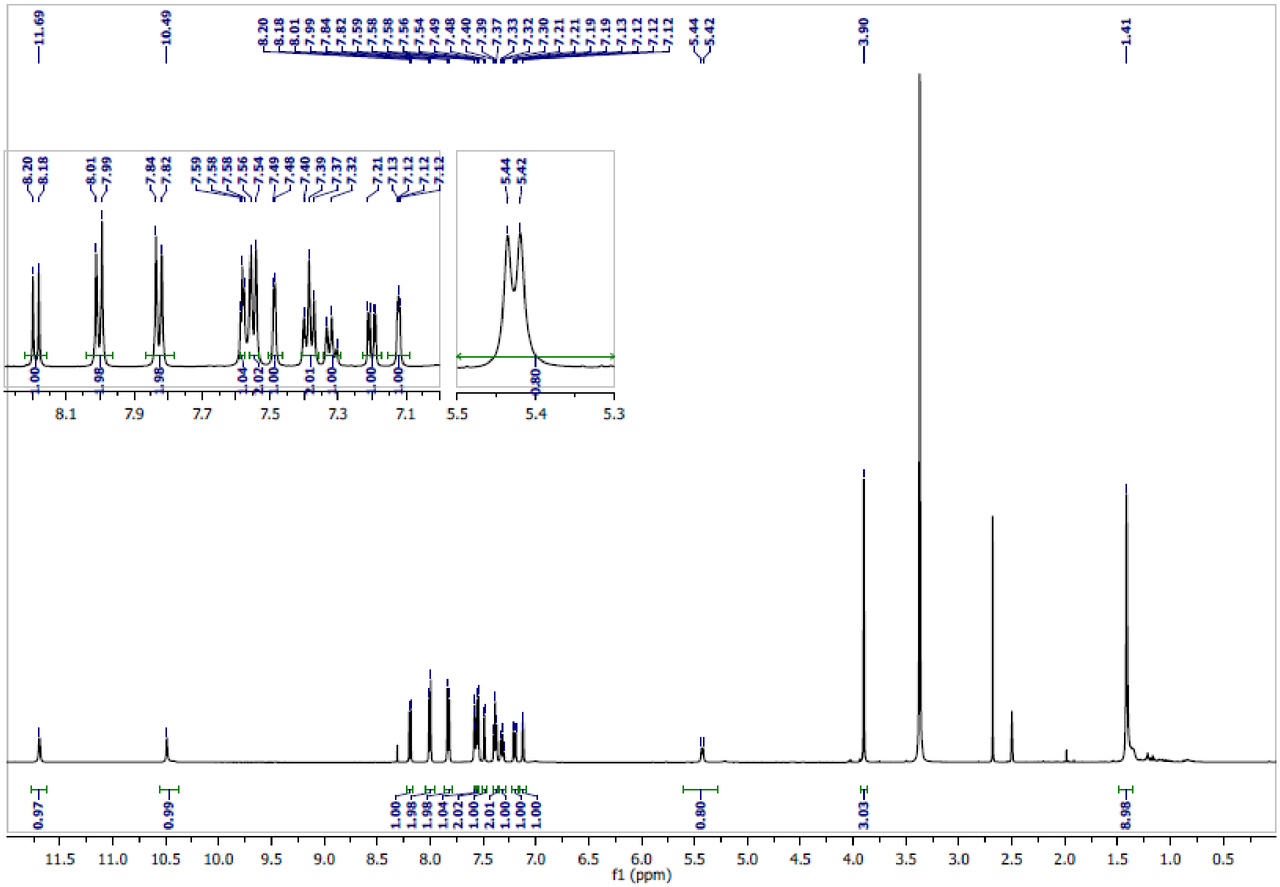

Figure S27. NMR Spectrum of ^{1}H of **10** (500 MHz, DMSO-d6).

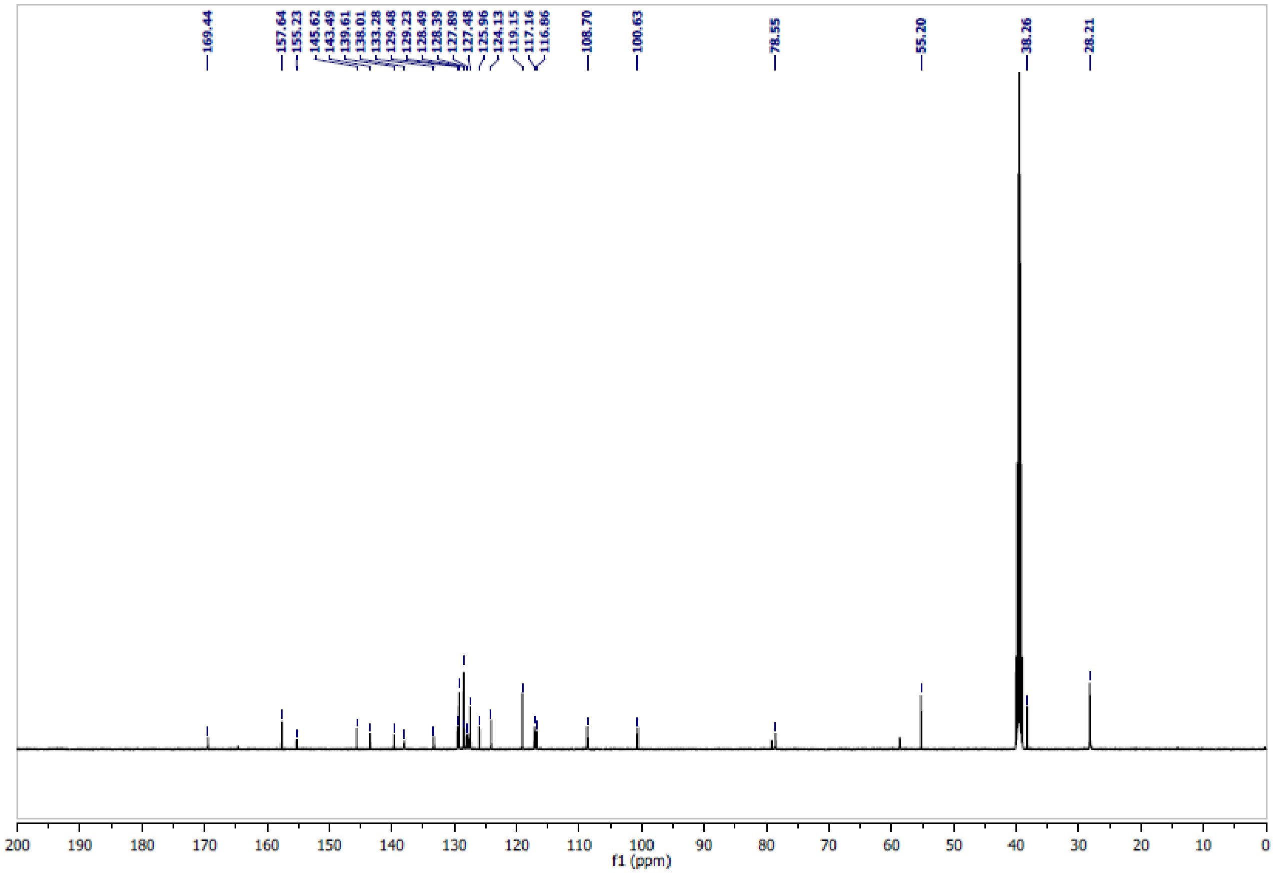

Figure S28. NMR Spectrum of ^{13}C of **10** (126 MHz, DMSO-d6).

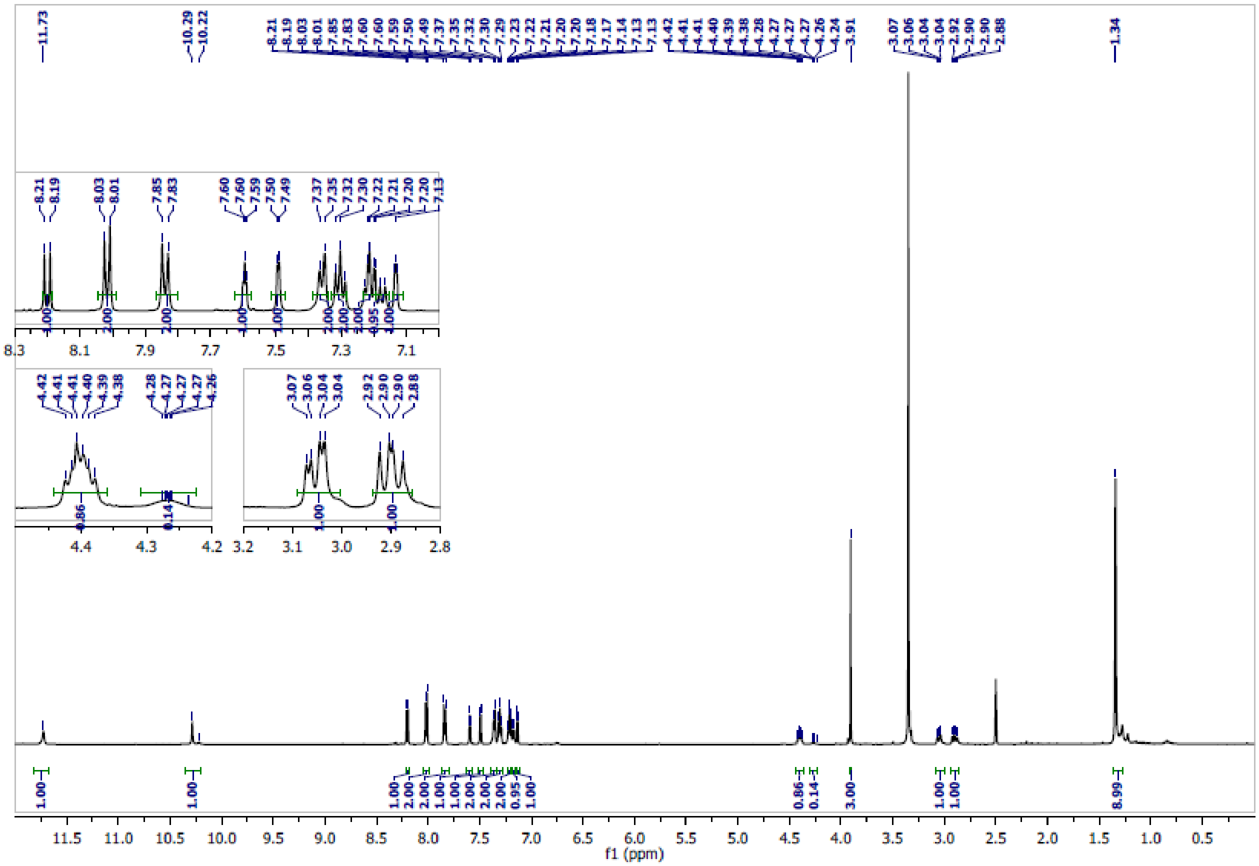

Figure S29. NMR Spectrum of ^{1}H of **11** (500 MHz, DMSO-d6).

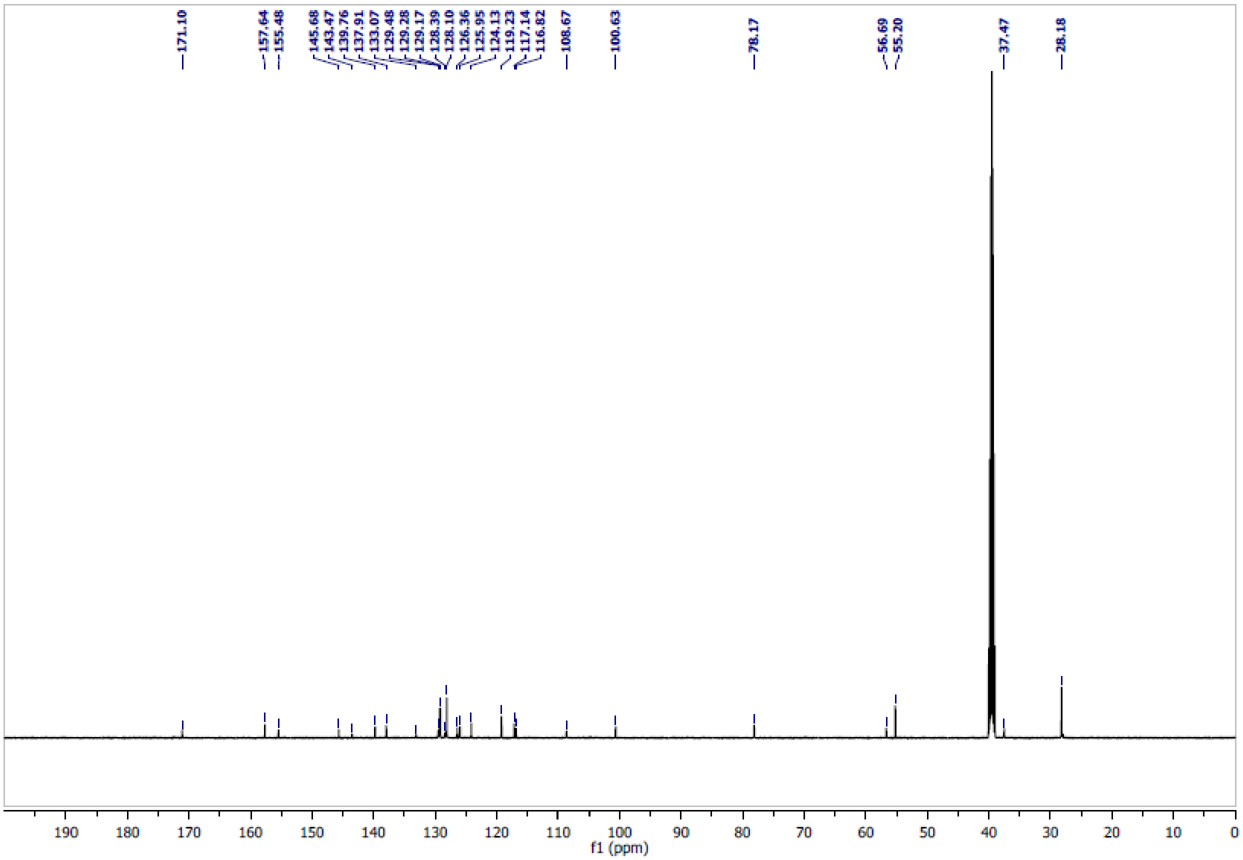

Figure S30. NMR Spectrum of ^{13}C of **11** (126 MHz, DMSO-d6).

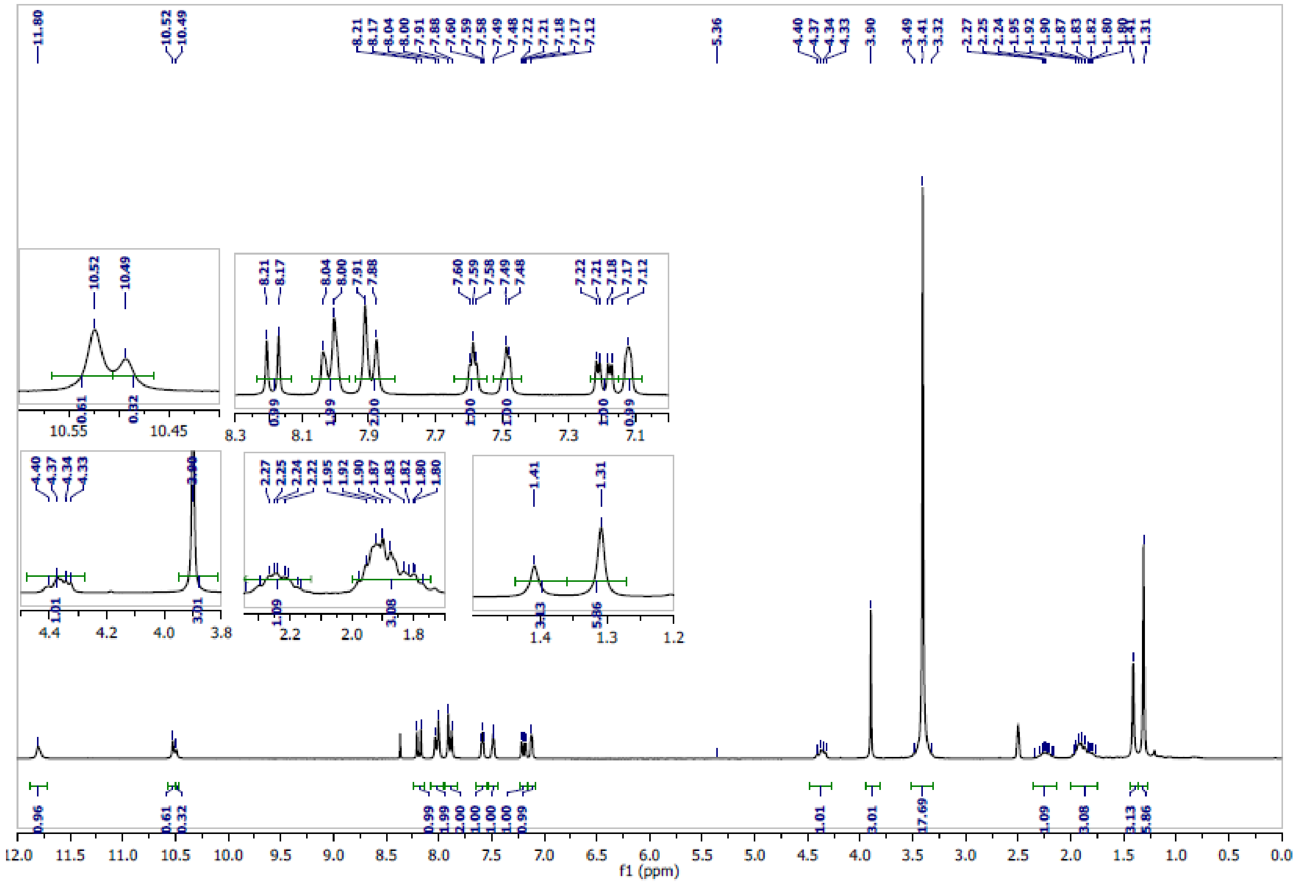

Figure S31. NMR Spectrum of ^{1}H of **12** (250 MHz, DMSO-d6).

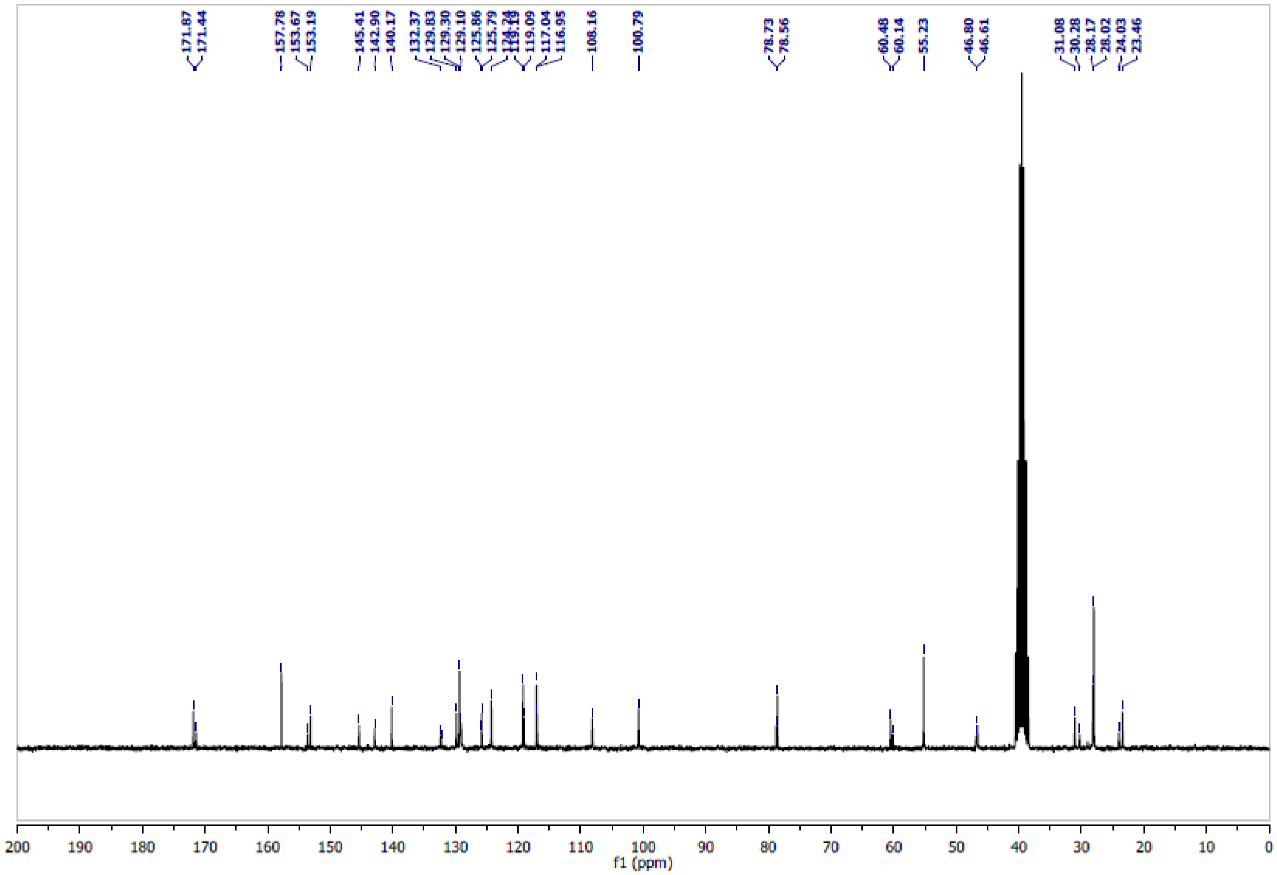

Figure S32. NMR Spectrum of ^{13}C of **12** (63 MHz, DMSO-d6).

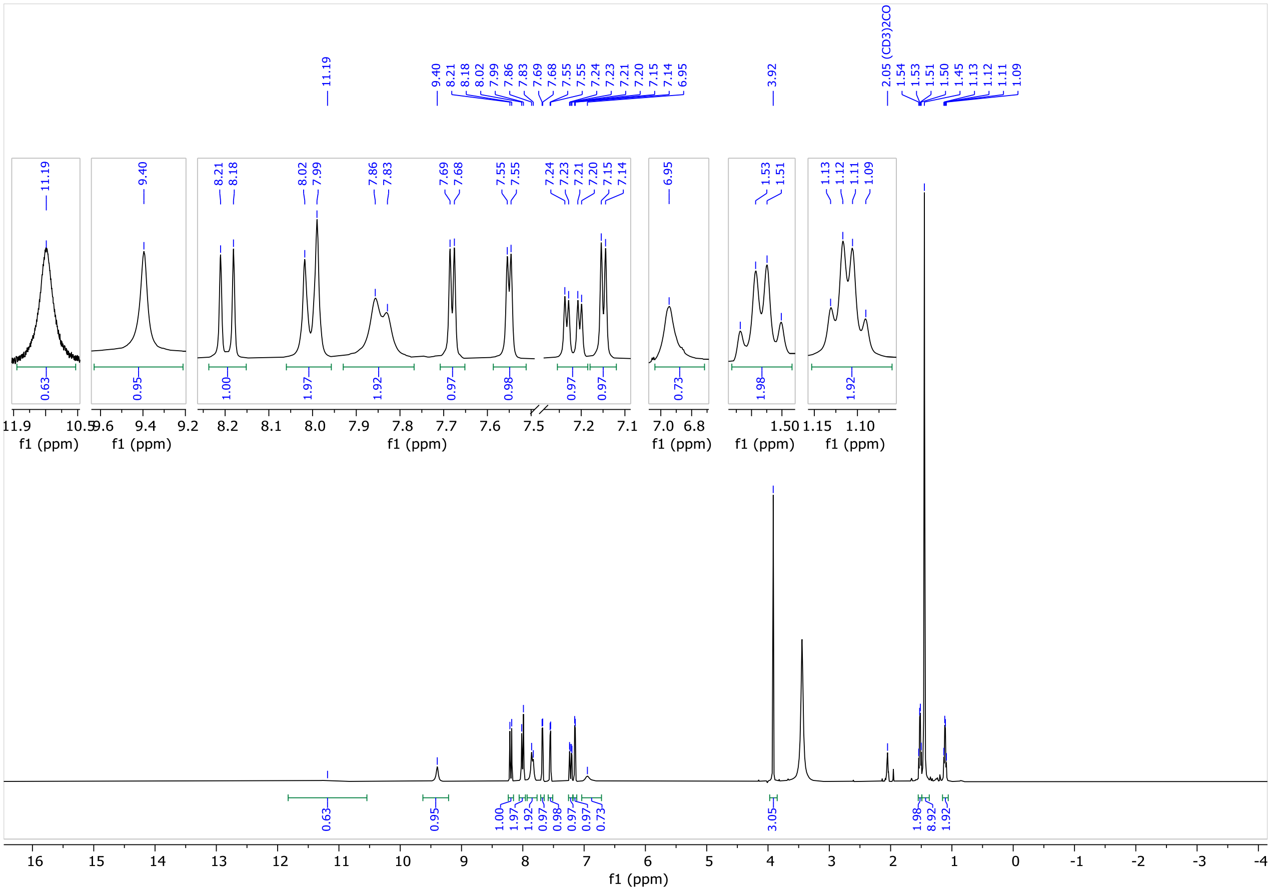

Figure S33. NMR Spectrum of ^{1}H of **13** (300 MHz, Acetone-d_6).

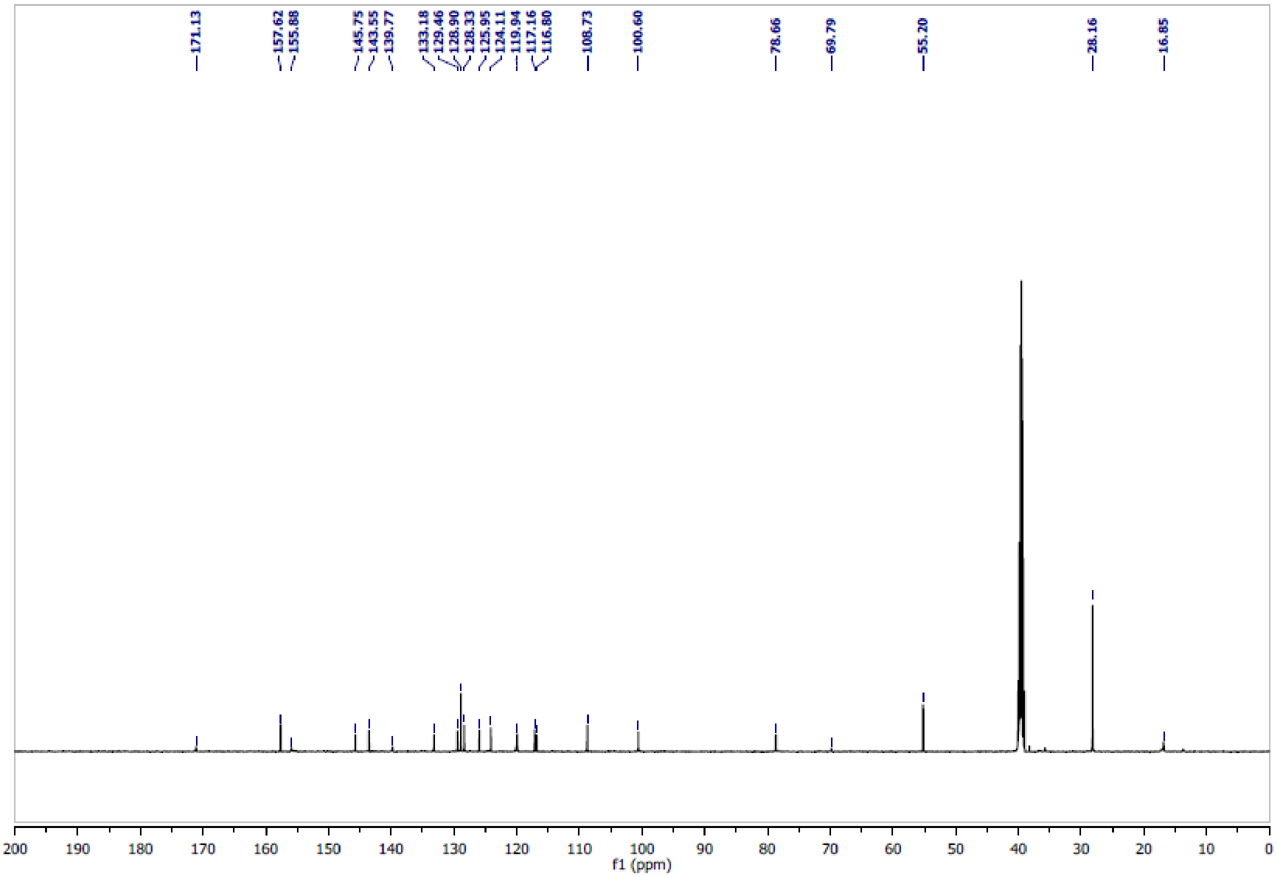

Figure S34. NMR Spectrum of ^{13}C of **13** (126 MHz, DMSO-d6).

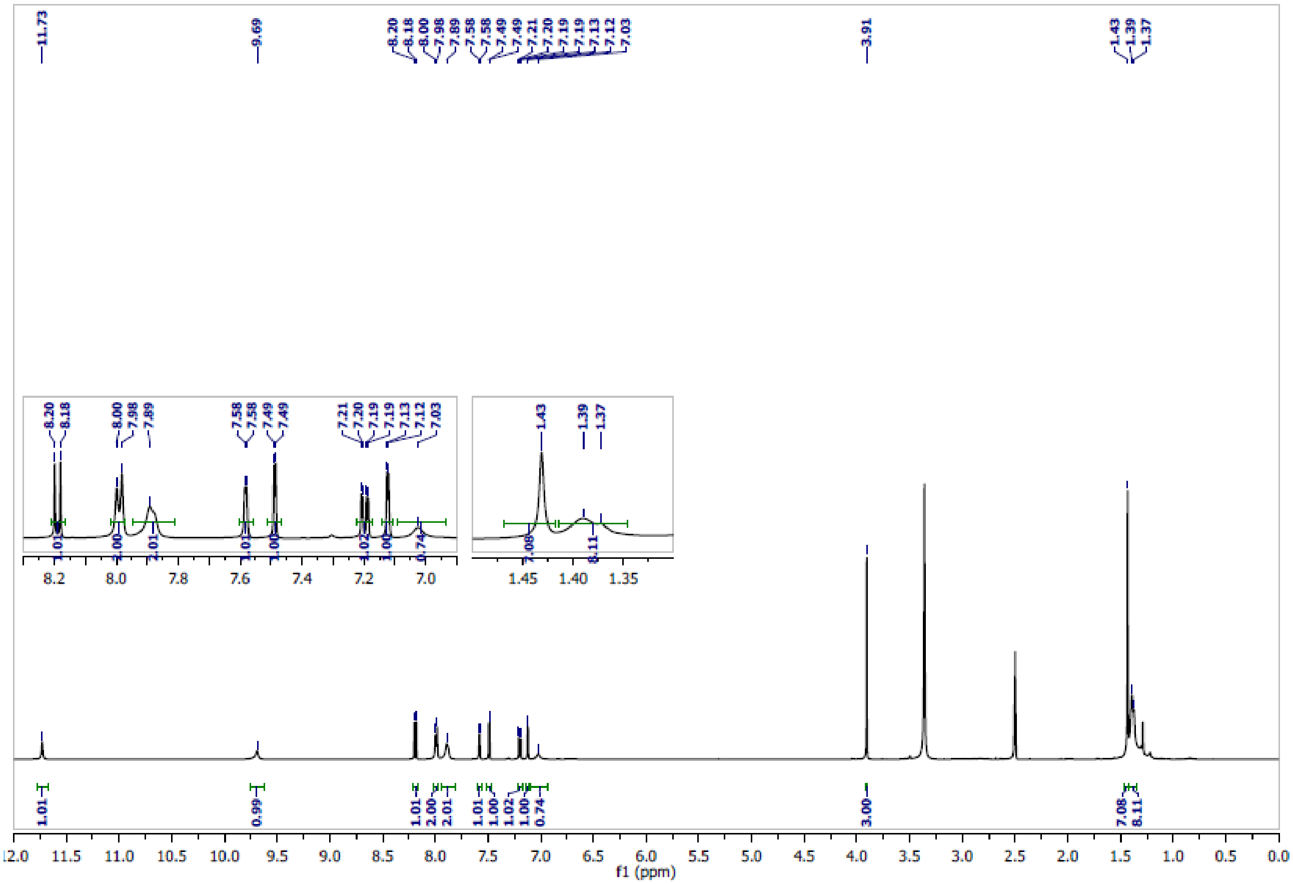

Figure S35. NMR Spectra of ^{1}H of **14** (500 MHz, DMSO-d6).

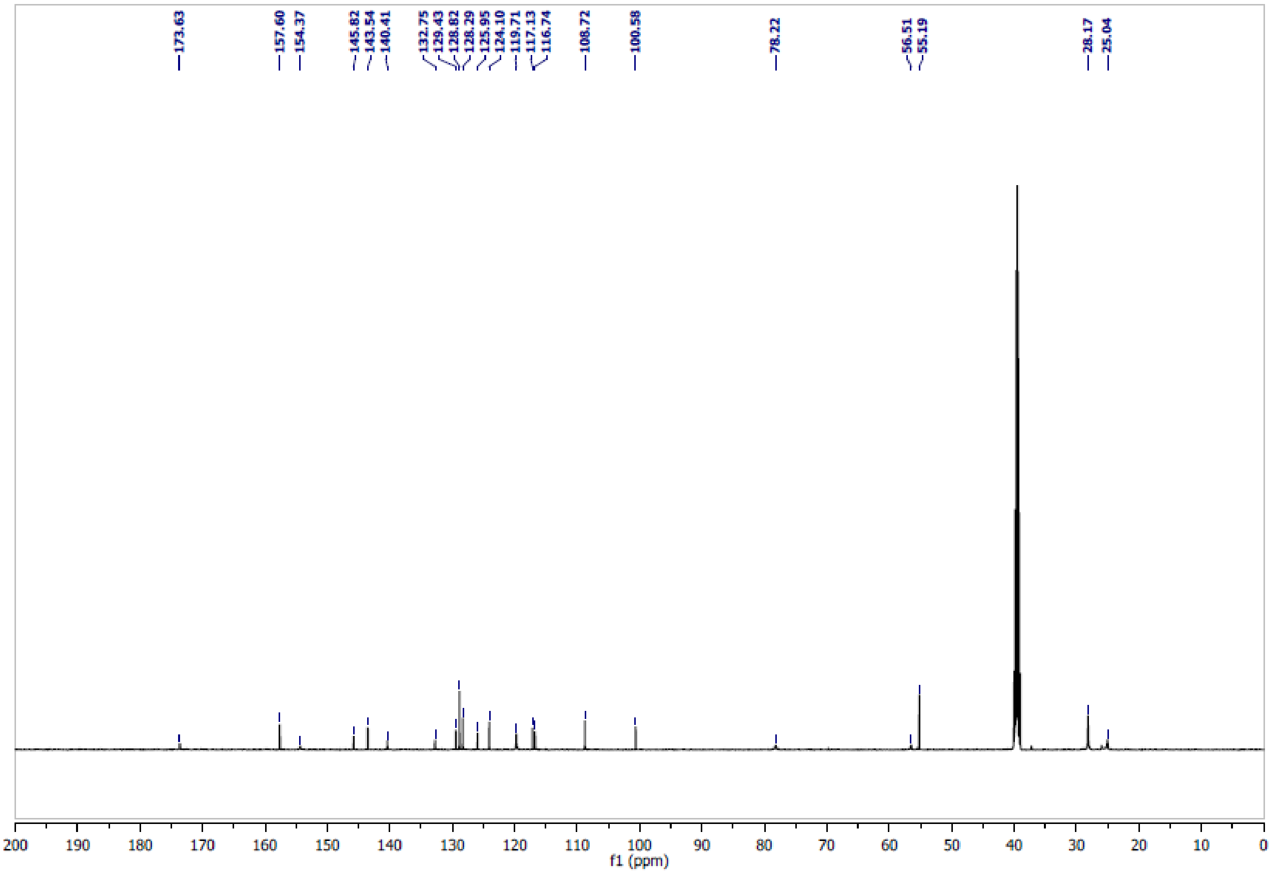

Figure S36. NMR Spectra of ^{13}C of **14** (126 MHz, DMSO-d6).

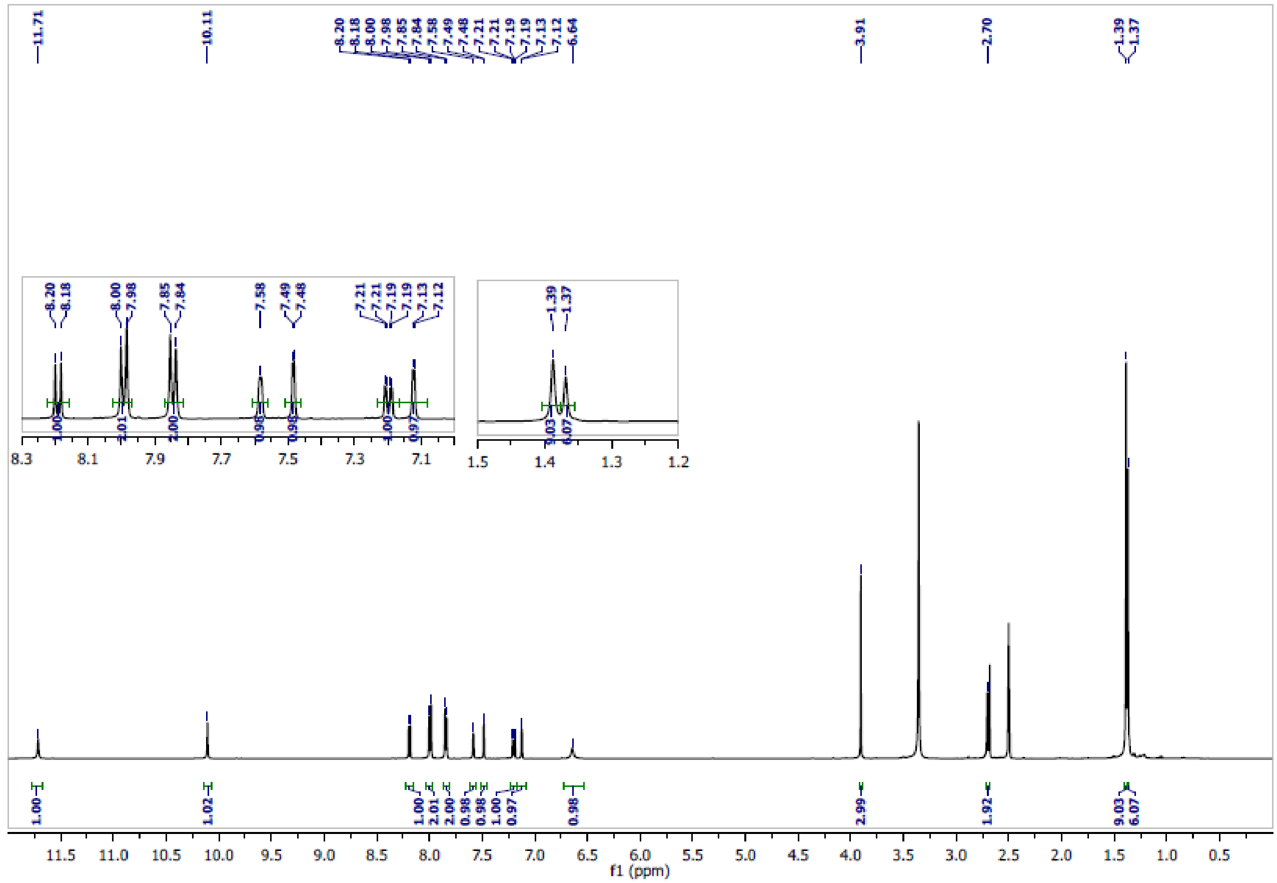

Figure S37. NMR Spectra of ^{1}H of **15** (500 MHz, DMSO-d6).

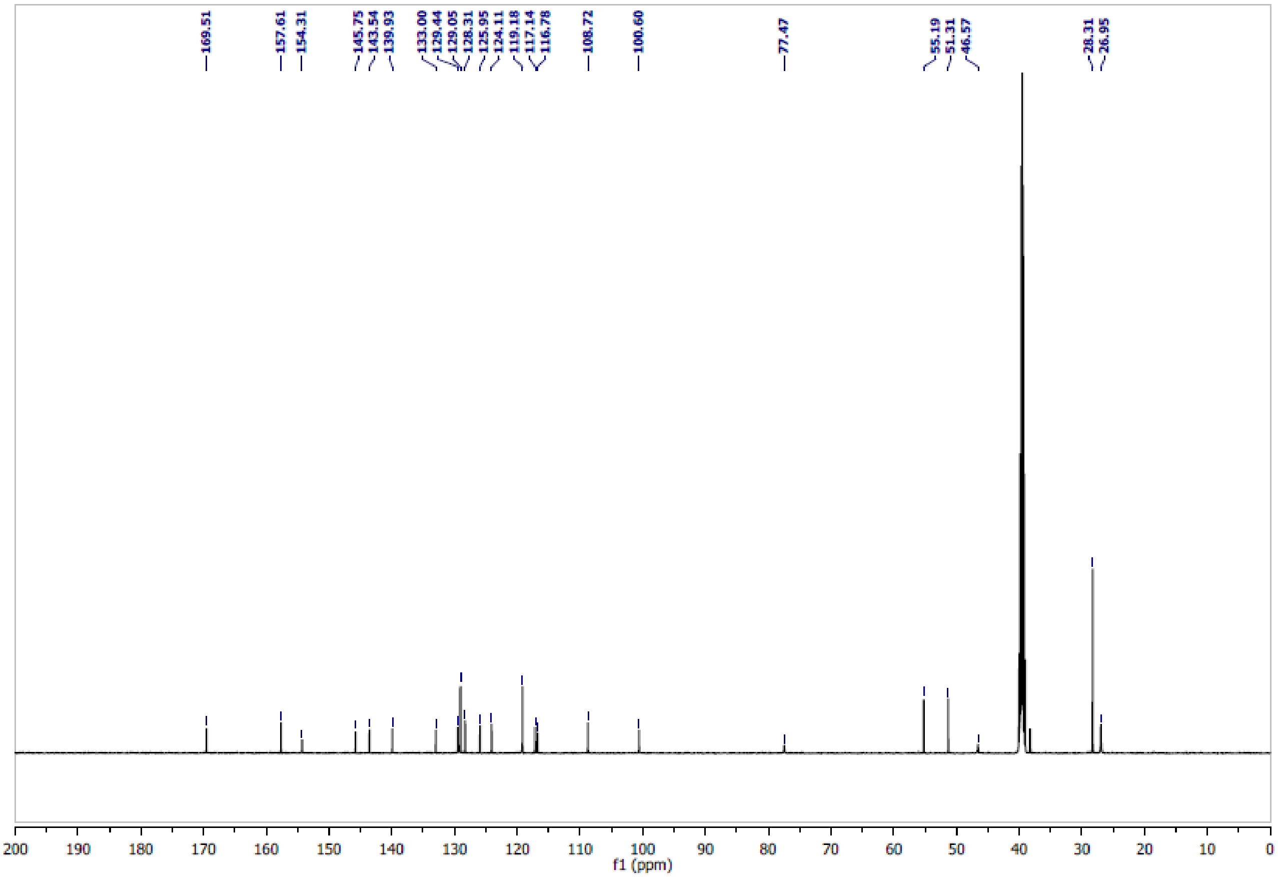

Figure S38. NMR Spectra of ^{13}C of **15** (126 MHz, DMSO-d6).

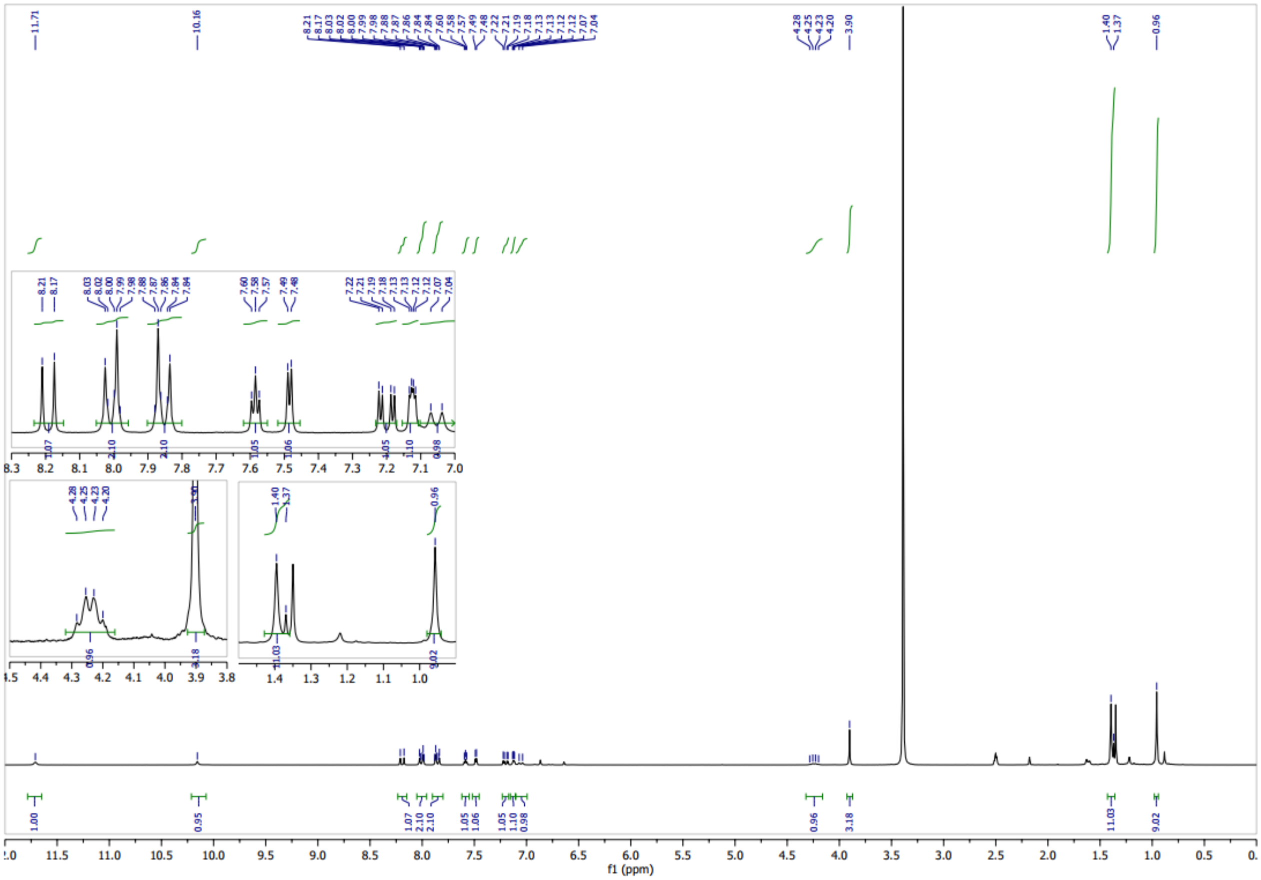

Figure S39. NMR Spectra of ^{1}H of **16** (250 MHz, DMSO-d6).

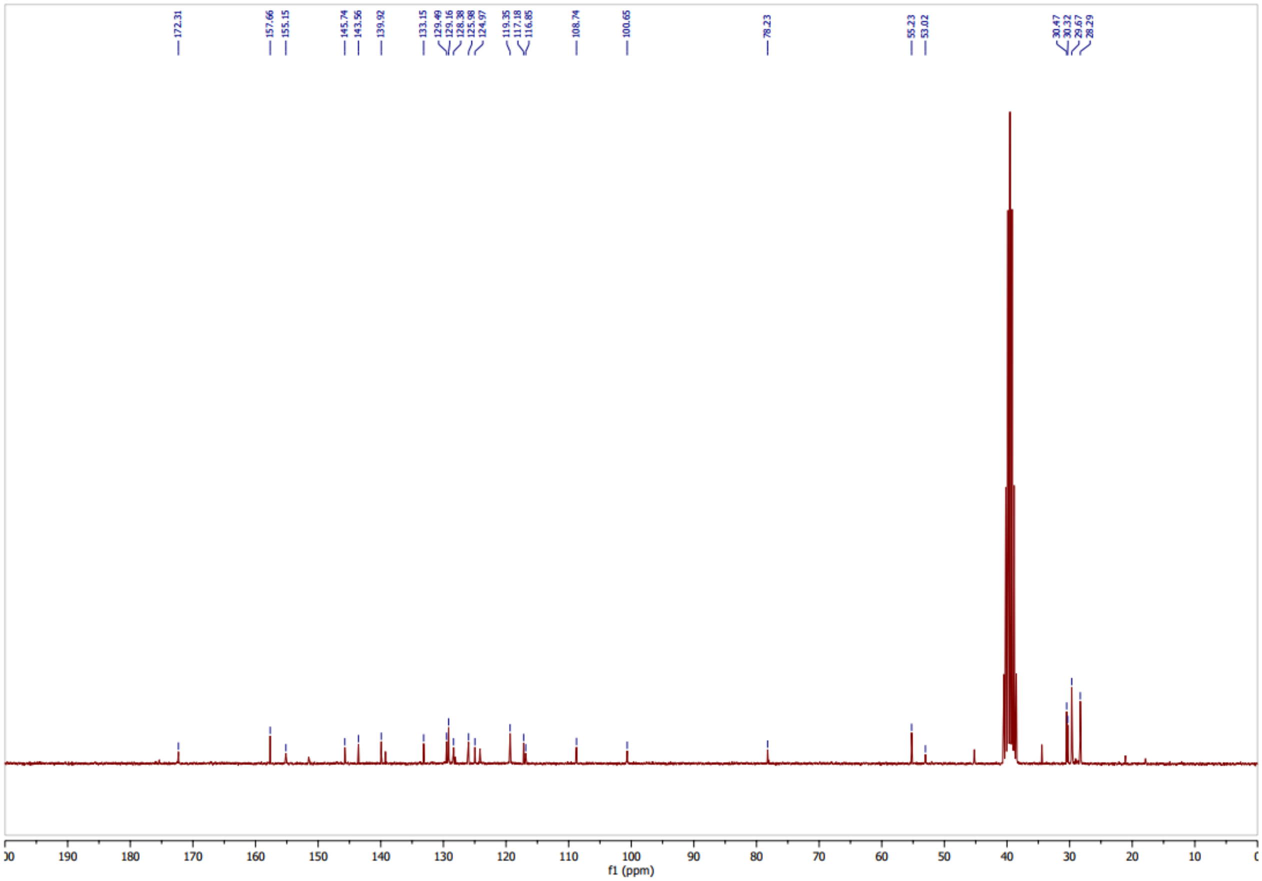

Figure S40. NMR Spectra of ^{13}C of **16** (63 MHz, DMSO-d6).

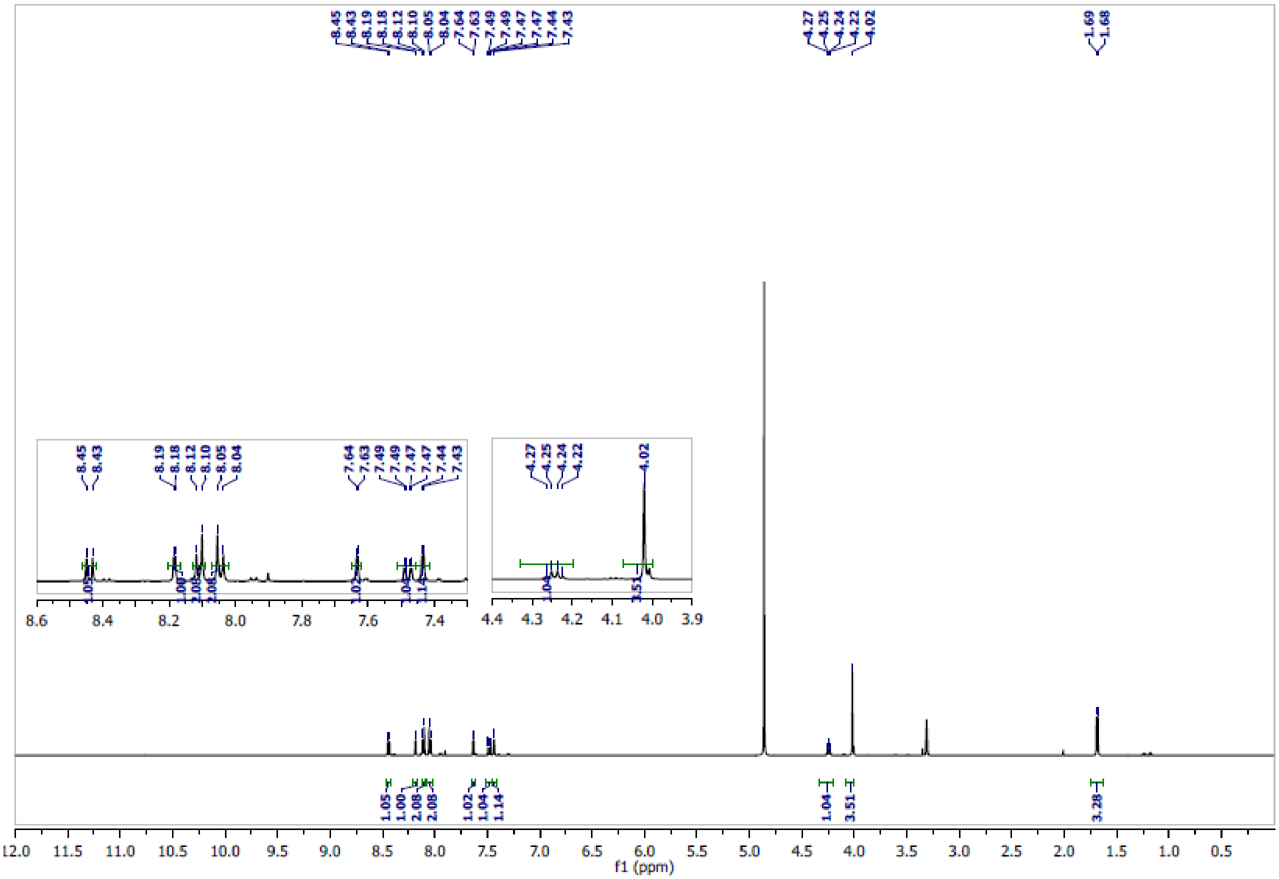

Figure S41. NMR Spectra of ^{1}H of **17** (500 MHz, MeOD).

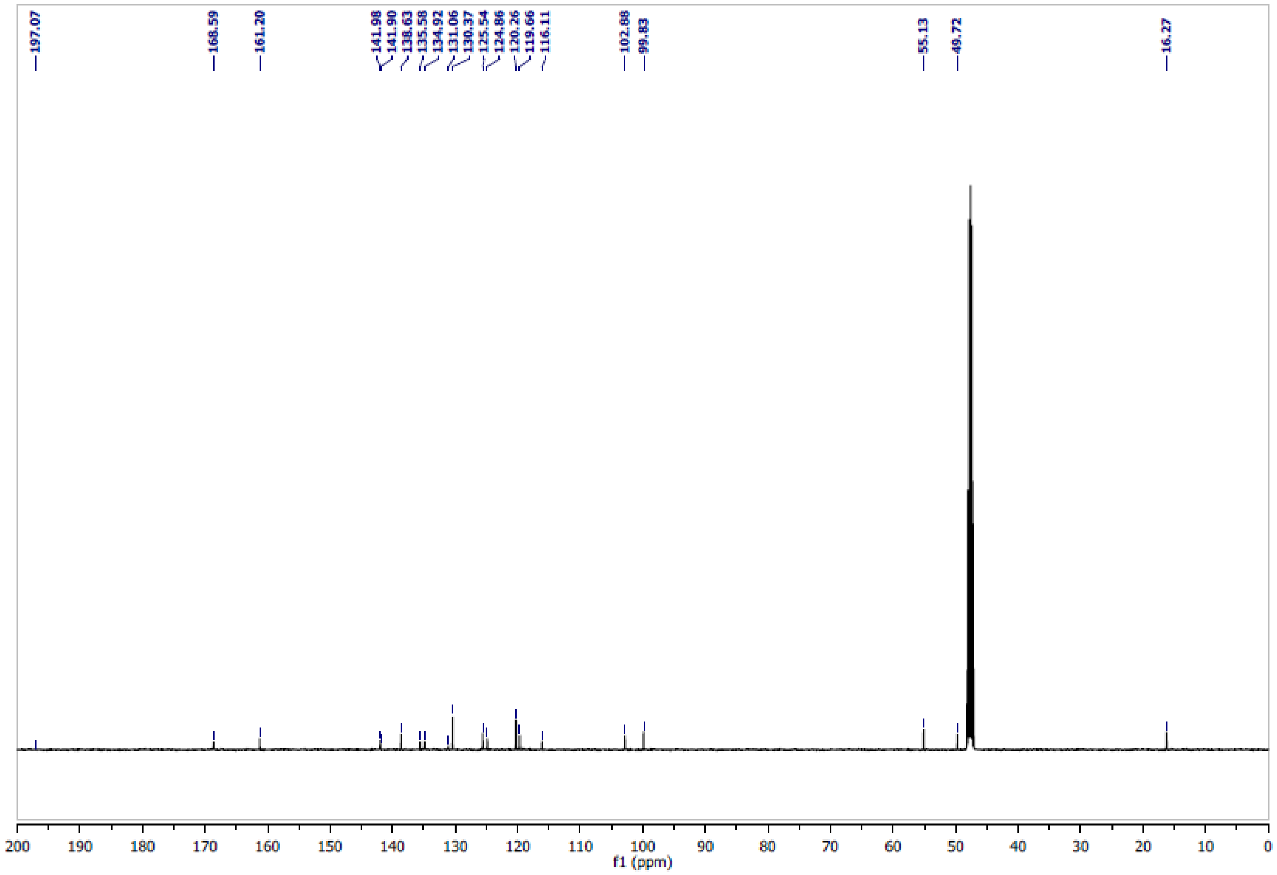

Figure S42. NMR Spectra of ^{13}C of **17** (126 MHz, MeOD).

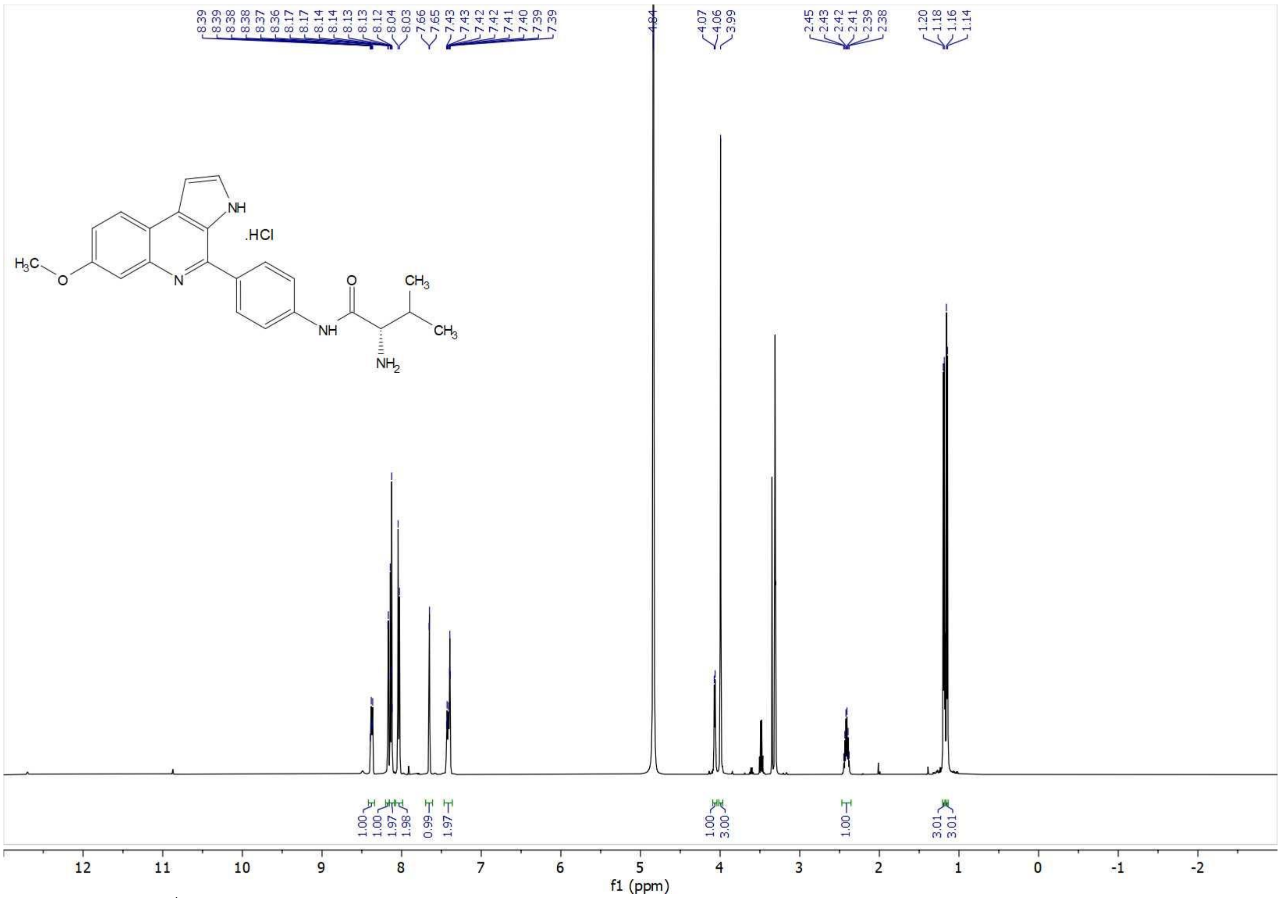

Figure S43. ^{1}H NMR spectra of **18** (500 MHz, MeOD).

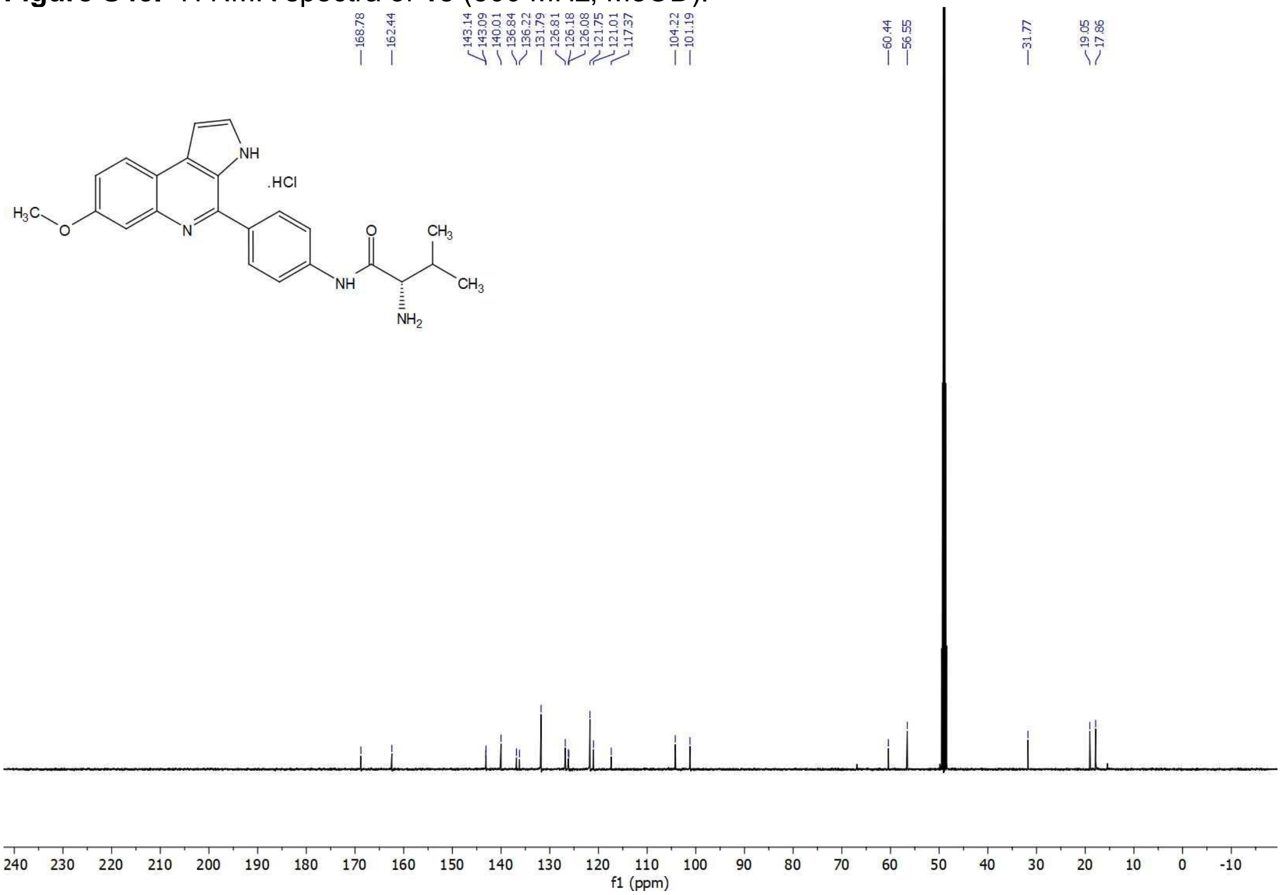

Figure S44. ^{13}C NMR spectra of **18** (126 MHz, MeOD).

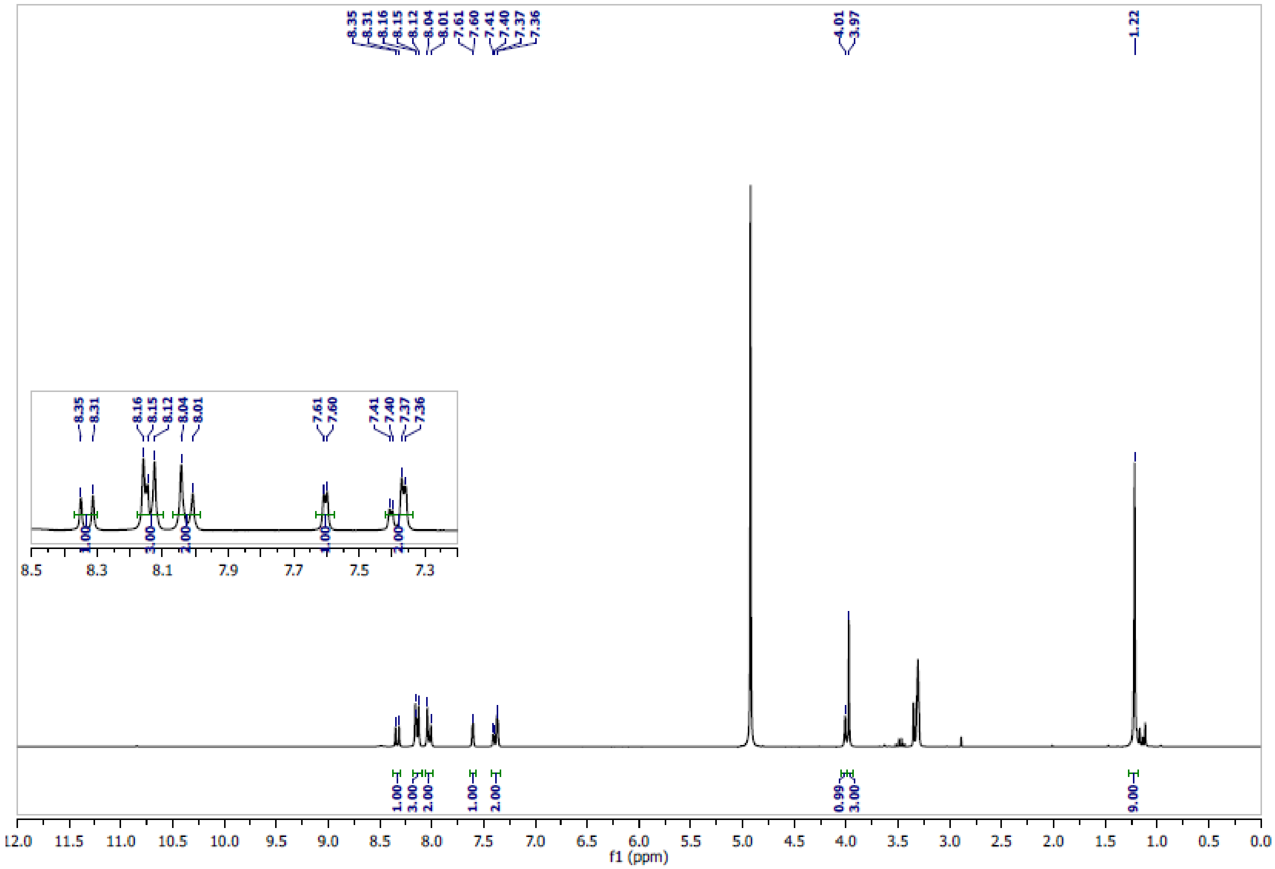

Figure S45. NMR Spectra of ^{1}H of **19** (250 MHz, MeOD).

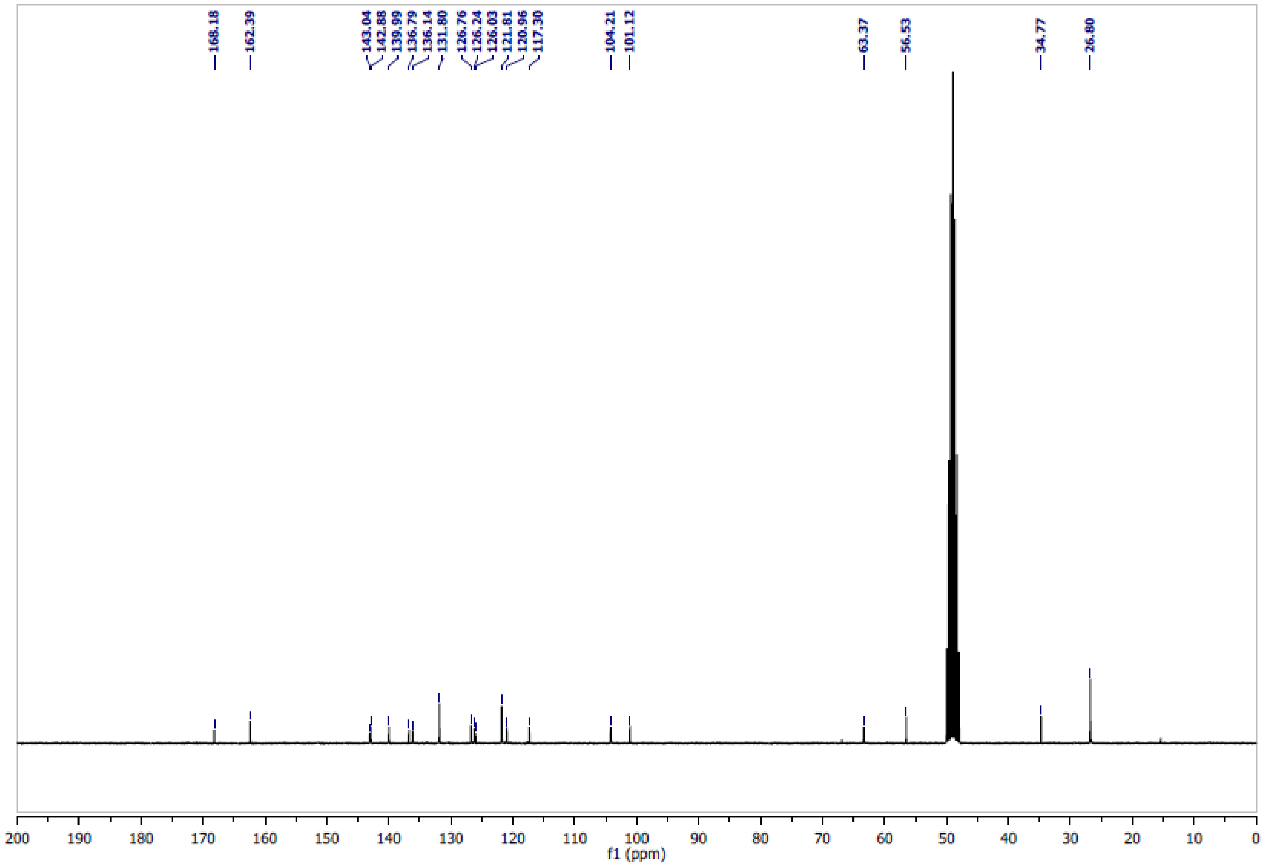

Figure S46. NMR Spectra of ^{13}C of **19** (63 MHz, MeOD).

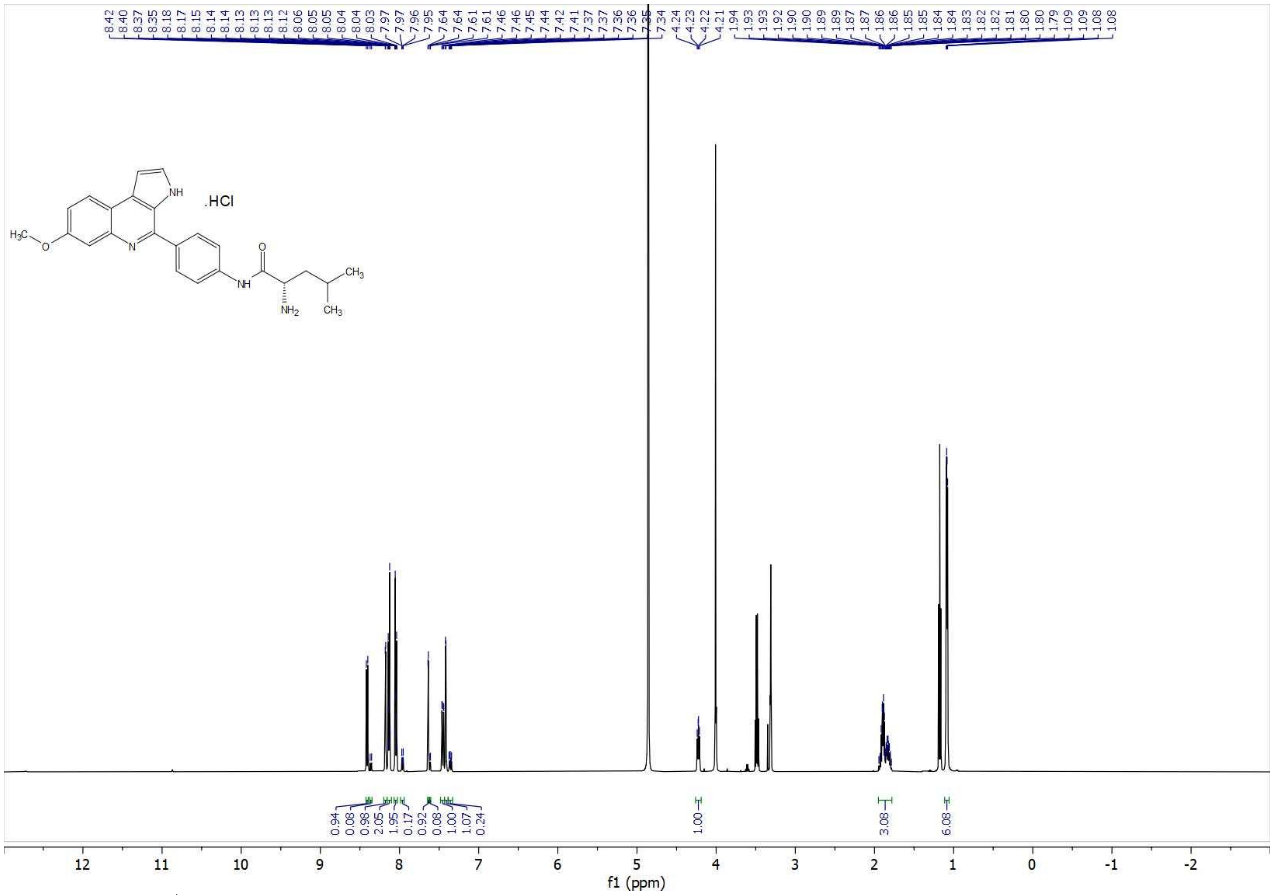

Figure S47. ^{1}H NMR spectra of **20** (500 MHz, MeOD).

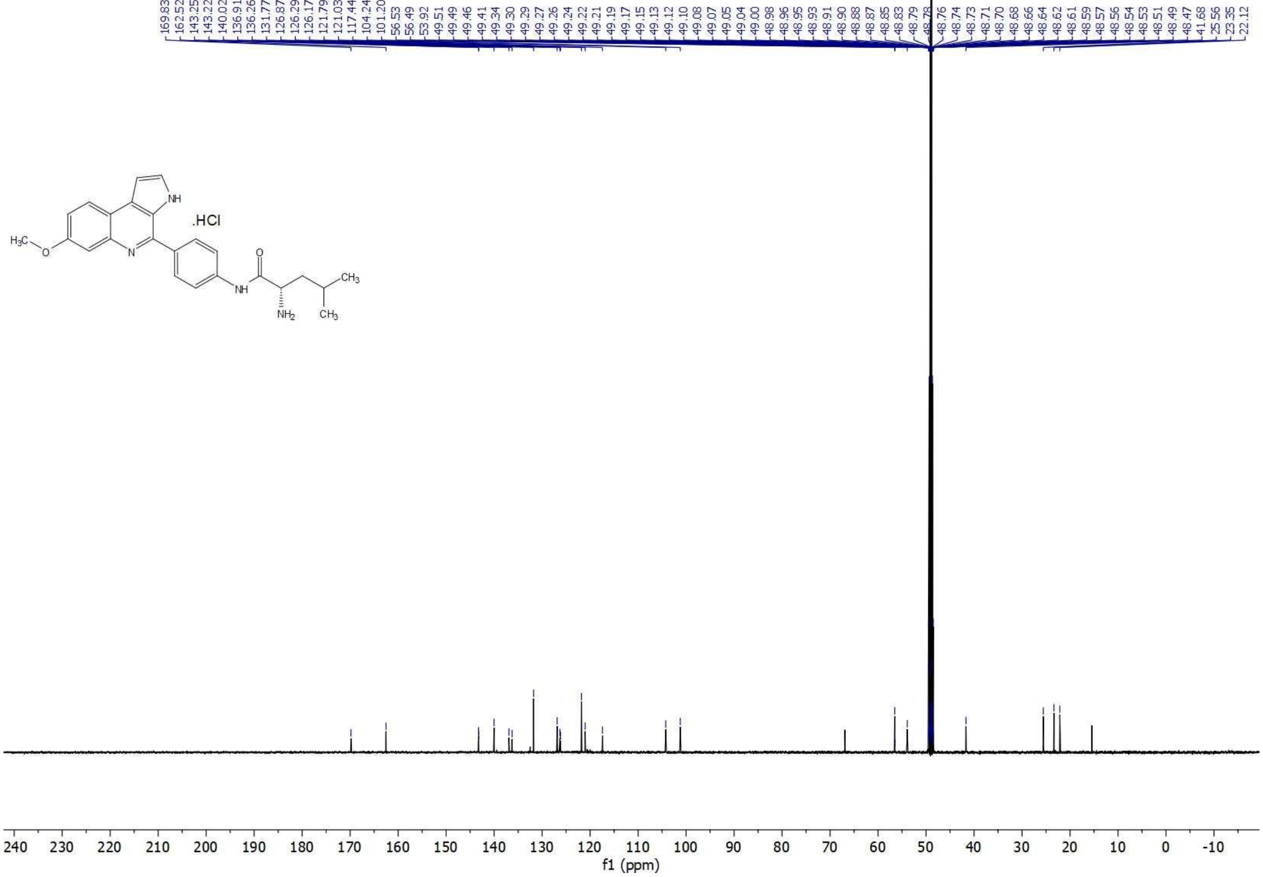

Figure S48. ^{13}C NMR spectra of **20** (126 MHz, MeOD).

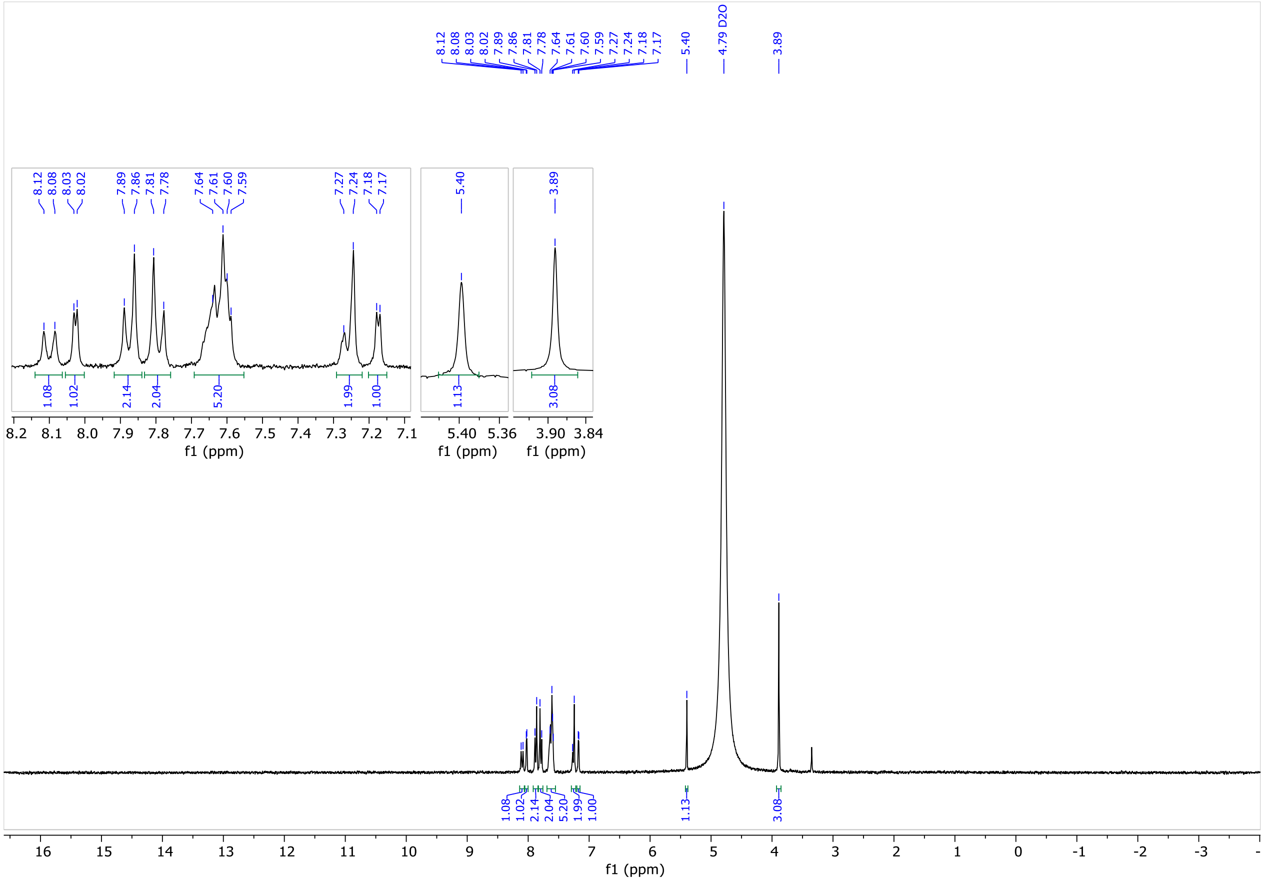

Figure S49. NMR Spectra of ^{1}H of **21** (300 MHz, D_2O).

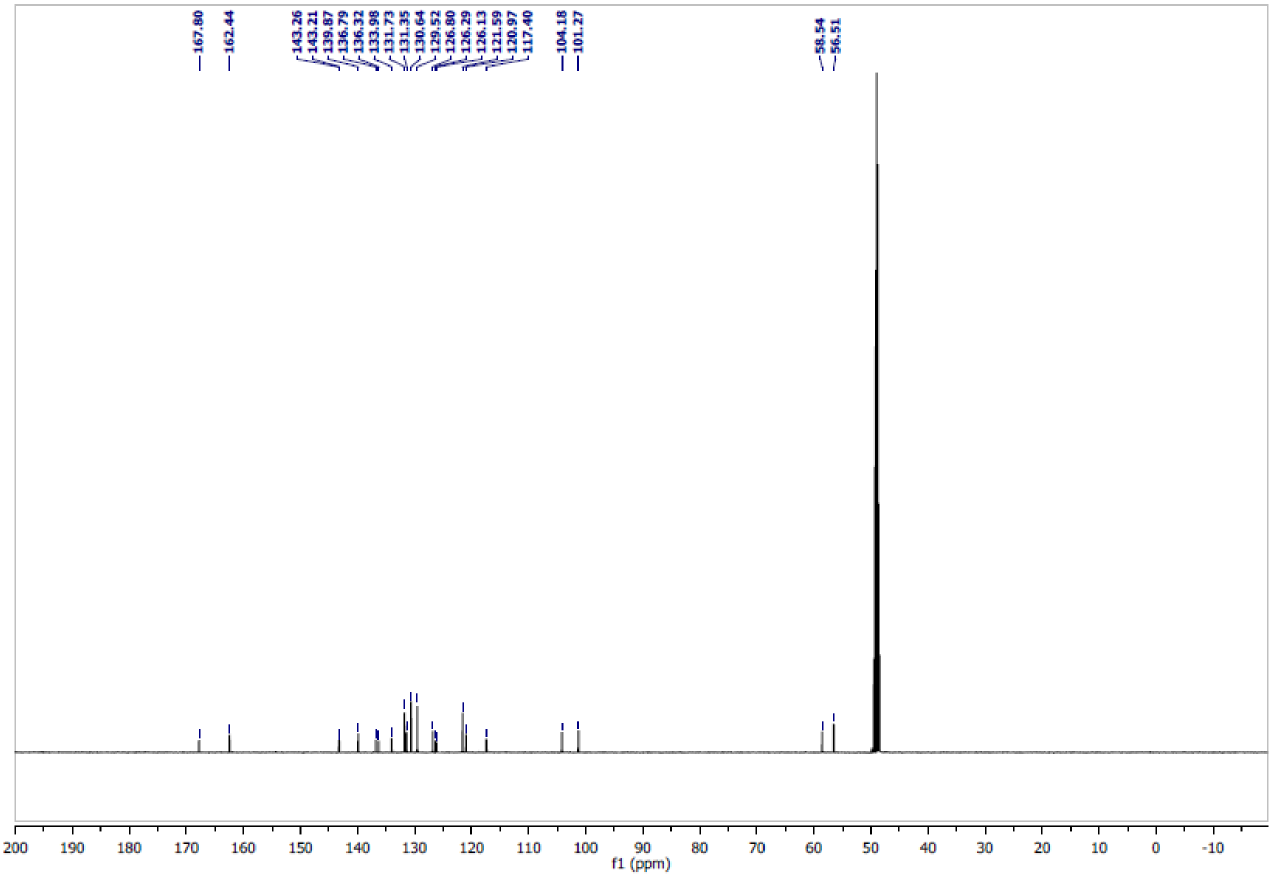

Figure S50. NMR Spectra of ^{13}C of **21** (126 MHz, MeOD).

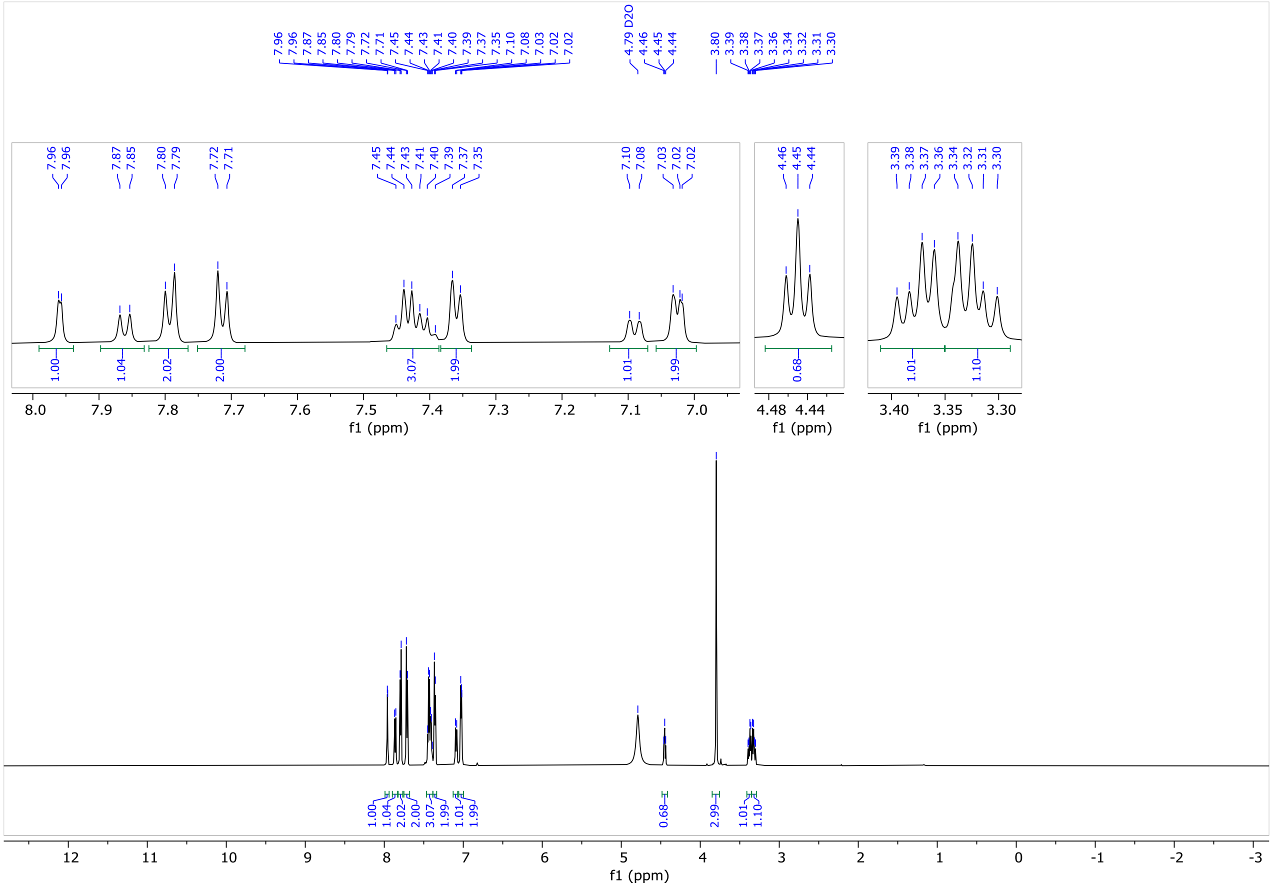

Figure S51. NMR Spectra of ^{1}H of **22** (600 MHz, D_2O, water-suppressed using the noesygppr1d pulse sequence).

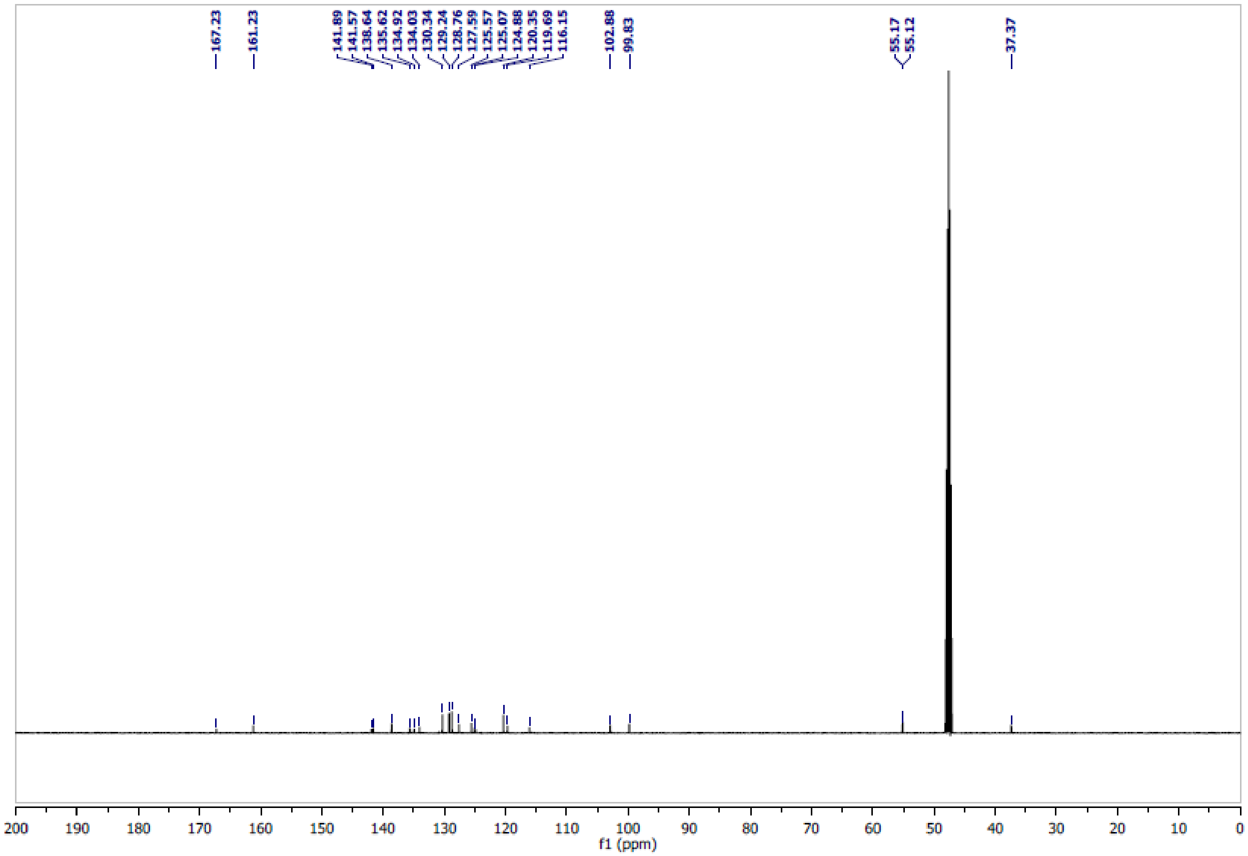

Figure S52. NMR Spectra of ^{13}C of **22** (126 MHz, MeOD).

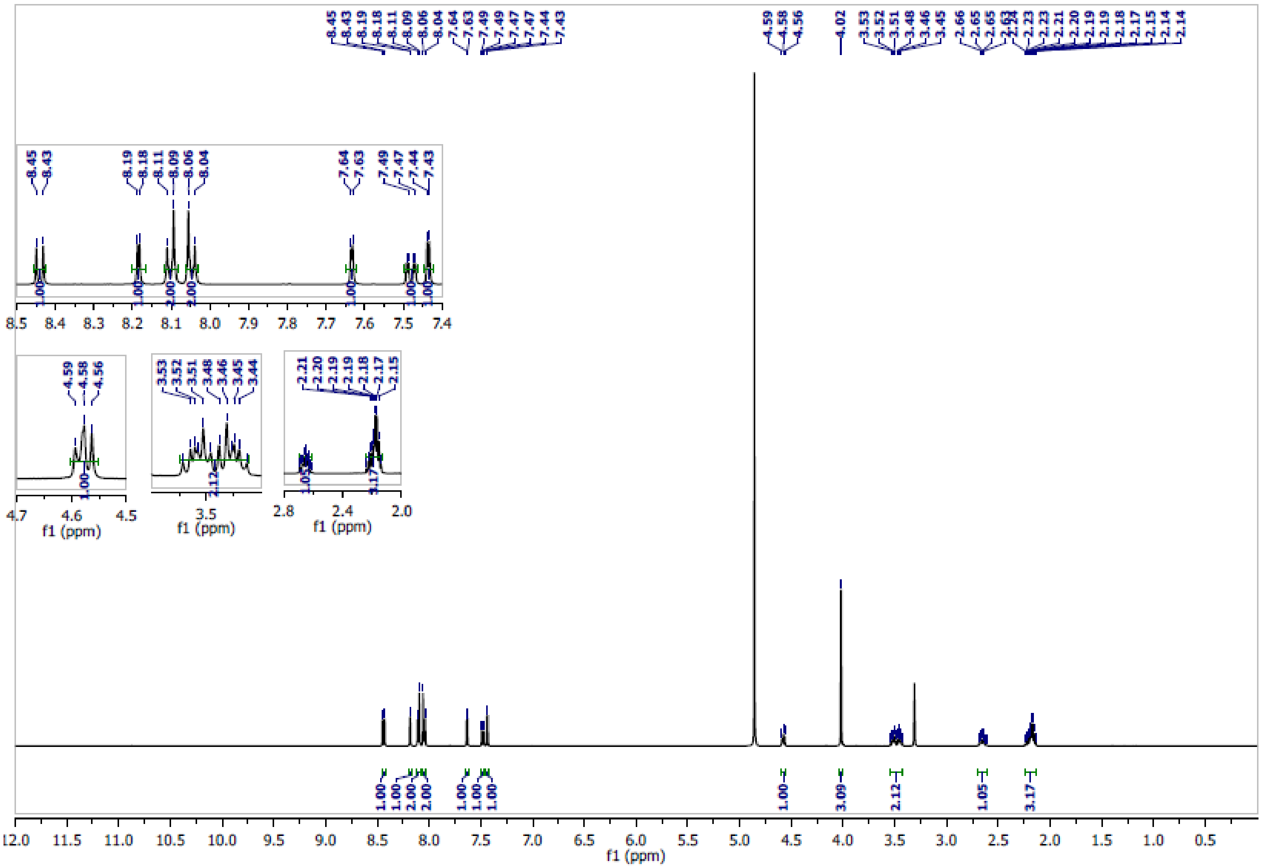

Figure S53. NMR Spectra of ^{1}H of **23** (500 MHz, MeOD).

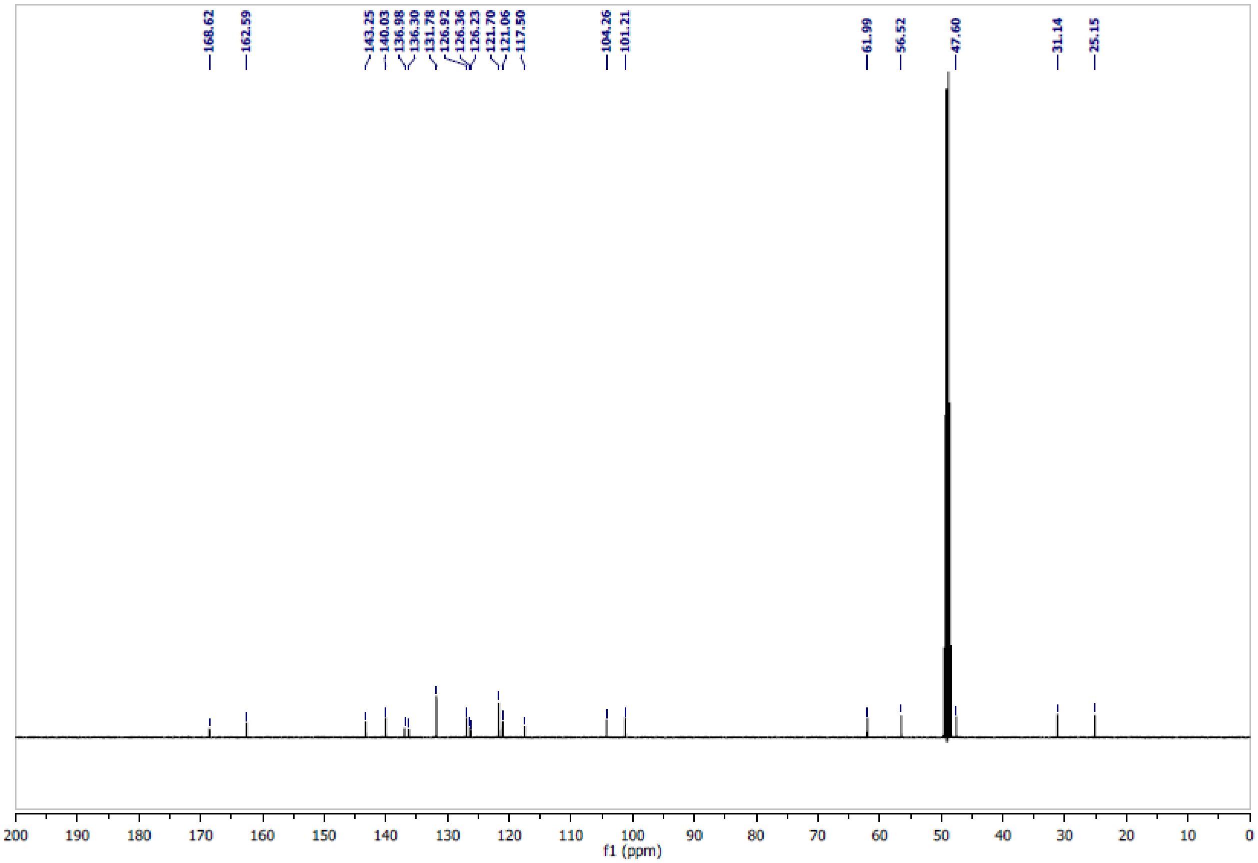

Figure S54. NMR Spectra of ^{13}C of **23** (126 MHz, MeOD).

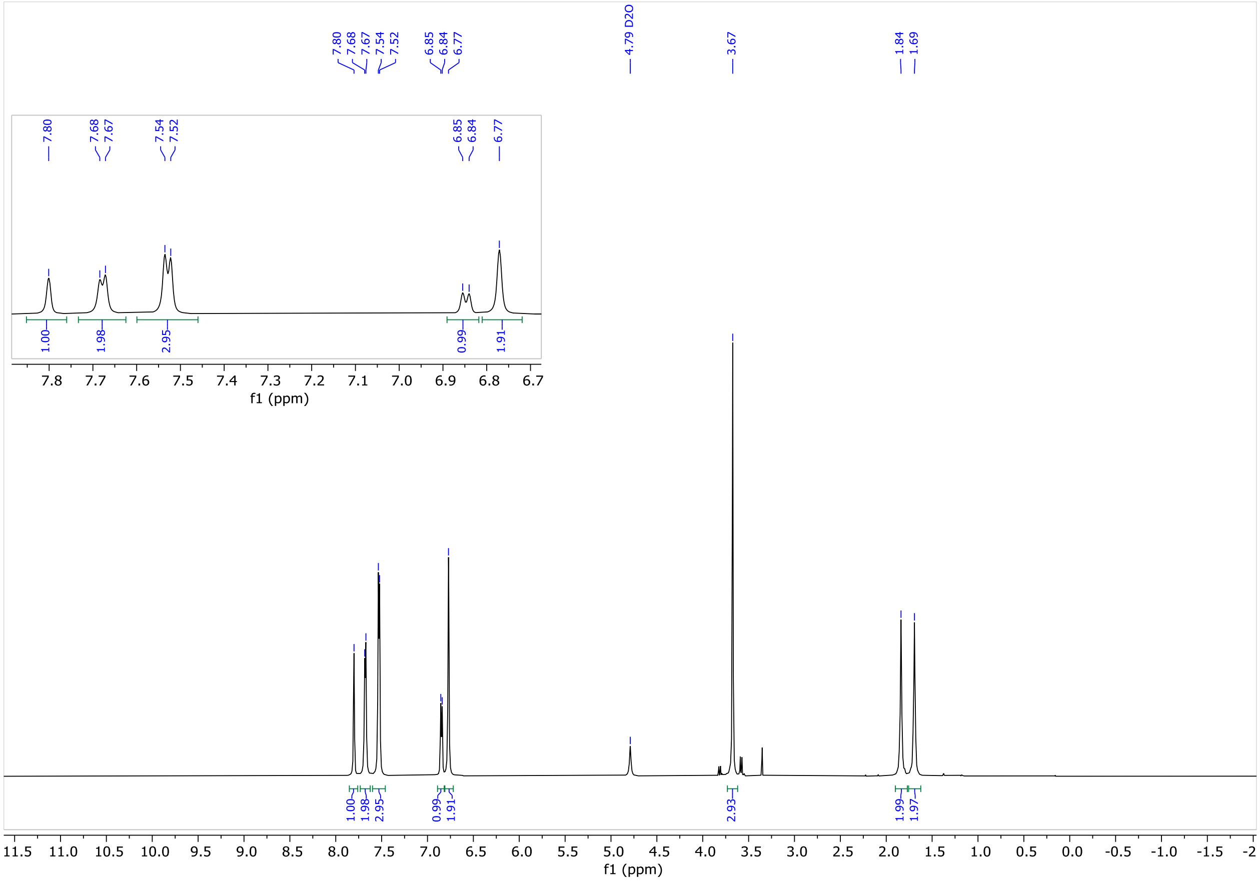

Figure S55. NMR Spectra of ^{1}H of **24** (600 MHz, D_2O, water-suppressed using the noesygppr1d pulse sequence).

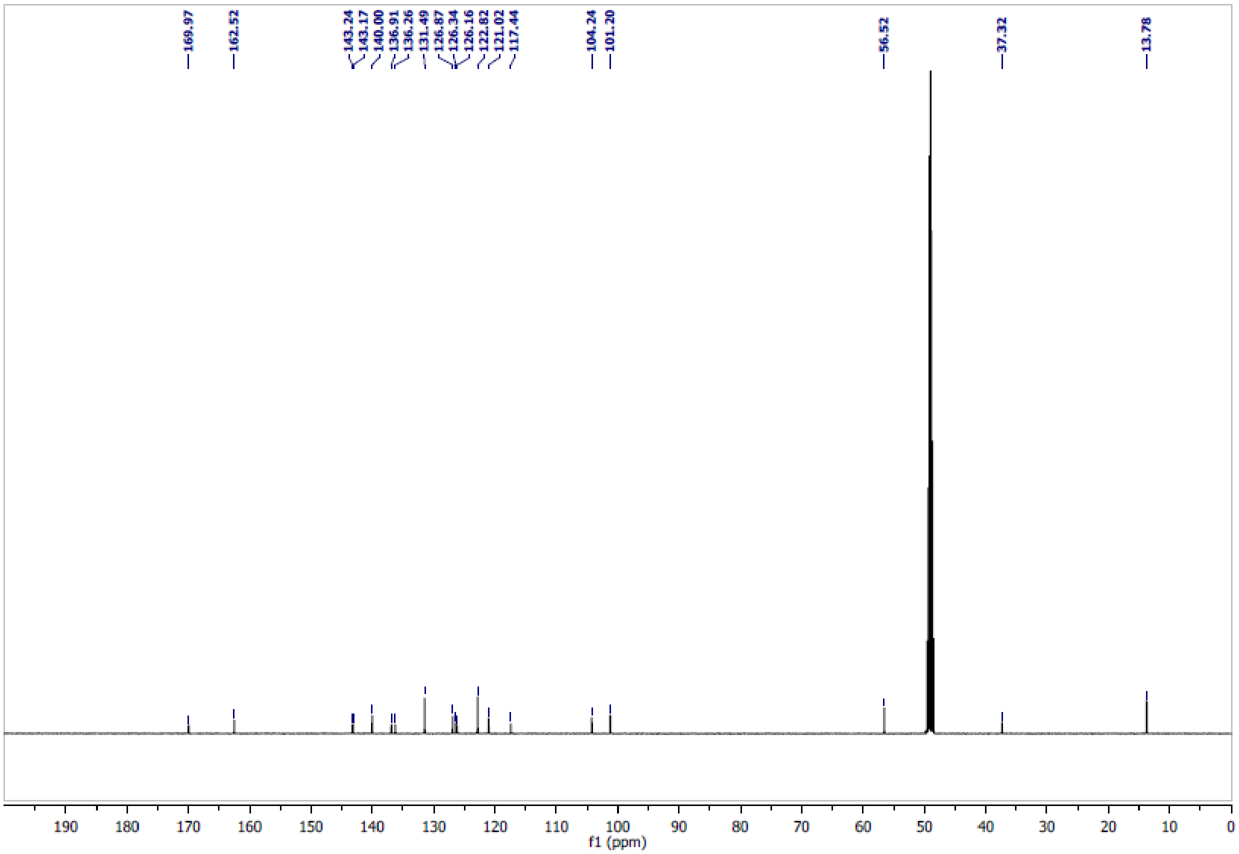

Figure S56. NMR Spectra of ^{13}C of **24** (126 MHz, DMSO-d6).

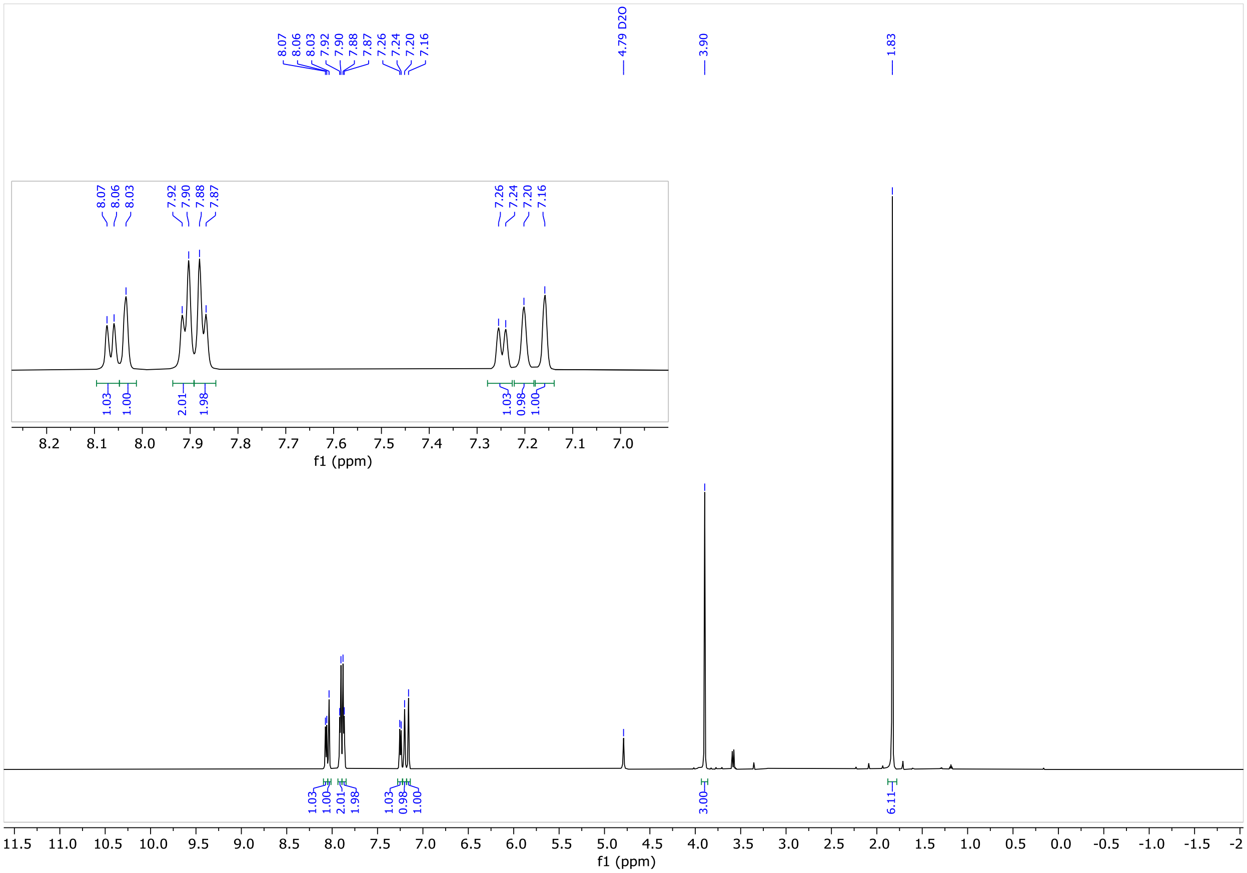

Figure S57. NMR Spectra of ^{1}H of **25** (600 MHz, D_2O, water-suppressed using the noesygppr1d pulse sequence).

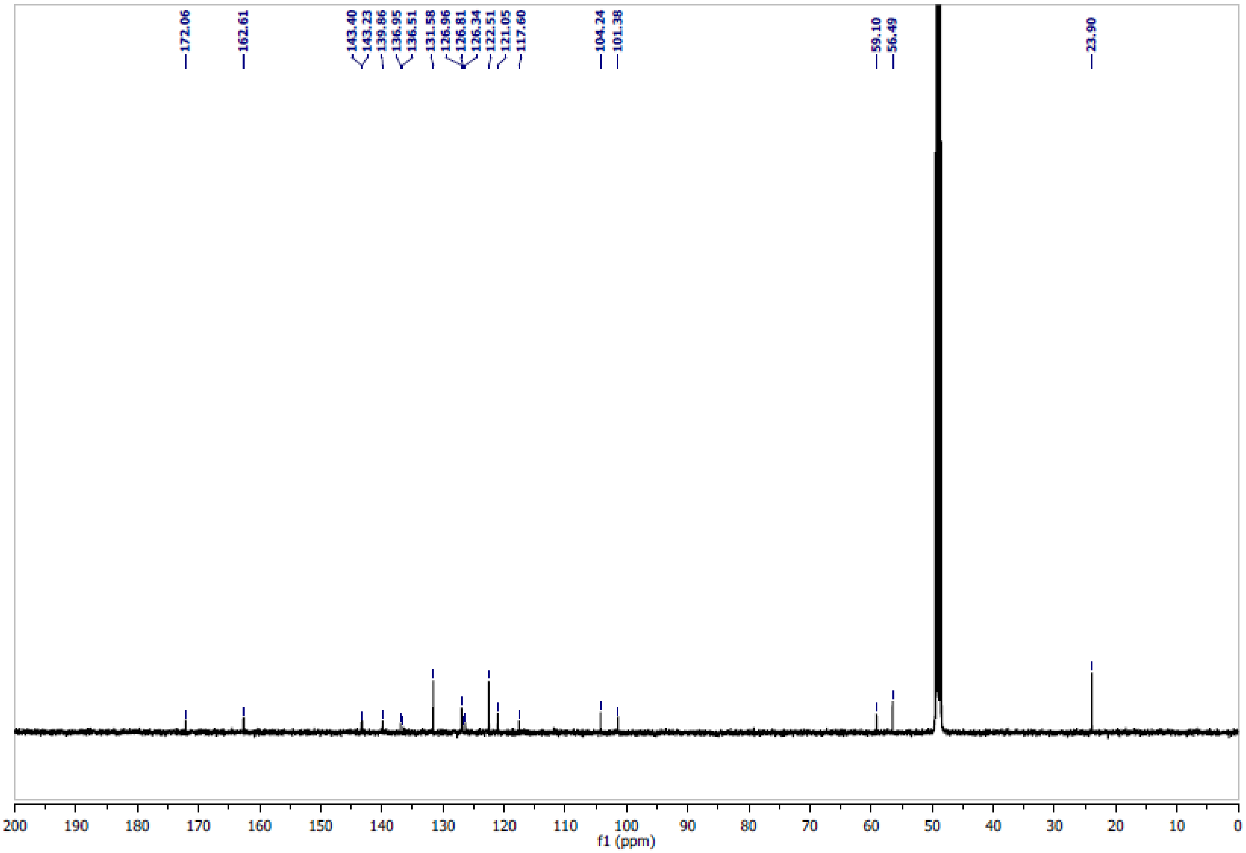

Figure S58. NMR Spectra of ^{13}C of **25** (126 MHz, MeOD).

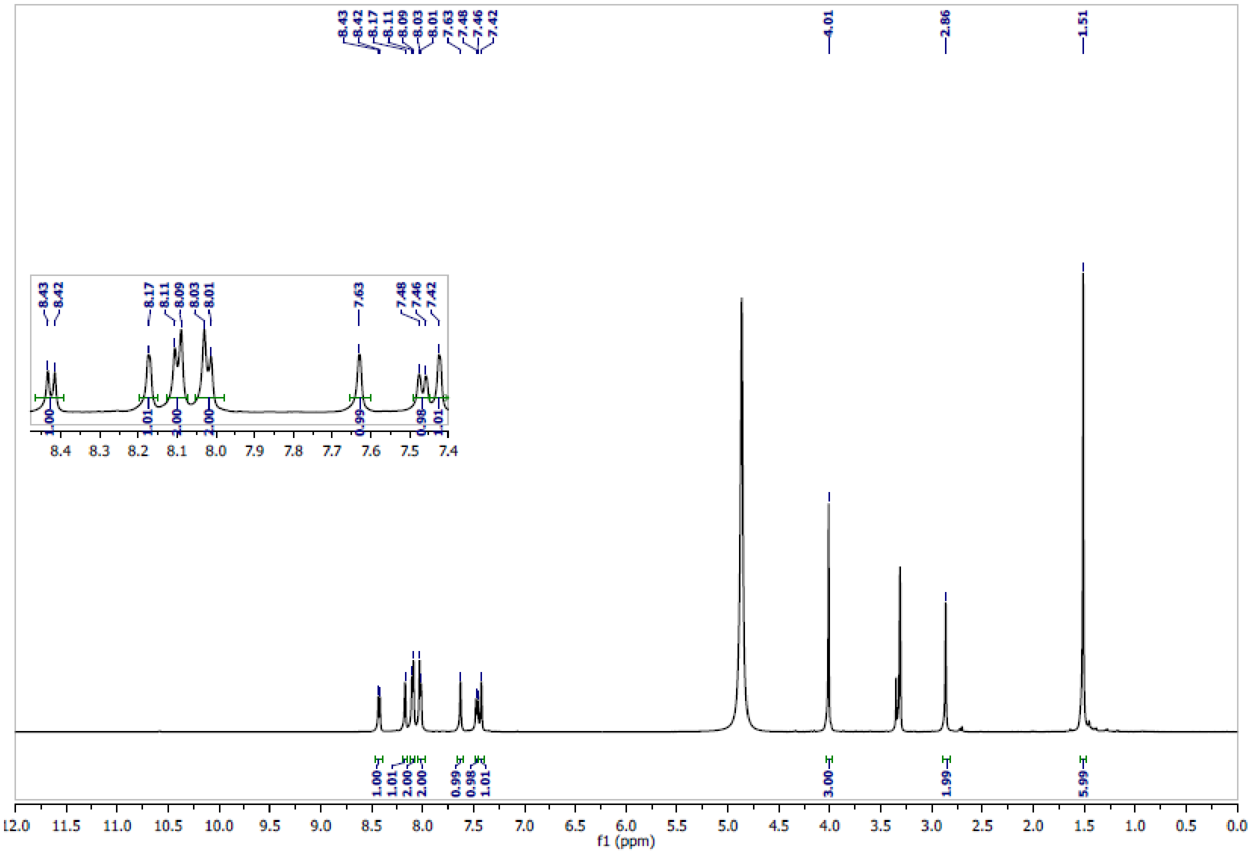

Figure S59. NMR Spectra of ^{1}H of **26** (500 MHz, MeOD).

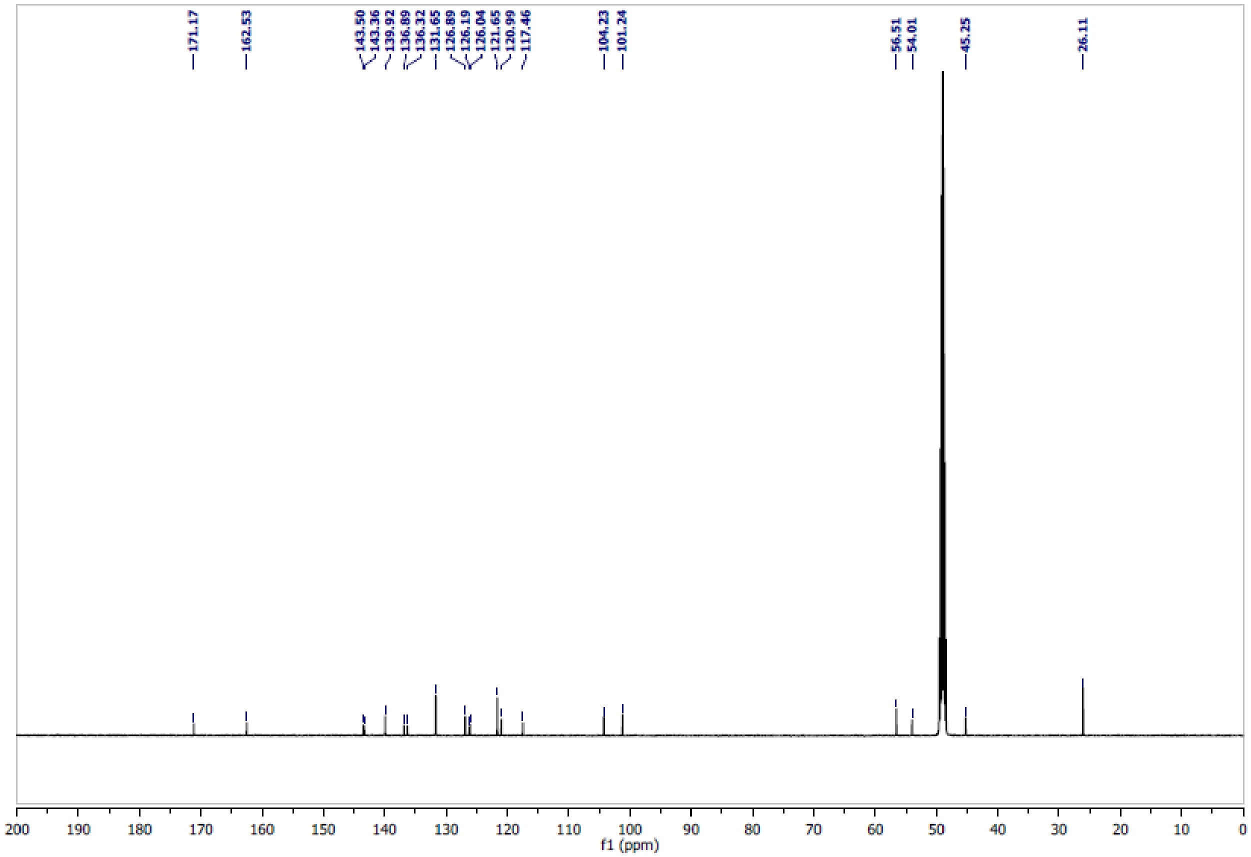

Figure S60. NMR Spectra of ^{13}C of **26** (126 MHz, MeOD).

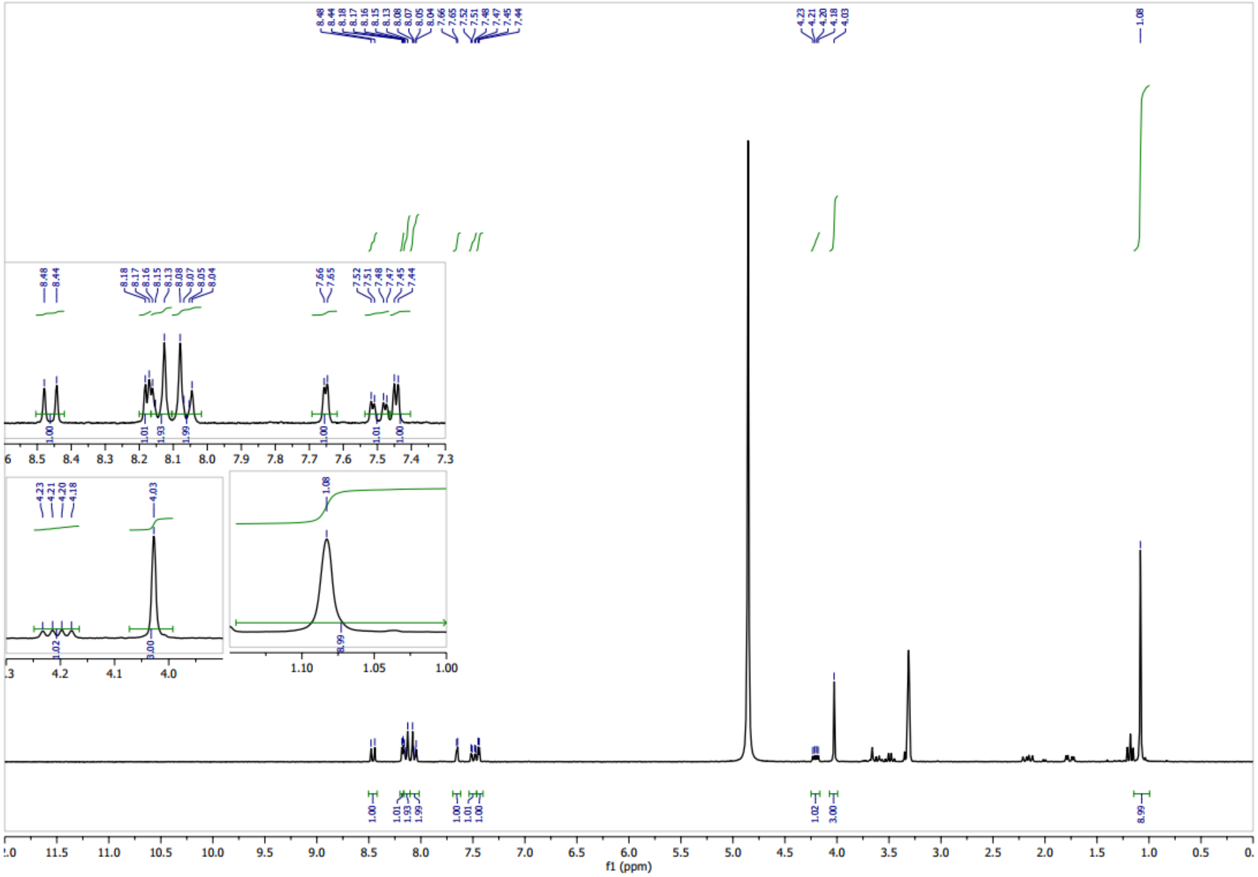

Figure S61. NMR Spectra of ^{1}H of **27** (250 MHz, MeOD).

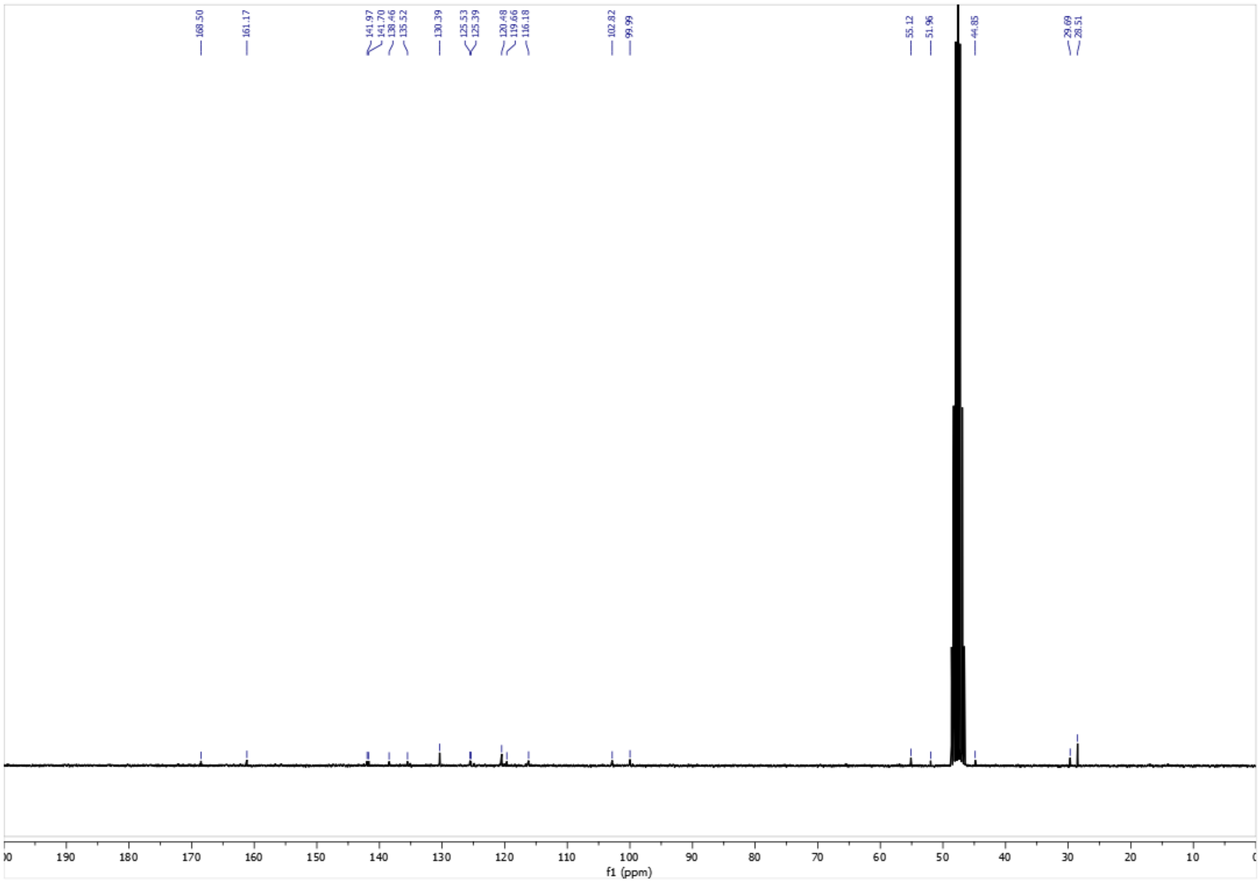

Figure S62. NMR Spectra of ^{13}C of **27** (126 MHz, MeOD).

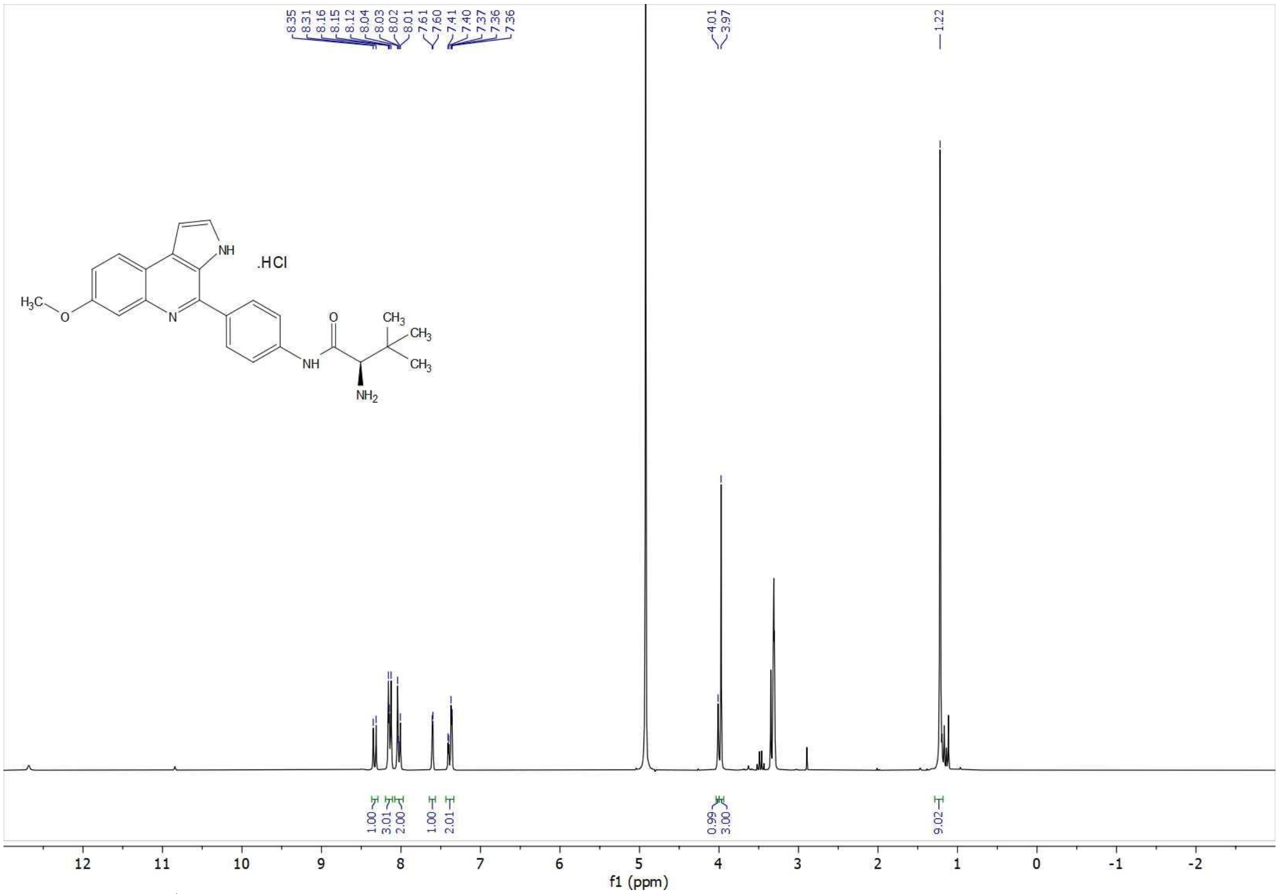

Figure S63. ^{1}H NMR spectra of **28** (250 MHz, MeOD).

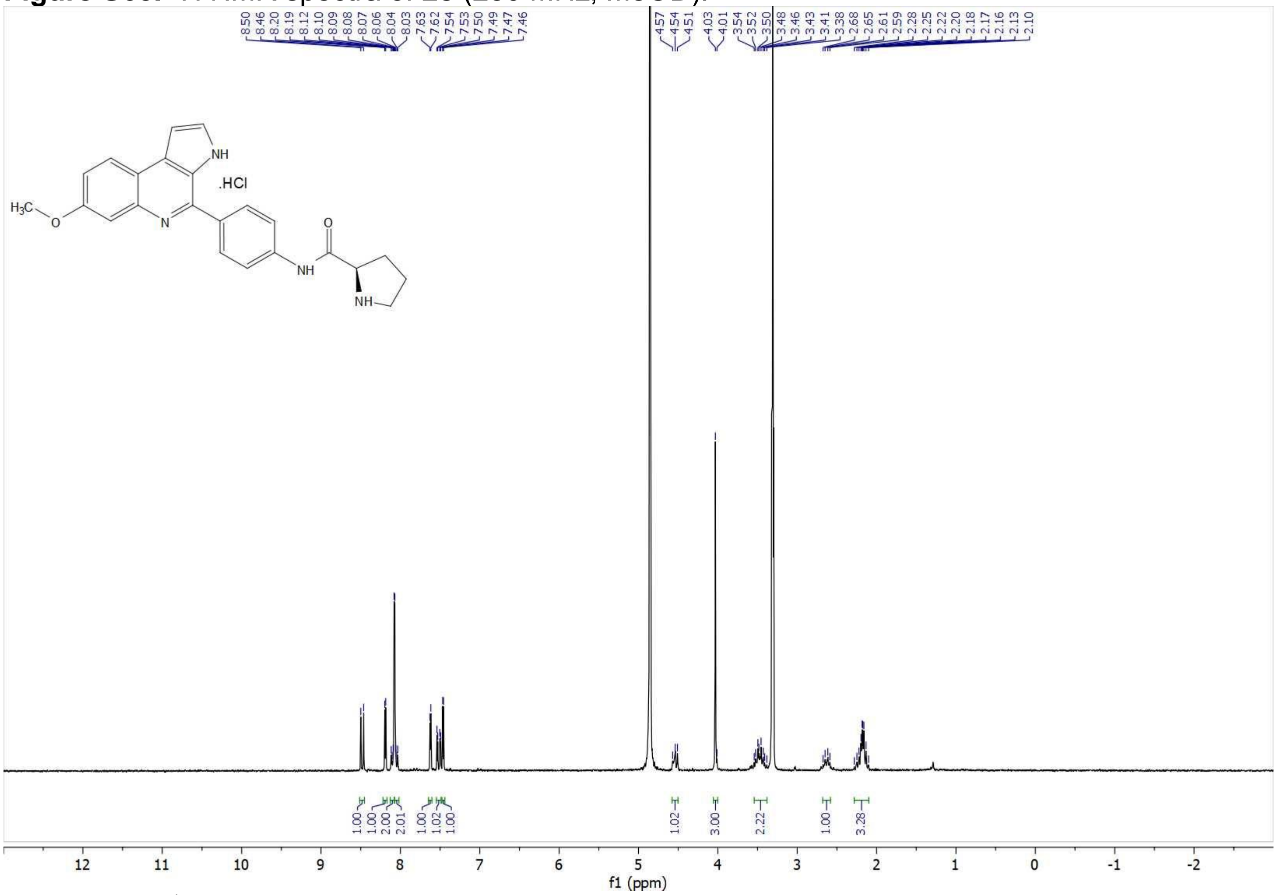

Figure S64. ^{1}H NMR spectra of **29** (250 MHz, MeOD).

(±)-**16** – starting material for (±)-**27**:

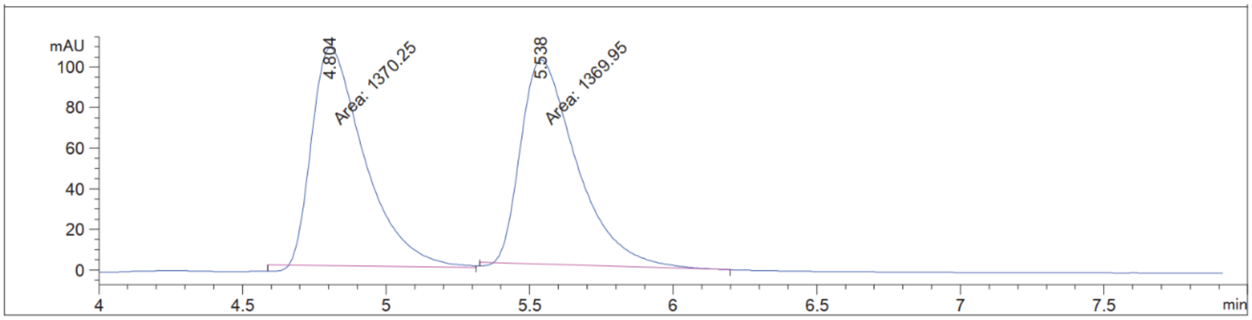

Peak #	RetTime [min]	Area [mAu*s]	Area %
1	4.804	1370.24792	50.0054
2	5.538	1369.95471	49.9946

(−)-**16** – starting material for (+)-**27:**

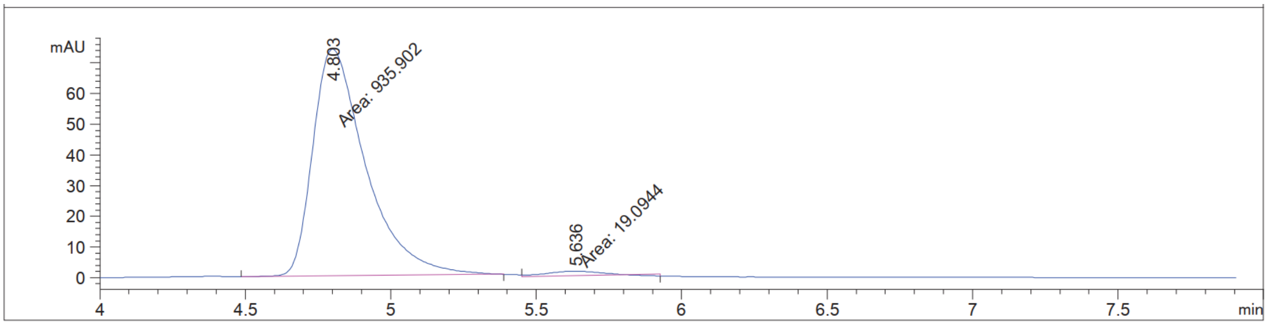

Peak #	RetTime [min]	Area [mAu*s]	Area %
**1**	**4.803**	**935.90161**	**98.0006**
2	5.636	19.09445	1.9994

(+)-**16** – starting material for (−)-**27:**

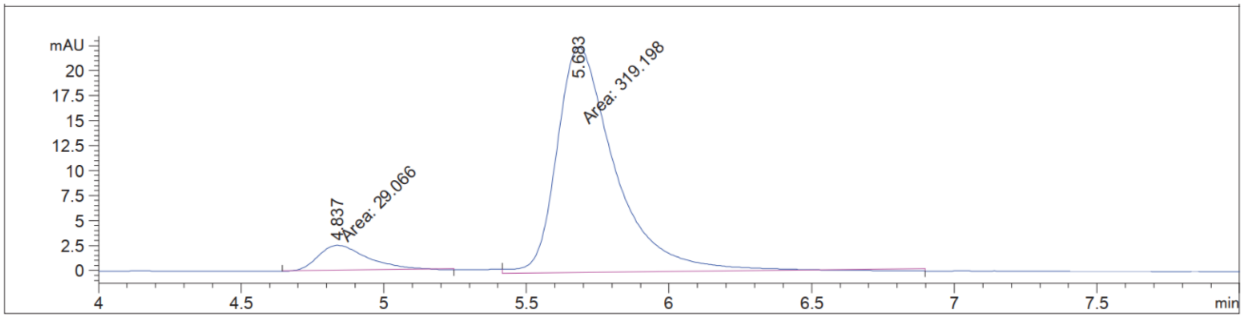

Peak #	RetTime [min]	Area [mAu*s]	Area %
1	4.837	29.06599	8.3460
**2**	**5.683**	**319.19760**	**91.6540**

Figure S65. Chiral chromatography data of compound 16. Daicel CHIRALPAK® IB column and 20% of isopropanol in hexanes as mobile phase (1.0 mL/min).

Table S1. Kinetic solubility of compound **19** and the positive control alprenolol following 0 and 1.5 h of incubation at 25 °C in aqueous buffers at pH 1.7 (acidic), 7.4 (neutral), and 8.9 (basic). Results are expressed as mean soluble fraction ± standard deviation.

Compound	pH	Time (h)	Mean soluble fraction ± SD (%)
**19**	1.7	0	99 ± 2
	7.4		82 ± 3
	8.9		64 ± 2
	1.7	1.5	96 ± 7
	7.4		81 ± 1
	8.9		65 ± 2
**Alprenolol**	1.7	0	92 ± 1
	7.4		96 ± 4
	8.9		88 ± 2
	1.7	1.5	91 ± 3
	7.4		93 ± 1
	8.9		85 ± 2

Table S2. Summary of the chemical stability and solubility of compound 19 and the positive control alprenolol after 0, 1.5, and 24 h of incubation at 37 °C in acidic (pH 1.7), neutral (pH 7.4), and basic (pH 8.9) aqueous buffers. Data are expressed as mean soluble fraction (% of initial concentration) ± standard deviation.

Compound	pH	Time (h)	Mean soluble fraction ± SD (%)
**19**	1.7	0	100 ± 1
	7.4		100 ± 2
	8.9		62 ± 3
	1.7	1.5	95 ± 1
	7.4		95 ± 3
	8.9		69 ± 3
	1.7	24	86 ± 3
	7.4		87 ± 2
	8.9		63 ± 3
**Alprenolol**	1.7	0	100 ± 8
	7.4		100 ± 1
	8.9		100 ± 2
	1.7	1.5	101 ± 2
	7.4		104 ± 7
	8.9		114 ± 3
	1.7	24	100 ± 2
	7.4		103 ± 7
	8.9		112 ± 3

Table S3. Predicted and experimental $LogD_{7.4}$ determined using the shake-flask method for **19** and reference compounds (tolbutamide and ketoconazole).

Compounds	Method	LogD at pH 7.4 ± SD
tolbutamide	Experimental	0.40 ± 0.02
	ACD or DM cLogD	0.47
	ACD or ChemDraw cLogP	2.93
ketoconazole	Experimental	3.67 ± 0.05
	ACD or DM cLogD	3.49
	ACD or ChemDraw cLogP	3.55
**19**	Experimental	3.9 ± 0.1
	DM cLogD	4.10
	ChemDraw cLogP	4.89

Table S4: *In vitro* metabolic stability parameters of **19** and verapamil (control) in liver microsomes from mouse, rat, and human.

Cpd	Parameters	Mouse	Rat	Human
**19**	$T_{1/2}$ (min)	151	182	204
	CL int. mic. (µL· min-1·mg-1)	0.92	0.76	0.68
	CL int. hep. (µL· min-1·kg-1)	3.73	1.37	0.61
	CLhep (mL· min-1·Kg-1)	0.69	0.255	0.11
**verapamil**	$T_{1/2}$ (min)	19	20	27
	CL int. mic. (µL· min-1·mg-1)	30	27	20
	CL int. hep. (µL· min-1·kg-1)	120	55	18
	CLhep (mL· min-1·Kg-1)	8	4	1

Table S5. Baseline characteristics of the isolates for the *ex vivo* assay.

Baseline characteristics	*P. vivax*	*P. falciparum*
Isolates reaching harvest *(% of total)*	10 (100)	7 (87.5)
Initial parasites/µL *mean (range)*	6,135 (3,930 – 10,250)	7,216 (2,100 – 11,200)
Duration of the assay *mean (range)*	47 h (42 – 51)	44 h (28 – 62)
Parasite at ring stage *mean (range)*	92% (87 – 96)	100% (98 – 100)
Schizont percentage at harvest *mean (range)*	50% (40 – 68)	51% (30 – 80)

Table S6: Outcomes of all FEP transformation steps in triplicate run of the **19**-FP2a complex.

Transformation	Complex (kcal/mol)			Ligand in Water (kcal/mol)			$\Delta\Delta G_{Total\_Predicted}$ (kcal/mol)
	$\Delta G_{decharge}$	$\Delta G_{recharge}$	ΔG_{vdW}	$\Delta G_{decharge}$	$\Delta G_{recharge}$	ΔG_{vdW}	
OMe -> H (t1)	19.7	9.0	22.4	19.5	9.1	20.8	1.7
OMe -> H (t2)	20.0	9.0	22.3	19.5	9.2	20.8	1.8
OMe -> H (t3)	19.7	9.0	22.6	19.4	9.2	20.8	1.9

REFERENCES

(1) Bray, B. L.; Mathies, P. H.; Naef, R.; Solas, D. R.; Tidwell, T. T.; Artis, D. R.; Muchowski, J. M. N-(Triisopropylsilyl)Pyrrole. A Progenitor “Par Excellence” of 3-Substituted Pyrroles. *J Org Chem* **1990**, *55* (26), 6317–6328. https://doi.org/10.1021/jo00313a019.

(2) Albrecht, F.; Sowada, O.; Fistikci, M.; Boysen, M. M. K. Heteroarylboronates in Rhodium-Catalyzed 1,4-Addition to Enones. *Org Lett* **2014**, *16* (19), 5212–5215. https://doi.org/10.1021/ol502630w.

(3) Billingsley, K.; Buchwald, S. L. Highly Efficient Monophosphine-Based Catalyst for the Palladium-Catalyzed Suzuki−Miyaura Reaction of Heteroaryl Halides and Heteroaryl Boronic Acids and Esters. *J Am Chem Soc* **2007**, *129* (11), 3358–3366. https://doi.org/10.1021/ja068577p.

(4) Verma, A. K.; Jha, R. R.; Kasi Sankar, V.; Singh, R. P. Selective Synthesis of 4,5-Dihydroimidazo- and Imidazo[1,5-a]Quinoxalines via Modified Pictet–Spengler Reaction. *Tetrahedron Lett* **2013**, *54* (45), 5984–5990. https://doi.org/10.1016/j.tetlet.2013.08.052.

(5) OECD. *OECD Guideline for the Testing of Chemicals. Test No. 107: Partition Coefficient (n-Octanol/Water): Shake Flask Method*; OECD Guidelines for the Testing of Chemicals, Section 1; OECD: Paris, 1995. https://doi.org/10.1787/9789264069626-en.

(6) U.S. Environmental Protection Agency (EPA). *Product Properties Test Guidelines OPPTS 830.7550 Partition Coefficient (n-Octanol/Water), Shake Flask Method*; Washington, DC, 1996.

(7) Hann, E.; Malagu, K.; Stott, A.; Vater, H. The Importance of Plasma Protein and Tissue Binding in a Drug Discovery Program to Successfully Deliver a Preclinical Candidate. *Prog Med Chem* **2022**, *61*, 163–214. https://doi.org/10.1016/bs.pmch.2022.04.002.

(8) Hartman, D. A. Determination of the Stability of Drugs in Plasma. *Curr Protoc Pharmacol* **2003**, *Chapter 7* (1), Unit 7.6. https://doi.org/10.1002/0471141755.ph0706s19.

(9) Waters, N. J.; Jones, R.; Williams, G.; Sohal, B. Validation of a Rapid Equilibrium Dialysis Approach for the Measurement of Plasma Protein Binding. *J Pharm Sci* **2008**, *97* (10), 4586–4595. https://doi.org/10.1002/jps.21317.

(10) Zhang, F.; Xue, J.; Shao, J.; Jia, L. Compilation of 222 Drugs’ Plasma Protein Binding Data and Guidance for Study Designs. *Drug Discov Today* **2012**, *17* (9–10), 475–485. https://doi.org/10.1016/j.drudis.2011.12.018.

(11) Knights, K. M.; Stresser, D. M.; Miners, J. O.; Crespi, C. L. In Vitro Drug Metabolism Using Liver Microsomes. *Curr Protoc Pharmacol* **2016**, *74*, 7.8.1-7.8.24. https://doi.org/10.1002/cpph.9.

(12) Obach, R. S.; Baxter, J. G.; Liston, T. E.; Silber, B. M.; Jones, B. C.; MacIntyre, F.; Rance, D. J.; Wastall, P. The Prediction of Human Pharmacokinetic Parameters from Preclinical and in Vitro Metabolism Data. *J Pharmacol Exp Ther* **1997**, *283* (1), 46–58.

(13) Obach, R. S. Predicting Clearance in Humans from in Vitro Data. *Curr Top Med Chem* **2011**, *11* (4), 334–339. https://doi.org/10.2174/156802611794480873.

(14) Vamsi Krishna, M.; Padmalatha, K.; Madhavi, G. *In Vitro* Metabolic Stability of Drugs and Applications of LC-MS in Metabolite Profiling. In *Drug Metabolism*; IntechOpen, 2021. https://doi.org/10.5772/intechopen.99762.
